# Unlocking Distinct Excited States of a Nickel(II) Complex Without Modulating the Primary Coordination Sphere

**DOI:** 10.1021/jacsau.6c00738

**Published:** 2026-08-13

**Authors:** Hailey Hendricks, Raina M. Morley, Angel Y. Gomez, Yokari Godínez-Loyola, Matthew V. Pecoraro, Gregory D. Scholes, Jose B. Roque

**Affiliations:** Department of Chemistry, Princeton University, Princeton, New Jersey 08544, United States

**Keywords:** transition metal, secondary coordination sphere, fluorenone, ligand design, bioinspired, photochemistry, photophysics

## Abstract

Traditional efforts in modulating the reactivity of metal centers focus on tuning parameters in the primary coordination sphere. The ability to embed stimuli-responsive functionality in the secondary coordination sphere to modulate metal center reactivity represents a distinct strategy for expanding transition metal reactivity while leaving the primary coordination sphere unmodified. Herein, we describe the incorporation of a photoresponsive unit, fluorenone, into the secondary coordination sphere of a Ni­(II) complex resulting in a significant perturbation of the complex electronic structure, manifesting in distinct excited states that cannot be predicted by the metal complex or fluorenone unit alone. Using the activation of strong nickel­(II) trifluoromethyl bonds as a readout of this strategy, the incorporation of latent ligand-centered radicals enabled near-quantitative generation of trifluoromethyl radical. Comparisons with a control complex lacking the appended fluorenone unit demonstrated the unique role of the appended fluorenone, showcasing the ability to endow photoreactivity to an otherwise unreactive complex. Mechanistic studies including TD-DFT calculations, cyclic voltammetry, and transient absorption spectroscopy support that irradiation with visible light leads to metal-to-ligand charge transfer followed by relaxation to a long-lived ^3^d–d state. The studies presented herein showcase a ligand design principle focused on secondary coordination sphere edits through the introduction of latent ligand-centered radicals, allowing for the direct tuning of the metal center reactivity.

## Graphical abstract

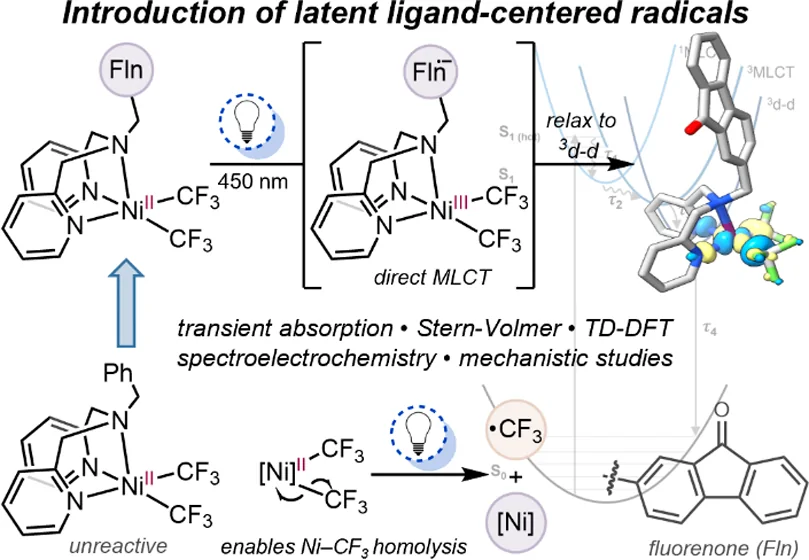

## Introduction

Given the critical role transition metals play in modern society, discovering strategies to unlock new reactivity by modulation of metal centers is imperative.^1^ Traditional approaches to improve the reactivity and selectivity of such transition metal complexes center around fine-tuning the steric and electronic properties of the supporting ligands directly bound to the metal center, known as the *primary* coordination sphere.^2–6^ Despite a limited set of ligands available within biological systems, a wealth of reactivity can be attributed to harnessing noncovalent interactions in the *secondary* coordination sphere (SCS), that is, the ligand environment not directly bound to the metal center.^7–10^ Inspired by nature’s enzymes, efforts have been directed toward the development of molecular complexes that incorporate functional elements into the SCS, such as hydrogen bond donors and acceptors, and Lewis acidic and basic sites (Figure 1A).^11–17^ Our research group focuses on the design of molecular systems that are not constrained to noncovalent interactions in the SCS but instead leverage the generation of open-shell systems upon the introduction of external stimuli.

**Figure 1:**
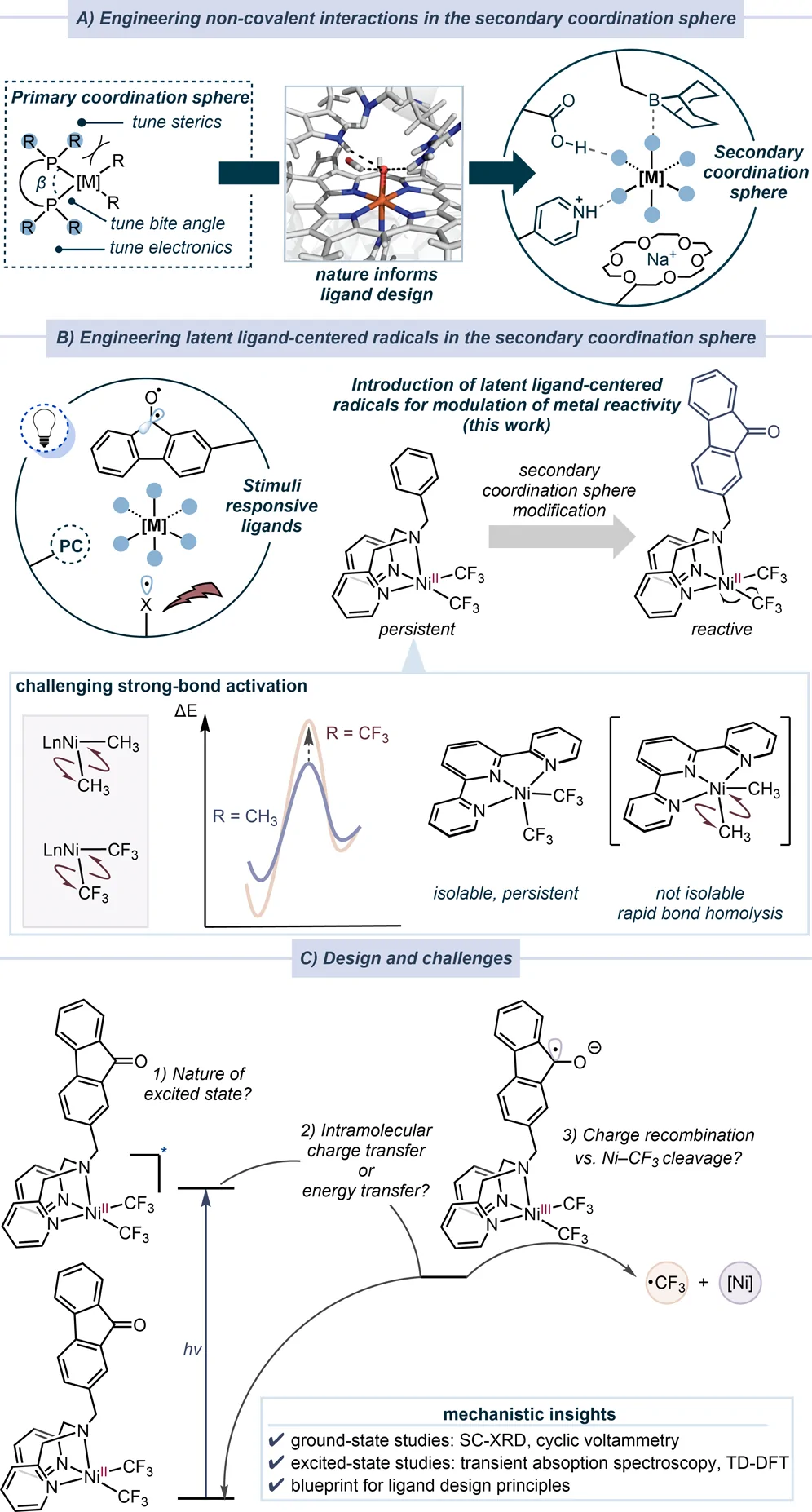
(A) Selected examples of secondary coordination sphere modifications in homogeneous transition metal catalysts.^9,12a,13^ (B) Incorporation of latent ligand-centered radicals in the secondary coordination sphere (this work) and properties of nickel-perfluoroalkyls.^42^ (C) Design and challenges of proposed concept.

Specifically, by engineering photoresponsive units whose functionality is unlocked through the application of an external stimulus, we can access an open-shell species in close proximity to the metal center. These latent ligand-centered radicals can modulate the reactivity of the metal center without being directly bound to it and instead exist in the ligand periphery (Figure 1B). This strategy decouples the function of the primary coordination sphere from appended functionality in the secondary coordination sphere, endowing photoreactivity to otherwise unreactive complexes. Under this regime, opportunities can arise for electronic coupling between the metal center and the appended functionality resulting in unexpected electronic transitions and altered excited-state manifolds that are not present in either component alone. Despite progress in designing bifunctional transition metal complexes, the mechanistic underpinnings and the underlying photophysical properties remain largely elusive, making it difficult to predict the nature of the excited state *a priori*.^18–32^ By accessing well-defined complexes with appended photoresponsive units in the SCS and evaluating their photophysical properties, we aim to deepen mechanistic insight and explore the potential of this design principle.

To evaluate this concept, our efforts centered on appending a photoresponsive diaryl ketone into the SCS of a metal complex. Diaryl ketones represent an attractive class of photoresponsive molecules, as they are inexpensive, readily available, and have well-studied photophysical properties.^33,34^ Specifically, we chose fluorenone for multiple reasons: (1) fluorenone exhibits highly efficient intersystem crossing (ISC) to its long-lived triplet state (Φ_ISC_ = 0.97 in acetonitrile); (2) it has a high triplet energy (55 kcal/mol); and (3) it is highly oxidizing in its triplet excited state (*E*_1/2_^*^ = 0.57 vs Fc/Fc^+^ in acetonitrile). These characteristics make fluorenone an ideal candidate to evaluate our design principle.^33–38^ With this design, we anticipated that the appended fluorenone could intramolecularly oxidize the metal center upon photoirradiation. To probe the oxidation of the metal center through latent ligand-centered radicals, we pursued strong metal–alkyl bond homolysis as a readout of this concept.

Nickel–trifluoromethyl bonds represent a compelling and challenging target for evaluating this strategy due to their enhanced persistence relative to nonfluorinated metal–alkyl analogs and their lack of reactivity in the Ni­(II) oxidation state (Figure 1B).^39–49^ We anticipated several challenges with this approach for strong-bond activation that influenced our ligand design. The photophysical properties of the fluorenone-appended transition metal complex are unknown, making it difficult to predict the exact nature of the excited states *a priori* (Figure 1C). Next, the distance between the fluorenone unit and the metal center becomes critical as the rate of electron transfer has an exponential dependence on the distance between the donor and acceptor.^50,51^ Finally, the rate of Ni–CF_3_ bond homolysis could be slower than charge recombination from the excited state, precluding generation of trifluoromethyl radical (Figure 1C).^25,52–55^ Considering these points, we targeted a tridentate bis­(pyridin-2-ylmethyl)­amine (bpma) scaffold, which allowed us to readily synthesize a fluorenone-appended analogue. These dipyridyl systems, featuring a pendant nitrogen, are expected to bind in a κ^3^ fashion and anchor the photoresponsive unit in proximity to the metal center.

Herein, we report the synthesis, reactivity, and insights into the photophysical properties of well-defined bpma bis­(trifluoromethyl)­nickel­(II) complexes with an appended photoresponsive motif, fluorenone. Mechanistic insights provided by cyclic voltammetry, computational calculations, and transient absorption spectroscopy demonstrate unexpected photophysical properties that are absent in the unreactive control nickel complex or the free photoresponsive unit. This work demonstrates the advantages and unique reactivity endowed by introducing a photoresponsive motif into the SCS of a metal center, leading to the activation of strong metal–ligand bonds at formally lower oxidation states without the need for external reagents. More broadly, these results demonstrate that latent ligand-centered radicals represent a distinct strategy for generating new excited-state manifolds in homogeneous transition metal complexes through remote secondary coordination sphere modifications.

## Results and Discussion

### Synthesis of Bis­(trifluoromethyl) Nickel­(II) Complexes

To evaluate the proposed concept, the synthesis of well-defined nickel­(II) complexes bearing appended stimuli-responsive units was pursued. The fluorenone-appended bpma ligand, **(** ^**Fluor**^ **N**_**3**_ **)**, was prepared in two steps (see Supporting Information for details).^56,57^ The fluorenone-appended bpma bis­(trifluoromethyl)­nickel­(II) complex, **(** ^**Fluor**^ **N**_**3**_ **)­Ni­(CF**_**3**_ **)**_**2**_, was synthesized by treatment of the ligand **(** ^**Fluor**^ **N**_**3**_ **)** with a nickel­(II) precursor, **(MeCN)**_**2**_ **Ni­(CF**_**3**_ **)**_**2**_, in THF to afford the target complex in 91% yield (Figure 2) (see Supporting Information for experimental details).^42^ To evaluate the impact of tethering the photoresponsive motif to the ligand framework of the bpma bis­(trifluoromethyl)­nickel­(II) complexes, the *N*-benzylated bpma ligand **(** ^**Bn**^ **N**_**3**_ **)** was treated with **(MeCN)**_**2**_ **Ni­(CF**_**3**_ **)**_**2**_ in THF to afford **(** ^**Bn**^ **N**_**3**_ **)­Ni­(CF**_**3**_ **)**_**2**_ in a 67% yield. The synthesized bis­(trifluoromethyl)­nickel­(II) complexes exhibited diamagnetic ground states and were characterized by ^1^H, ^19^F, and ^13^C NMR spectroscopy. ^19^F NMR analysis of **(** ^**Fluor**^ **N**_**3**_ **)­Ni­(CF**_**3**_ **)**_**2**_ and **(** ^**Bn**^ **N**_**3**_ **)­Ni­(CF**_**3**_ **)**_**2**_ revealed a single resonance for the trifluoromethyl group in benzene-*d*_6_ at −24.80 and −24.79 ppm, respectively, consistent with previously reported nickel­(II) trifluoromethyl complexes (^19^F NMR referenced to hexafluorobenzene at −162.82 ppm as internal standard).^42,58–63^

**Figure 2:**
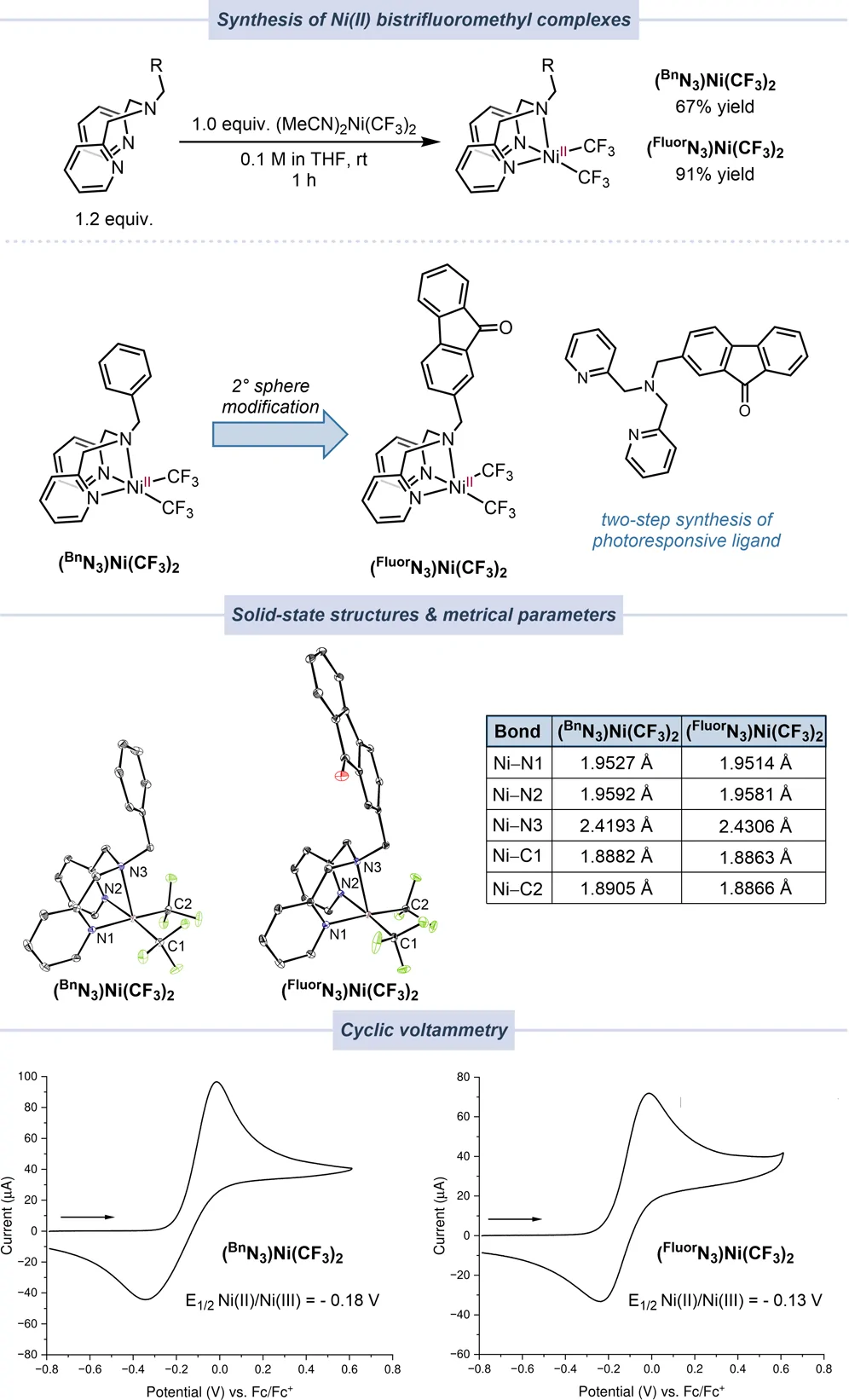
Synthesis of Ni­(II) bistrifluoromethyl complexes with corresponding metrical parameters from XRD structures. Cyclic voltammograms of **(** ^**Bn**^ **N**_**3**_ **)­Ni­(CF**_**3**_ **)**_**2**_ and **(** ^**Fluor**^ **N**_**3**_ **)­Ni­(CF**_**3**_ **)**_**2**_ in acetonitrile. Conditions: [Ni] = 0.075 M, [NBu_4_PF_6_] = 0.1 M, [AgNO_3_] = 0.01 M; scan rate = 100 mV/s.

Crystals of **(** ^**Fluor**^ **N**_**3**_ **)­Ni­(CF**_**3**_ **)**_**2**_ suitable for analysis by single-crystal X-ray diffraction (XRD) were obtained by recrystallization out of a saturated acetonitrile solution at −35 °C. The solid-state structure of **(** ^**Fluor**^ **N**_**3**_ **)­Ni­(CF**_**3**_ **)**_**2**_ revealed a square pyramidal geometry (τ_5_ = 0.07) around the Ni center (Figure 2).^64^ The axial Ni–N3 bond distance [2.4306 (13) Å] is significantly longer than the equatorial Ni–N1 and Ni–N2 bond distances [1.9514 (13) and 1.9581 (13) Å, respectively]. Compared to the van der Waals radii of Ni and N atoms (1.63 Å and 1.55 Å, respectively), the length of the Ni–N3 bond supports covalency. The Ni–CF_3_ bond distances of 1.8863 (16) Å and 1.8866 (16) Å are similar to previously reported Ni–CF_3_ bond distances.^42,58–63^ While carbonyl groups can coordinate to metal centers in an η^2^ fashion, our ligand architecture prevents this coordination mode. Beyond XRD analysis, the unbound nature of the carbonyl group was further corroborated by the distinctive ^13^C NMR peak at 193.27 ppm and ATR-IR peak at 1711.06 cm^–1^, compared to the free ligand peaks at 194.14 ppm and 1710.58 cm^–1^ (see Supporting Information for details and additional computational modeling).^65^ Crystals of **(** ^**Bn**^ **N**_**3**_ **)­Ni­(CF**_**3**_ **)**_**2**_ also revealed a square pyramidal geometry around the Ni center (τ_5_ = 0.05) and similar bond distances were observed (Figure 2). Notably, the nearly identical metrical parameters of the Ni–CF_3_, axial Ni–N3, and equatorial Ni–N bond distances across the complexes indicate that the structural features of the primary coordination sphere remain unchanged.

### Cyclic Voltammetry Experiments

To determine the influence of SCS modification on the redox properties of the metal complex, cyclic voltammetry (CV) experiments were conducted on the **(** ^**Fluor**^ **N**_**3**_ **)­Ni­(CF**_**3**_ **)**_**2**_ and **(** ^**Bn**^ **N**_**3**_ **)­Ni­(CF**_**3**_ **)**_**2**_ complexes (Figure 2). The potentials of the complexes were found to be similar despite SCS modifications: (**(** ^**Fluor**^ **N**_**3**_ **)­Ni­(CF**_**3**_ **)**_**2**_ *E*_1/2_ Ni­(II)/Ni­(III) = −0.13 V vs Fc/Fc^+^; **(** ^**Bn**^ **N**_**3**_ **)­Ni­(CF**_**3**_ **)**_**2**_ *E*_1/2_ Ni­(II)/Ni­(III) = −0.18 V vs Fc/Fc^+^).^66^ Fluorenone in its excited state (*E*_1/2_^*^ = 0.57 vs Fc/Fc^+^) is sufficiently oxidizing to oxidize the metal center, consistent with our mechanistic proposal.^33a^ If this intramolecular oxidation takes place to generate an oxidized metal center and appended ketyl radical anion, the resulting ketyl radical anion is highly reducing (*E*_1/2_ fluorenone/fluorenone^•‑^ = −1.70 V vs Fc/Fc^+^) (Figure S2). This species could therefore facilitate a back electron transfer event to reduce Ni­(III) to Ni­(II), one of the challenges previously mentioned if charge recombination significantly outcompetes bond homolysis.^50–52,67^ Appending fluorenone to the bpma ligand scaffold was not found to influence the reduction potential of fluorenone (*E*_1/2_^Fluor^N_3_/^Fluor^N_3_^•–^ = −1.70 V vs Fc/Fc^+^) (Figure S3).

Taken together, the results from XRD analysis and CV studies support that the primary coordination sphere is unchanged upon the introduction of fluorenone into the SCS.

### Reactivity of Bis­(trifluoromethyl) Ni­(II) Complexes under Thermal Heating and 405 nm Irradiation

After synthesizing the bis­(trifluoromethyl)­nickel­(II) complexes, their reactivity was examined under thermal heating and visible light irradiation. A solution of **(** ^**Fluor**^ **N**_**3**_ **)­Ni­(CF**_**3**_ **)**_**2**_ in benzene revealed no conversion after 24 h in the dark, with quantitative recovery of starting material (Figure S13). Likewise, heating the solution to 60 °C for 12 h yielded no product formation and 96% recovery of starting material (Figures 3A and S17). Similar persistence was observed for **(** ^**Bn**^ **N**_**3**_ **)­Ni­(CF**_**3**_ **)**_**2**_ under thermal conditions, highlighting the challenge of activating nickel­(II) trifluoromethyl bonds (Figures 3A and S14 and S18).^39–44^ In order to trap any CF_3_ radical generated and gain insights into potential reversible bond homolysis, the reactivity of the bis­(trifluoromethyl)­nickel­(II) complexes was evaluated in the presence of 2,2,6,6-tetramethylpiperidine-1-oxyl (TEMPO) at room temperature and 60 °C (see Supporting Information for details). No conversion to the TEMPO–CF_3_ adduct was observed for the complexes at either temperature (Figures 3A and S15, S16, S19, S20).

**Figure 3:**
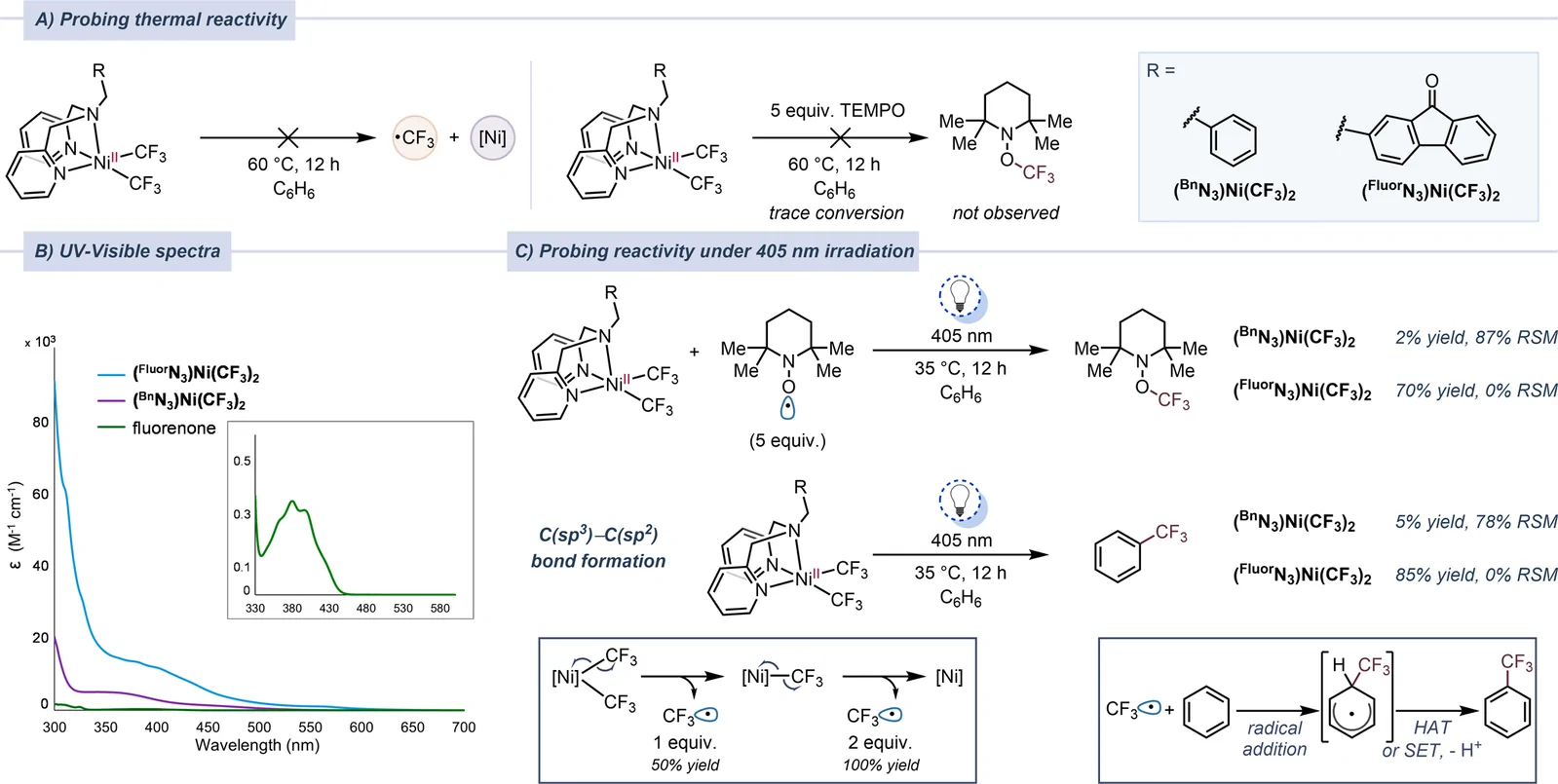
(A) Thermal reactivity of bis­(trifluoromethyl)­nickel­(II) complexes with and without TEMPO (B) UV–visible spectra of bis­(trifluoromethyl)­nickel­(II) complexes and fluorenone; inset shows zoomed in spectrum of fluorenone. (C) Photoreactivity of bis­(trifluoromethyl)­nickel­(II) complexes with and without TEMPO under 405 nm irradiation. RSM refers to recovered starting material. Yield >50% defined as activation of both Ni–CF_3_ bonds. Trifluorotoluene generated via radical addition to solvent molecule of benzene, followed by HAT or sequential SET and deprotonation.

After establishing the lack of thermal reactivity, the complexes’ persistence under visible light irradiation was examined. Given the prominent absorption features of the bpma bis­(trifluoromethyl)­nickel­(II) complexes around 400 nm (Figure 3B), their persistence was evaluated under 405 nm irradiation in the presence of five equivalents of TEMPO (Figure 3C). After irradiation with 405 nm light for 12 h, analysis of the **(** ^**Fluor**^ **N**_**3**_ **)­Ni­(CF**_**3**_ **)**_**2**_ reaction mixture by ^19^F NMR revealed generation of the TEMPO–CF_3_ adduct in a 70% yield (Figures 3C and S23). In contrast, analysis of the **(** ^**Bn**^ **N**_**3**_ **)­Ni­(CF**_**3**_ **)**_**2**_ reaction mixture by ^19^F NMR revealed a 2% yield of the TEMPO–CF_3_ adduct, highlighting unique reactivity achieved by appending the fluorenone motif in the SCS of the nickel complex (Figures 3C and S24). A yield greater than 50% is defined as homolysis of both Ni–CF_3_ bonds, yielding two equivalents of CF_3_ radical (Figure 3C).

To further evaluate the activation of persistent Ni–CF_3_ bonds, radical trapping with benzene was probed (Figure 3C).^49b,68–71^ The complexes were dissolved in benzene and exposed to 405 nm light in the absence of TEMPO. Indeed, irradiation of **(** ^**Fluor**^ **N**_**3**_ **)­Ni­(CF**_**3**_ **)**_**2**_ in benzene led to an 85% yield of trifluorotoluene, while **(** ^**Bn**^ **N**_**3**_ **)­Ni­(CF**_**3**_ **)**_**2**_ led to only a 5% yield, further showcasing stark differences in the reactivity of the complexes (Figures 3C and S21 and S22). Although the identity of the postreaction [Ni] complex could not be verified, the reaction mixture was filtered through a silica plug to decompose residual metal intermediates and assess the integrity of the ligand. High-resolution mass spectrometry confirmed the presence of the intact free ^**Fluor**^ **N**_**3**_ ligand, indicating that during the course of the reaction, the complex does not undergo significant decomposition or promote unproductive side reactions.

Based on the potentials provided by cyclic voltammetry experiments, the excited fluorenone is sufficiently oxidizing to remove an electron from the Ni­(II) complexes via SET.^33a,66^ To investigate this hypothesis and determine the importance of the intramolecular nature of the photoresponsive unit, exogenous fluorenone was added to the reaction of **(** ^**Bn**^ **N**_**3**_ **)­Ni­(CF**_**3**_ **)**_**2**_ under 405 nm irradiation, resulting in a 7% yield of trifluorotoluene with 85% recovered starting material (Figures 4 and S26). The reactivity of **(** ^**Bn**^ **N**_**3**_ **)­Ni­(CF**_**3**_ **)**_**2**_ was not significantly altered by the addition of exogenous fluorenone, exemplifying the importance of tethering the photoresponsive unit in the SCS. Incorporation into the ligand framework presumably enhances the rate of electron transfer and mitigates challenges associated with diaryl ketones, such as their low extinction coefficients and limited photostability, which constrain their photocatalytic potential and necessitate high catalyst loadings.^33c,38^ Observing similar reactivity of **(** ^**Bn**^ **N**_**3**_ **)­Ni­(CF**_**3**_ **)**_**2**_ with and without exogenous fluorenone, we questioned if the metal complex was quenching the excited state of fluorenone. Accordingly, steady-state Stern–Volmer quenching studies were conducted, which revealed no significant electron transfer to fluorenone from **(** ^**Bn**^ **N**_**3**_ **)­Ni­(CF**_**3**_ **)**_**2**_ (Figure 4, see Supporting Information for details).^72–75^

**Figure 4:**
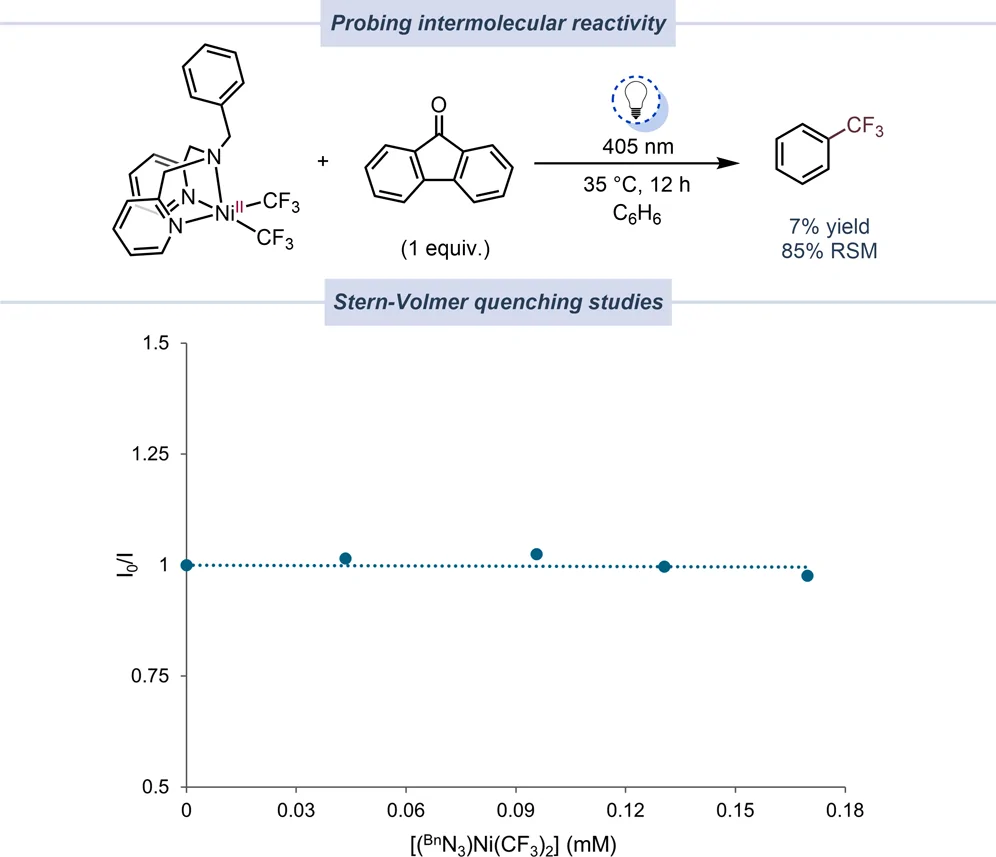
Reactivity of **(** ^**Bn**^ **N**_**3**_ **)­Ni­(CF**_**3**_ **)**_**2**_ with exogenous fluorenone and subsequent Stern–Volmer quenching study with fluorenone and **(** ^**Bn**^ **N**_**3**_ **)­Ni­(CF**_**3**_ **)**_**2**_ in benzene.

Taken together, these reactivity studies support our hypothesis and ligand design principle that by tethering a photoresponsive unit into the SCS of an otherwise unreactive Ni­(II) complex, we can promote previously inaccessible reactivity.

### Transient Absorption & Computational Insights

To further understand the inherent photophysical behavior of bpma bis­(trifluoromethyl)­nickel­(II) complexes, transient absorption spectroscopy (TA) was performed. To gain insight into the excited state spectroscopic features of the appended fluorenone unit, transient absorption spectroscopy was carried out on free fluorenone with a 400 nm pump excitation. In line with literature precedent, fluorenone exhibited strong excited state absorption (ESA) features that do not decay within the lifetime of the measurement (>7 ns) (Figure 5A). Evaluation of the collected transient absorption spectra matched well with the known photophysical properties of fluorenone, validating our experimental setup (Figure S31).^34,73–77^ The fluorenone-appended ligand ^**Fluor**^ **N**_**3**_ was studied under the same conditions and behaved similarly, with excited state absorption features outlasting the measurement lifetime (Figure S33). Next, **(** ^**Fluor**^ **N**_**3**_ **)­Ni­(CF**_**3**_ **)**_**2**_ was studied using a 400 nm pump excitation. Notably, the spectral signal of **(** ^**Fluor**^ **N**_**3**_ **)­Ni­(CF**_**3**_ **)**_**2**_ at this excitation wavelength resembles that of free fluorenone, both exhibiting excited state absorption peaks at 340 and 493 nm (Figure 5B). Despite similar spectral features, the signal lifetime for **(** ^**Fluor**^ **N**_**3**_ **)­Ni­(CF**_**3**_ **)**_**2**_ decays within 2 ns, significantly shorter than that of free fluorenone (Figure 5A,B). This significant decrease in excited-state lifetime suggests that the photophysical decay pathways of fluorenone, when appended to the metal complex, differ from those of free fluorenone.

**Figure 5:**
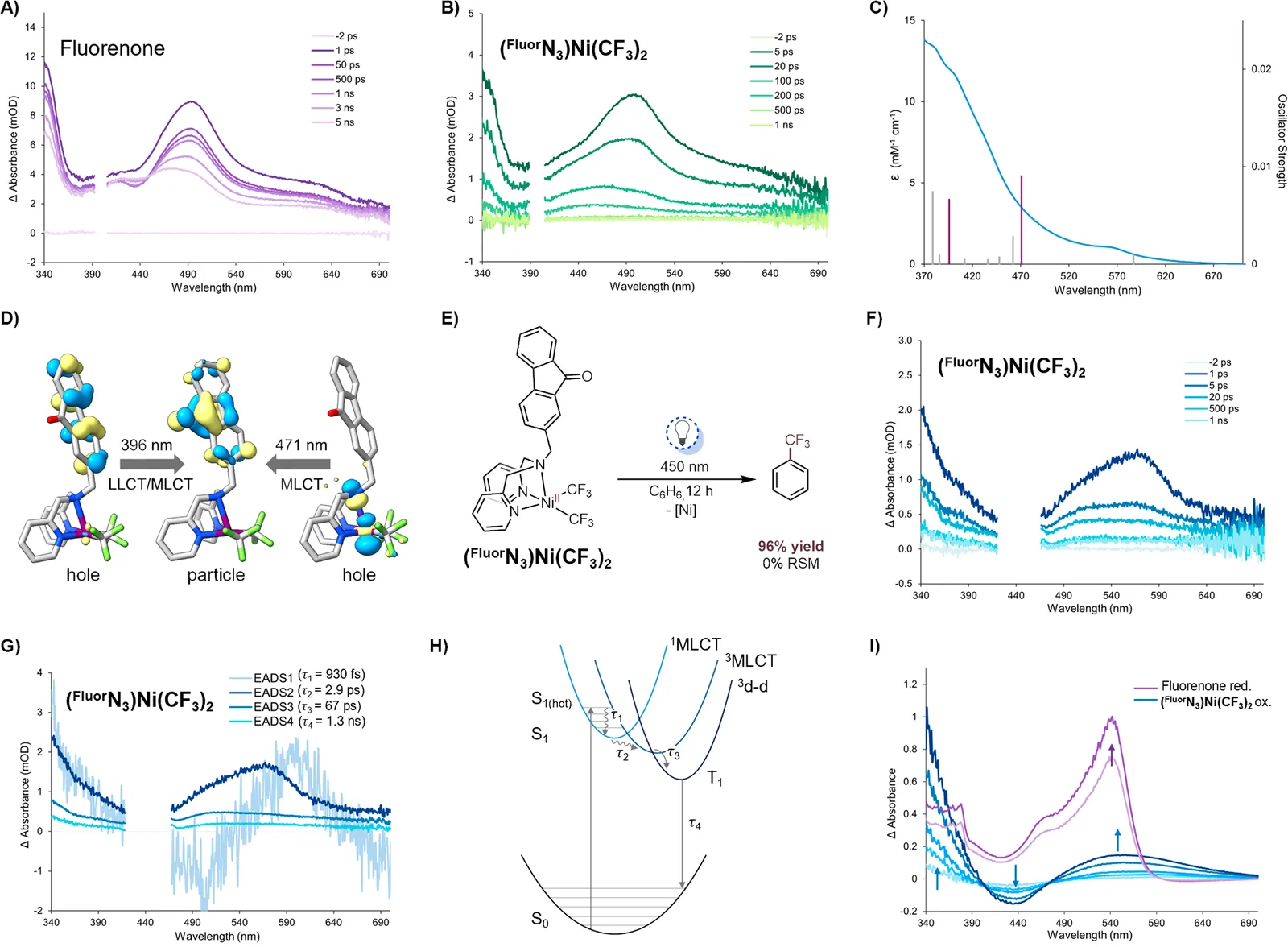
Transient absorption spectra of (A) free fluorenone and (B) **(** ^**Fluor**^ **N**_**3**_ **)­Ni­(CF**_**3**_ **)**_**2**_ excited with a 400 nm pump pulse in a 1:1 mixture of benzene:1,2-difluorobenzene. (C) Experimental UV–visible spectra of **(** ^**Fluor**^ **N**_**3**_ **)­Ni­(CF**_**3**_ **)**_**2**_ in benzene; computationally calculated electronic transitions overlaid in gray, with dominant transitions highlighted in purple. (D) NTO hole and particle visualizations of selected electronic transitions. (E) Evaluation of complex reactivity under 450 nm irradiation. (F) Transient absorption spectra of **(** ^**Fluor**^ **N**_**3**_ **)­Ni­(CF**_**3**_ **)**_**2**_ excited with a 430 nm pump pulse in a 1:1 mixture of benzene:1,2-difluorobenzene. (G) EADS extracted from transient absorption spectra (F). (H) Qualitative Jablonski diagram of proposed mechanism. (I) Difference spectrum resulting from (blue) electrochemical oxidation of **(** ^**Fluor**^ **N**_**3**_ **)­Ni­(CF**_**3**_ **)**_**2**_ in acetonitrile over the course of 20 s and (purple) electrochemical reduction of free fluorenone in acetonitrile over the course of 3 s.

To probe the nature of the excited states of the complex, time-dependent density functional theory (TD-DFT) calculations were performed at the M06-def2-tzvp level of theory (Figure 5C).^78–80^ Analysis of the electronic transitions present in the **(** ^**Fluor**^ **N**_**3**_ **)­Ni­(CF**_**3**_ **)**_**2**_ complex revealed an electronic transition at 396 nm (Figure 5D). Visualization of the natural transition orbitals (NTOs) involved in this excitation revealed a donor NTO with metal d and appended fluorenone π character. Analysis of the acceptor NTO showed population of molecular orbitals centered on the appended fluorenone π* system. Taken together, the electronic transition at 396 nm can be assigned as a mixed state comprised of some metal-to-ligand charge transfer (MLCT) and majority ligand-to-ligand charge transfer (LLCT) character.^25,26,53^ The ligand-centered π–π* transition is analogous to that of free fluorenone, which accounts for the similar spectral features observed in the transient absorption measurements (Figure 5A,B).

Most notably, further analysis of the dominant electronic transitions present in the **(** ^**Fluor**^ **N**_**3**_ **)­Ni­(CF**_**3**_ **)**_**2**_ complex revealed a strong transition at 471 nm (Figure 5D), well outside the window of absorption for free fluorenone (Figure 3B). Visualization of the involved NTOs revealed donor orbitals with Ni d_*z* ^2^_ character and acceptor orbitals localized on the appended fluorenone π* system. This transition, unlike the mixed state at 396 nm, is purely MLCT in nature.^25,26,53^ We questioned whether irradiation at this wavelength, thereby directly populating this MLCT state, could better promote the generation of trifluoromethyl radical. Accordingly, **(** ^**Fluor**^ **N**_**3**_ **)­Ni­(CF**_**3**_ **)**_**2**_ was dissolved in benzene and subjected to 450 nm irradiation for 12 h. Analysis of the reaction mixture by ^19^F NMR revealed a 96% yield of trifluorotoluene and full consumption of the starting material (Figures 5E and S44). When **(** ^**Bn**^ **N**_**3**_ **)­Ni­(CF**_**3**_ **)**_**2**_ was subjected to the same conditions, analysis of the reaction mixture revealed only a 9% yield of trifluorotoluene with 84% recovered starting material, highlighting the differences in reactivity under 450 nm irradiation (Figure S45).

To study the nature of this MLCT excited state, transient absorption spectroscopy of **(** ^**Fluor**^ **N**_**3**_ **)­Ni­(CF**_**3**_ **)**_**2**_ was conducted with a 430 nm pump excitation. By shifting the excitation wavelength to 430 nm, we hypothesized that we could avoid population of the mixed MLCT/LLCT excited state and gain insights into the pure MLCT state predicted by TD-DFT calculations. When exciting free fluorenone at 430 nm, the characteristic ligand-centered peak at 493 nm is still present (Figure S35). Alternatively, excitation of **(** ^**Fluor**^ **N**_**3**_ **)­Ni­(CF**_**3**_ **)**_**2**_ at 430 nm produced spectral signals different from those observed when exciting at 400 nm, exhibiting a red-shifted excited-state absorption feature. This spectral profile is notably missing the characteristic fluorenone ESA peak at 493 nm, suggesting that we are no longer populating a ligand-localized transition and instead have populated the pure MLCT state (Figure 5F). Overlapping excited-state absorption features in the 480–600 nm spectral window of Figure 5F complicate extraction of the dynamics of each component. Therefore, global analysis was applied to more clearly resolve these kinetics. Global analysis is employed to deconvolute systems with overlapping spectral features, allowing for the extraction of the distinct kinetic components contributing to the dynamics.^81–86^ A four-component kinetic scheme provided the best fit to the experimental data. The number of distinguishable components contributing to the dynamics was determined on the basis of root-mean-square error values and residuals (details of analysis are described in the Supporting Information, Figures S32 and S39.^81–86^ Evolution-associated difference spectra (EADS), displayed in Figure 5G, were generated by global analysis to monitor the evolution of the **(** ^**Fluor**^ **N**_**3**_ **)­Ni­(CF**_**3**_ **)**_**2**_ excited-state absorption.

The individual features and lifetimes of each EADS trace provide insight into the excited state decay mechanism. Upon excitation, a hot S_1_ state is populated that relaxes after 930 fs into the proposed ^1^MLCT S_1_ state, represented by EADS1. This assignment aligns well with typical transition metal MLCT systems, as we observe strong ESA in the 330–380 nm region corresponding to population of the ligand-centered π* system.^53,87–93^ Additional excited state absorption features partially overlap the ground state bleach signal around 490 nm.

These features, along with the subpicosecond lifetime of the trace, indicate that the system has not yet fully relaxed vibrationally, consistent with a nonequilibrated MLCT state.^53,73,94^ The ^1^MLCT state undergoes intersystem crossing on a 2.9 ps time scale, represented by EADS2, to a proposed ^3^MLCT state. This assignment is supported by the retention of ESA in the 330–380 nm region, along with the appearance of ESA in the 480–560 nm region. These changes, along with the time scale, are characteristic of intersystem crossing and suggest the formation of a triplet MLCT.^53,90,93–95^

We then propose that the ^3^MLCT state decays over 67 ps to a lower energy ^3^d–d state, which decays back to the S_0_ state (Figure 5H). This assignment is supported by the spectral profile of the EADS3 component, where most of the visible ESA has decayed and weak residual signal remains, suggesting conversion out of the MLCT manifold.^53,90,93^ Relaxation from a higher-energy MLCT state (EADS3) into a lower-lying d–d state is expected to exhibit comparatively weaker transient absorption features, as metal-centered states can act as dark excited-state traps and often display reduced ESA intensity relative to charge-transfer states within the measured probe window.^96–99^ This is consistent with the stark decrease in signal intensity. Notably, the final state has a lifetime on the nanosecond time scale represented by EADS4 (1.27 ns), in line with literature reports on ^3^d–d lifetimes for nickel complexes.^25,53,93,100^ These assignments are supported by further experiments (*vide infra*) and are consistent with existing transient absorption studies on nickel complexes.^25,53,93,100^

In summary, transient absorption spectroscopy revealed that the absorption features characteristic of free fluorenone are absent in the spectra of **(** ^**Fluor**^ **N**_**3**_ **)­Ni­(CF**_**3**_ **)**_**2**_ upon excitation with a 430 nm pump pulse (see the Supporting Information for experimental details). When combined with the results from TD-DFT, which revealed transitions originating from metal-centered orbitals and terminating in ligand orbitals localized on the fluorenone unit in the SCS (i.e., a new MLCT state), these spectra highlight a significant perturbation of the electronic structure. This behavior indicates a substantially more complex excited state manifold than would be expected for a minimally perturbed, ligand-localized excited state.

### Insights Into MLCT

To further support the assignment of EADS2 as relaxation of a ^1^MLCT state, spectroelectrochemistry experiments were conducted on free fluorenone and **(** ^**Fluor**^ **N**_**3**_ **)­Ni­(CF**_**3**_ **)**_**2**_ in acetonitrile. In an MLCT transition, the metal center is formally oxidized and the ligand reduced. To mimic this, the **(** ^**Fluor**^ **N**_**3**_ **)­Ni­(CF**_**3**_ **)**_**2**_ complex was electrochemically oxidized through bulk electrolysis (120 mV) and its absorption profile monitored over 20 s (Figures 5I and S47). Spectral features resulting from this oxidation include a bleach centered at 430 nm and a broad growth centered at 540 nm. Similarly, free fluorenone was electrochemically reduced (−1800 mV) to the fluorenone ketyl radical anion, giving rise to a sharp signal with a λ_max_ of 550 nm (Figures 5I and S46). Overlaid together, the spectroelectrochemistry experiments reveal absorption features that resemble those of EADS1 and EADS2. These experiments support the identity of EADS1 as vibrational relaxation of the hot S_1_ MLCT state to the relaxed S_1_^1^MLCT state, and EADS2 as the relaxation of the ^1^MLCT state to a ^3^MLCT state.

To probe the nature of metal–ligand bonding during this MLCT transition in which the metal has been formally oxidized, we set out to synthesize a well-defined Ni­(III) d^7^ species. Treatment of **(** ^**Fluor**^ **N**_**3**_ **)­Ni­(CF**_**3**_ **)**_**2**_ with one equivalent of AgBF_4_ in C_6_H_6_ led to an intractable mixture containing trifluorotoluene as determined by ^1^H and ^19^F NMR spectroscopy. As a result, a preparative scale synthesis of a well-defined Ni­(III) complex was pursued. Treatment of **(** ^**Fluor**^ **N**_**3**_ **)­Ni­(CF**_**3**_ **)**_**2**_ with one equivalent of AgBF_4_ in MeCN at −35 °C led to an immediate color change from pale orange to deep maroon, consistent with previously reported Ni­(III) species (Figure 6A).^43,44^ ^1^H NMR analysis revealed the formation of a new paramagnetic species suggestive of a single electron oxidation taking place. Concentration and filtration afforded the Ni­(III) complex in an 86% yield. Single crystals of **[(** ^**Fluor**^ **N**_**3**_ **)­Ni­(CF**_**3**_ **)**_**2**_ **(MeCN)]­BF**_**4**_ suitable for analysis by XRD were obtained by recrystallization from a saturated acetonitrile solution at −35 °C. The solid-state structure of **[(** ^**Fluor**^ **N**_**3**_ **)­Ni­(CF**_**3**_ **)**_**2**_ **(MeCN)]­BF**_**4**_ revealed an octahedral geometry around the Ni center with the apical positions of the octahedron occupied by the pendant amine and acetonitrile solvent. The Ni–CF_3_ bond distances of 1.957 (2) and 1.950 (2) Å are longer than the bond distances found in **(** ^**Fluor**^ **N**_**3**_ **)­Ni­(CF**_**3**_ **)**_**2**_ (1.8863 (16) and 1.8866 (16) Å), highlighting the impact of one-electron oxidation on the metal–ligand bond lengths. In the square pyramidal d^8^ Ni­(II) complex **(** ^**Fluor**^ **N**_**3**_ **)­Ni­(CF**_**3**_ **)**_**2**_, two electrons populate the e_g_ set in the d_*z*_^2^ molecular orbital, which is antibonding in nature, and the d_*x* ^2^–*y* ^2^_ molecular orbital remains unoccupied. Upon oxidation to Ni­(III) and binding of acetonitrile, the complex distorts to an octahedral d^7^ complex, as evidenced by the XRD structure (Figure 6A). The addition of the axial acetonitrile ligand introduces a second σ-donor interaction along the *z*-axis, raising the energy of the d_*z* ^2^_ orbital, while the d_*x* ^2^–y^2^_ orbital is largely unaffected. As a result, in this **[(** ^**Fluor**^ **N**_**3**_ **)­Ni­(CF**_**3**_ **)**_**2**_ **(MeCN)]­BF**_**4**_ complex, an electron populates the d_*x* ^2^–y^2^_ antibonding (σ*) molecular orbital, which contributes to the observed bond lengthening. Simultaneously, there is a depopulation of the d_*z* ^2^_ molecular orbital, as evidenced by the contraction of the axial Ni–N bond from 2.431 Å to 2.233 Å.

**Figure 6:**
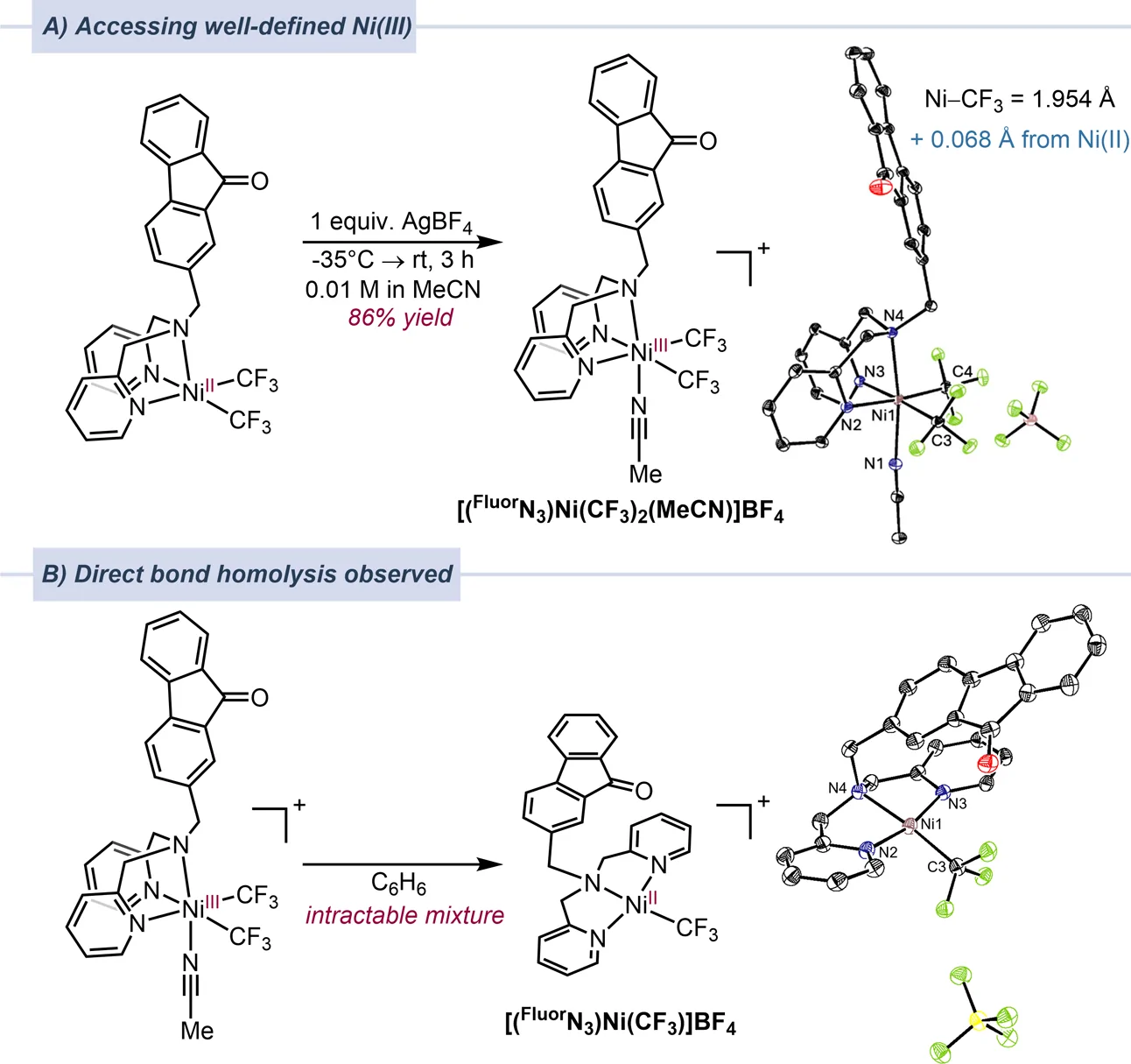
(A) Synthesis of **[(** ^**Fluor**^ **N**_**3**_ **)­Ni­(CF**_**3**_ **)**_**2**_ **(MeCN)]­BF**_**4**_ via single-electron oxidation; crystal structure showcasing lengthened Ni–CF_3_ bonds. (B) Direct bond homolysis observed leading to the generation of **[(** ^**Fluor**^ **N**_**3**_ **)­Ni­(CF**_**3**_ **)]­BF**_**4**_.

Electron paramagnetic resonance spectroscopy showed signals consistent with an *S* = 1/2 Ni­(III) species (Figure S52). The obtained spectrum is in good agreement with that of a structurally related octahedral d^7^ Ni­(III) complex reported by Sanford and co-workers.^43^ Spin density analysis revealed 90% of the unpaired spin density is localized on the metal center (see Supporting Information for computational details).

Exposure of **[(** ^**Fluor**^ **N**_**3**_ **)­Ni­(CF**_**3**_ **)**_**2**_ **(MeCN)]­BF**_**4**_ to benzene resulted in an immediate color change from deep maroon to yellow-orange, accompanied by the precipitation of a black solid, suggesting potential Ni(0) formation (Figure 6B). Analysis of the mixture by ^1^H and ^19^F NMR revealed an intractable mixture containing trifluorotoluene, supporting the generation of trifluoromethyl radical. Presumably, the high concentration of Ni­(III) in the reaction led to uncontrolled side reactions and disproportionation events, producing the intractable mixture. Single crystals suitable for XRD analysis were obtained from the mixture and identified as the cationic Ni­(II) species, **[(** ^**Fluor**^ **N**_**3**_ **)­Ni­(CF**_**3**_ **)]­BF**_**4**_, providing insight into the reactivity of bpma bis­(trifluoromethyl)­Ni­(III) complexes (Figure 6B).

### Probing Reactivity from d–d Manifolds

Examination of the electronic transitions disclosed by TD-DFT calculations revealed weak ^1^d–d transitions for the **(** ^**Fluor**^ **N**_**3**_ **)­Ni­(CF**_**3**_ **)**_**2**_ and **(** ^**Bn**^ **N**_**3**_ **)­Ni­(CF**_**3**_ **)**_**2**_ complexes at 587 and 592 nm, respectively. Visualization of the NTOs for these excited states revealed donor orbitals with Ni d_*z* ^2^_ character, and acceptor orbitals with Ni d_*x* ^2^–*y* ^2^_ character, populating antibonding orbitals along the Ni–CF_3_ and equatorial Ni–N axes (Figure 7, see Supporting Information for visualization of **(** ^**Bn**^ **N**_**3**_ **)­Ni­(CF**_**3**_ **)**_**2**_ NTOs).^26,53^ As a result, we considered whether direct excitation of this transition could lead to bond homolysis.^96^ To probe the potential for reactivity from a d–d excited state, the complexes were dissolved in benzene and irradiated with red light for 72 h, due to the low oscillator strengths of the d–d transitions.^101^ Analysis of the **(** ^**Fluor**^ **N**_**3**_ **)­Ni­(CF**_**3**_ **)**_**2**_ and **(** ^**Bn**^ **N**_**3**_ **)­Ni­(CF**_**3**_ **)**_**2**_ reaction mixtures revealed the generation of trifluorotoluene, albeit in lower yields (31% and 24%, respectively), suggesting that population of a d–d state can promote bond homolysis of the Ni–CF_3_ bonds (Figures 7 and S54 and S55). Similar yields across the complexes further demonstrate that the population of Ni–CF_3_ antibonding orbitals leads to bond lengthening and weakening, providing a platform for bond homolysis. Examination of the triplet excited states using TD-DFT revealed an analogous d–d transition as the lowest energy excited state for both complexes (see Supporting Information). In the optimized ground state geometry of **(** ^**Fluor**^ **N**_**3**_ **)­Ni­(CF**_**3**_ **)**_**2**_, the average Ni–CF_3_ bond length is 1.919 Å. In the computed triplet state, the Ni–CF_3_ bonds lengthen by 0.138 Å to 2.057 Å, supporting the population of antibonding orbitals (Figure S41). While the equatorial Ni–N bonds likewise lengthen in the computed triplet state geometry by an average of 0.192 Å, the L_3_ nature of the ligand provides additional stabilization due to the chelate effect, and no ligand dissociation is observed. Further analysis of the computed triplet state metrical parameters revealed a contraction of the axial Ni–N bond by 0.351 Å, from 2.493 Å in the ground state to 2.142 Å in the triplet state. Presumably, this contraction is attributed to the depopulation of axial Ni–N antibonding molecular orbitals in the d–d state, showcased in the NTO depiction (Figure 7).

**Figure 7:**
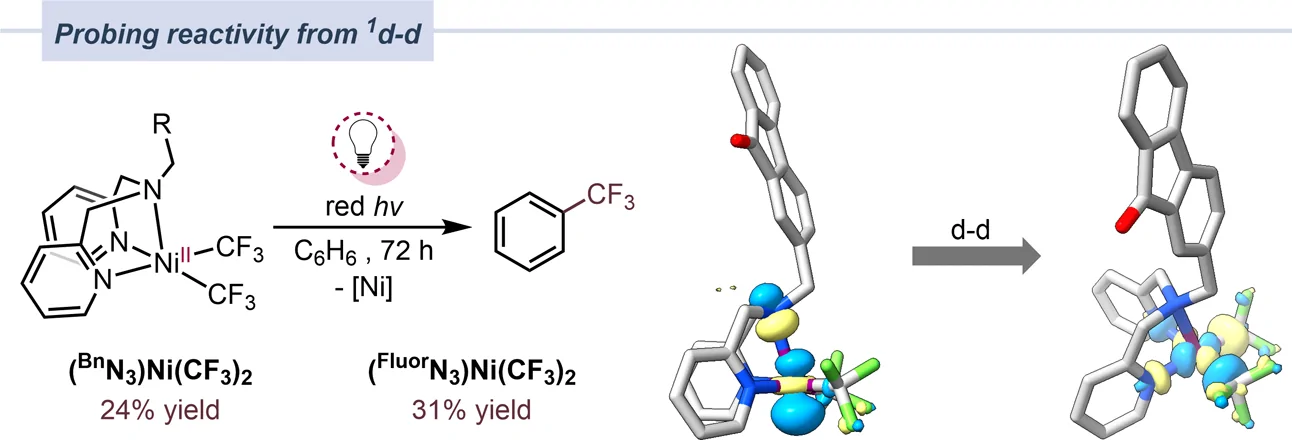
Reactivity of bis­(trifluoromethyl)­nickel­(II) complexes under red light irradiation for 72 h; visualization of NTOs of ^1^d–d transition at 587 nm for **(** ^**Fluor**^ **N**_**3**_ **)­Ni­(CF**_**3**_ **)**_**2**_.

Relaxation to a ^3^d–d state has been previously postulated to account for metal–ligand bond homolysis in photoactive transition metal complexes, including in bipyridine and related nitrogen-based nickel complexes.^25,26,53,93,100^ For bipyridine-ligated Ni­(*o-tol*)­(Cl) complexes, relaxation to a final ^3^d–d state has been proposed and supported by transient absorption and transient IR spectroscopies, and K-edge and L-edge spectroscopies.^53,93^ For the same system, temperature-dependent photolysis experiments support bond homolysis occurring from a repulsive ^3^LMCT state.^102^ While the involvement of ^3^LMCT energy surfaces cannot be excluded in our system, our results demonstrate that direct excitation of MLCT and d–d manifolds leads to bond homolysis.^25,103,104^

### Comparison of Visible Light Irradiation versus Chemical Oxidation

Finally, to assess the advantages of tethering a photoresponsive unit for Ni–CF_3_ bond cleavage, the reactivity under visible light irradiation was compared to chemical oxidation. Photochemical studies revealed the ability to activate two trifluoromethyl bonds when a solution of (^**Fluor**^ **N**_**3**_ **)­Ni­(CF**_**3**_ **)**_**2**_ is irradiated with visible light (*vide supra*). Lengthening of the Ni–CF_3_ bond was observed in the solid-state structure of the bis­(trifluoromethyl)­nickel­(II) complex upon chemical oxidation, affording a six-coordinate bis­(trifluoromethyl)­nickel­(III) complex with acetonitrile coordinated. Given the observed bond lengthening and proposed MLCT state leading to formal oxidation of the metal center in the excited state, **(** ^**Bn**^ **N**_**3**_ **)­Ni­(CF**_**3**_ **)**_**2**_ was treated with a chemical oxidant and the reactivity was assessed. Treatment with FcPF_6_ led to an 8% yield of trifluorotoluene after 18 h at room temperature (Figures 8 and S56). Next, the addition of stoichiometric oxidant combined with visible light irradiation was evaluated. Treatment of **(** ^**Bn**^ **N**_**3**_ **)­Ni­(CF**_**3**_ **)**_**2**_ with one equivalent of FcPF_6_ in benzene, followed by visible light irradiation at 405 nm for 12 h, resulted in a 14% yield of trifluorotoluene (Figures 8 and S56). Overall, these results highlight that the introduction of a photoresponsive diaryl ketone motif into the secondary coordination sphere of a bis­(trifluoromethyl)­nickel­(II) complex presents significant advantages over the addition of external oxidants, as even in the presence of light irradiation, the generation of CF_3_ radical from chemical oxidation of **(** ^**Bn**^ **N**_**3**_ **)­Ni­(CF**_**3**_ **)**_**2**_ does not compare to the yields obtained from irradiation of **(** ^**Fluor**^ **N**_**3**_ **)­Ni­(CF**_**3**_ **)**_**2**_ (Figure S56).

**Figure 8:**
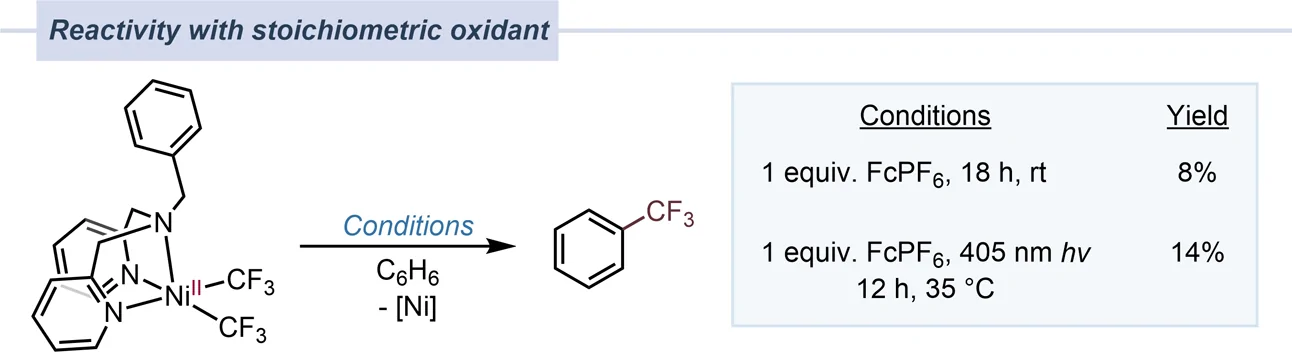
Reactivity of **(** ^**Bn**^ **N**_**3**_ **)­Ni­(CF**_**3**_ **)**_**2**_ upon one-electron oxidation with chemical oxidant.

## Conclusions

In summary, engineering latent ligand-centered radicals into the SCS of a Ni­(II) complex enabled previously inaccessible reactivity. Using the activation of persistent Ni–CF_3_ bonds as a readout of this concept, the study presented herein demonstrates a new design principle for metal center modulation. Mechanistic insights from CV, TD-DFT, and transient absorption spectroscopy support the initial population of an MLCT state that decays on the picosecond time scale to a long-lived ^3^d–d state. A significant perturbation of the electronic structure was observed as new electronic transitions were uncovered when examining the photophysical properties of (^**Fluor**^ **N**_**3**_ **)­Ni­(CF**_**3**_ **)**_**2**_. The control complex bearing no photoresponsive unit, **(** ^**Bn**^ **N**_**3**_ **)­Ni­(CF**_**3**_ **)**_**2**_, exhibited minimal reactivity under thermal heating, chemical oxidation, and visible light irradiation with and without exogenous fluorenone, underscoring the utility of latent ligand-centered radicals.

These studies demonstrate that introducing a stimuli-responsive fluorenone unit into the SCS can unlock reactivity at the metal center. More broadly, this work demonstrates the potential of latent ligand-centered radicals to expand the scope of secondary coordination sphere modifications and to broaden the reactivity of transition metal complexes. Further efforts toward exploring latent ligand-centered radical strategies for generating open-shell species in proximity to metal centers are currently underway in our laboratory.

## Supplementary Material



## Notes

Experimental procedures, NMR spectra, and crystallographic data (PDF)

## †

Division of Chemistry and Chemical Engineering, California Institute of Technology, Pasadena, California 91125, United States
