# Supplementary material for: Unlocking Distinct Excited States of a Nickel(II) Complex Without Modulating the Primary Coordination Sphere
Source: JACS Au. 2026 Aug 13;6(9):5068–80. doi: 10.1021/jacsau.6c00738 (PMC13625514; doi:10.1021/jacsau.6c00738)
Supplement: Supplementary file 1 [file au6c00738_si_001.pdf]

*Supporting Information*

# Unlocking Distinct Excited States of a Nickel(II) Complex without Modulating the Primary Coordination Sphere

*Hailey Hendricks, Raina M. Morley, Angel Yahir Gomez, Yokari Godínez-Loyola, Matthew V. Pecoraro, Gregory D. Scholes, Jose B. Roque**

*Department of Chemistry, Princeton University, Princeton, New Jersey 08644, United States*

*jroque@princeton.edu*

## Table of Contents

| | |
|---|---|
| I. General Considerations | S2 |
| II. Synthesis of Ligand $^{\mathbf{Fluor}}\mathbf{N_3}$ | S4 |
| III. Synthesis of Ni(II) Bis(trifluoromethyl) Complexes | S7 |
| IV. Cyclic Voltammetry | S9 |
| V. Experimental UV-Visible Absorption Spectra | S12 |
| VI. Preparation of Reaction Mixtures | S16 |
| VII. Reactivity Under Thermal Heating | S17 |
| VIII. Reactivity Under Visible Light Irradiation (405 nm, 365 nm) | S25 |
| IX. Stern-Volmer Quenching Studies | S32 |
| X. Transient Absorption Spectroscopy | S33 |
| XI. Computational Calculations | S41 |
| XII. Reactivity Under Visible Light Irradiation (450 nm) | S59 |
| XIII. Spectroelectrochemistry | S61 |
| XIV. Synthesis and Reactivity of Ni(III) Bis(trifluoromethyl) Complex | S64 |
| XV. EPR Experiments | S67 |
| XVI. Reactivity Under Red Light Irradiation | S68 |
| XVII. Evaluation of Chemical Oxidation | S71 |
| XVIII. Attempts at Characterization Post-Irradiation | S73 |
| XIX. NMR Spectra | S74 |
| XX. Geometry Scan Along Ni–O Distance | S78 |
| XXI. Hirshfeld Spin Density Analysis | S79 |
| XXII. Crystallographic Data | S82 |
| XXIII. References | S122 |

## I. General Considerations.

All air and moisture sensitive manipulations were carried out in an MBraun inert atmosphere (nitrogen) dry glovebox unless otherwise noted. All glassware was stored in a preheated oven prior to use. The solvents used for air and moisture sensitive manipulations were dried and degassed using literature procedures.[1] Deuterated solvents for NMR spectroscopy analysis of air sensitive nickel complexes (benzene-d6) were distilled from sodium metal under high vacuum and stored in the glovebox over sodium metal. All liquid reagents used in the synthesis of metal precursors were dried by stirring over $CaH_2$ under static vacuum at 23°C for 24 hours and then distilled under high vacuum and stored in the glovebox. Solid substrates and synthesized ligands were dried under high vacuum for 24 hours at 23°C and then stored in the glovebox. $\mathbf{^{Bn}N_3}$ and $\mathbf{(MeCN)_2Ni(CF_3)_2}$ were prepared according to literature procedures.[2,3] Prior to bringing into the glovebox, 2,2,6,6-tetramethyl-1-piperidinyloxy (TEMPO) was purified by sublimation under an $N_2$ atmosphere and stored in the glovebox freezer.

$^1H$ NMR spectra were recorded on either Bruker ADVANCE 400 or 500 spectrophotometers operating at 399.8 MHz, and 500.46 MHz, respectively. $^{13}C$ NMR spectra were recorded on Bruker ADVANCE 500 spectrometer operating AT 125.85 MHz, respectively. $^{19}F$ NMR spectra were recorded on Bruker ADVANCE 500 spectrometer operating at 471 MHz and were referenced to CFCl3 as an external standard. All $^1H$ and $^{13}C$ NMR chemical shifts are reported in ppm relative to SiMe4 using the $^1H$ (chloroform-d: 7.26 ppm; benzene-d6: 7.16 ppm) and $^{13}C$ (chloroform-d: 77.16 ppm; benzene-d6: 128.06 ppm) chemical shifts of the solvent as a standard. $^1H$ NMR data for diamagnetic compounds are reported as follows: chemical shift, multiplicity (s = singlet, d = doublet, t = triplet, q = quartet, p = pentet, br = broad, m = multiplet), coupling constants (Hz), integration, assignment. $^{13}C$ NMR data for diamagnetic compounds are reported as follows: chemical shift. Elemental analyses were performed at Robertson Microlit Laboratories, Inc., in Ledgewood, NJ. Due to the air-sensitive nature of the complexes, elemental analysis results may deviate from theoretical values due to decomposition during transport.

Single crystals suitable for X-ray diffraction were coated with polyisobutylene oil in the dry box, transferred to a nylon loop and then quickly transferred to either the goniometer head of a diffractometer equipped with a Bruker PHOTON III detector and Cu X-Ray tube ($\lambda$ = 1.54178 Å) or a Rigaku XtaLAB Synergy-i equipped with a Mo X-ray tube ($\lambda$ = 0.71073 Å) and a Cu X-ray tube ($\lambda$ = 1.54178 Å). Preliminary data revealed the crystal system. The data collection strategy was optimized for completeness and redundancy using either the Bruker APEXII software suite or Rigaku CrysAlis$^{Pro}$ software suite. The space group was identified, and the data were processed

and corrected for absorption. The structures were solved using intrinsic phasing (SHELXT) and completed by subsequent Fourier synthesis and refined by full-matrix least-squares procedures in Olex2. Unless otherwise specified, hydrogen atoms were modelled as riding atoms.

## II. Synthesis of Ligand $^{Fluor}N_3$.

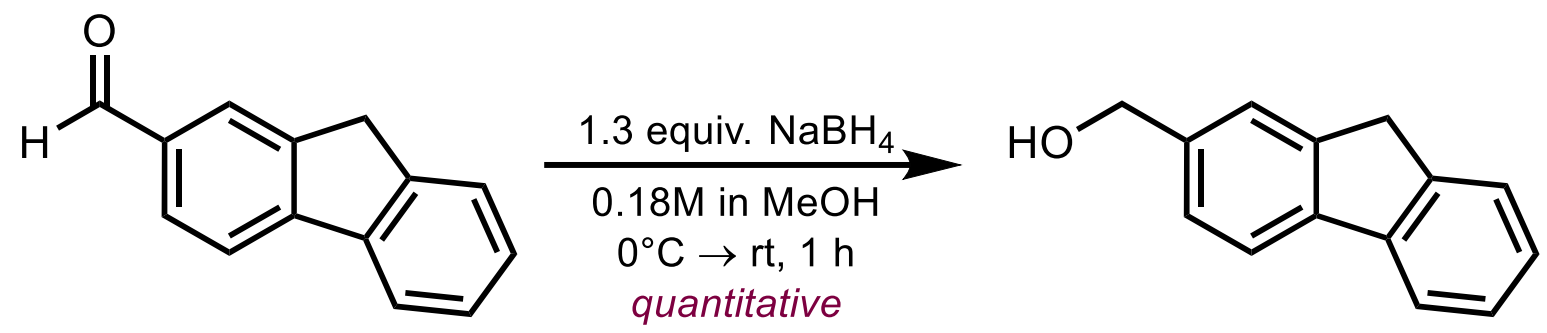


**(9H-fluoren-2-yl)methanol (S1)**. The title compound was synthesized according to the literature procedure.[4] To a suspension of fluorene-2-carboxaldehyde (4.00 g, 20.39 mmol, 1.0 equiv.) in methanol (250 mL) in a 500 mL round bottom flask was added $NaBH_4$ (1.01 g, 26.77 mmol, 1.3 equiv.) portion wise at 0 °C. The resulting mixture was warmed to room temperature and vigorously stirred for 1 h. The reaction was quenched by the addition of saturated aqueous $NH_4Cl$ solution (100 mL) and extracted with EtOAc (3 x 150 mL). The combined organic layers were washed with brine, dried over anhydrous $Na_2SO_4$, filtered, and concentrated under reduced pressure to yield clean product **S1** as a white solid in quantitative yield. $^1H$ NMR data in accordance with reported literature.[4]

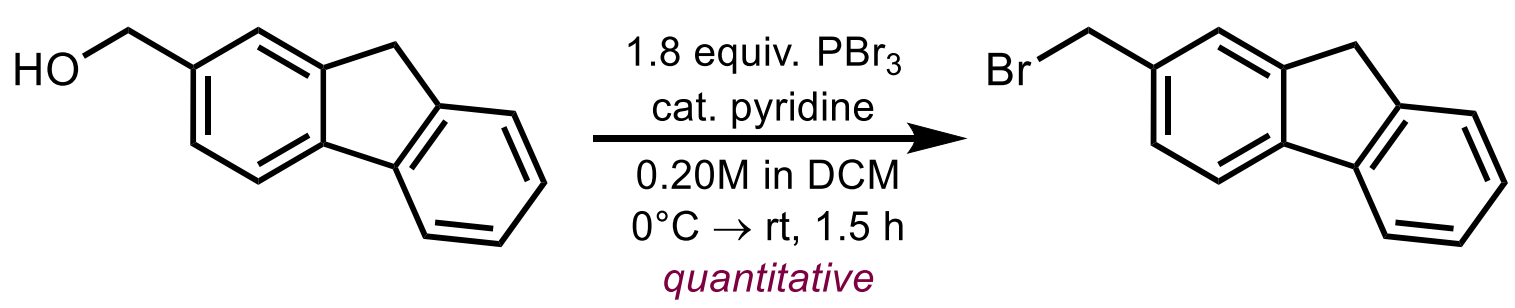


**2-(bromomethyl)-9H-fluorene (S2)**. The title compound was synthesized according to the literature procedure.[4] To a suspension of **S1** (4.116 g, 20.97 mmol, 1.0 equiv.) in DCM (250 mL) was added $PBr_3$ (2.97 mL, 31.46 mmol, 1.8 equiv.) dropwise and pyridine (10 drops) at 0 °C under $N_2$. The resulting solution was warmed to room temperature and stirred for an additional 1.5 h. The reaction was quenched by the addition of water (250 mL) and extracted with DCM (3 x 150 mL). The combined organic layers were washed with brine, dried over anhydrous $Na_2SO_4$, filtered, and concentrated under reduced pressure to yield clean product **S2** as a white solid in quantitative yield. $^1H$ NMR data in accordance with reported literature.[4]

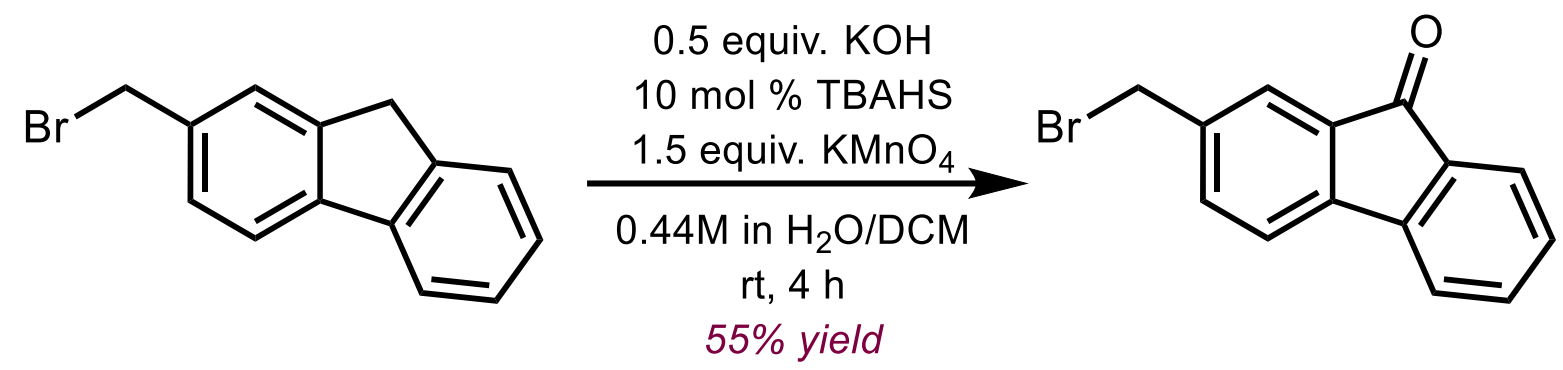


**2-(bromomethyl)-9H-fluoren-9-one (S3)**. The title compound was synthesized according to the adapted literature procedure.[5] A 250 mL round bottom flask was charged with $KMnO_4$ (4.82 g, 30.47 mmol, 1.5 equiv.), KOH (0.57 g, 10.16 mmol, 0.5 equiv.), tetrabutylammonium hydrogen sulfate (0.69 g, 2.03 mmol, 10 mol %), and water (71 mL). To the stirring solution was added **S2** (5.26 g, 20.31 mmol, 1.0 equiv.) as a solution in DCM (71 mL). The resulting solution was vigorously stirred until the purple color was discharged or TLC indicated full consumption of starting material (approximately 4 hours). The mixture was acidified with HOAc (15 mL) and sodium sulfite was added portion wise until the brown color was discharged, yielding a lime green solution. The reaction was extracted with DCM (3 x 50 mL) and the combined organic phases were washed with brine, dried over anhydrous $Na_2SO_4$, and concentrated under reduced pressure. The crude material was purified by column chromatography (DCM/Hexanes, 3:1) to yield clean product **S3** as a yellow powder in a 55% yield. $^1$H NMR data in accordance with reported literature.[5]

**Alternative synthesis of S3**.

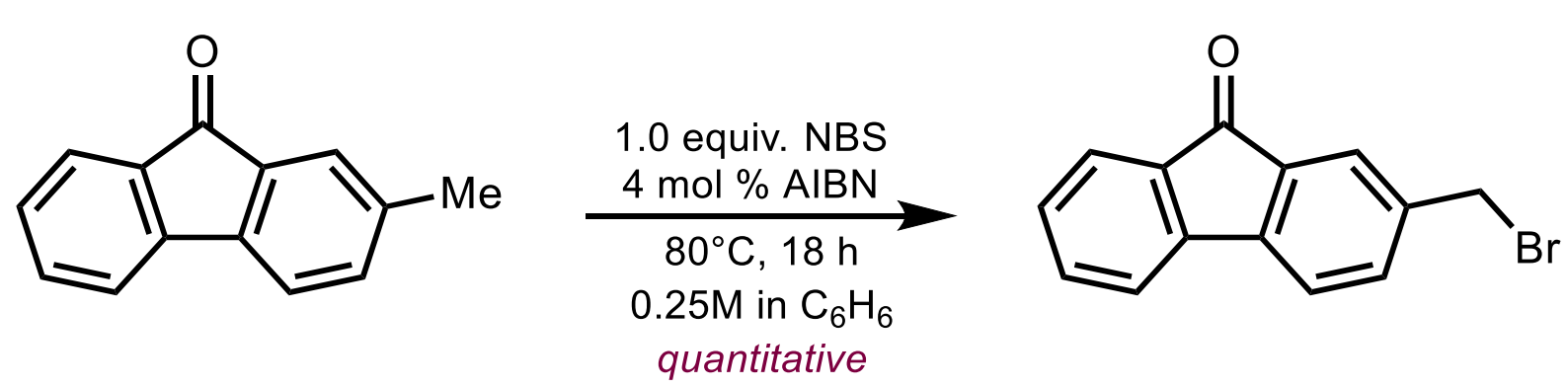


**2-(bromomethyl)-9H-fluoren-9-one (S3)**. A 500 mL round bottom flask was charged with 2-methyl-9H-fluoren-9-one (6.441 g, 33.16 mmol, 1.0 equiv.), N-bromosuccinimide (5.902 g, 33.16 mmol, 1.0 equiv.), azobisisobutyronitrile (200 mg, 1.33 mmol, 4 mol %), and benzene (250 mL). The resulting solution was brought to reflux and allowed to further stir at reflux for 18 h. Upon cooling to room temperature water (200 mL) was added and the mixture was extracted with EtOAc (3 x 100 mL). The combined organic layers were washed with brine (100 mL), dried over anhydrous $Na_2SO_4$, and concentrated under reduced pressure. The crude material was purified by silica plug chromatography (EtOAc/Hexanes, 1:1) to yield clean product **S3** as a yellow powder in quantitative yield. $^1$H NMR data in accordance with reported literature.[5]

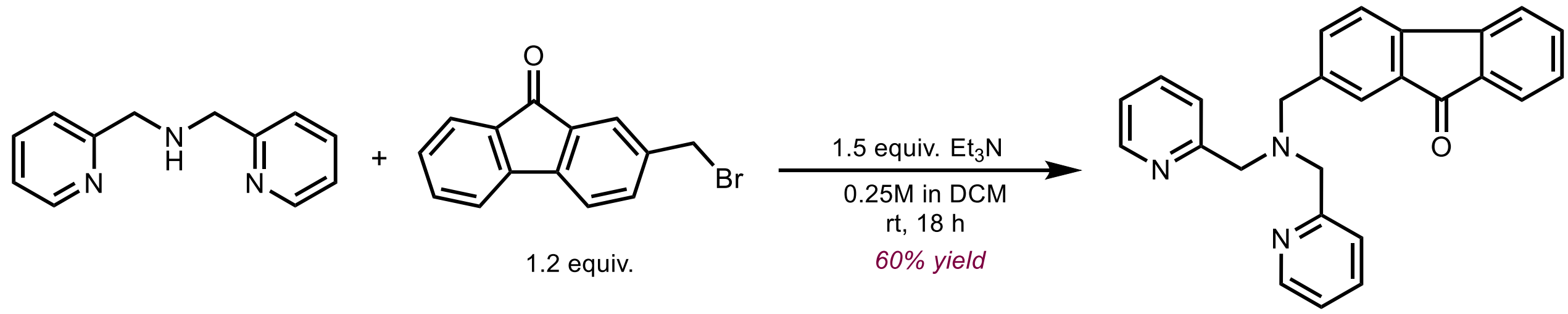


**2-((bis(pyridin-2-ylmethyl)amino)methyl)-9H-fluoren-9-one ($^{Fluor}N_3$).** The title compound was synthesized by an adapted literature procedure.[2] To a round bottom flask was added di-(2-picolyl)amine (1.81 g, 9.09 mmol, 1 equiv.) dissolved in dichloromethane (0.25M). $Et_3N$ (1.90 mL, 13.64 mmol, 1.5 equiv.) was added, and the solution was allowed to stir for 5 minutes. **S3** (2.98 g, 10.90 mmol, 1.2 equiv.) was added portion wise, and the reaction was allowed to stir at room temperature (RT) for 18 hours. The reaction was quenched upon the addition of 0.1M NaOH (aq.) solution. The aqueous layer was separated and extracted 3x with DCM, maintaining a basic pH throughout the workup with the addition of 0.1M NaOH (aq.) solution as needed. The combined organic layers were brine washed, dried with $Na_2SO_4$, and concentrated to yield the crude residue as an orange-brown oil. The residue was purified via column chromatography (MeOH/DCM, 1:99) to yield clean product **$^{Fluor}N_3$** as a yellow-orange solid in 60% yield.

**$^1$H NMR** (500 MHz, $CDCl_3$) δ 8.51 (m, 2H), 7.71 (d, *J* = 1.5 Hz, 1H), 7.67 (td, *J* = 7.7, 7.6, 1.8 Hz, 2H), 7.62 (dt, *J* = 7.2, 1.0, 1.0 Hz, 1H), 7.56 (dt, *J* = 7.9, 1.1, 1.1 Hz, 2H), 7.49 (m, 1H), 7.44 (m, 3H), 7.25 (m, 1H), 7.14 (ddd, *J* = 7.5, 4.8, 1.2 Hz, 2H), 3.81 (s, 4H), 3.68 (s, 2H).

**$^{13}$C NMR** (126 MHz, $CDCl_3$) δ 194.1, 159.4, 149.1, 144.5, 143.5, 140.8, 136.7, 135.4, 134.8, 134.6, 134.5, 129.0, 124.8, 124.4, 123.1, 122.2, 120.3, 120.3, 60.2, 58.3.

**HRMS** (ESI): calc for $C_{26}H_{21}N_3O$ [M+H]+ 392.1757, found 392.1765

**ATR-IR** ($cm^{-1}$): C=O stretch: 1710.6

## III. Synthesis of Ni(II) Bis(trifluoromethyl) Complexes.

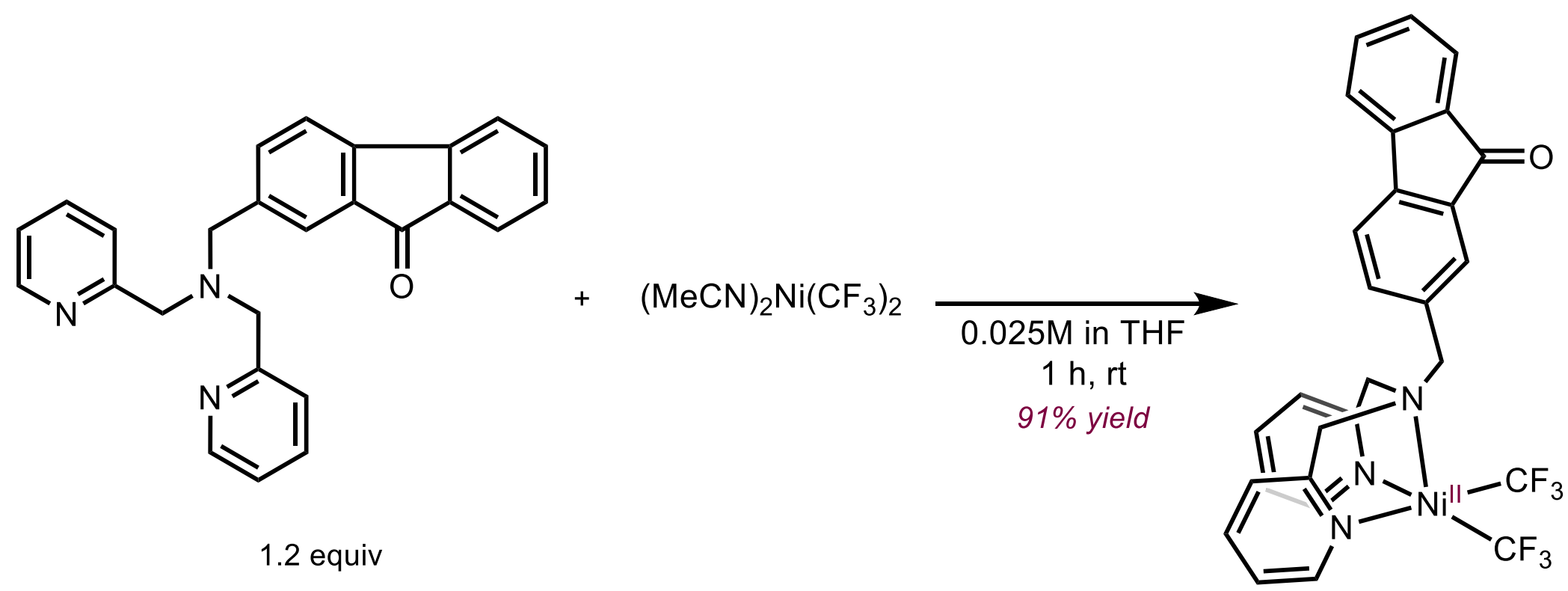


**(2-((bis(pyridin-2-ylmethyl)amino)methyl)-9H-fluoren-9-one)bis(trifluoromethyl)nickel**

**($^{Fluor}N_3$)$Ni(CF_3)_2$** was synthesized from an adapted literature procedure.[3] In a nitrogen filled glovebox, **$(MeCN)_2Ni(CF_3)_2$** (0.100 g, 0.359 mmol, 1 equiv.) was added to a 20 mL scintillation vial and dissolved in 5 mL of THF with a stir bar. In a separate vial, **$^{Fluor}N_3$** (0.168 g, 0.430 mmol, 1.2 equiv.) was dissolved in 10 mL of THF. The ligand solution was added dropwise to the reaction mixture and allowed to stir at room temperature for 1 hour. Pentanes (5 mL) was added to the reaction mixture to precipitate a yellow-orange solid. The mixture was filtered through a pad of celite and the solid washed with pentanes to remove excess free ligand. The product **($^{Fluor}N_3$)$Ni(CF_3)_2$** was extracted with THF and concentrated under reduced pressure to yield the desired complex as a yellow-orange solid in a 91% yield.

**$^1$H NMR** (500 MHz, $CD_3CN$) δ 9.24 (d, J = 4.1 Hz, 2H), 7.94 (d, J = 1.6 Hz, 1H), 7.90 (dd, J = 7.6, 1.7 Hz, 1H), 7.81 (dd, J = 7.6, 4.1 Hz, 1H), 7.76 (d, J = 7.4 Hz, 1H), 7.67 (d, J = 7.4 Hz, 1H), 7.62 (m, 1H), 7.51 (td, J = 7.7, 7.7, 1.6 Hz, 2H), 7.40 (m, 1H), 7.14 (t, J = 5.9, 5.9 Hz, 2H), 7.00 (d, J = 7.8 Hz, 2H), 5.20 (s, 2H), 4.91 (d, J = 16.3 Hz, 2H), 3.89 (d, J = 16.3 Hz, 2H).

**$^{19}$F NMR** (471 MHz, $CD_3CN$) δ -25.74.

**$^{13}$C NMR** (126 MHz, $DMSO_{d6}$) δ 193.3, 158.3, 149.3, 143.8, 143.5, 137.6, 137.5, 137.4, 135.5, 133.8 (q, J = 366.8), 133.74, 133.5, 129.6, 125.6, 124.0, 123.2, 123.05, 121.5, 121.32, 63.4, 60.4, 25.1.

**HRMS-ESI:** calculated for $[C_{27}H_{21}F_3N_3NiO]^+$: 518.0985, found: 518.0994.

Note: The eluent for HRMS includes formic acid, causing heterolysis of the **($^{Fluor}N_3$)$Ni^{II}(CF_3)_2$** complex to the observed **[($^{Fluor}N_3$)$Ni^{II}CF_3$]$^+$** cation. This fragmentation pattern is consistent with structurally related bistrifluoromethyl nickel(II) complexes.[6]

Anal Calcd. for $C_{28}H_{21}F_6N_3NiO$: C, 57.18; H, 3.60, N, 7.14. Found: C, 56.39; H, 3.02, N, 6.67.

**ATR-IR** ($cm^{-1}$): C=O stretch: 1711.1

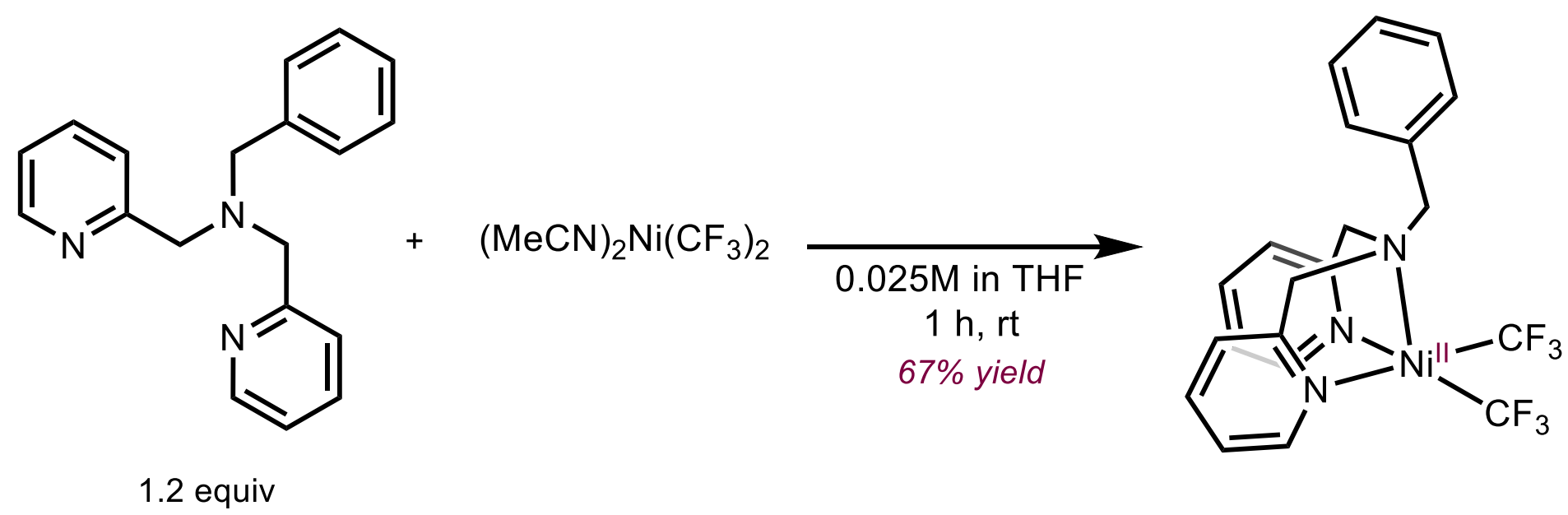


**(N-benzyl-1-(pyridin-2-yl)-N-(pyridin-2-ylmethyl)methanamine)bis(trifluoromethyl)nickel**

**($^{Bn}N_3$)Ni($CF_3$)$_2$** was synthesized from an adapted literature procedure.[3] In a nitrogen filled glovebox, **$(MeCN)_2Ni(CF_3)_2$** (0.100 g, 0.359 mmol 1 equiv.) was added to a 20 mL scintillation vial and dissolved in 5 mL of THF with a stir bar. In a separate vial, **$^{Bn}N_3$** (0.124 g, 0.430 mmol, 1.2 equiv.) was dissolved in 10 mL of THF. The ligand solution was added dropwise to the reaction mixture and allowed to stir at room temperature for 1 hour. Pentanes (5 mL) was added to the reaction mixture to precipitate a yellow-orange solid. The mixture was filtered through a pad of celite and the solid washed with pentanes to remove excess free ligand. The product **($^{Bn}N_3$)Ni($CF_3$)$_2$** was extracted with THF and concentrated under reduced pressure to yield the desired complex as an orange-brown solid in a 67% yield.

**$^1$H NMR** (500 MHz, $C_6D_6$) δ 8.97 (d, $J$ = 5.4 Hz, 2H), 7.24 (t, $J$ = 7.0 Hz, 3H), 7.13 (dd, $J$ = 7.2, 1.7 Hz, 2H), 6.33 (t, $J$ = 7.7, 7.7 Hz, 2H), 6.14 (t, $J$ = 6.5, 6.5 Hz, 2H), 5.81 (d, $J$ = 7.8 Hz, 2H), 5.38 (s, 2H), 4.58 (d, $J$ = 16.2 Hz, 2H), 3.14 (d, $J$ = 16.2 Hz, 2H).

**$^{19}$F NMR** (471 MHz, $C_6D_6$) δ -24.77.

**$^{13}$C NMR** (126 MHz, $DMSO_{d6}$) δ 158.4, 149.3, 137.5, 135.7, 133.8 (q, J = 369.7 MHz), 130.2, 128.6, 127.8, 123.2, 122.9, 63.9, 60.4.

**HRMS-ESI:** calculated for $[C_{20}H_{19}F_3N_3Ni]^+$: 416.0879, found: 416.0880.

Note: The eluent for HRMS includes formic acid, causing heterolysis of the **($^{Bn}N_3$)$Ni^{II}$($CF_3$)$_2$** complex to the observed **[($^{Bn}N_3$)$Ni^{II}CF_3$]$^+$** cation. This fragmentation pattern is consistent with structurally related bistrifluoromethyl nickel(II) complexes.[6]

Anal Calcd. for $C_{21}H_{19}F_6N_3Ni$: C, 51.89; H, 3.94, N, 8.64. Found: C, 50.61; H, 3.96, N, 8.22.

## IV. Cyclic Voltammetry.

Cyclic voltammetry on fluorenone, **$^{Fluor}N_3$, $(^{Bn}N_3)Ni(CF_3)_2$,** and **$(^{Fluor}N_3)Ni(CF_3)_2$** was performed in an MBraun inert atmosphere (nitrogen) dry glovebox with $O_2$ < 0.5 ppm and $H_2O$ < 0.5 ppm. The acetonitrile used was dried and degassed using literature procedures.[1] All solutions were prepared in the glovebox. $NBu_4PF_6$, $AgNO_3$, and ferrocene were recrystallized prior to use. The cyclic voltammetry was performed using a 3-electrode cell consisting of a $Ag/Ag^+$ reference electrode with a Ag wire in a solution of $AgNO_3$ (0.01 M) and $NBu_4PF_6$ (0.1 M) in acetonitrile, a 3 mm glassy carbon working electrode, and a Pt wire counter electrode. A 3 mL solution of each species (0.01 M unless otherwise noted) and $NBu_4PF_6$ (0.1 M) in acetonitrile was added to the cell. All CV's were run with a scan rate of 100 mV/s. All samples were referenced to ferrocene as an external standard scanning at 100 mV/s with a BASi eclipse potentiostat. The peak separation of the external standard couple [$Fc/Fc^+$] was found to be 82 mV, suggesting that the broad peak separation observed for the **$(^{Bn}N_3)Ni(CF_3)_2$** and **$(^{Fluor}N_3)Ni(CF_3)_2$** couples is inherent to the complexes in acetonitrile.[7]

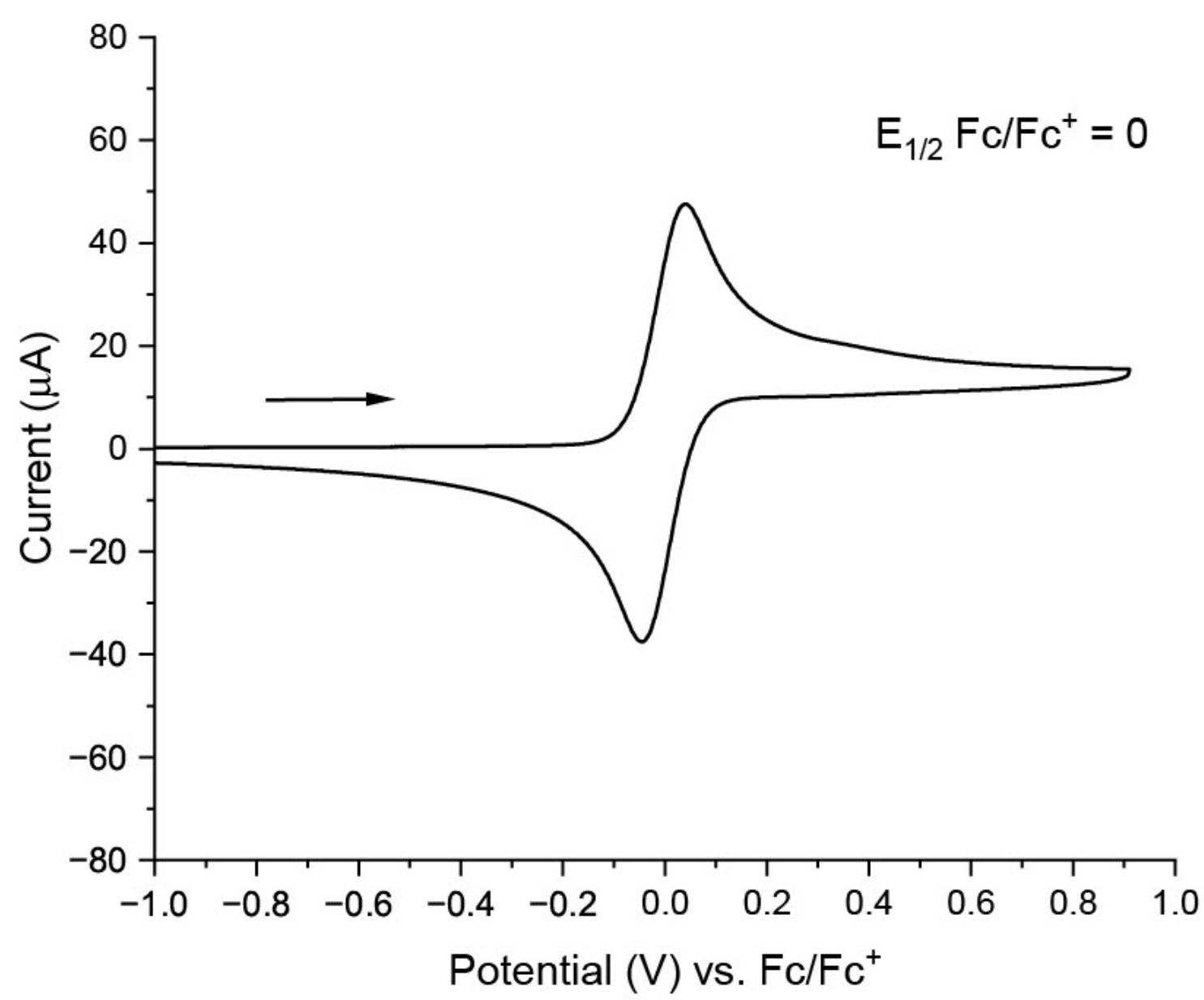


**Figure S1**: Cyclic voltammogram of ferrocene in acetonitrile. $\Delta E_p$ = 82 mV.

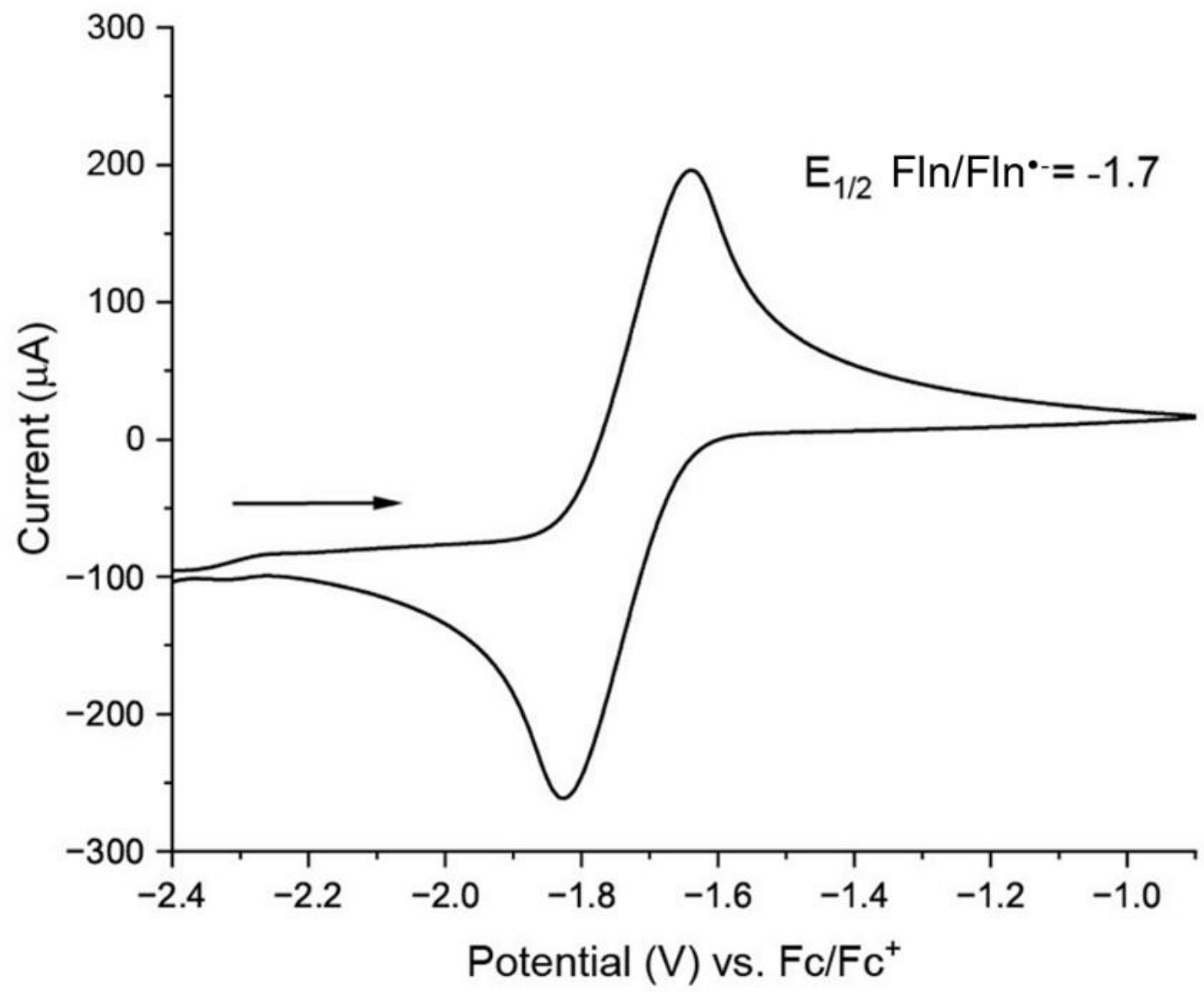


**Figure S2**: Cyclic voltammogram of fluorenone in acetonitrile. $\Delta E_p$ = 189 mV.

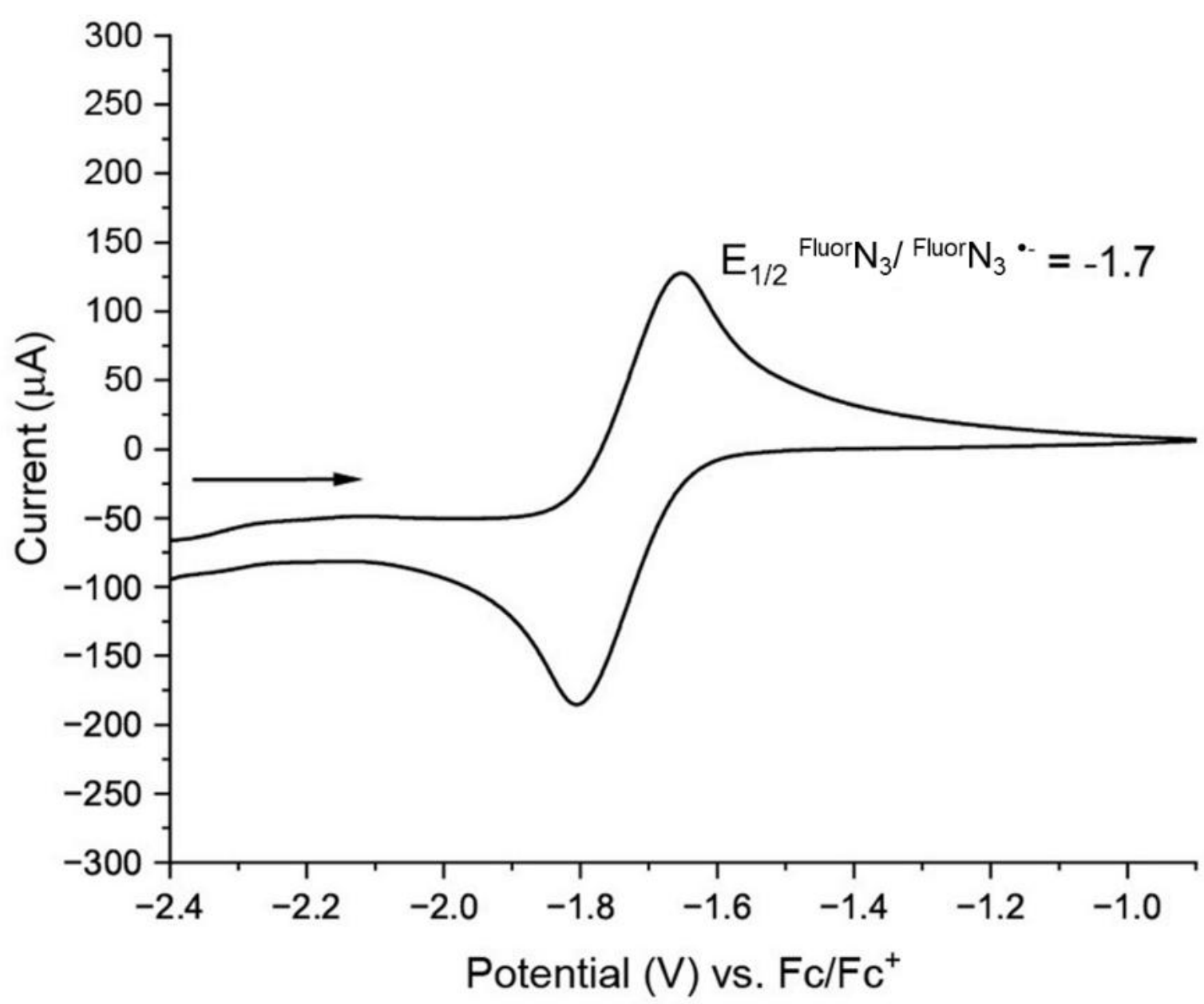


**Figure S3**: Cyclic voltammogram of $^{Fluor}N_3$ in acetonitrile. $\Delta E_p$ = 157 mV.

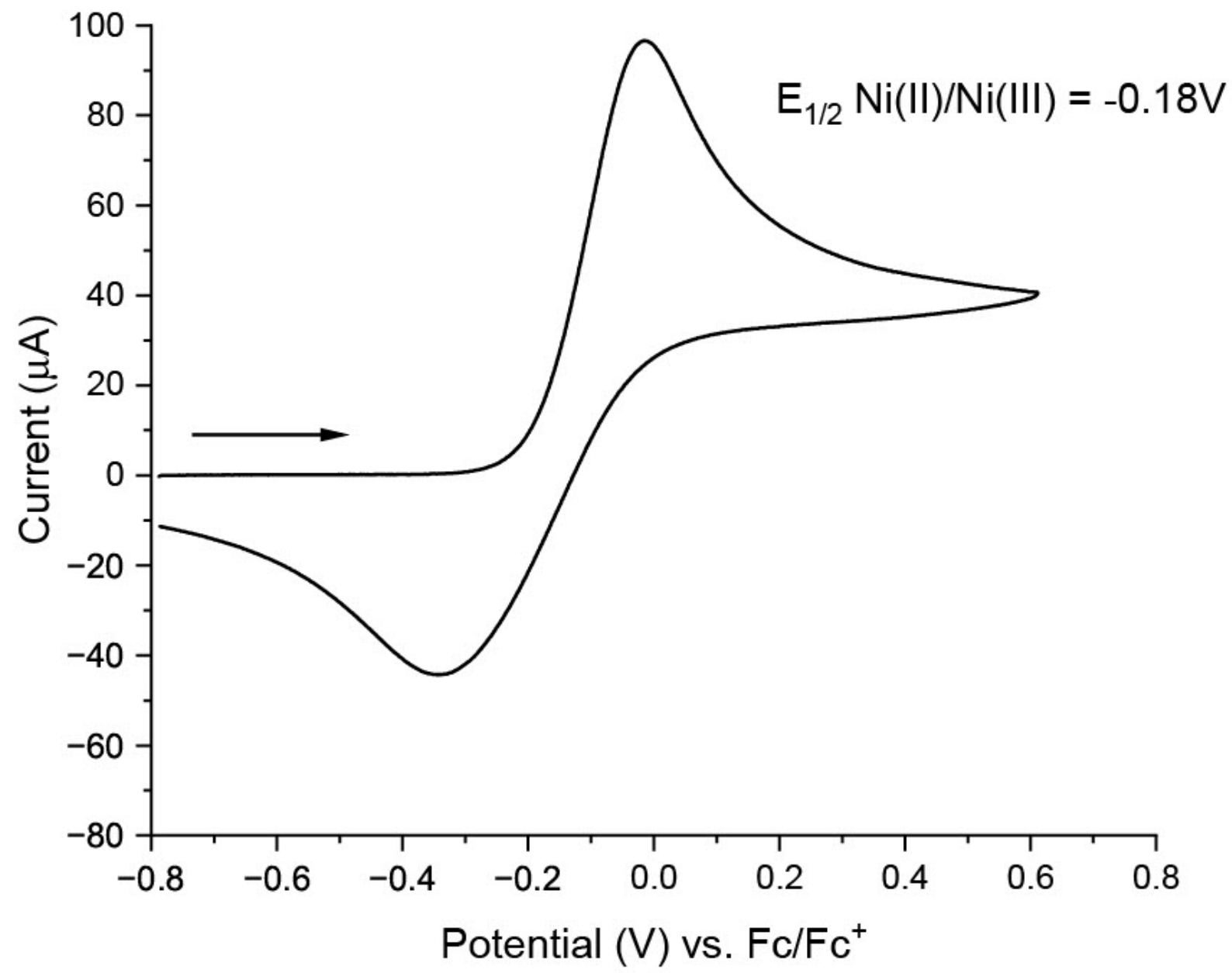


**Figure S4**: Cyclic voltammogram of $(^{Bn}N_3)Ni(CF_3)_2$ (0.075 M) in acetonitrile. $\Delta E_p$ = 326 mV.

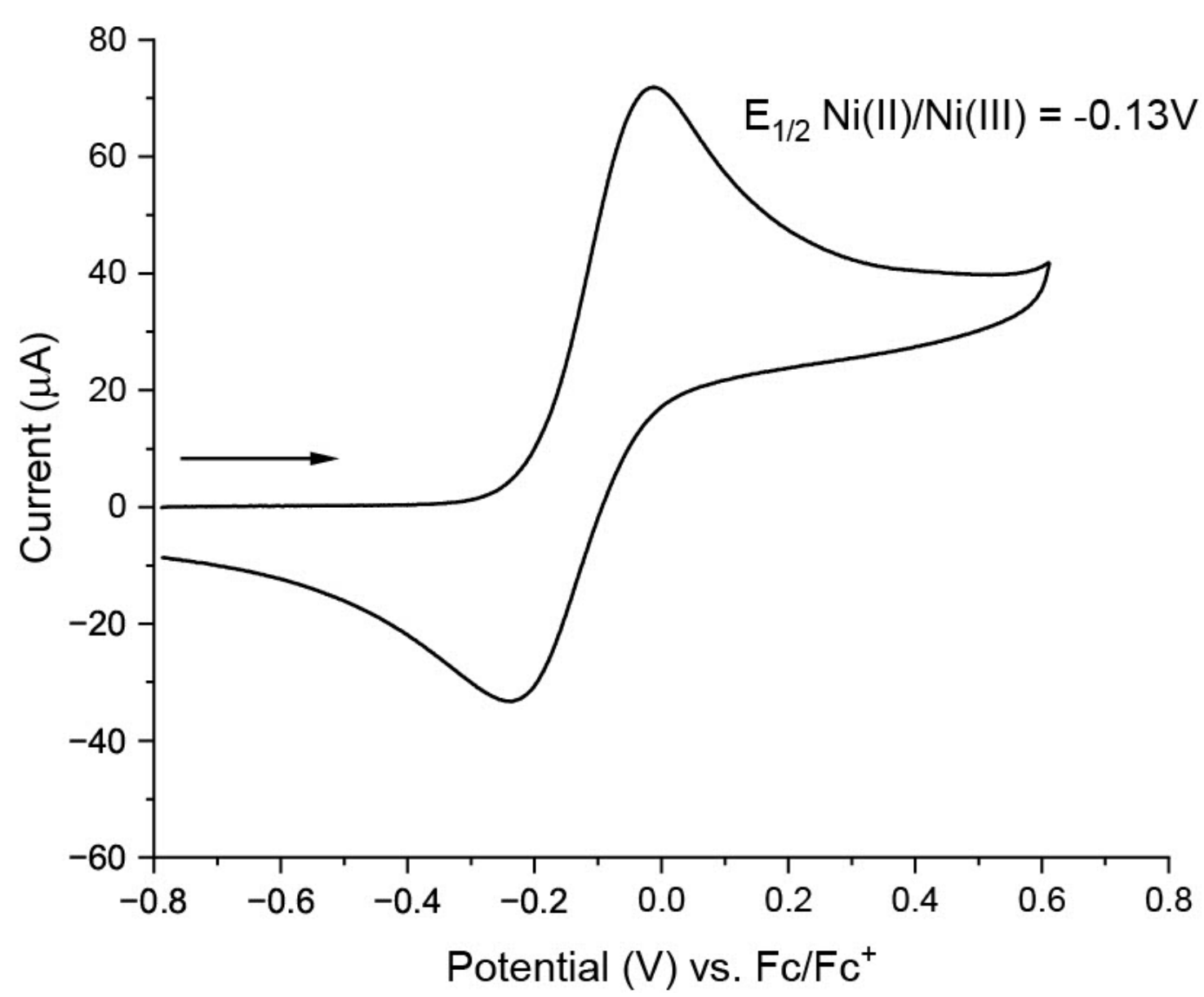


**Figure S5**: Cyclic voltammogram of $(^{Fluor}N_3)Ni(CF_3)_2$ (0.075 M) in acetonitrile. $\Delta E_p$ = 225 mV.

## V. Experimental UV-Visible Absorption Spectra.

All samples were prepared in an MBraun inert atmosphere (nitrogen) dry glovebox. The solvents used for sample preparation were dried and degassed using literature procedures.[1] Samples were prepared in a 1 cm quartz cuvette that was sealed for the duration of the UV-Visible experiments. UV-Vis spectra were acquired with an Agilent 8453 G1103A spectrophotometer. Blank samples were run in between each new species.

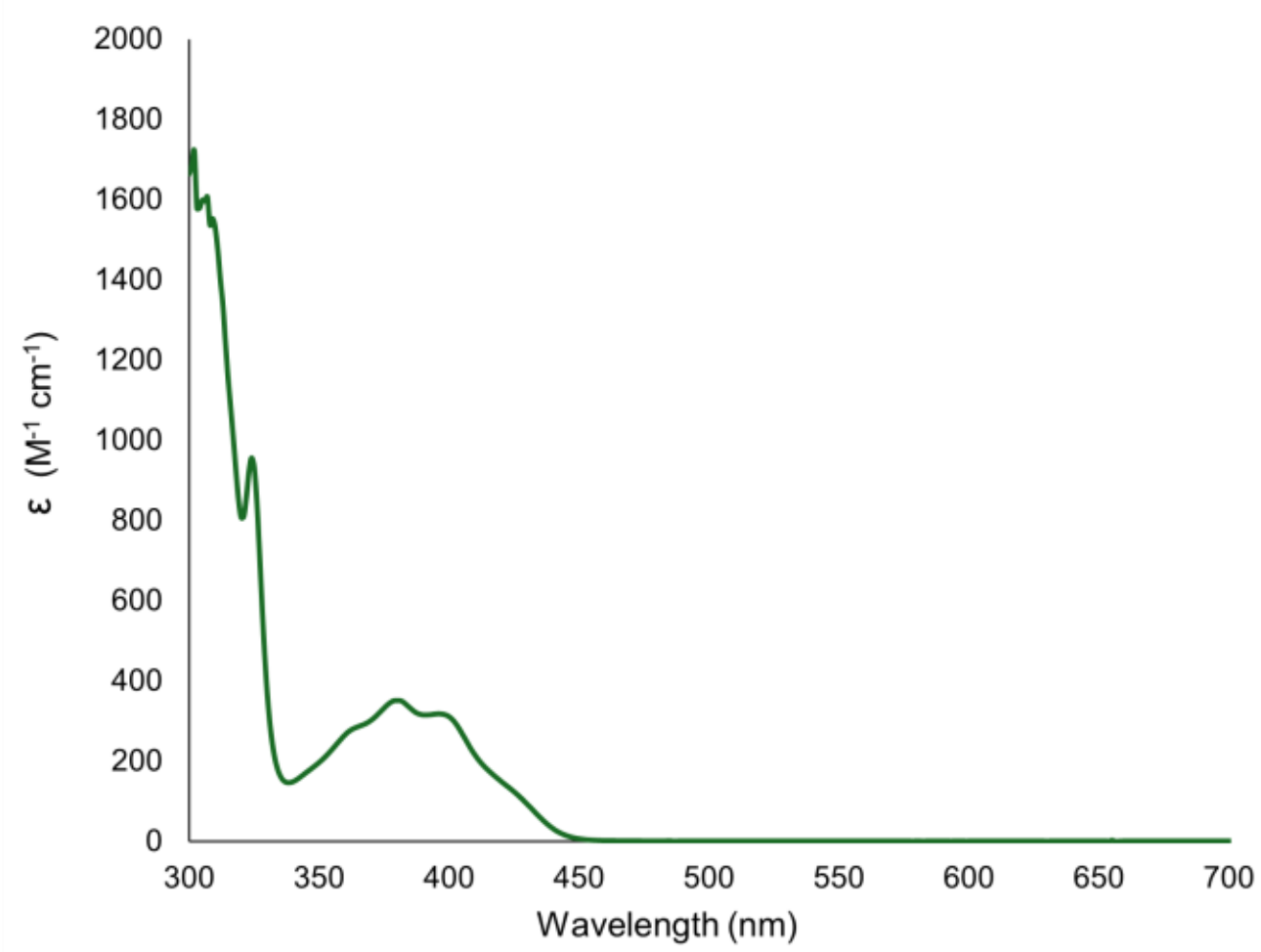


**Figure S6:** UV-Vis spectrum of fluorenone in benzene.

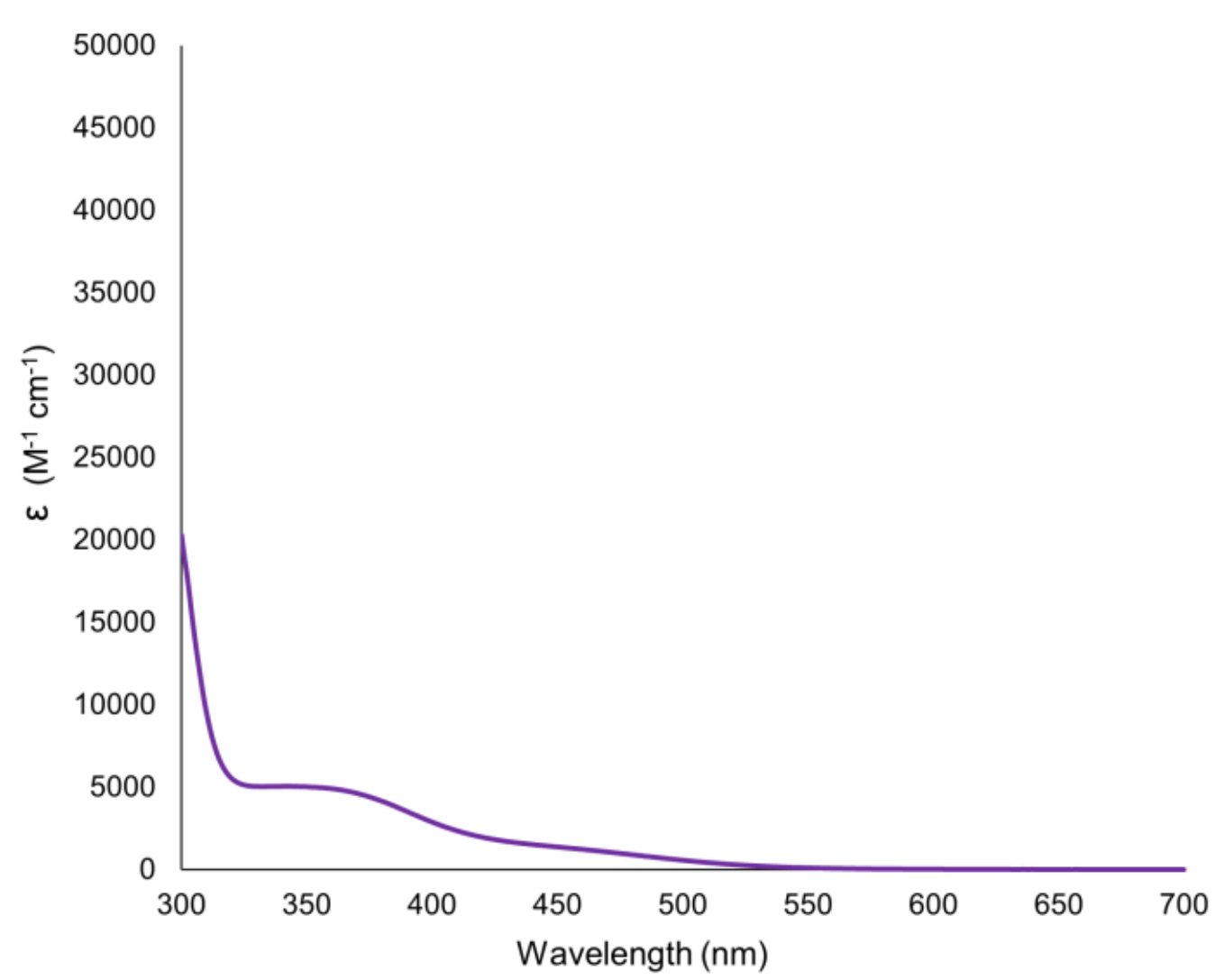


**Figure S7:** UV-Vis spectrum of **$(^{Bn}N_3)Ni(CF_3)_2$** in benzene.

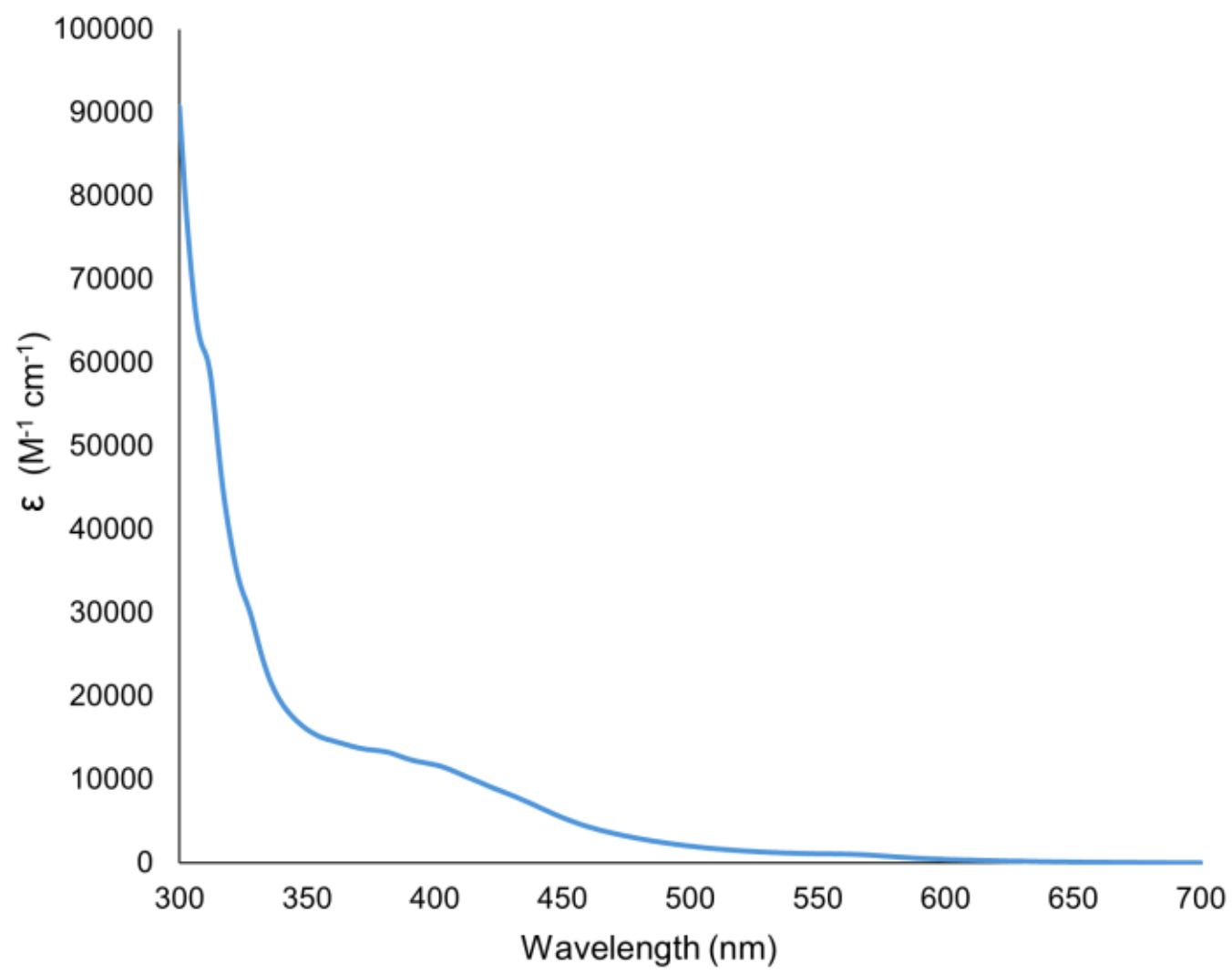


**Figure S8:** UV-Vis spectrum of **($^{Fluor}N_3$)Ni($CF_3$)$_2$** in benzene.

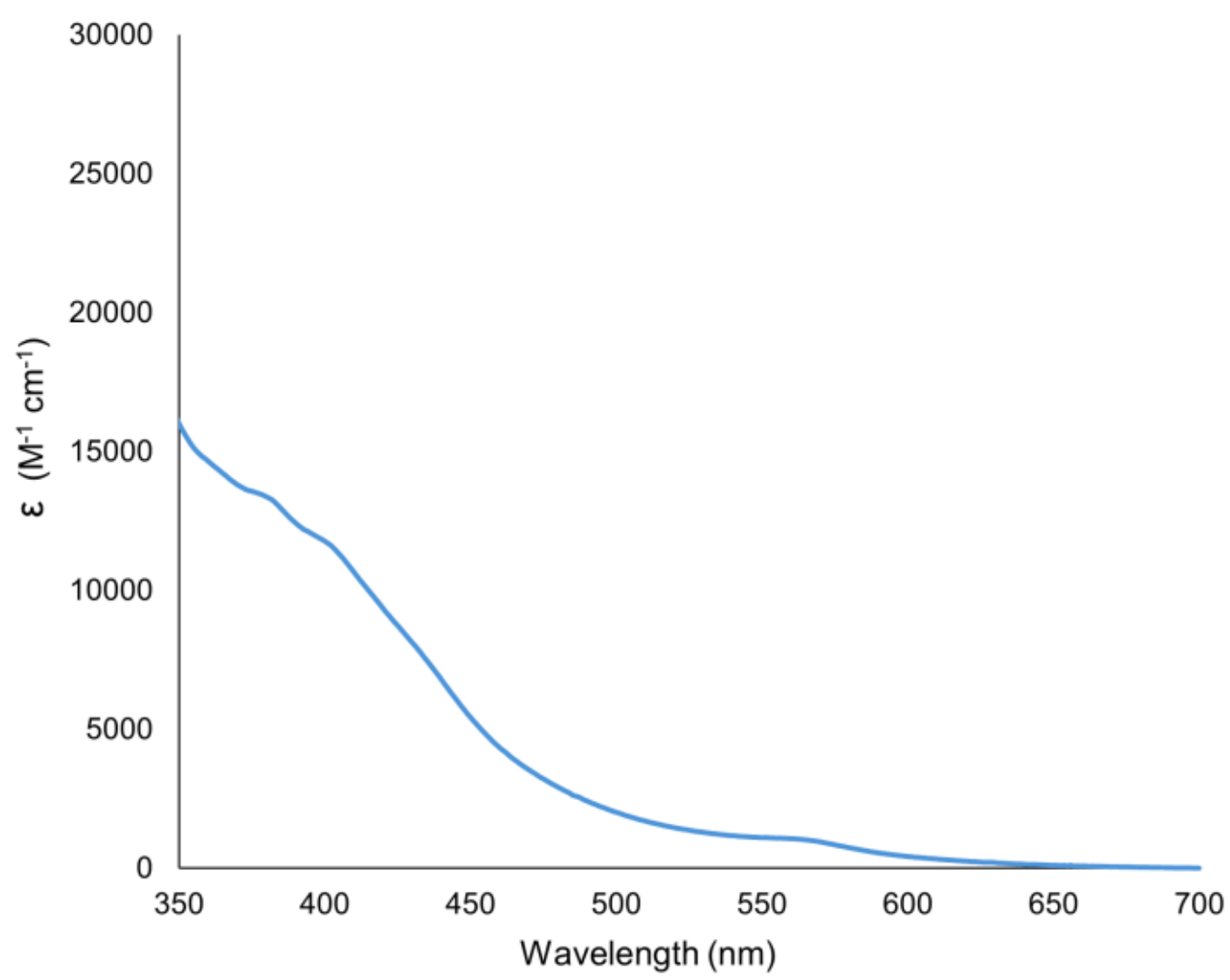


**Figure S9:** UV-Vis spectrum of **($^{Fluor}N_3$)Ni($CF_3$)$_2$** in benzene, zoomed in.

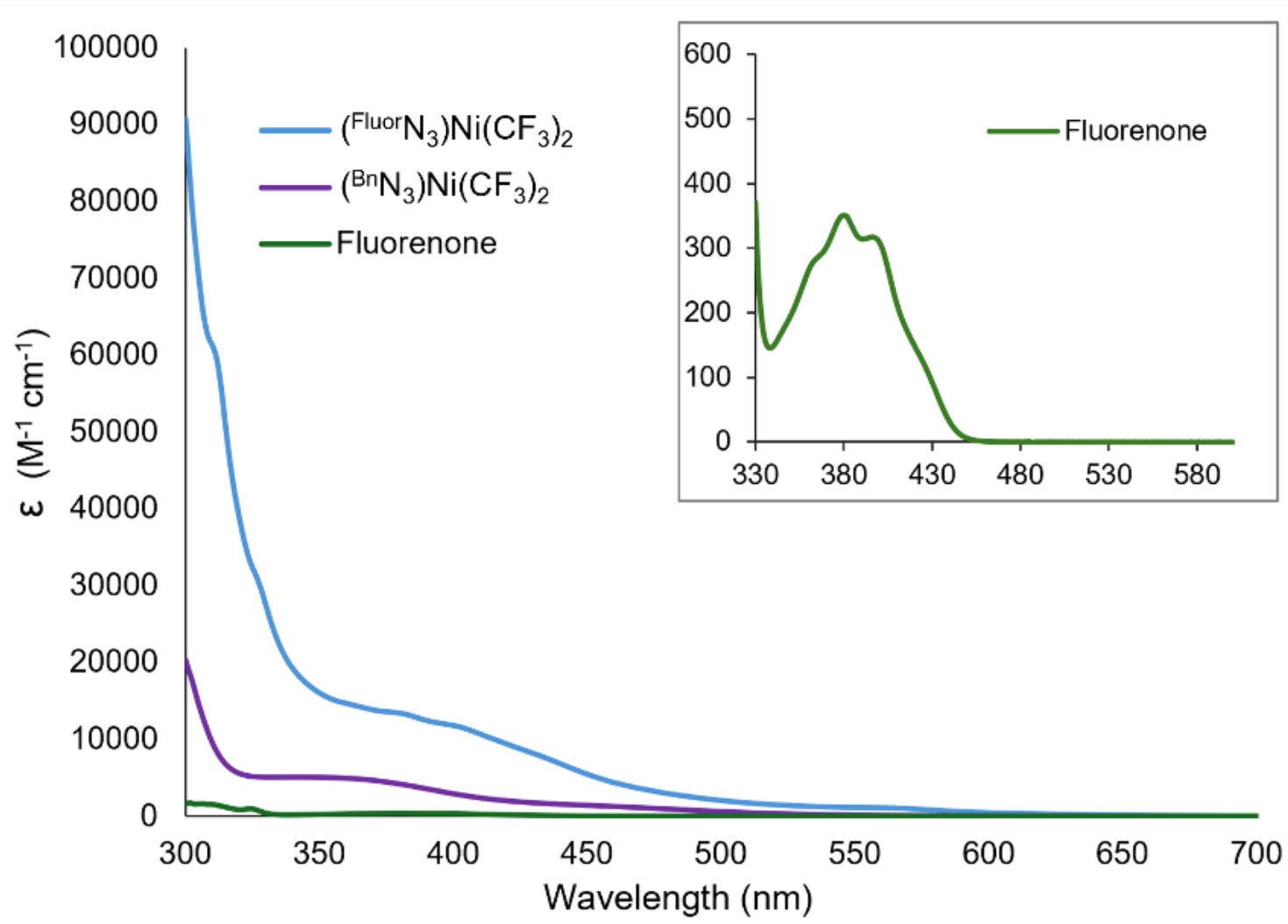


**Figure S10:** Stacked UV-Vis spectra in benzene of **($^{Fluor}N_3$)Ni($CF_3$)$_2$**, **($^{Bn}N_3$)Ni($CF_3$)$_2$**, and fluorenone.

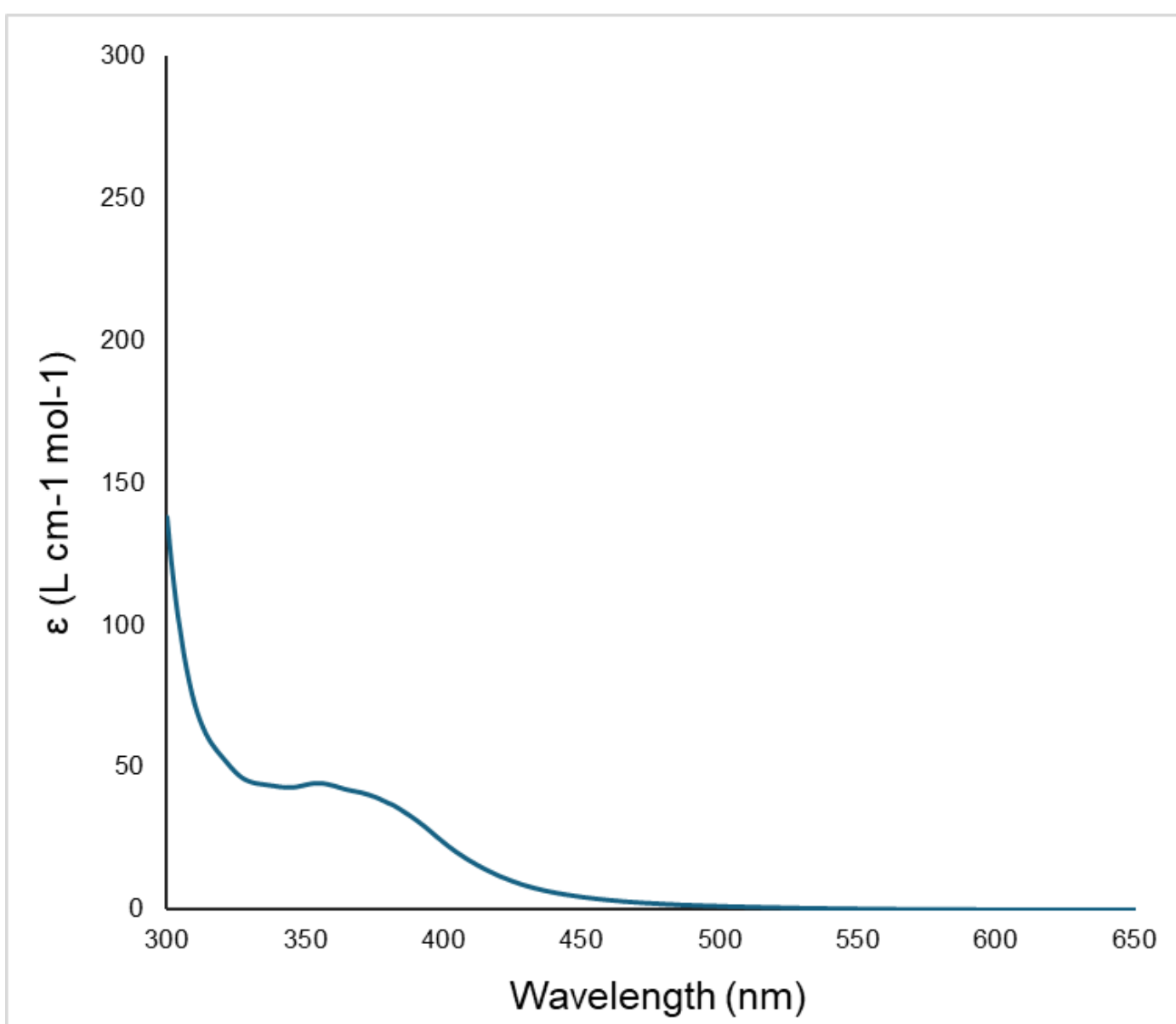


**Figure S11:** UV-Vis spectrum of **($^{Bn}N_3$)** in benzene.

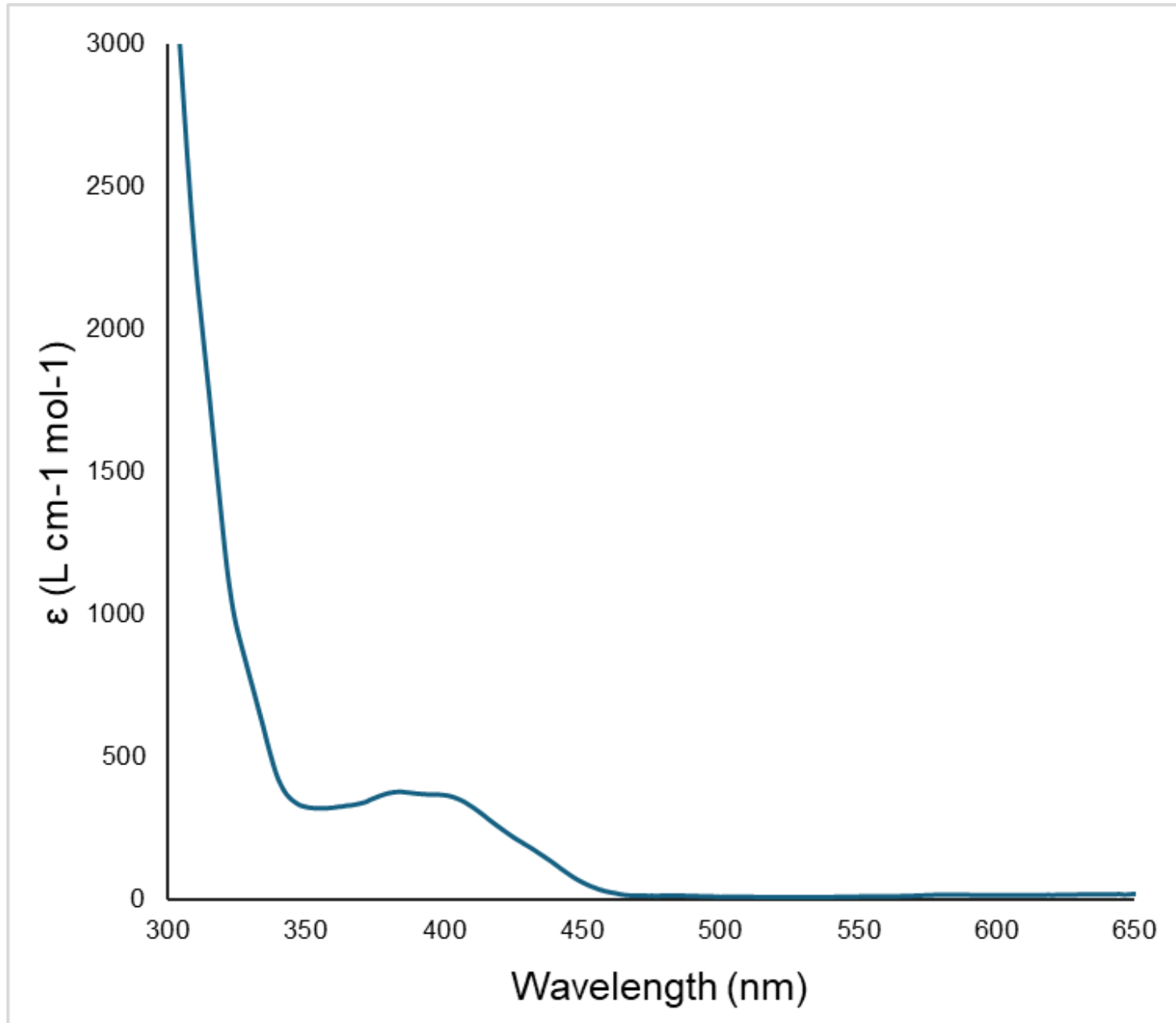


**Figure S12:** UV-Vis spectrum of **($^{Fluor}N_3$)** in benzene.

## VI. Preparation of Reaction Mixtures.

i. Preparation of hexafluorobenzene internal standard solution.

To a 5 mL volumetric flask was added 8 µL of hexafluorobenzene with a microsyringe. The volume of the solution was brought to 5 mL via the addition of benzene, bringing the concentration to 0.0138 M. This internal standard stock solution was used for all experiments.

ii. Preparation of reaction mixtures of **($^{Fluor}N_3$)Ni($CF_3$)$_2$**.

To a 20 mL scintillation vial was added 0.010 g (0.017 mmol) of **($^{Fluor}N_3$)Ni($CF_3$)$_2$**. 20 mL of benzene was added and the solution was agitated on a vortex plate to aid in solubility. The solution was filtered through glass wool and 50 µL of internal standard solution was added to the mixture. A small aliquot of the homogeneous solution was transferred to a J young tube and analyzed by $^{19}$F NMR after the addition of 5 drops of benzene-d6 (for NMR lock protocol). Integration by $^{19}$F NMR was used to back calculate the concentration of **($^{Fluor}N_3$)Ni($CF_3$)$_2$** to be 0.032 mM.

iii. Preparation of reaction mixtures of **($^{Bn}N_3$)Ni($CF_3$)$_2$**.

To a 20 mL scintillation vial was added 0.008 g (0.017 mmol) of **($^{Bn}N_3$)Ni($CF_3$)$_2$**. 20 mL of benzene was added and the solution was agitated on a vortex plate to aid in solubility. The solution was filtered through glass wool and 50 µL of internal standard solution was added to the mixture. A small aliquot of the homogeneous solution was transferred to a J young tube and analyzed by $^{19}$F NMR after the addition of 5 drops of benzene-d6 (for NMR lock protocol). Integration by $^{19}$F NMR was used to back calculate the concentration of **($^{Bn}N_3$)Ni($CF_3$)$_2$** to be 0.173 mM. Accordingly, the solution was diluted to a concentration of 0.032 mM to mimic the reaction concentration of **($^{Fluor}N_3$)Ni($CF_3$)$_2$**.

iv. Preparation of TEMPO stock solution.

To a 20 mL scintillation vial was added 0.020 g (0.128 mmol) of TEMPO. 10 mL of benzene was added to give a concentration of 0.0128 M.

## VII. Reactivity Under Thermal Heating.

i. Probing complex stability at room temperature in benzene solution

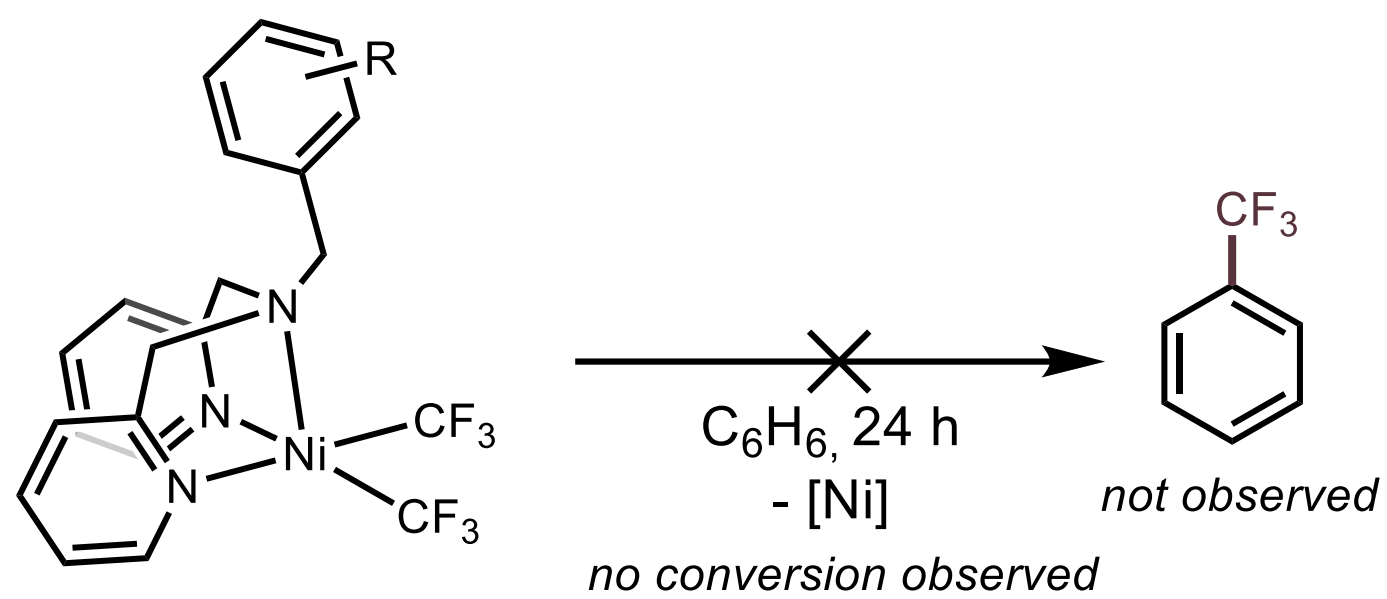


To a 20 mL scintillation vial was added 4 mL of a 0.032 mM solution of **($^{Fluor}N_3$)Ni($CF_3$)$_2$** in benzene containing hexafluorobenzene as an internal standard. A small aliquot of the homogeneous solution was transferred to a J young tube and analyzed by $^{19}$F NMR after the addition of 5 drops of benzene-d6 (for NMR lock protocol). After sitting in the dark for 24 hours, the J young tube was again analyzed by $^{19}$F NMR.

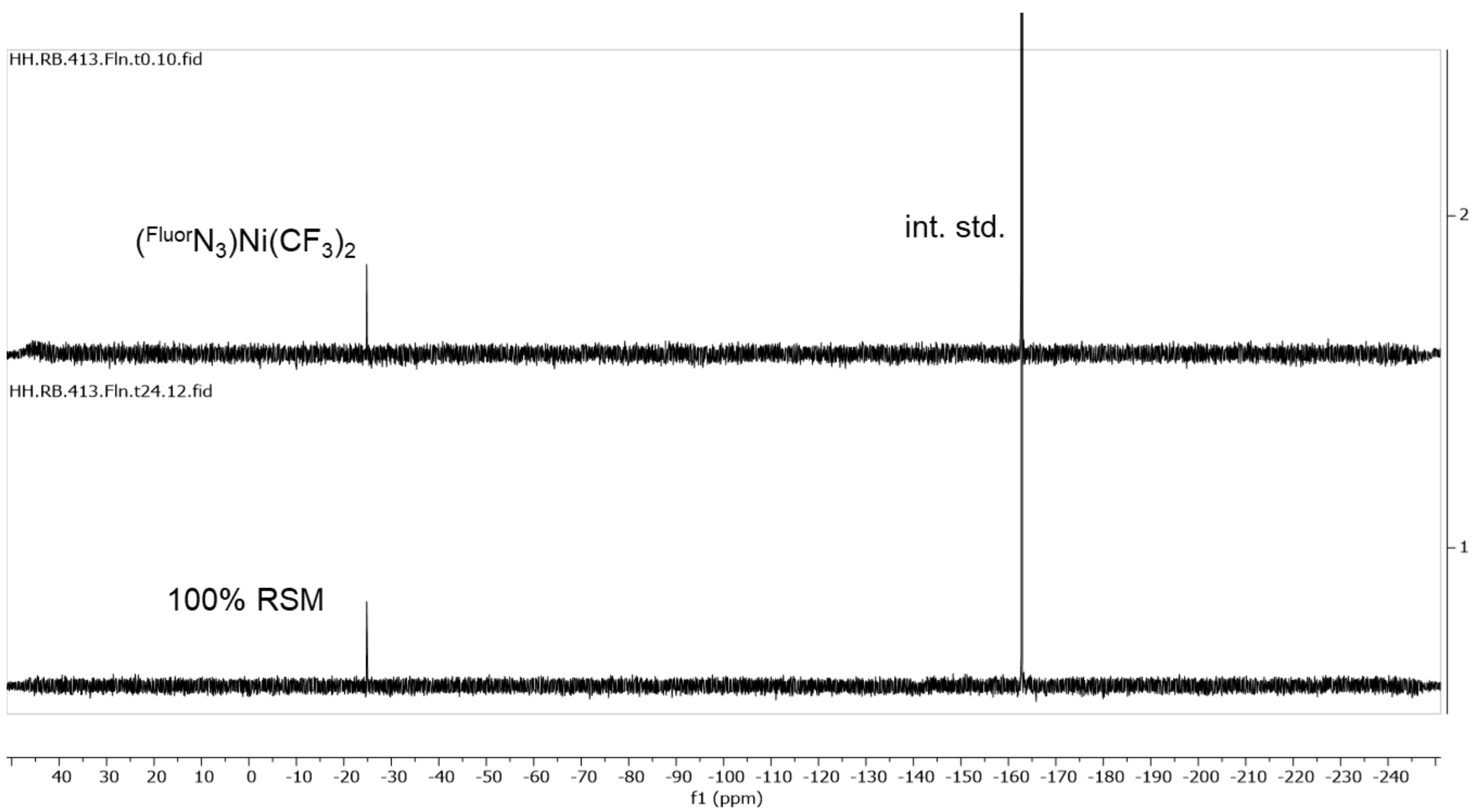


**Figure S13**: **($^{Fluor}N_3$)Ni($CF_3$)$_2$** $^{19}$F NMR of reaction mixture at T = 0 (top) and T = 24 h (bottom), showing 100% RSM after sitting in a benzene solution in the dark for 24 hours, using hexafluorobenzene as an internal standard (471 MHz, $C_6D_6$).

To a 20 mL scintillation vial was added 4 mL of a 0.032 mM solution of **($^{Bn}N_3$)Ni($CF_3$)$_2$** in benzene containing hexafluorobenzene as an internal standard. A small aliquot of the homogeneous solution was transferred to a J young tube and analyzed by $^{19}F$ NMR after the addition of 5 drops of benzene-d6 (for NMR lock protocol). After sitting in the dark for 24 hours, the J young tube was again analyzed by $^{19}F$ NMR.

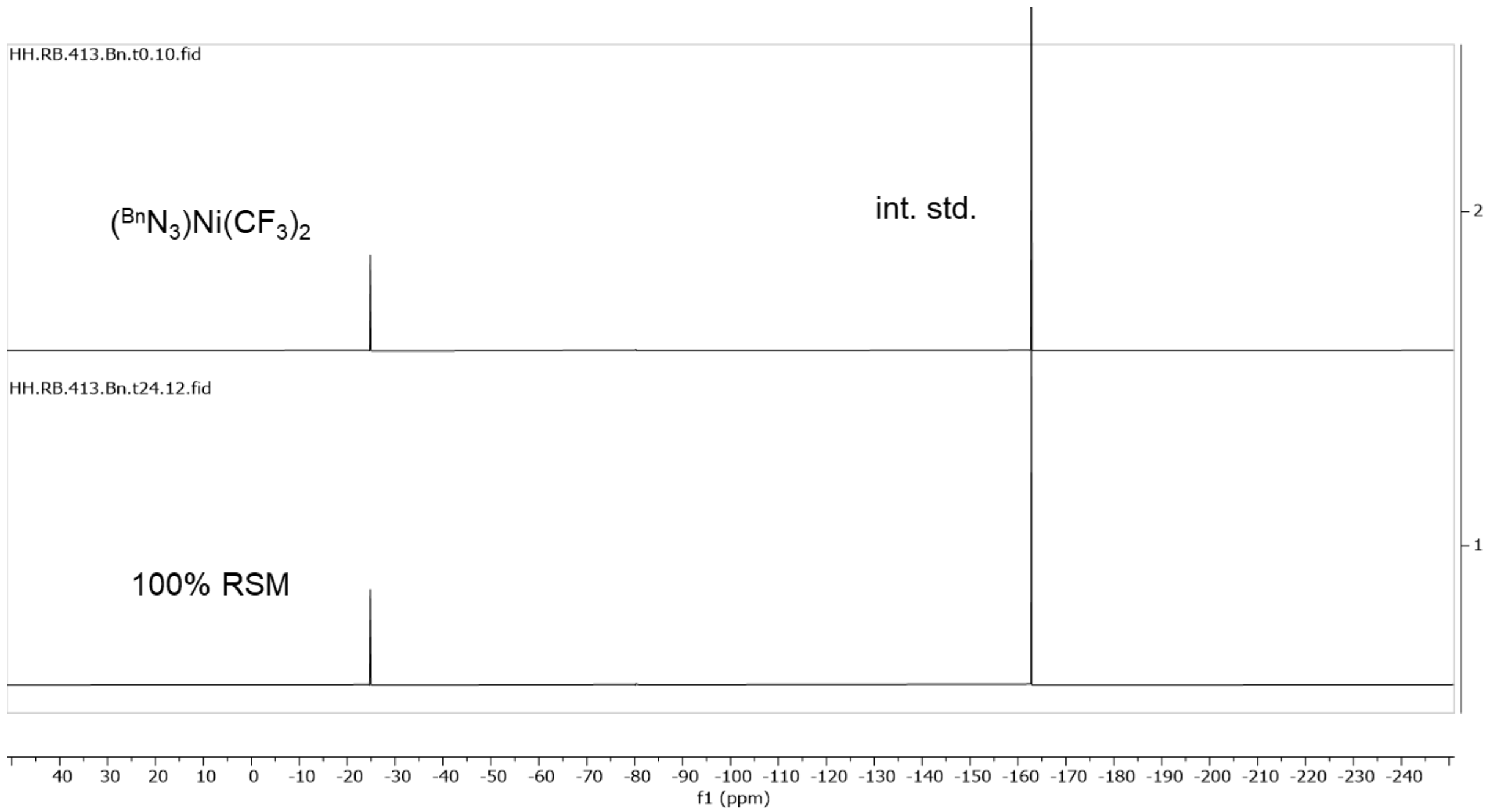


**Figure S14**: **($^{Bn}N_3$)Ni($CF_3$)$_2$** $^{19}F$ NMR of reaction mixture at T = 0 (top) and T = 24 h (bottom), showing 100% RSM after sitting in a benzene solution in the dark for 24 hours, using hexafluorobenzene as an internal standard (471 MHz, $C_6D_6$).

ii. Radical trapping room temperature reactions in benzene

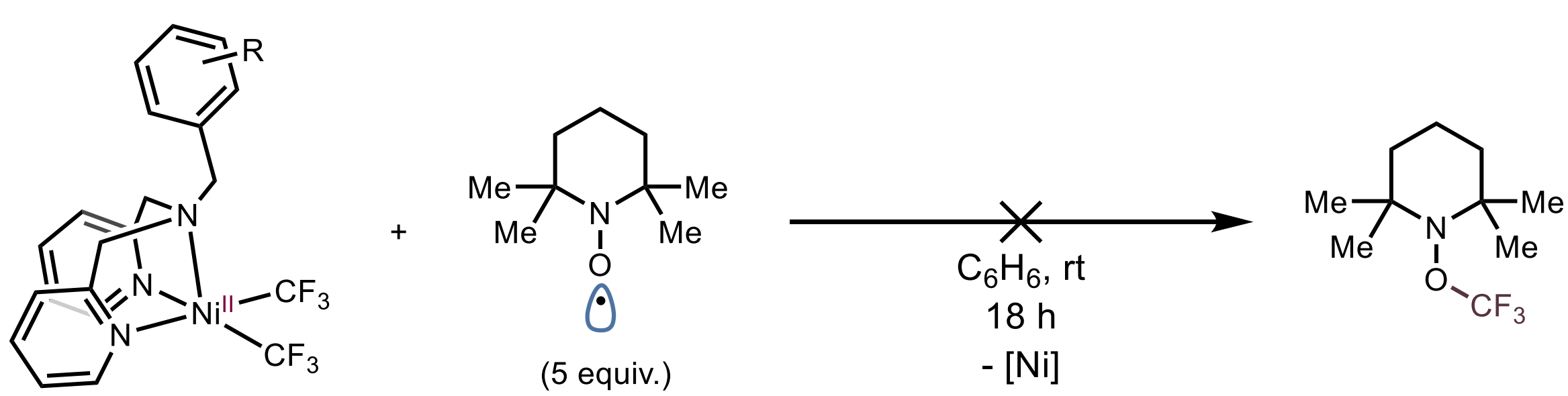

To a 20 mL scintillation vial was added 4 mL of a 0.032 mM solution of **($^{Fluor}N_3$)Ni($CF_3$)$_2$** in benzene containing hexafluorobenzene as an internal standard. 5 equivalents of TEMPO (0.00064 mmol) were added via the addition of 0.05 mL of a 0.0128 M stock solution. A small aliquot of the homogeneous solution was transferred to a J young tube and analyzed by $^{19}$F NMR after the addition of 5 drops of benzene-d6 (for NMR lock protocol). After sitting in the dark for 18 hours, the J young tube was again analyzed by $^{19}$F NMR.

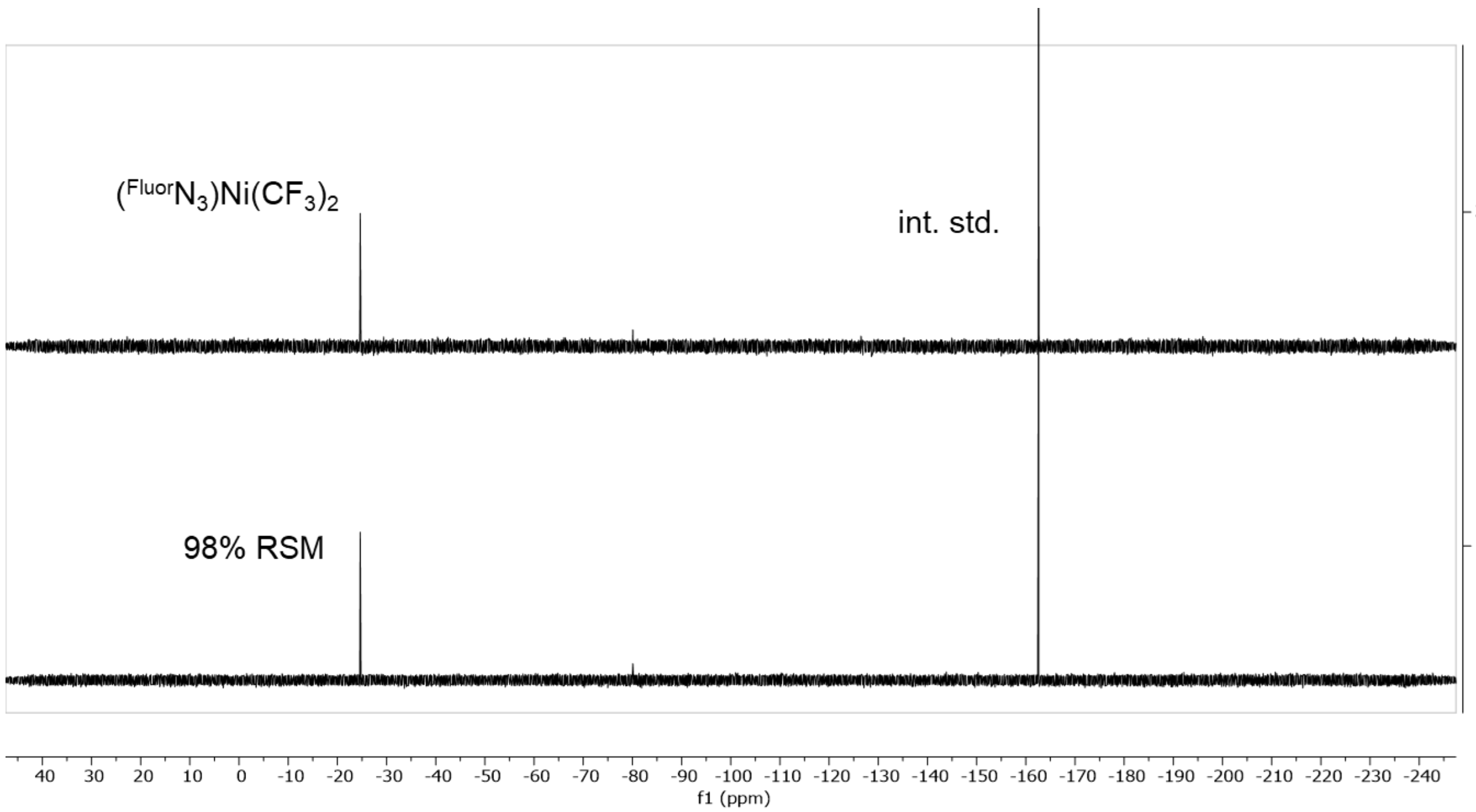


**Figure S15**: **($^{Fluor}N_3$)Ni($CF_3$)$_2$** $^{19}$F NMR of reaction mixture at T = 0 (top) and T = 18 h (bottom), showing 98% RSM and 0% yield of trifluorotoluene or TEMPO-$CF_3$ adduct after sitting in a benzene solution in the dark for 18 hours, using hexafluorobenzene as an internal standard (471 MHz, $C_6D_6$).

To a 20 mL scintillation vial was added 4 mL of a 0.032 mM solution of **($^{Bn}N_3$)Ni($CF_3$)$_2$** in benzene containing hexafluorobenzene as an internal standard. 5 equivalents of TEMPO (0.00064 mmol) were added via the addition of 0.05 mL of a 0.0128 M stock solution. A small aliquot of the homogeneous solution was transferred to a J young tube and analyzed by $^{19}$F NMR after the addition of 5 drops of benzene-d6 (for NMR lock protocol). After sitting in the dark for 18 hours, the J young tube was again analyzed by $^{19}$F NMR.

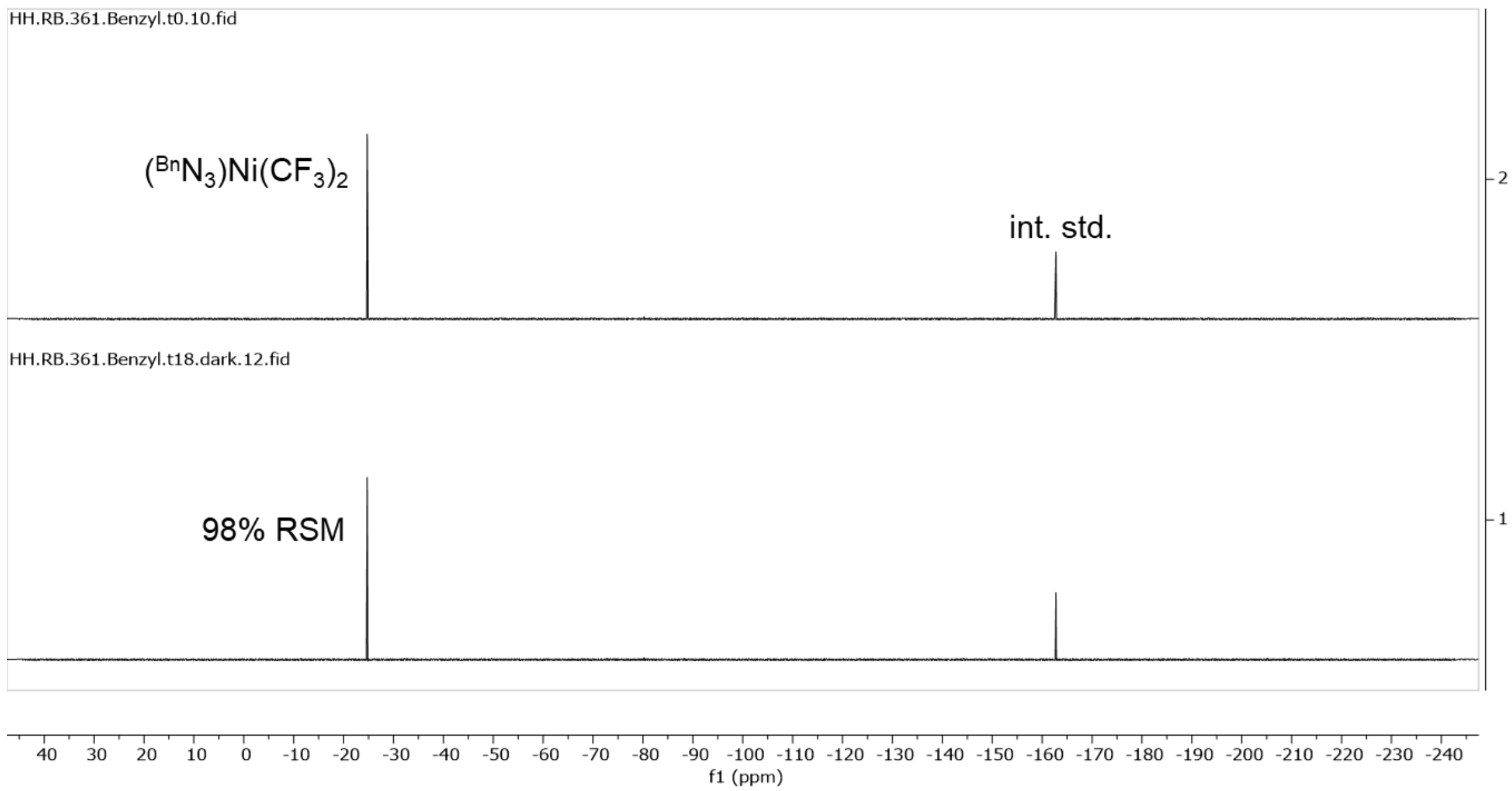


**Figure S16**: **($^{Bn}N_3$)Ni($CF_3$)$_2$** $^{19}F$ NMR of reaction mixture at T = 0 (top) and T = 18 h (bottom), showing 98% RSM and 0% yield of trifluorotoluene or TEMPO-$CF_3$ adduct after sitting in a benzene solution in the dark for 18 hours, using hexafluorobenzene as an internal standard (471 MHz, $C_6D_6$).

iii. Thermal reactions in benzene

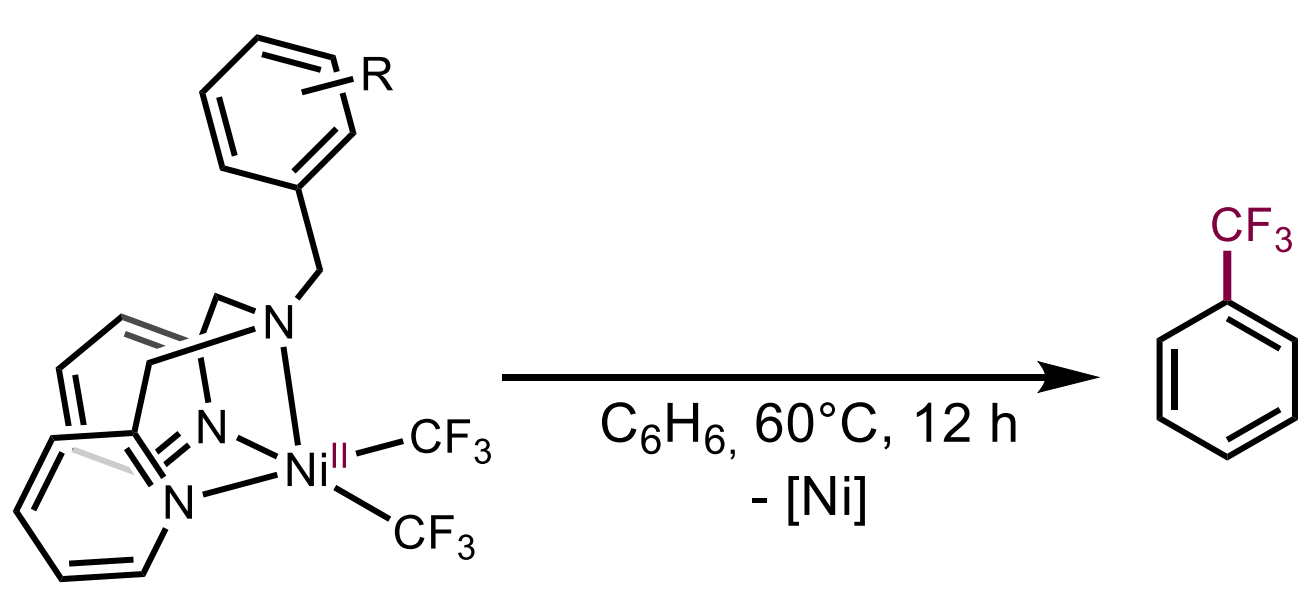


To a 20 mL scintillation vial was added 4 mL of a 0.032 mM solution of **($^{Fluor}N_3$)Ni($CF_3$)$_2$** in benzene containing hexafluorobenzene as an internal standard. A small aliquot of the homogeneous solution was transferred to a J young tube and analyzed by $^{19}F$ NMR after the addition of 5 drops of benzene-d6 (for NMR lock protocol). A stir bar was added to the reaction vial; the vial was capped and rigorously sealed with electrical tape before being removed from the glovebox and placed in an aluminum bead bath heated to 60°C. The vials were allowed to stir at this temperature for 12 hours before being brought back into the glovebox. An aliquot of the homogeneous reaction

mixture was analyzed by $^{19}F$ NMR after the addition of 5 drops of benzene-d6 (for NMR lock protocol).

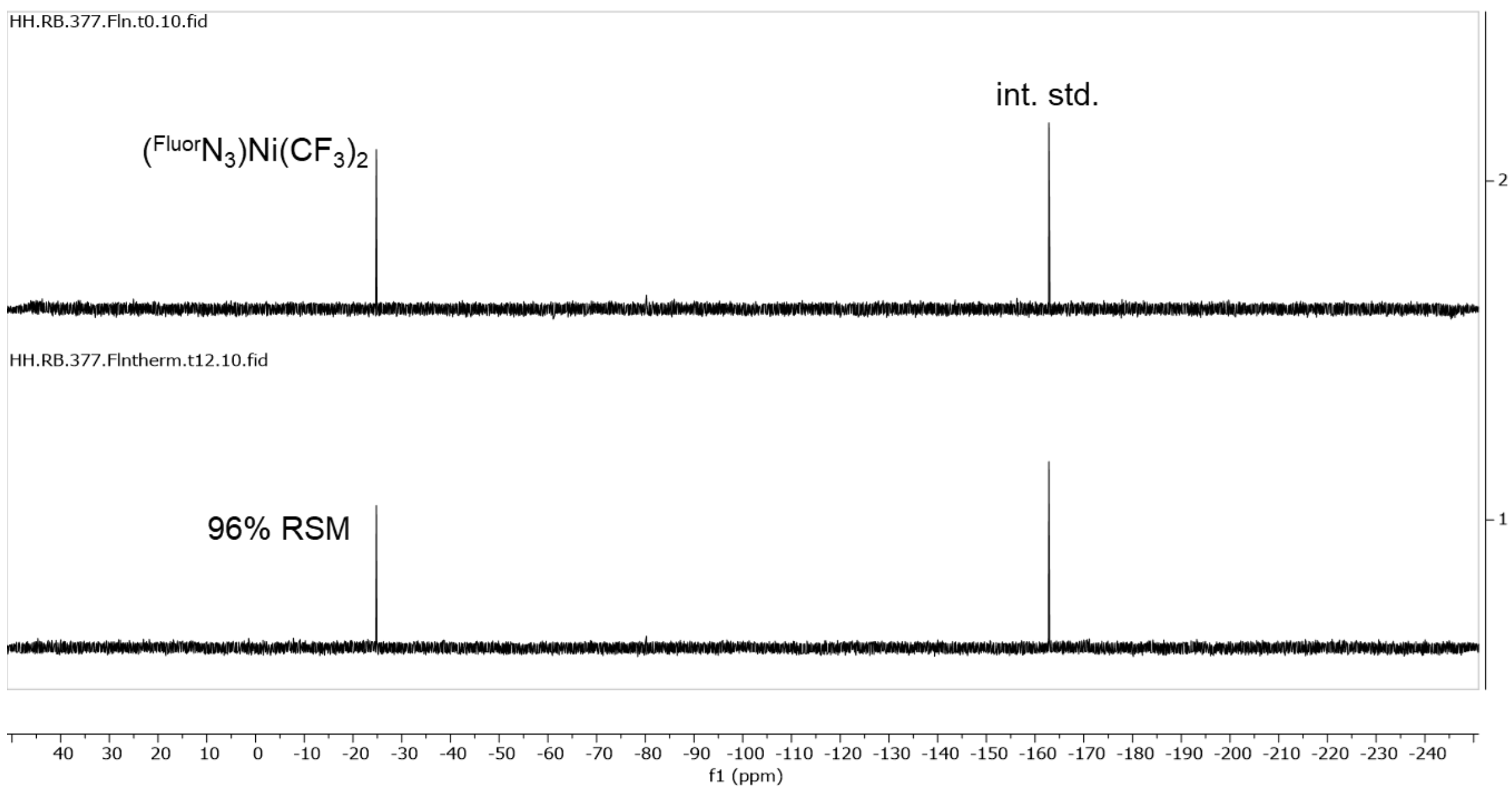


**Figure S17**: **($^{Fluor}N_3$)Ni($CF_3$)$_2$** $^{19}F$ NMR of reaction mixture at T = 0 (top) and T = 12 h (bottom), showing 96% RSM and 0% yield of trifluorotoluene after heating to 60°C for 12 h in benzene, using hexafluorobenzene as an internal standard (471 MHz, $C_6D_6$).

To a 20 mL scintillation vial was added 4 mL of a 0.032 mM solution of **($^{Bn}N_3$)Ni($CF_3$)$_2$** in benzene containing hexafluorobenzene as an internal standard. A small aliquot of the homogeneous solution was transferred to a J young tube and analyzed by $^{19}F$ NMR after the addition of 5 drops of benzene-d6 (for NMR lock protocol). A stir bar was added to the reaction vial; the vial was capped and rigorously sealed with electrical tape before being removed from the glovebox and placed in an aluminum bead bath heated to 60°C. The vials were allowed to stir at this temperature for 12 hours before being brought back into the glovebox. An aliquot of the homogeneous reaction mixture was analyzed by $^{19}F$ NMR after the addition of 5 drops of benzene-d6 (for NMR lock protocol).

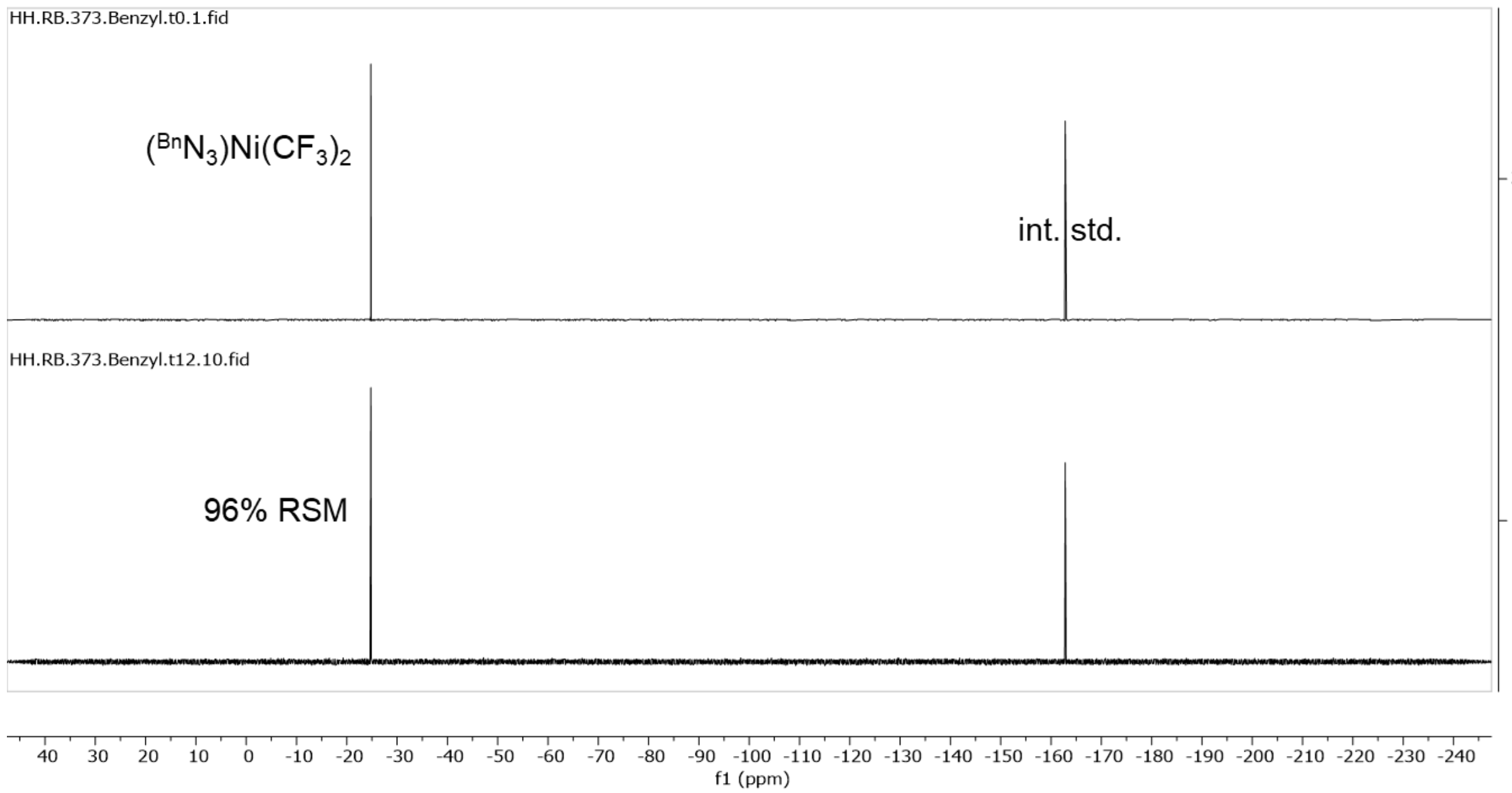


**Figure S18**: **($^{Bn}N_3$)Ni($CF_3$)$_2$** $^{19}$F NMR of reaction mixture at T = 0 (top) and T = 12 h (bottom), showing 96% RSM and 0% yield of trifluorotoluene after heating to 60°C for 12 h in benzene, using hexafluorobenzene as an internal standard (471 MHz, $C_6D_6$).

iv. Radical trapping thermal reactions in benzene

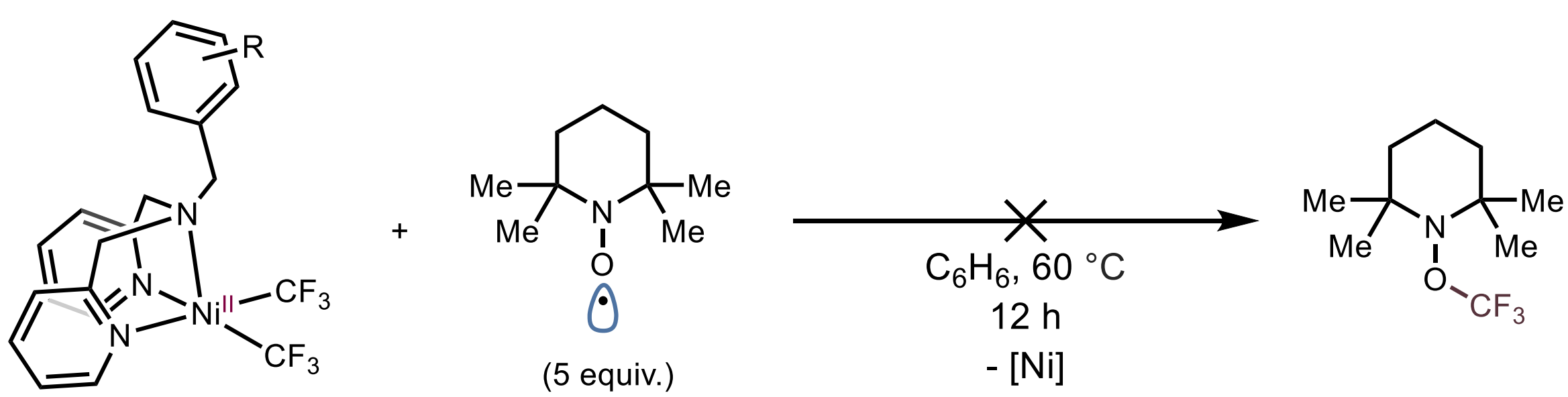


To a 20 mL scintillation vial was added 4 mL of a 0.032 mM solution of **($^{Fluor}N_3$)Ni($CF_3$)$_2$** in benzene containing hexafluorobenzene as an internal standard. 5 equivalents of TEMPO (0.00064 mmol) were added via the addition of 0.05 mL of a 0.0128 M stock solution. A small aliquot of the homogeneous solution was transferred to a J young tube and analyzed by $^{19}$F NMR after the addition of 5 drops of benzene-d6 (for NMR lock protocol). A stir bar was added to the reaction vial; the vial was capped and rigorously sealed with electrical tape before being removed from the glovebox and placed in an aluminum bead bath heated to 60°C. The vials were allowed to stir at this temperature for 12 hours before being brought back into the glovebox. An aliquot of the

homogeneous reaction mixture was analyzed by $^{19}F$ NMR after the addition of 5 drops of benzene-d6 (for NMR lock protocol).

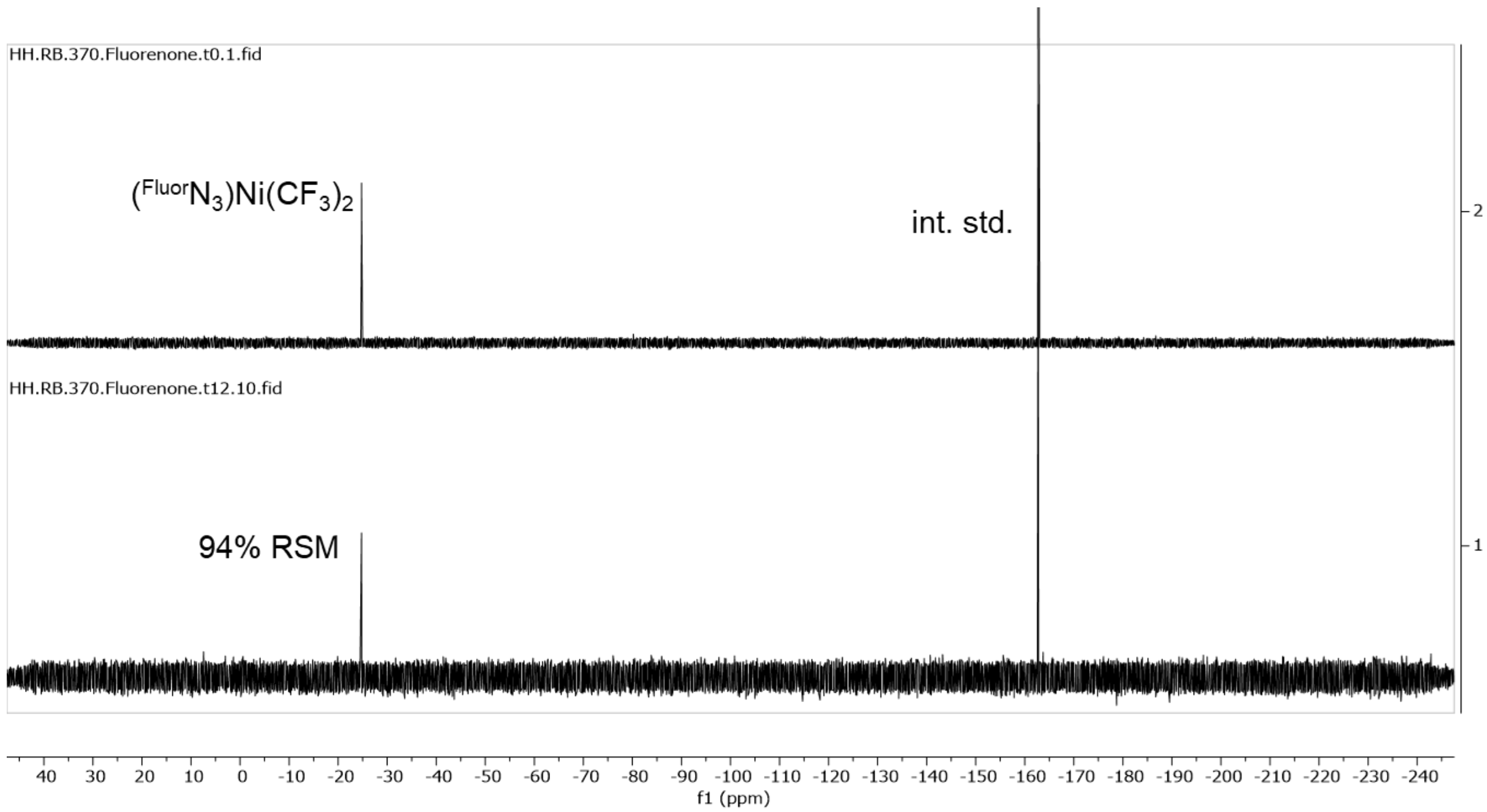


**Figure S19**: **$(^{Fluor}N_3)Ni(CF_3)_2$** $^{19}F$ NMR of reaction mixture at T = 0 (top) and T = 12 h (bottom), showing 94% RSM and 0% yield of trifluorotoluene or TEMPO-$CF_3$ adduct after being heated to 60°C for 12 hours , using hexafluorobenzene as an internal standard (471 MHz, $C_6D_6$).

To a 20 mL scintillation vial was added 4 mL of a 0.032 mM solution of **$(^{Bn}N_3)Ni(CF_3)_2$** in benzene containing hexafluorobenzene as an internal standard. 5 equivalents of TEMPO (0.00064 mmol) were added via the addition of 0.05 mL of a 0.0128 M stock solution. A small aliquot of the homogeneous solution was transferred to a J young tube and analyzed by $^{19}F$ NMR after the addition of 5 drops of benzene-d6 (for NMR lock protocol). A stir bar was added to the reaction vial; the vial was capped and rigorously sealed with electrical tape before being removed from the glovebox and placed in an aluminum bead bath heated to 60°C. The vials were allowed to stir at this temperature for 12 hours before being brought back into the glovebox. An aliquot of the homogeneous reaction mixture was analyzed by $^{19}F$ NMR after the addition of 5 drops of benzene-d6 (for NMR lock protocol).

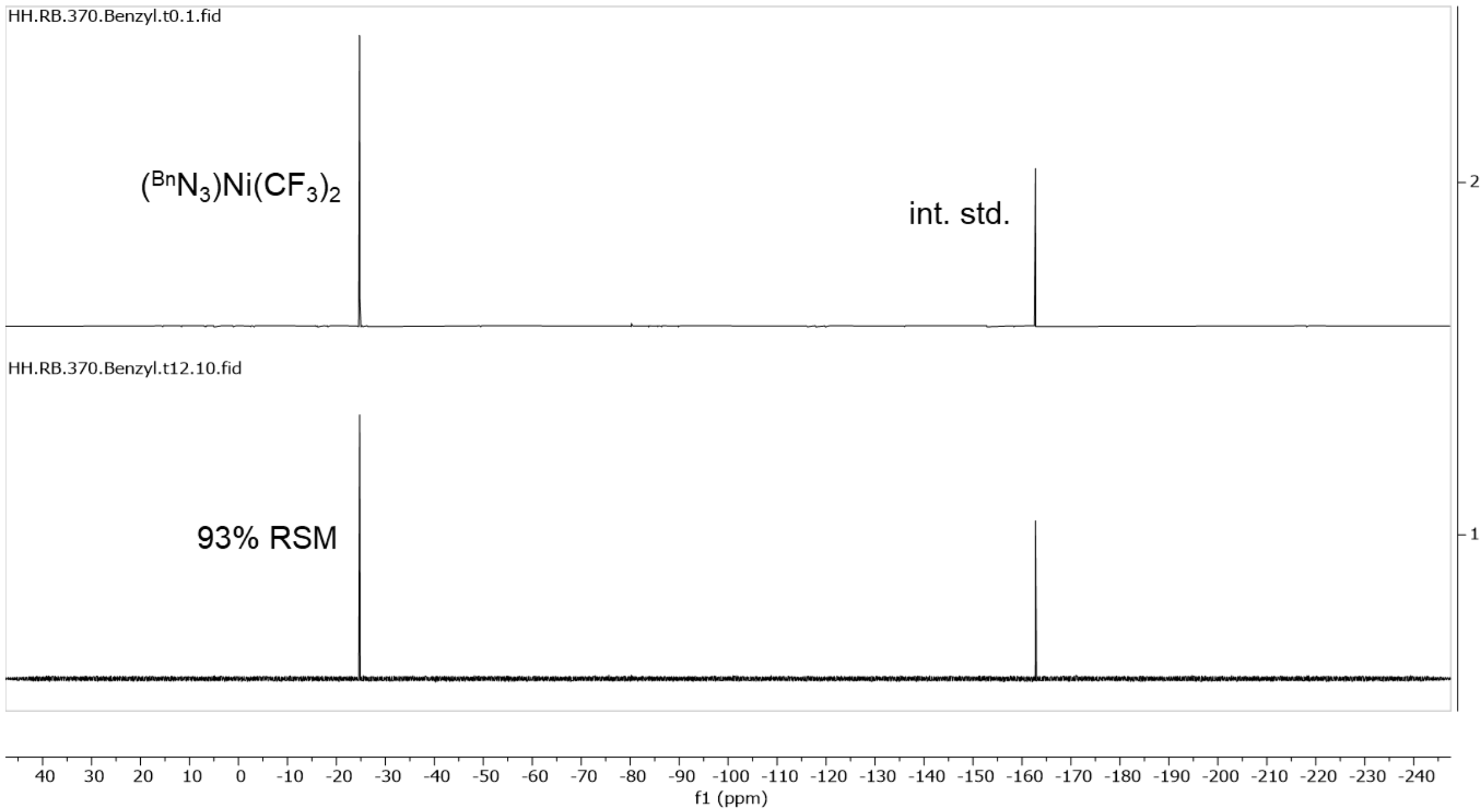


**Figure S20**: **($^{Bn}N_3$)Ni($CF_3$)$_2$** $^{19}F$ NMR of reaction mixture at T = 0 (top) and T = 12 h (bottom), showing 93% RSM and 0% yield of trifluorotoluene or TEMPO-$CF_3$ adduct after being heated to 60°C for 12 hours , using hexafluorobenzene as an internal standard (471 MHz, $C_6D_6$).

## VIII. Reactivity Under Visible Light Irradiation (405 nm, 365 nm).

i. 405 nm *hv* reactions

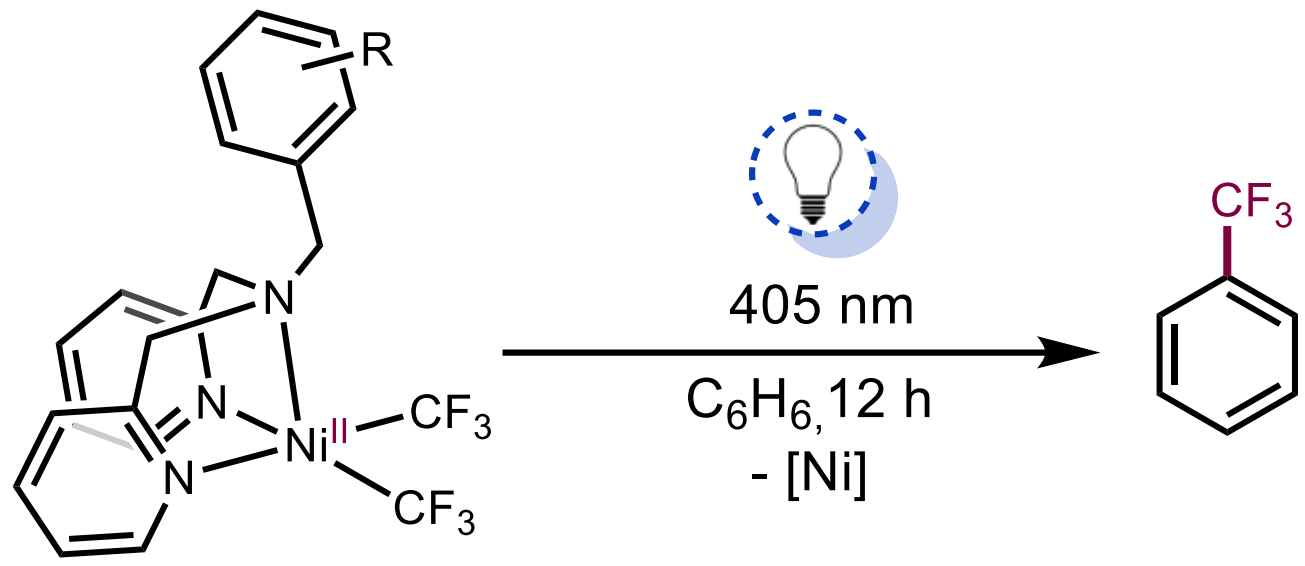


To a 20 mL scintillation vial was added 4 mL of a 0.032 mM solution of **($^{Fluor}$N$_3$)Ni(CF$_3$)$_2$** in benzene containing hexafluorobenzene as an internal standard. A small aliquot of the homogeneous solution was analyzed by $^{19}$F NMR after the addition of 5 drops of benzene-d6 (for NMR lock protocol). A stir bar was added to the reaction vial, the vial was capped and rigorously sealed with electrical tape before being removed from the glovebox and placed in the Hepatochem Lucent 360 photoreactor. The photoreactor was equipped with four 405 nm bulbs, with intensity set to 20%. A temperature of 35°C was maintained throughout the reaction with the use of an external Julabo chiller connected to the photoreactor water bath. The vials were irradiated with stirring for 12 hours before being brought back into the glovebox. An aliquot of the homogeneous reaction mixture was analyzed by $^{19}$F NMR after the addition of 5 drops of benzene-d6 (for NMR lock protocol).

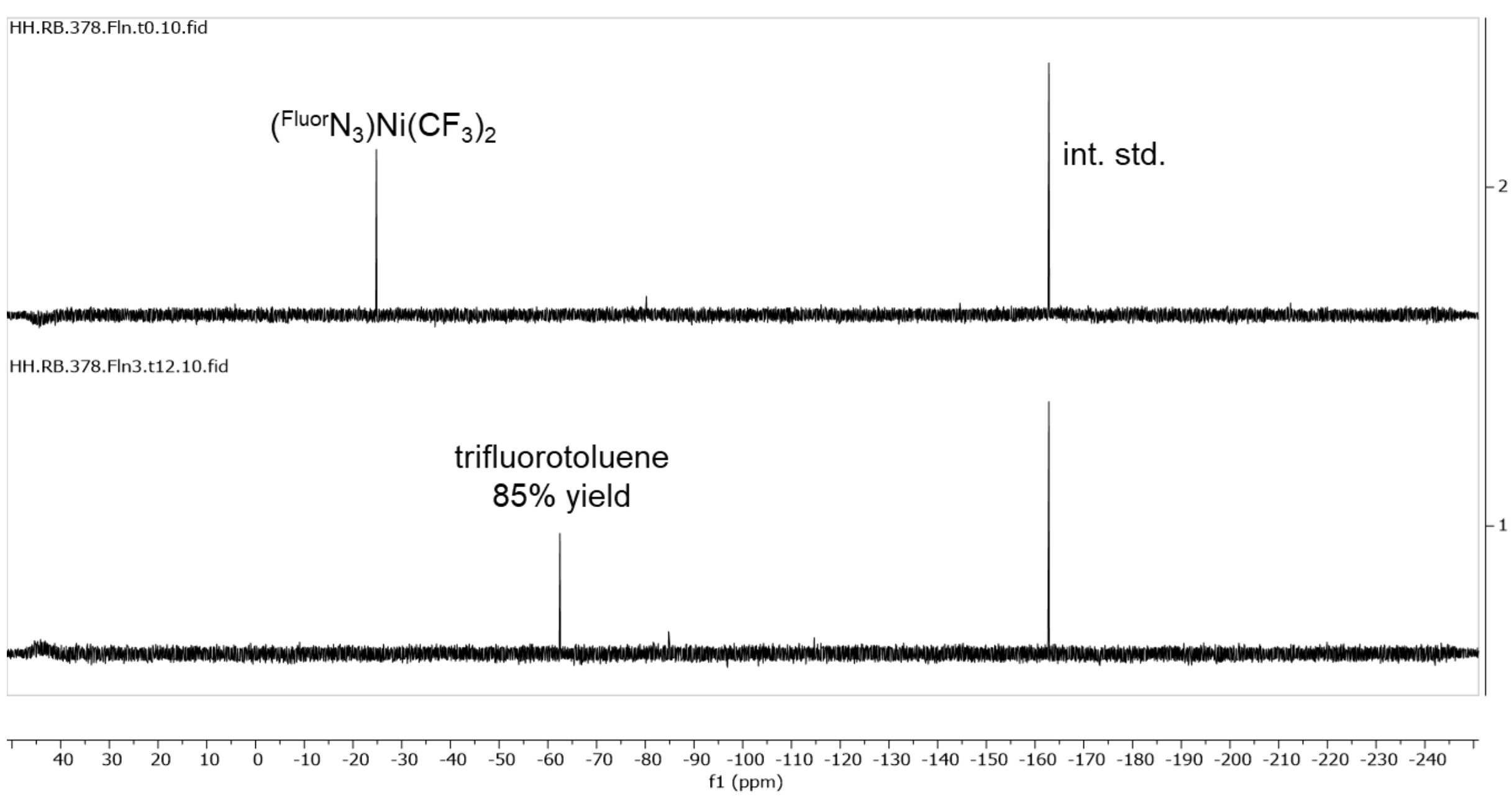

**Figure S21**: **($^{Fluor}N_3$)Ni($CF_3$)$_2$** $^{19}$F NMR of reaction mixture at T = 0 (top) and T = 12 h (bottom), showing 85% yield of trifluorotoluene after 405 nm irradiation for 12 h in benzene, using hexafluorobenzene as an internal standard (471 MHz, $C_6D_6$).

To a 20 mL scintillation vial was added 4 mL of a 0.032 mM solution of **($^{Bn}N_3$)Ni($CF_3$)$_2$** in benzene containing hexafluorobenzene as an internal standard. A small aliquot of the homogeneous solution was analyzed by $^{19}$F NMR after the addition of 5 drops of benzene-d6 (for NMR lock protocol). A stir bar was added to the reaction vial, the vial was capped and rigorously sealed with electrical tape before being removed from the glovebox and placed in the Hepatochem Lucent 360 photoreactor. The photoreactor was equipped with four 405 nm bulbs, with intensity set to 20%. A temperature of 35°C was maintained throughout the reaction with the use of an external Julabo chiller connected to the photoreactor water bath. The vials were irradiated with stirring for 12 hours before being brought back into the glovebox. An aliquot of the homogeneous reaction mixture was analyzed by $^{19}$F NMR after the addition of 5 drops of benzene-d6 (for NMR lock protocol).

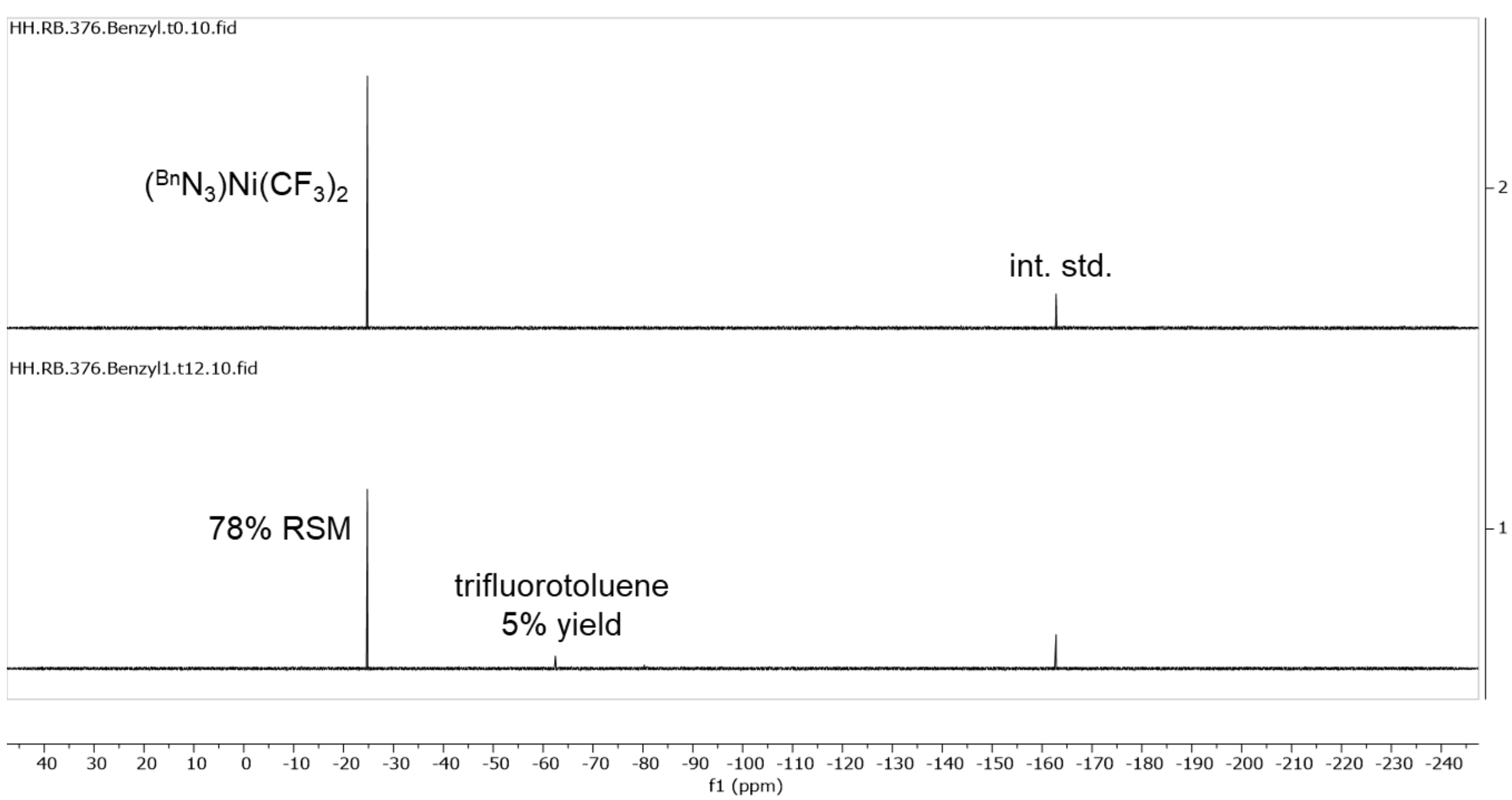


**Figure S22**: **($^{Bn}N_3$)Ni($CF_3$)$_2$** $^{19}$F NMR of reaction mixture at T = 0 (top) and T = 12 h (bottom), showing 5% yield of trifluorotoluene after 405 nm irradiation for 12 h in benzene, using hexafluorobenzene as an internal standard (471 MHz, $C_6D_6$).

ii. Radical trapping under 405 nm *hv*.

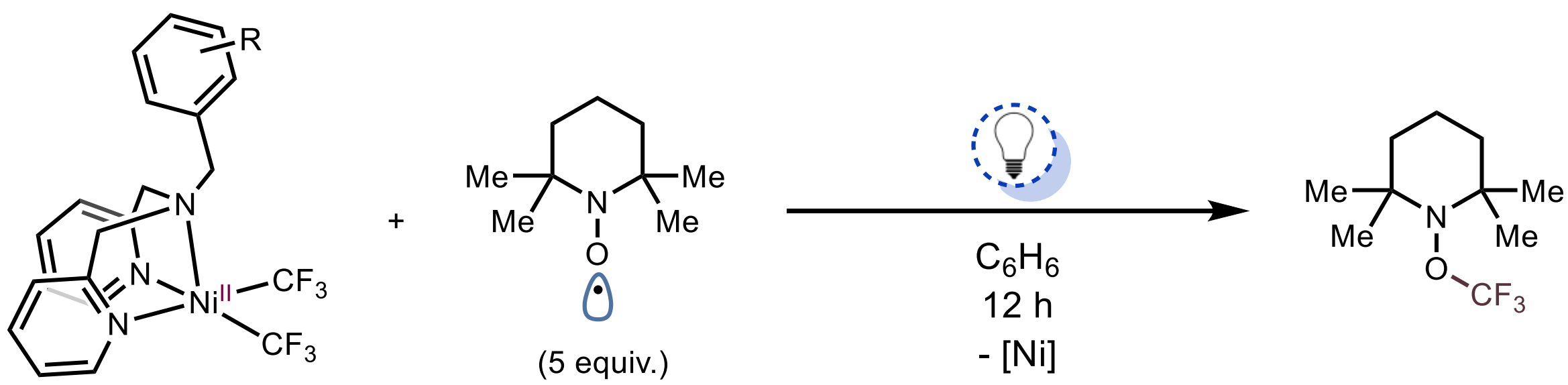


To a 20 mL scintillation vial was added 4 mL of a 0.032 mM solution of **($^{Fluor}N_3$)Ni($CF_3$)$_2$** in benzene containing hexafluorobenzene as an internal standard. 5 equivalents of TEMPO (0.00064 mmol) were added via the addition of 0.05 mL of a 0.0128 M stock solution. A small aliquot of the homogeneous solution was transferred to a J young tube and analyzed by $^{19}F$ NMR after the addition of 5 drops of benzene-d6 (for NMR lock protocol). A stir bar was added to the reaction vial, the vial was capped and rigorously sealed with electrical tape before being removed from the glovebox and placed in the Hepatochem Lucent 360 photoreactor. The photoreactor was equipped with four 405 nm bulbs, with intensity set to 20%. A temperature of 35°C was maintained throughout the reaction with the use of an external Julabo chiller connected to the photoreactor water bath. The vials were irradiated with stirring for 12 hours before being brought back into the glovebox. An aliquot of the homogeneous reaction mixture was analyzed by $^{19}F$ NMR after the addition of 5 drops of benzene-d6 (for NMR lock protocol).

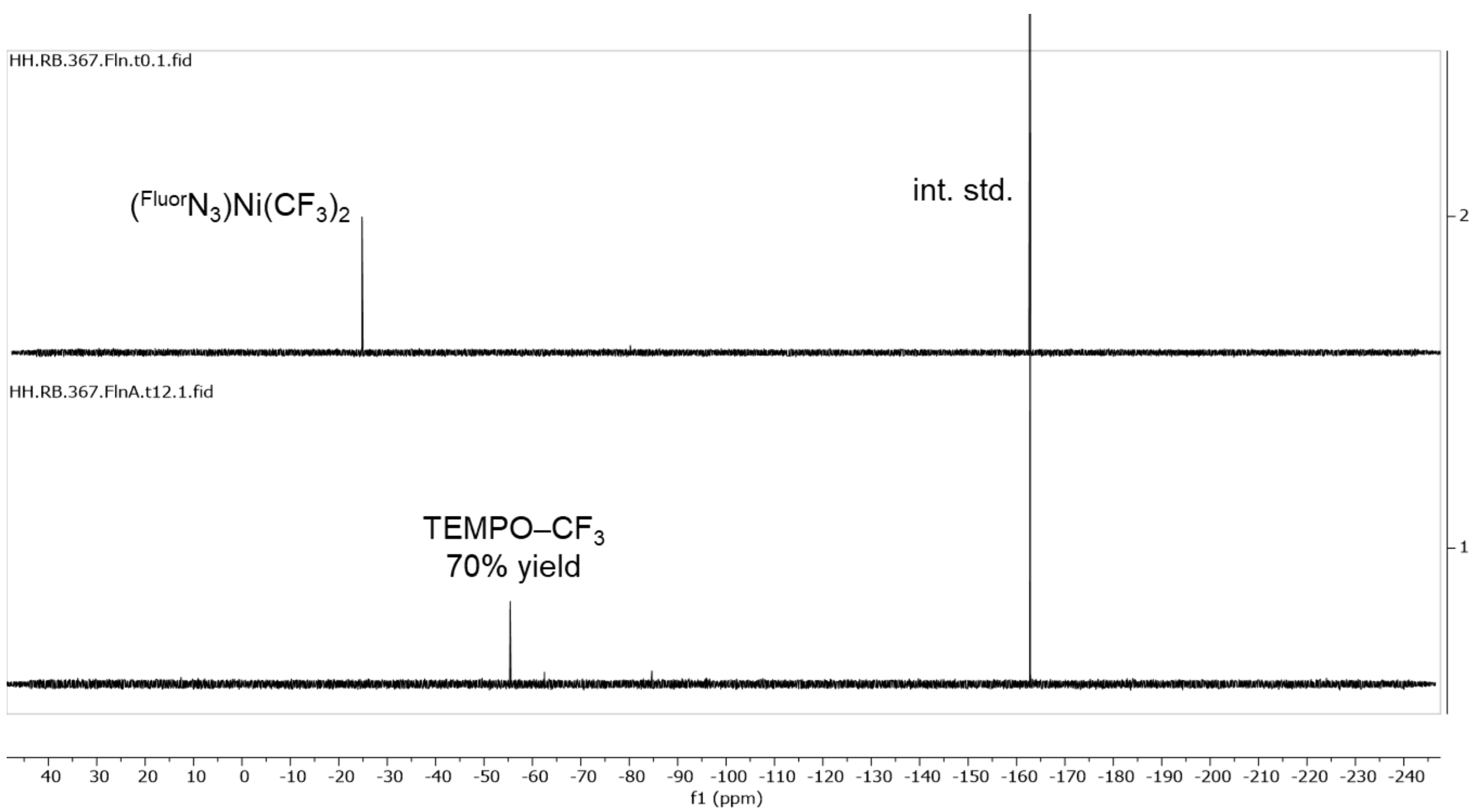

**Figure S23**: **($^{Fluor}N_3$)Ni($CF_3$)$_2$** $^{19}$F NMR of reaction mixture at T = 0 (top) and T = 12 h (bottom), showing 70% yield of TEMPO-$CF_3$ adduct and 0% yield of trifluorotoluene after 405 nm irradiation for 12 h in benzene, using hexafluorobenzene as an internal standard (471 MHz, $C_6D_6$).

To a 20 mL scintillation vial was added 4 mL of a 0.032 mM solution of **($^{Bn}N_3$)Ni($CF_3$)$_2$** in benzene containing hexafluorobenzene as an internal standard. 5 equivalents of TEMPO (0.00064 mmol) were added via the addition of 0.05 mL of a 0.0128 M stock solution. A small aliquot of the homogeneous solution was transferred to a J young tube and analyzed by $^{19}$F NMR after the addition of 5 drops of benzene-d6 (for NMR lock protocol). A stir bar was added to the reaction vial, the vial was capped and rigorously sealed with electrical tape before being removed from the glovebox and placed in the Hepatochem Lucent 360 photoreactor. The photoreactor was equipped with four 405 nm bulbs, with intensity set to 20%. A temperature of 35°C was maintained throughout the reaction with the use of an external Julabo chiller connected to the photoreactor water bath. The vials were irradiated with stirring for 12 hours before being brought back into the glovebox. An aliquot of the homogeneous reaction mixture was analyzed by $^{19}$F NMR after the addition of 5 drops of benzene-d6 (for NMR lock protocol).

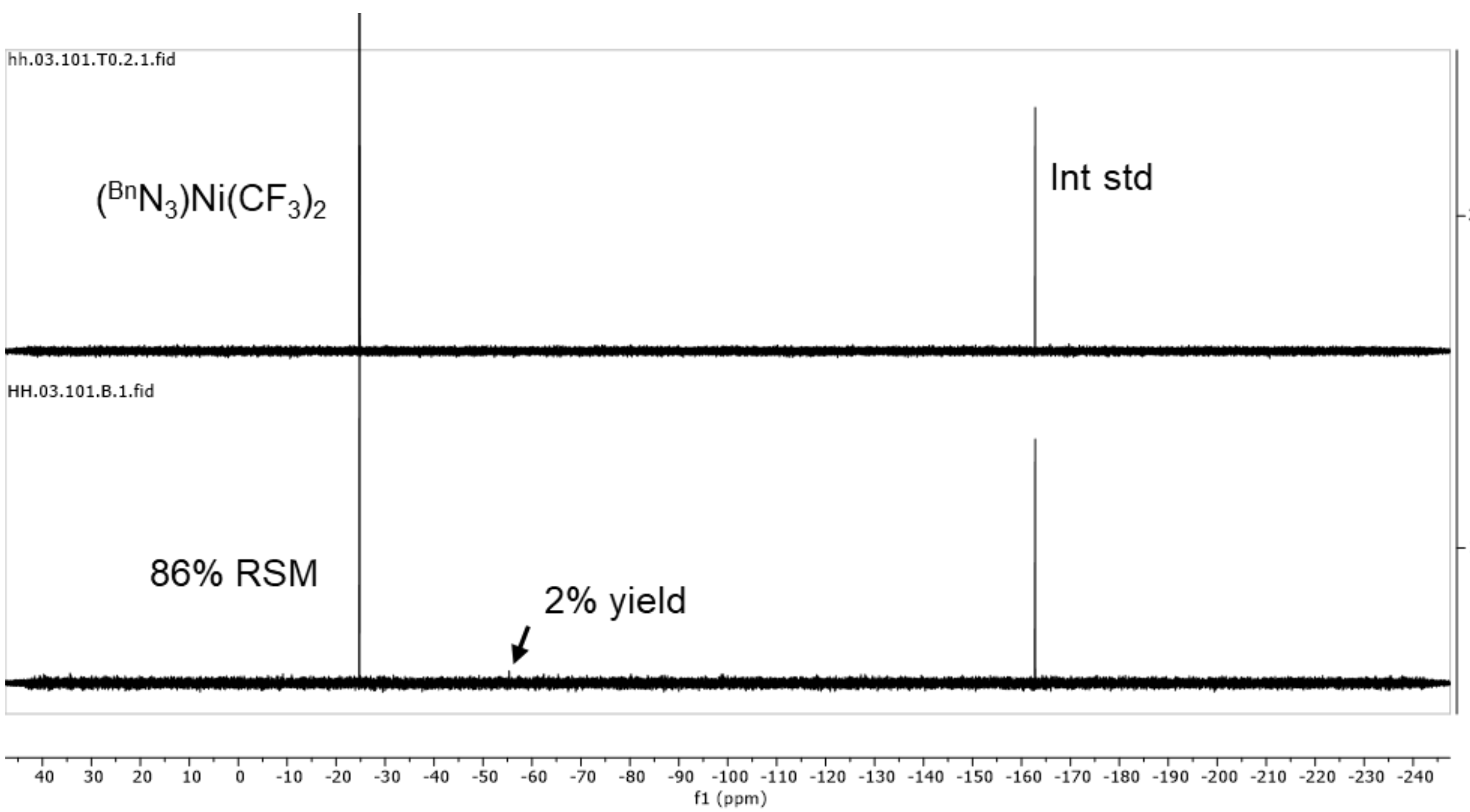


**Figure S24**: **($^{Bn}N_3$)Ni($CF_3$)$_2$** $^{19}$F NMR of reaction mixture at T = 0 (top) and T = 12 h (bottom), showing 86% RSM and 2% yield of TEMPO-$CF_3$ adduct after 405 nm irradiation for 12 h in benzene, using hexafluorobenzene as an internal standard (471 MHz, $C_6D_6$).

iii. 365 nm *hv* reaction.

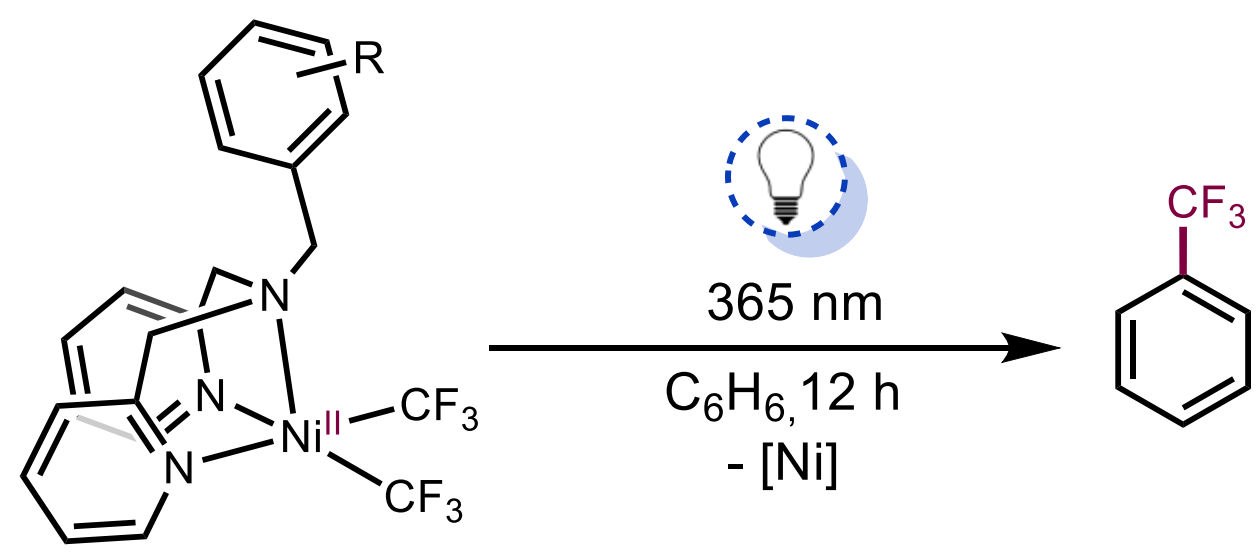


As an additional control experiment, given the stronger absorption of **($^{Bn}N_3$)Ni($CF_3$)$_2$** at 365 nm than 405 nm, the complex was irradiated at this wavelength. To a 20 mL scintillation vial was added 4 mL of a 0.032 mM solution of **($^{Bn}N_3$)Ni($CF_3$)$_2$** in benzene containing hexafluorobenzene as an internal standard. A small aliquot of the homogeneous solution was analyzed by $^{19}$F NMR after the addition of 5 drops of benzene-d6 (for NMR lock protocol). A stir bar was added to the reaction vial, the vial was capped and rigorously sealed with electrical tape before being removed from the glovebox and placed in the Hepatochem Lucent 360 photoreactor. The photoreactor was equipped with four 405 nm bulbs, with intensity set to 20%. A temperature of 35°C was maintained throughout the reaction with the use of an external Julabo chiller connected to the photoreactor water bath. The vials were irradiated with stirring for 12 hours before being brought back into the glovebox. An aliquot of the homogeneous reaction mixture was analyzed by $^{19}$F NMR after the addition of 5 drops of benzene-d6 (for NMR lock protocol).

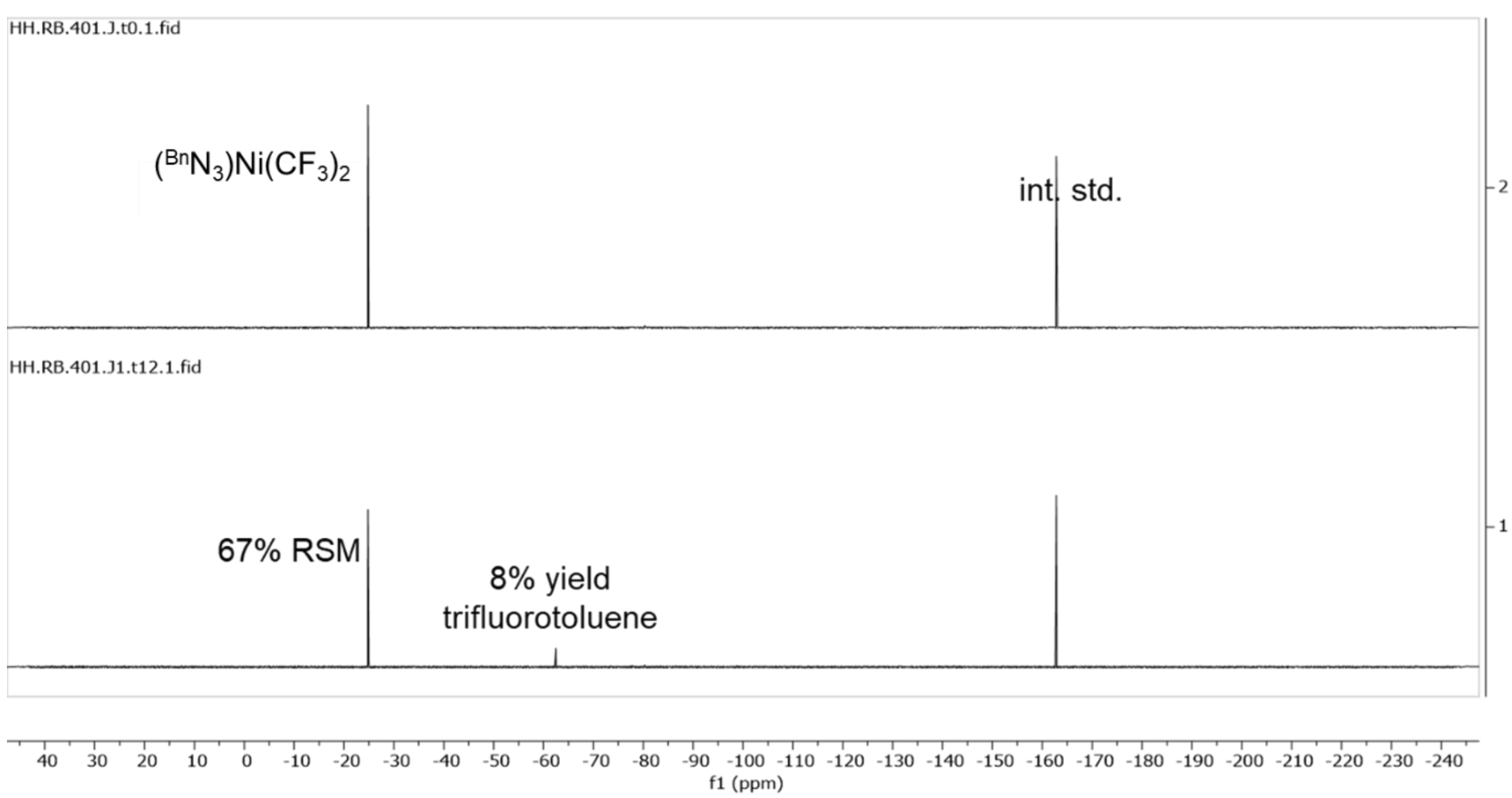

**Figure S25**: **($^{Bn}N_3$)Ni($CF_3$)$_2$** $^{19}$F NMR of reaction mixture at T = 0 (top) and T = 12 h (bottom), showing 8% yield of trifluorotoluene after 365 nm irradiation for 12 h in benzene, using hexafluorobenzene as an internal standard (471 MHz, $C_6D_6$).

iv. Intermolecular experiments (addition of exogeneous fluorenone)

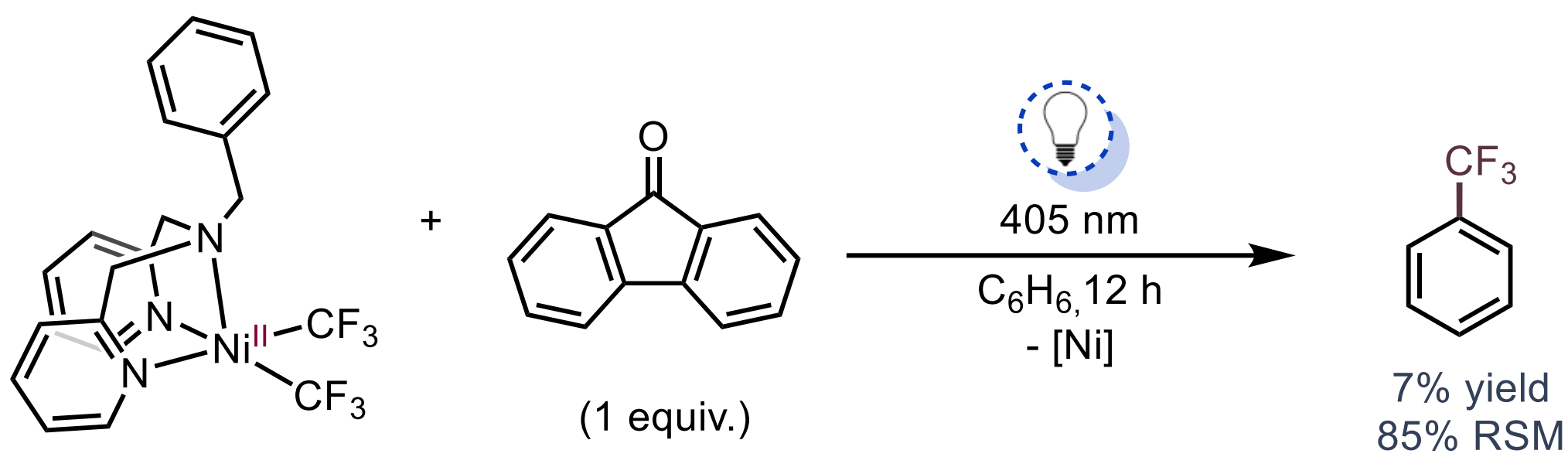


To a 20 mL scintillation vial was added 4 mL of a 0.032 mM solution of **($^{Bn}N_3$)Ni($CF_3$)$_2$** in benzene containing hexafluorobenzene as an internal standard. 1 equivalent of fluorenone (0.000128 mmol) were added via the addition of 0.05 mL of a 0.0256 M stock solution. A small aliquot of the homogeneous solution was analyzed by $^{19}$F NMR after the addition of 5 drops of benzene-d6 (for NMR lock protocol). A stir bar was added to the reaction vial, the vial was capped and rigorously sealed with electrical tape before being removed from the glovebox and placed in the Hepatochem Lucent 360 photoreactor. The photoreactor was equipped with four 405 nm bulbs, with intensity set to 20%. A temperature of 35°C was maintained throughout the reaction with the use of an external Julabo chiller connected to the photoreactor water bath. The vials were irradiated with stirring for 12 hours before being brought back into the glovebox. An aliquot of the homogeneous reaction mixture was analyzed by $^{19}$F NMR after the addition of 5 drops of benzene-d6 (for NMR lock protocol).

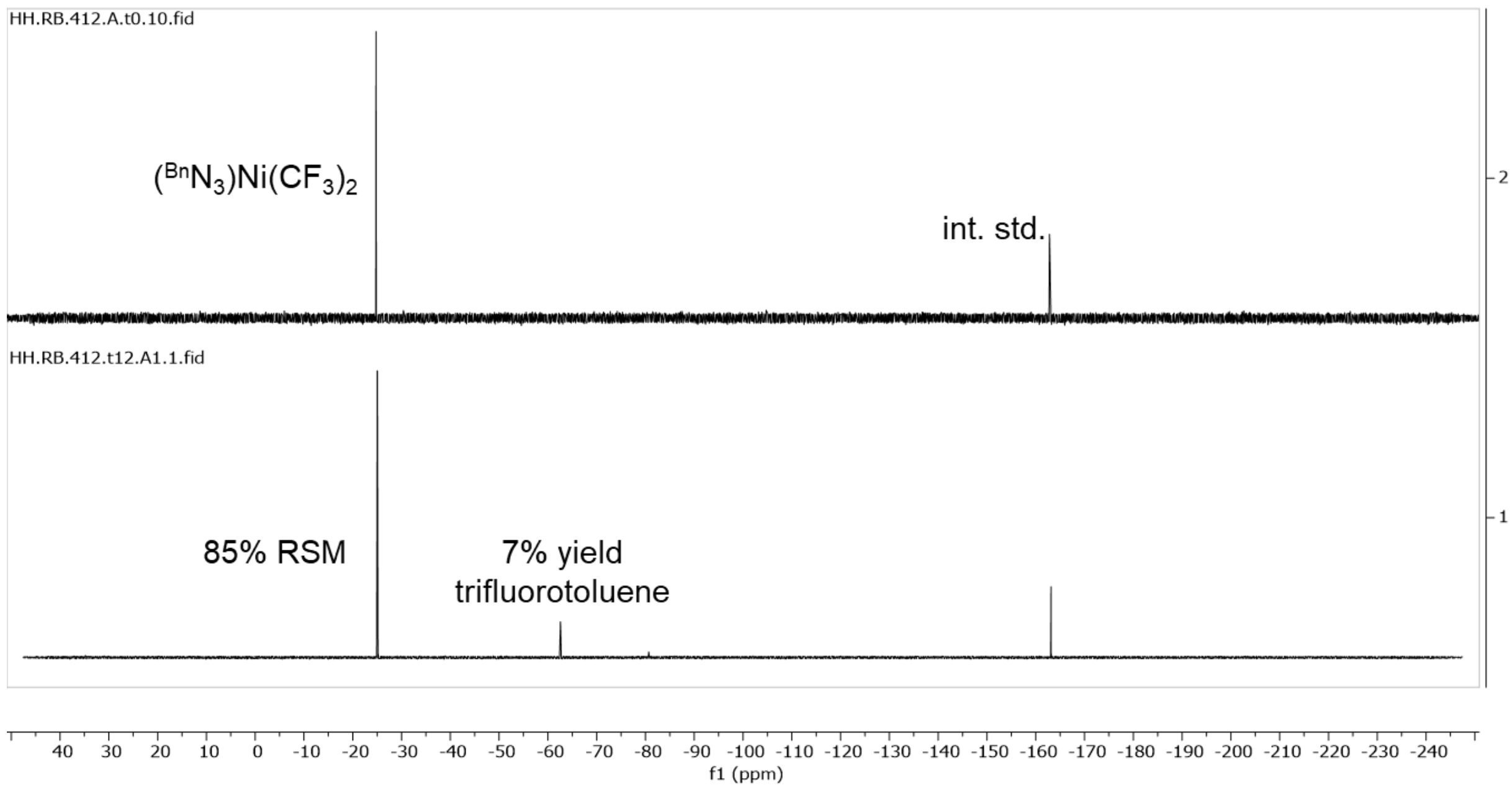


**Figure S26**: $(^{Bn}N_3)Ni(CF_3)_2$ $^{19}F$ NMR of reaction mixture at T = 0 (top) and T = 12 h (bottom), showing 85% RSM and 7% yield of trifluorotoluene after 405 nm irradiation for 12 h in benzene, using hexafluorobenzene as an internal standard (471 MHz, $C_6D_6$).

## IX. Stern-Volmer Quenching Studies.

Steady state Stern–Volmer luminescence quenching experiments were conducted using a Photon Technology International (PTI) QuantaMaster 400 steady state spectrofluorometer with the PTI FelixGX software. Quenching experiments were performed with freshly prepared and degassed 0.15 mM solutions of fluorenone in acetonitrile in 1 cm quartz cuvettes at room temperature in a nitrogen-filled glovebox. The quencher was added via a stock solution in acetonitrile, such that the concentration of fluorenone was a constant 0.15 mM. The solutions were irradiated at 370 nm, and luminescence was measured at the $\lambda_{max}$ for fluorenone (485 nm). The quencher **$(^{Bn}N_3)Ni(CF_3)_2$** is not emissive. The luminescence intensity (I) was measured for each sample, and the experiments were repeated in triplicate. The $I_0/I$ (a ratio of the initial and subsequent intensities in the presence of quencher [Q]) value of each quencher concentration were generated from the average luminescence intensity of all three measurements. Linear regression of $I_0/I$ as a function of quencher concentrate ion to yield $k_{sv}$, as described by the Stern-Volmer relationship (Equation S1), was performed using Microsoft Excel. Error bars were calculated using propagation of error from standard deviation of triplicate intensity measurements.[8]

(Equation S1):
$$I_0/I = 1 + k_{SV}[Q]$$

Table S1: Quenching data for fluorenone with **$(^{Bn}N_3)Ni(CF_3)_2$** (**[Ni]**), monitored at $\lambda$ = 485 nm.

| **Run** | **[Ni] (mM)** | **Intensity (I)** | | | | **$I_0$ / I** |
|---|---|---|---|---|---|---|
| | | **Trial 1** | **Trial 2** | **Trial 3** | **Average** | |
| A | 0 | 453700 | 445300 | 430200 | 443066.667 | 1 |
| B | 0.0435 | 440800 | 439900 | 428700 | 436466.667 | 1.015 |
| C | 0.0957 | 437300 | 447100 | 412600 | 432333.333 | 1.025 |
| D | 0.1305 | 457800 | 449800 | 426700 | 444766.667 | 0.9962 |
| E | 0.1697 | 467200 | 465800 | 428600 | 453866.667 | 0.9762 |

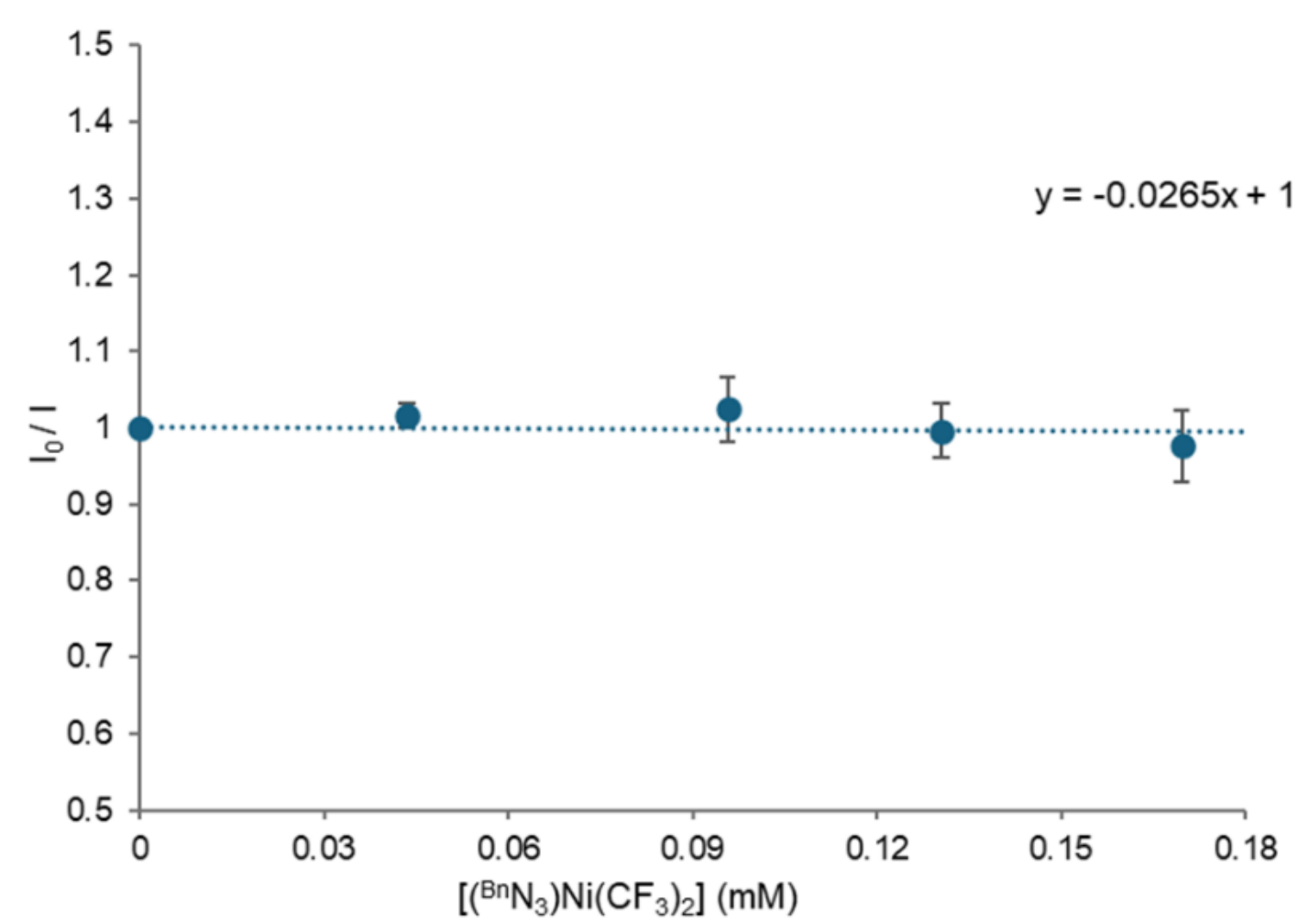


**Figure S27**: Steady-state Stern-Volmer relationship for fluorenone and **$(^{Bn}N_3)Ni(CF_3)_2$**.

## X. Transient Absorption Spectroscopy.

All samples were prepared in an MBraun inert atmosphere (nitrogen) dry glovebox. The solvents used for sample preparation were dried and degassed using literature procedures.[1] Samples were prepared in a specially designed 2 mm quartz cuvette that was sealed for the duration of the transient absorption study. Samples were prepared in a 1:1 v/v mixture of benzene:1,2-difluorobenzene to aid in solubility of the metal complex for optimal signal intensity. The solutions were continually stirred during the experiment to minimize sample degradation. Each sample was excited with a pulse centered at 400 nm (power of 400 nJ) or 430 nm (power of 750 nJ) (Figure S28, S29) and probed using a white-light supercontinuum white light spectrum generated in a continuously vertically translating calcium fluoride crystal. The first and last scan of each data set were overlapped and showed no decay in the signal for the chemically active species. The transient absorption system consists of a Coherent Astrella ultrafast amplifier system (2x 2.5W, 1kHz, 45 fs, 800 nm) one output seeds a Coherent OperA Solo OPA (optical parametric amplifier) to generate the excitation pulses. The second output split 90/10 where the 10% reflection is directed into the Helios (Ultrafast Systems), a commercially designed transient absorption spectrometer.

Due to the complex dynamics of the system, global analysis was used to analyze the transient absorption data. To determine the number of distinguishable components in the TA dynamics, we applied a sequential model to fit the time-wavelength matrix with first order kinetics. Three components best described the dynamics of free fluorenone, while four components best described the dynamics of **($^{Fluor}N_3$)Ni($CF_3$)$_2$**. Global analysis was performed using Glotaran and the R-package (TIMP). All models included an instrument response function (fit with a Gaussian profile), chirp correction, coherent artifact, and a sequential, irreversible rate matrix [A → B → C and so forth] with increasing lifetimes.[9-14] This picture allows us to clearly visualize the evolution of the excited states within the systems.

The three-component model is analogous to the excited-state evolution of free fluorenone after photoexcitation at 400 nm shown in Equation 2.

Equation 2: $$S_n \rightarrow S_1 \rightarrow T_1 \rightarrow S_0$$

whereas the four-component scheme is analogous to the evolution of excited-state components in **($^{Fluor}N_3$)Ni($CF_3$)$_2$** after photoexcitation at 430 nm shown in Equation 3.

Equation 3: $$hot\ {}^1MLCT \rightarrow {}^1MLCT \rightarrow {}^3MLCT \rightarrow {}^3d-d \rightarrow S_0$$

Figure S31 shows the resulting EADS traces of free fluorenone excited at 400 nm. Figure S38 shows the resulting EADS traces of **($^{Fluor}N_3$)Ni($CF_3$)$_2$** excited at 430 nm. Figures S32 and S39 show the goodness of the global analysis fits to the transient absorption data (for free fluorenone and **($^{Fluor}N_3$)Ni($CF_3$)$_2$**, respectively) , with fit residuals and root-mean-square error (RMSE) given.

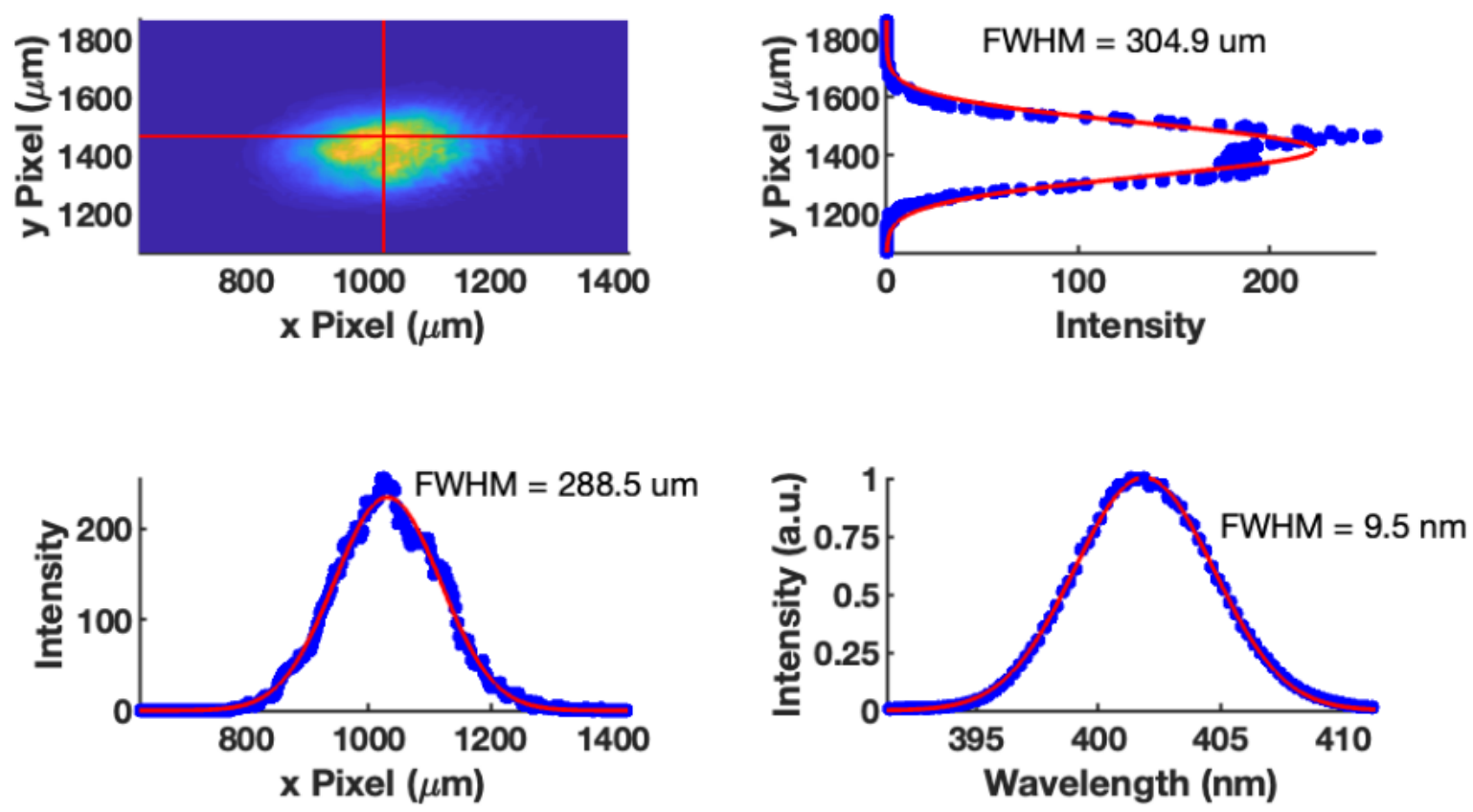


**Figure S28**: Beam profile of excitation pulse (400 nm) at sample position.

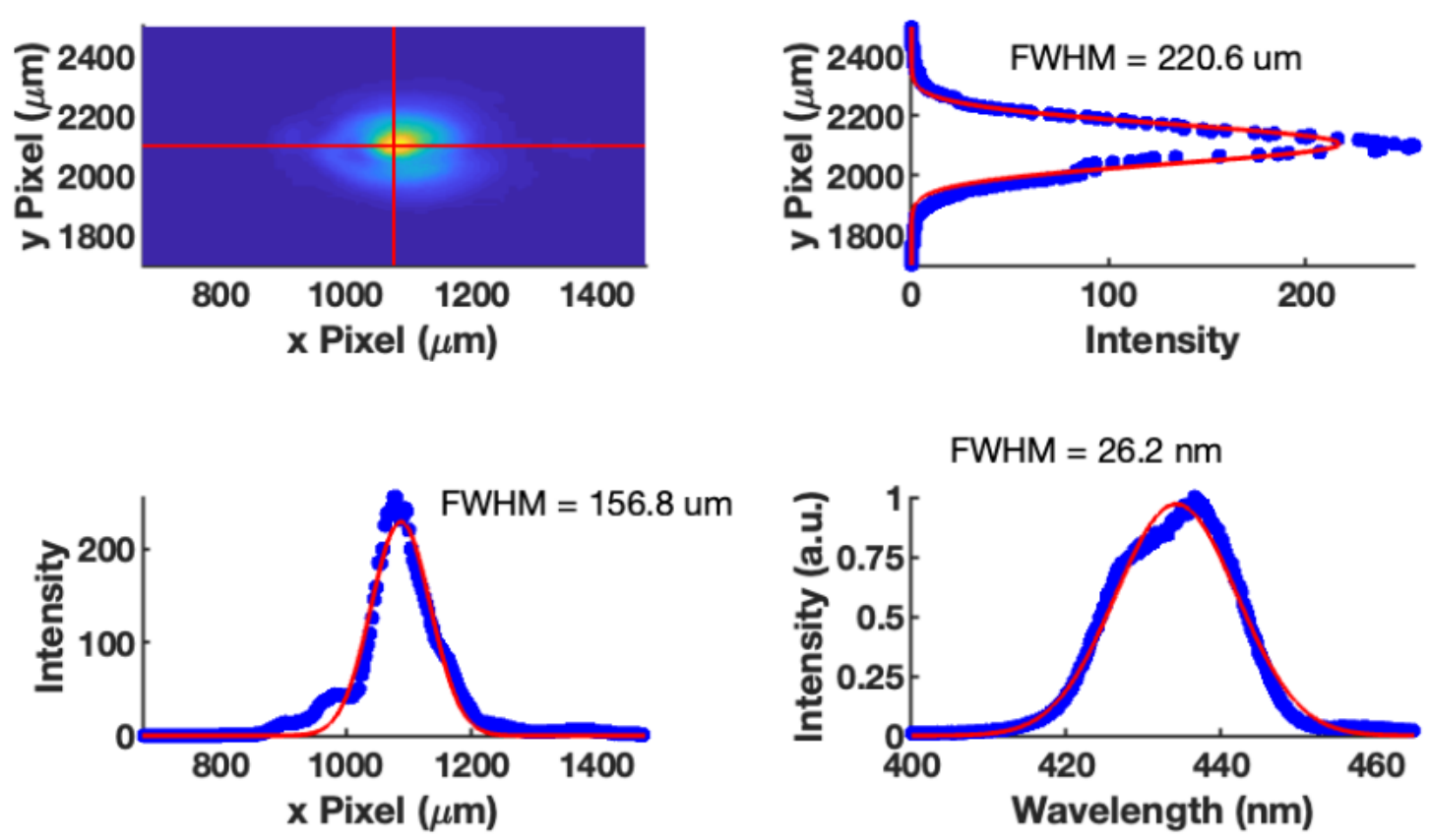


**Figure S29**: Beam profile of excitation pulse (430 nm) at sample position.

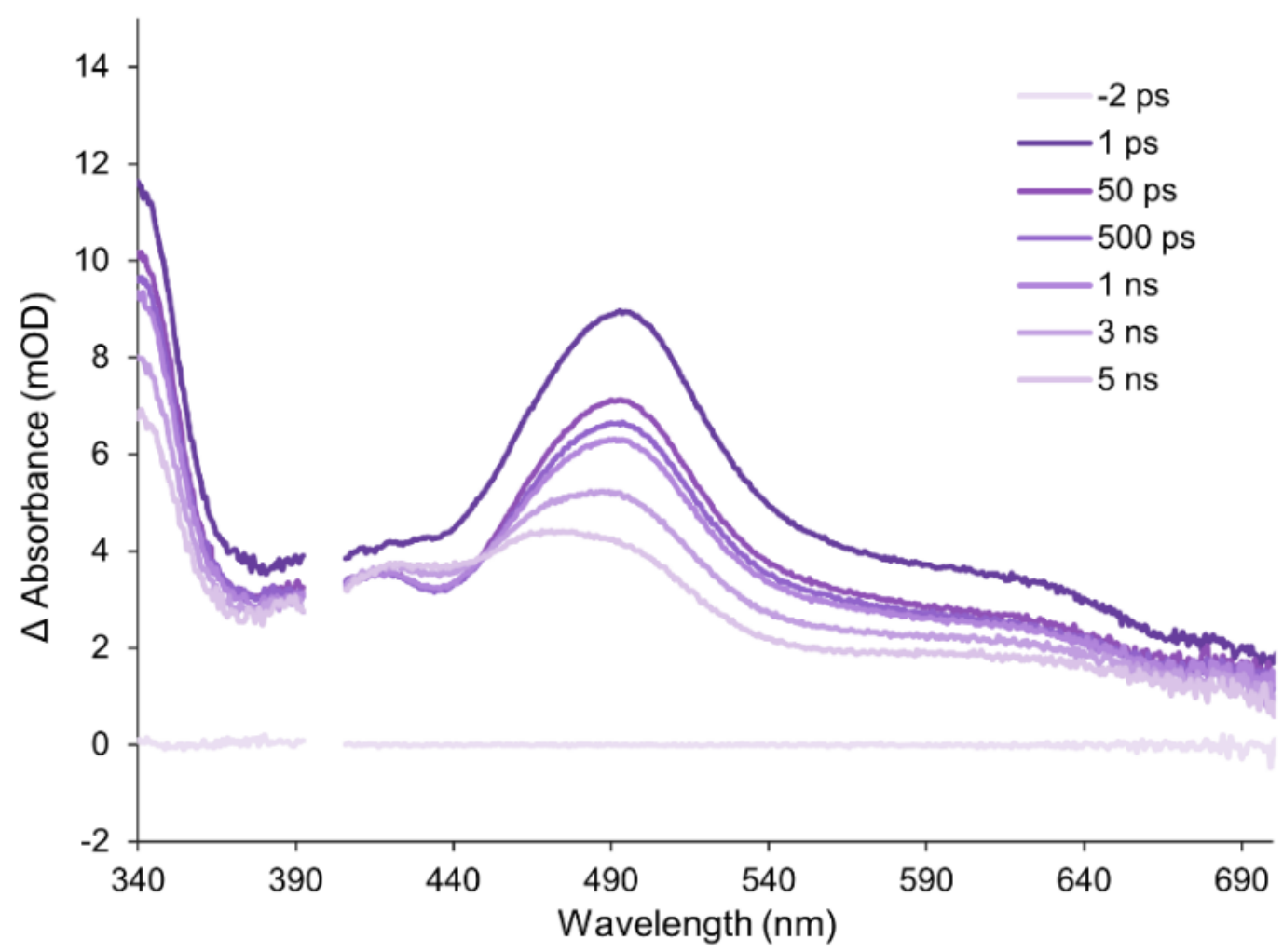


**Figure S30**: Transient absorption spectra of free fluorenone with a 400 nm pump excitation.

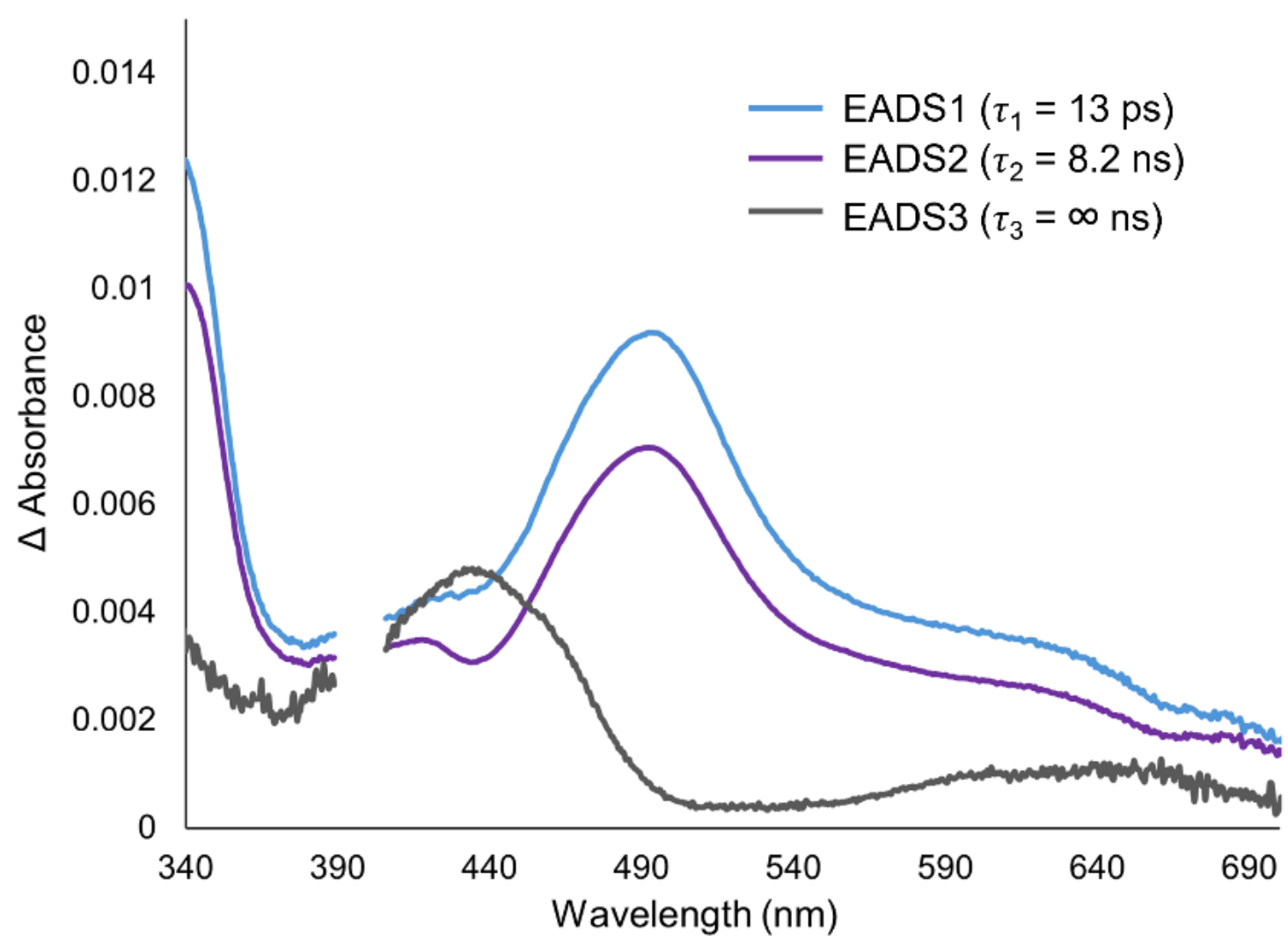


**Figure S31**: EADS of free fluorenone from transient absorption spectra at 400 nm pump excitation.

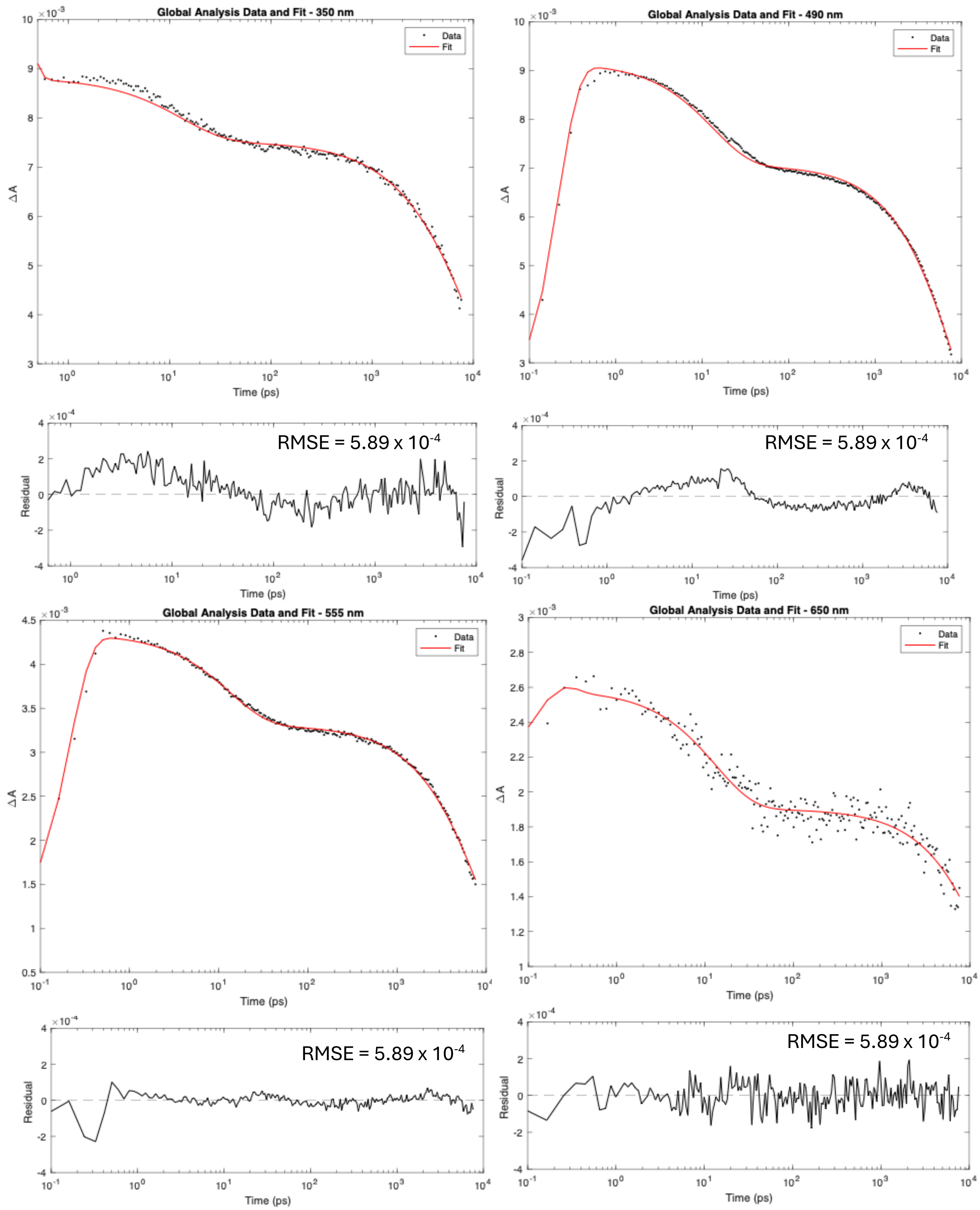


**Figure S32**: Data and fits with residuals resulting from a global analysis of the free fluorenone transient absorption data excited at 400 nm using a three-component kinetic scheme plotted for select wavelengths at 350, 490, 555, and 650 nm.

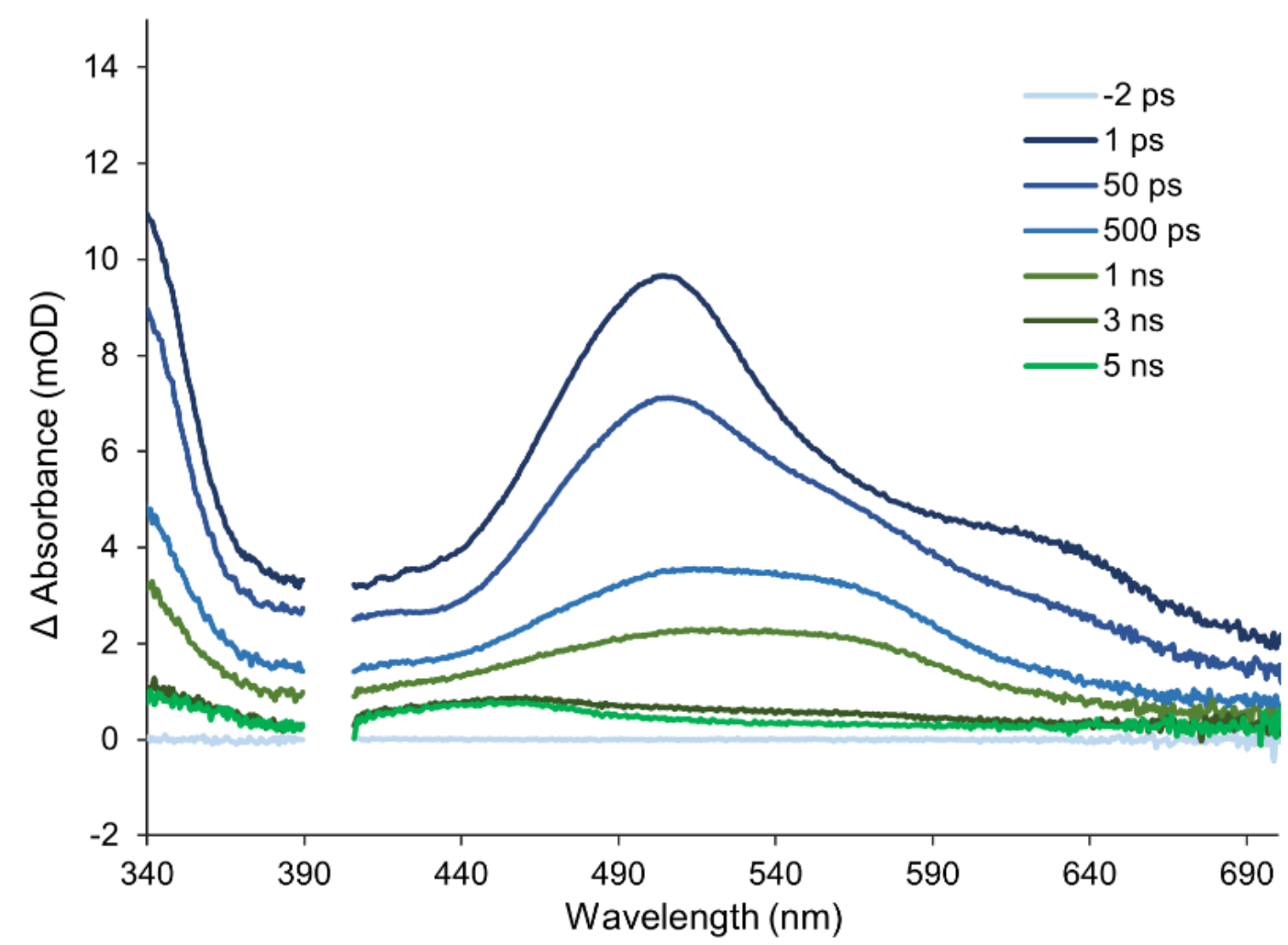


**Figure S33**: Transient absorption spectra of $^{Fluor}$**N$_3$** with a 400 nm pump excitation.

After verifying that the spectral features observed for the free ligand (Figure S33) are absent from the spectral features observed for the metal complex ($^{Fluor}N_3$)Ni($CF_3$)$_2$, we focused our efforts on analysis of the ($^{Fluor}N_3$)Ni($CF_3$)$_2$ data (Figure S37). While the evolution of the spectral features of the free ligand $^{Fluor}$N3 was not deconvoluted using global analysis, we hypothesize that the spectral decay may involve the lone pair of the pendant nitrogen atom. In the metal complex, this lone pair is involved in bonding with the metal center, so these spectral features are not observed.

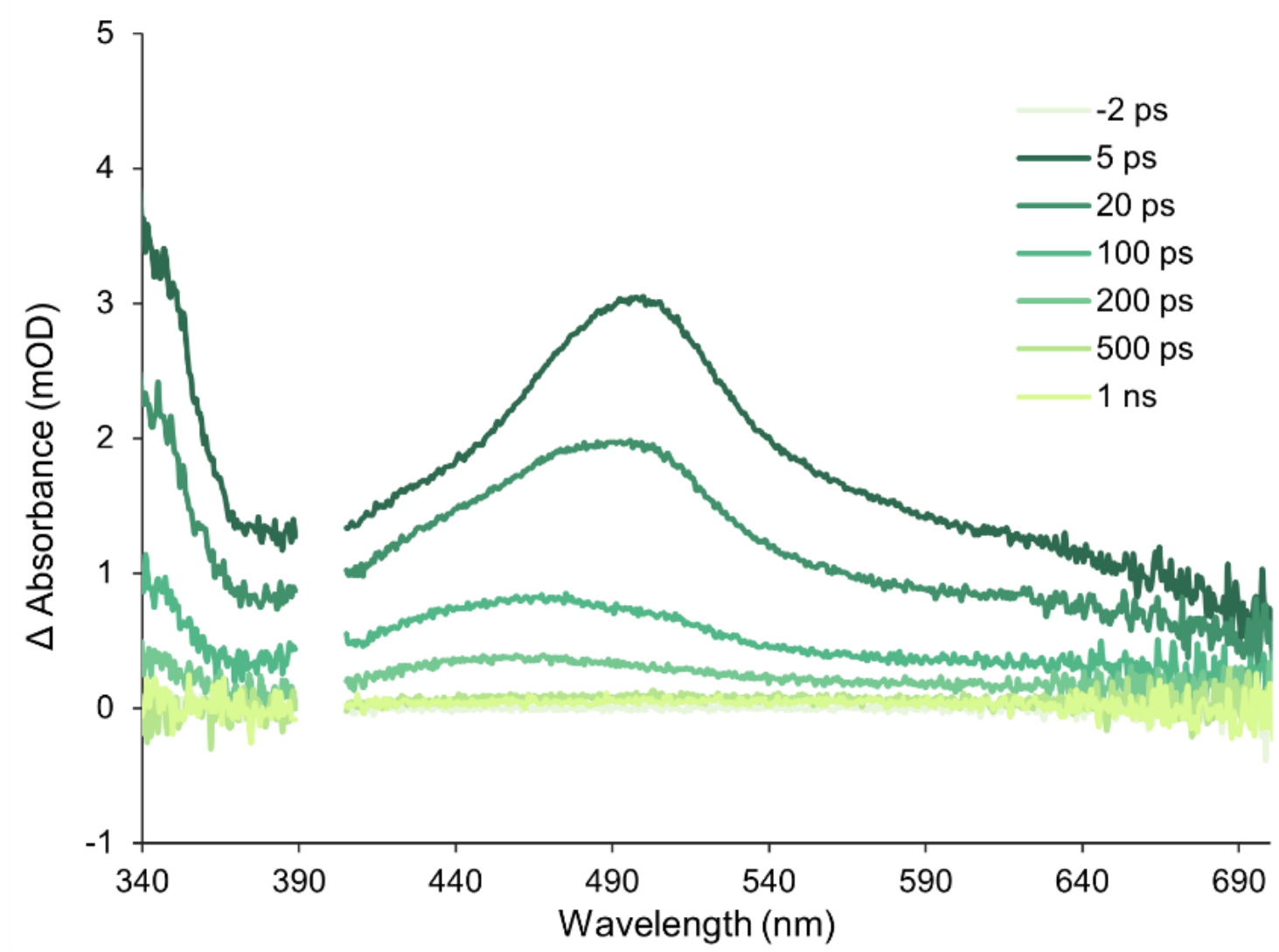


**Figure S34**: Transient absorption spectra of (**$^{Fluor}N_3$)Ni($CF_3$)$_2$** with a 400 nm pump excitation.

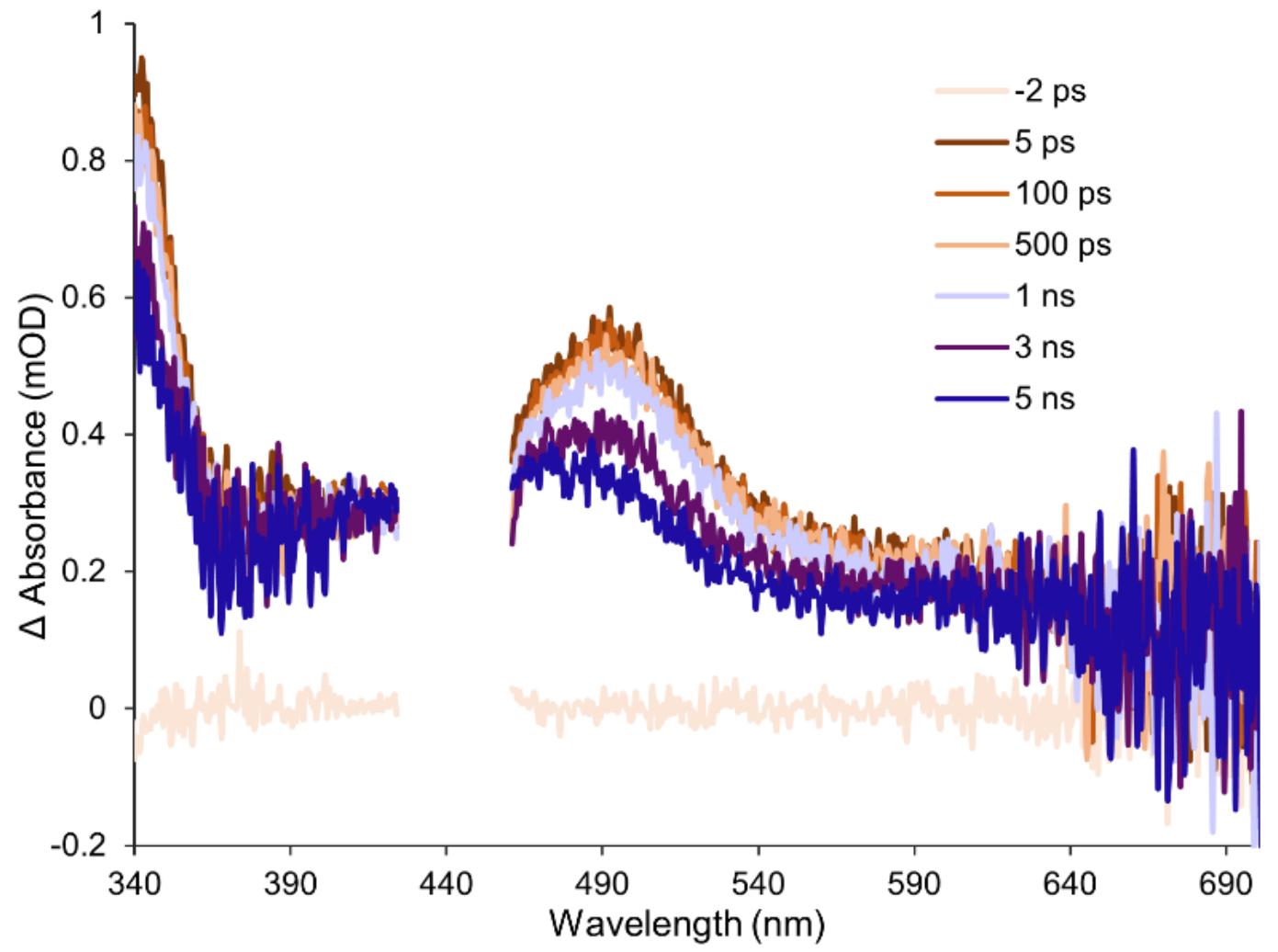


**Figure S35**: Transient absorption spectra of free fluorenone with a 430 nm pump excitation.

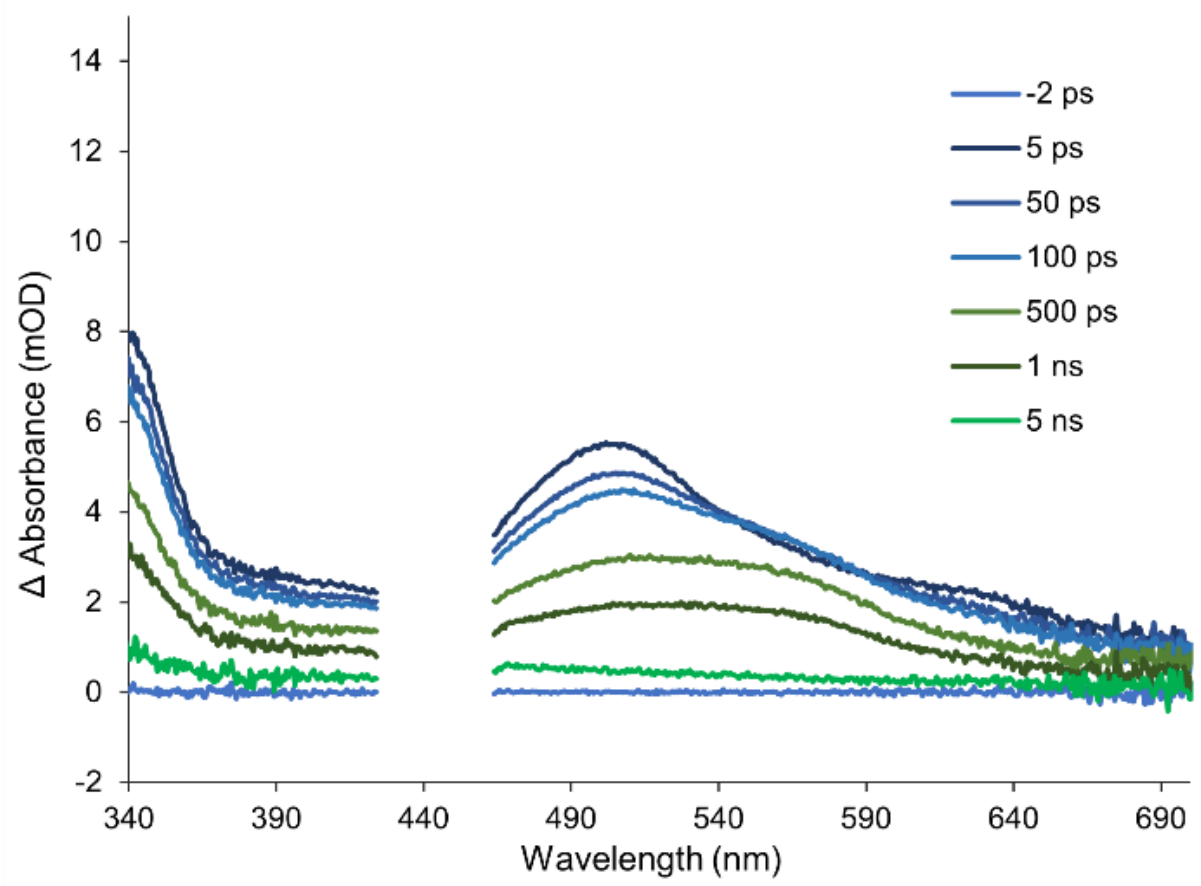


**Figure S36**: Transient absorption spectra of $^{\mathbf{Fluor}}\mathbf{N_3}$ with a 430 nm pump excitation.

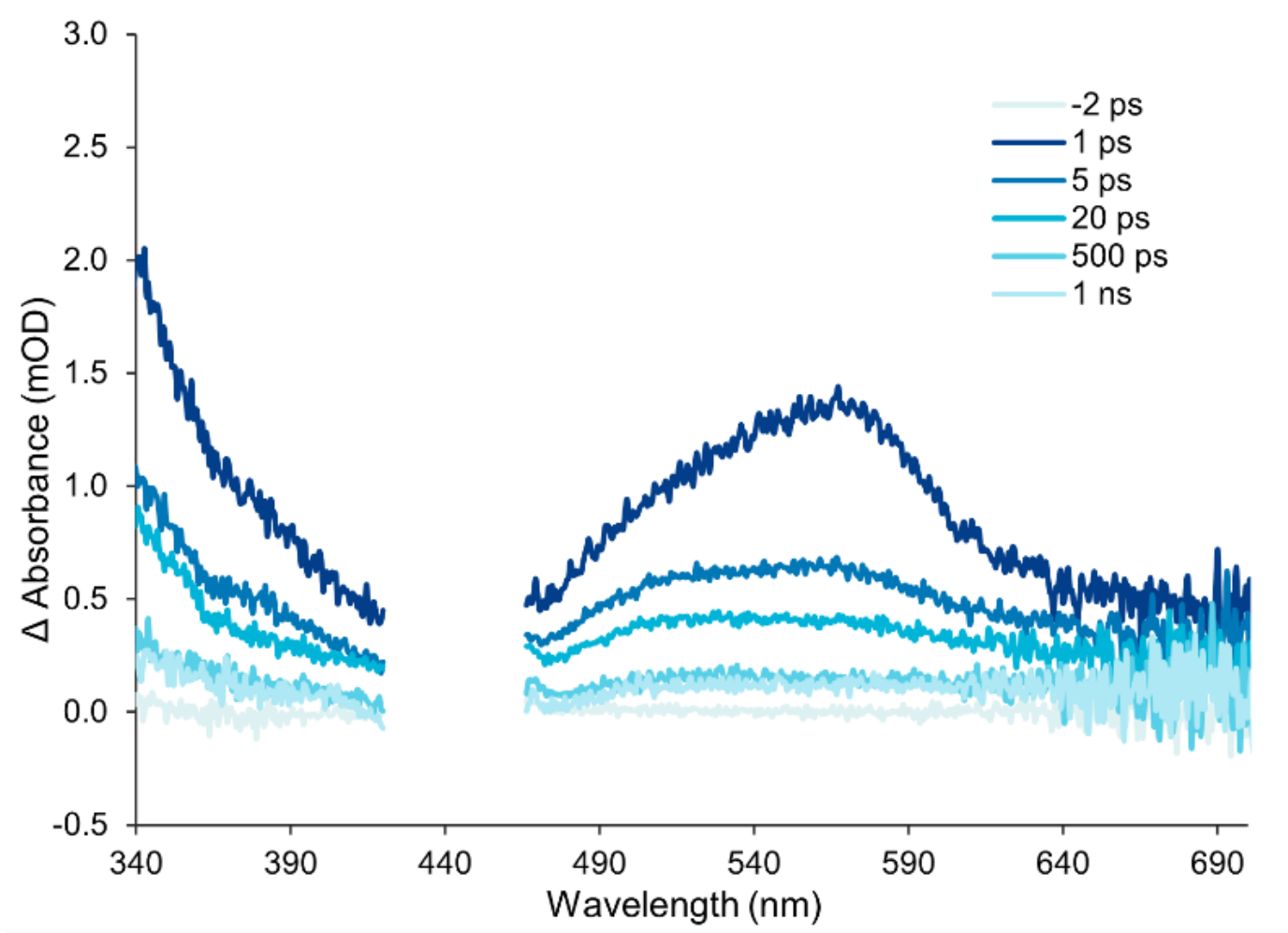


**Figure S37**: Transient absorption spectra of ($^{Fluor}N_3$)$Ni(CF_3)_2$ with a 430 nm pump excitation.

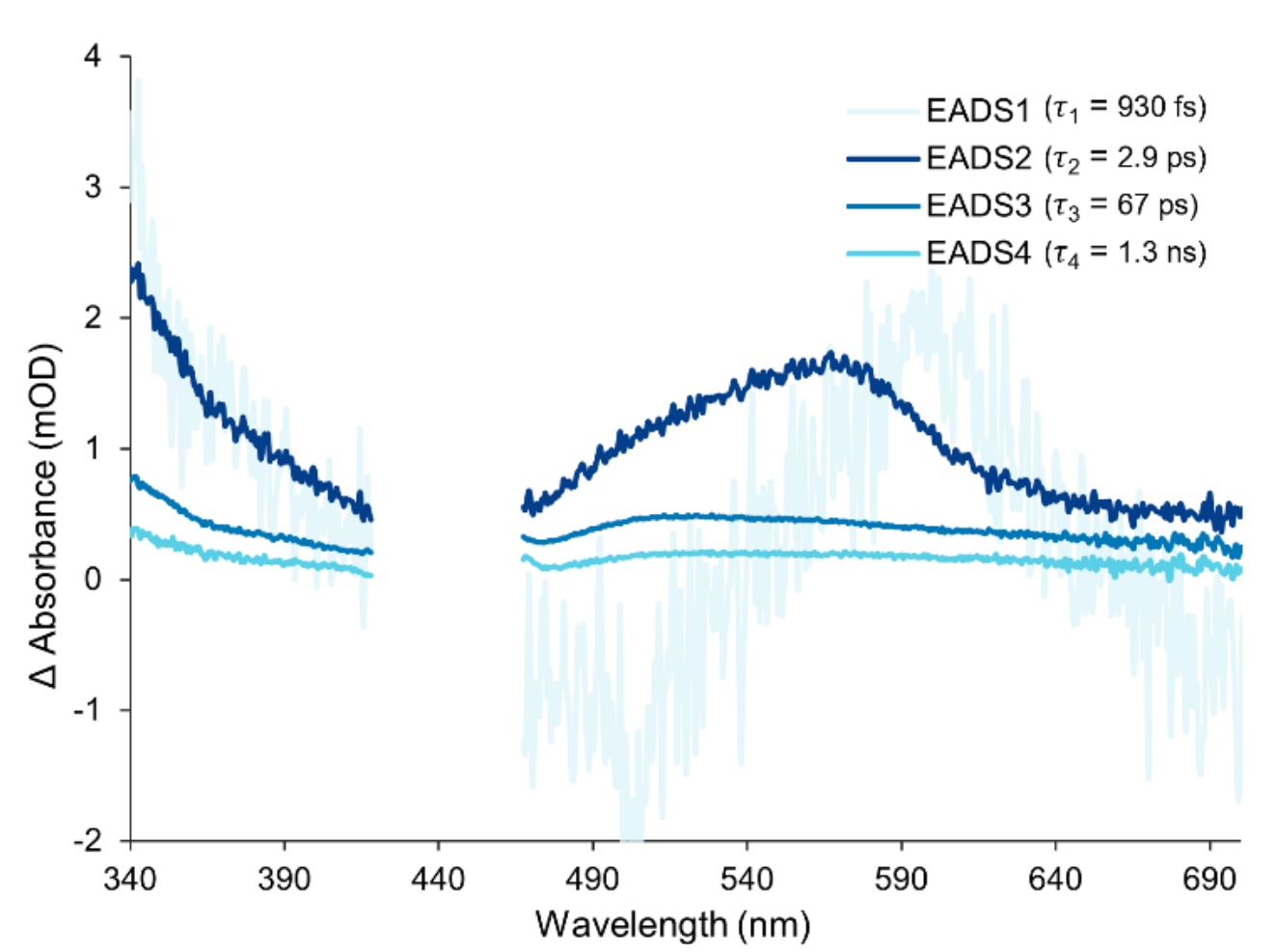


**Figure S38**: EADS of ($^{Fluor}N_3$)$Ni(CF_3)_2$ from transient absorption spectra at 430 nm pump excitation.

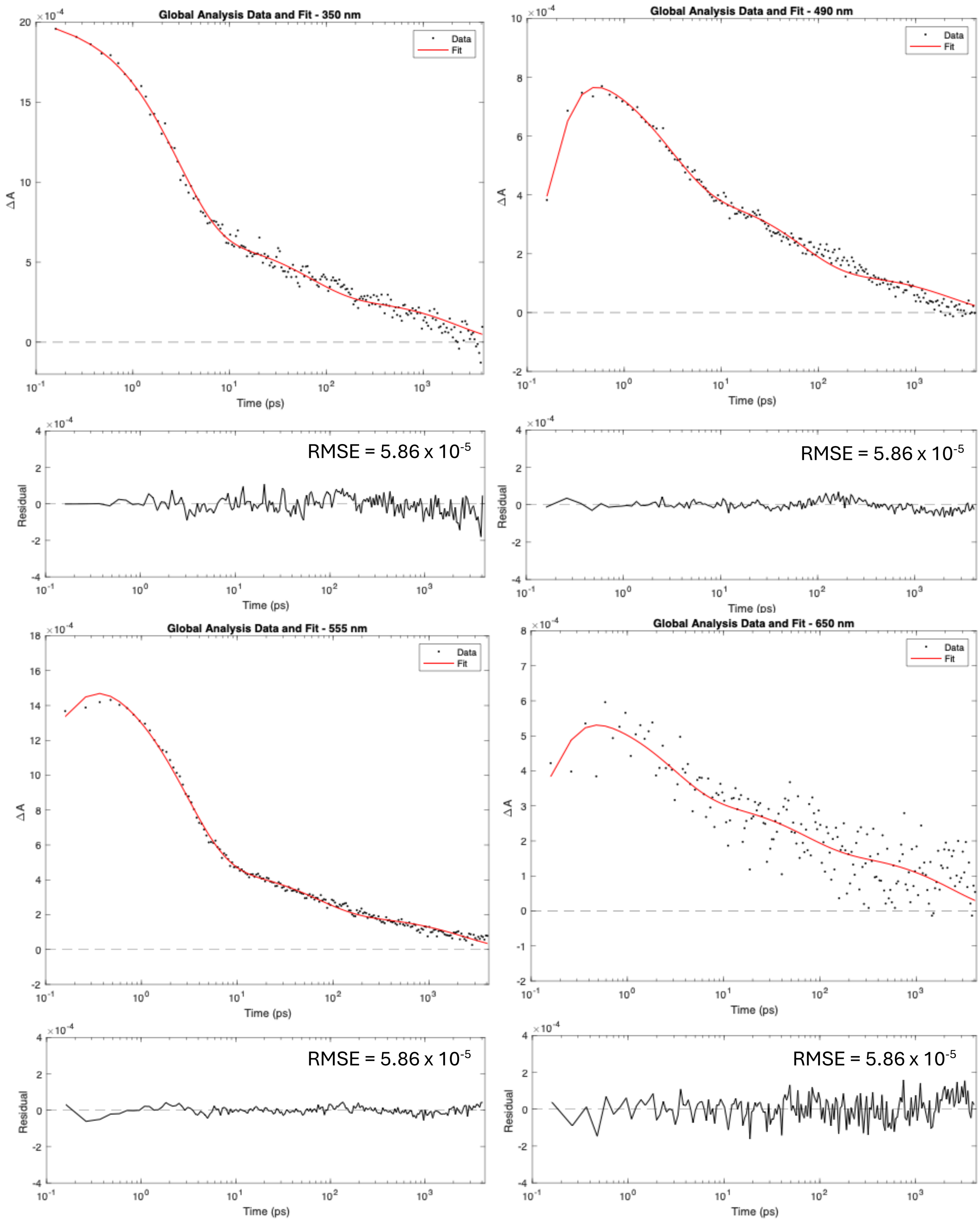


**Figure S39**: Data and fits with residuals resulting from a global analysis of the **$(^{Fluor}N_3)Ni(CF_3)_2$** transient absorption data excited at 430 nm using a four-component kinetic scheme plotted for select wavelengths at 350, 490, 555, and 650 nm.

## XI. Computational Calculations.

Density functional theory (DFT) calculations were performed with the Gaussian16 software package.[15] All geometries were optimized using the M06 functional with the tzvp basis set.[16,17] Vibrational frequencies were computed at the same level of theory to characterize optimized geometries as global minima (zero imaginary frequencies). Time-dependent DFT calculations (TD-DFT) were carried out with the M06 functional with the def2tzvp basis set. Three-dimensional structures of molecular orbitals were rendered using USCF Chimera X.[18] Solvent effects were considered through the self-consistent reaction field (SCRF) model.[19]

### i. $(^{Fluor}N_3)Ni(CF_3)_2$

**Geometry optimization:**
# opt freq m06 tzvp scrf=(solvent=benzene)

**Output of ground state geometry optimization:**

Zero-point correction= 0.439285 (Hartree/Particle)
Thermal correction to Energy= 0.472576
Thermal correction to Enthalpy= 0.473520
Thermal correction to Gibbs Free Energy= 0.371624
Sum of electronic and zero-point Energies= -3425.590995
Sum of electronic and thermal Energies= -3425.557704
Sum of electronic and thermal Enthalpies= -3425.556759
Sum of electronic and thermal Free Energies= -3425.658656

**Cartesian coordinates of optimized ground state geometry:**

| | | | |
|---|---|---|---|
| Ni | -2.767966 | 0.478922 | -0.277985 |
| F | -1.913419 | 2.967270 | 0.459058 |
| F | -3.846067 | 2.474038 | 1.283035 |
| F | -3.695432 | 3.133234 | -0.740654 |
| F | -2.221093 | 1.968745 | -2.588713 |
| F | -4.060988 | 0.847444 | -2.703390 |
| F | -2.157629 | -0.148139 | -2.875941 |
| O | 5.041046 | -2.181562 | -1.608130 |
| N | -2.629607 | 0.002781 | 1.647317 |
| N | -2.723281 | -1.492341 | -0.587539 |
| N | -0.347578 | -0.076794 | -0.053730 |
| C | -3.089520 | 2.324997 | 0.150629 |
| C | -2.814778 | 0.828714 | -2.162395 |
| C | -3.726534 | -0.273678 | 2.354460 |
| H | -4.670686 | -0.013911 | 1.888849 |
| C | -3.677113 | -0.845060 | 3.609824 |
| H | -4.593771 | -1.051123 | 4.146259 |
| C | -2.438821 | -1.147817 | 4.147765 |
| H | -2.359466 | -1.604052 | 5.127284 |
| C | -1.302048 | -0.857201 | 3.416778 |
| H | -0.314710 | -1.077235 | 3.804993 |
| C | -1.427094 | -0.276608 | 2.164522 |
| C | -0.217575 | 0.132526 | 1.373956 |
| H | -0.079869 | 1.207590 | 1.538742 |
| H | 0.672366 | -0.368181 | 1.791294 |

| | | | |
|---|---|---|---|
| C | -0.277419 | -1.462668 | -0.462867 |
| H | 0.449689 | -2.040599 | 0.132101 |
| H | 0.080783 | -1.496128 | -1.497574 |
| C | -1.596472 | -2.195663 | -0.468465 |
| C | -1.616510 | -3.583323 | -0.447304 |
| H | -0.685149 | -4.127321 | -0.338268 |
| C | -2.820107 | -4.247522 | -0.573880 |
| H | -2.852754 | -5.330451 | -0.561262 |
| C | -3.982271 | -3.508732 | -0.723725 |
| H | -4.946876 | -3.985415 | -0.836751 |
| C | -3.888145 | -2.133137 | -0.718425 |
| H | -4.766400 | -1.504989 | -0.827784 |
| C | 0.454398 | 0.858419 | -0.850993 |
| H | 0.046337 | 1.859562 | -0.683393 |
| H | 0.275026 | 0.624294 | -1.903660 |
| C | 1.929286 | 0.848245 | -0.542853 |
| C | 2.490251 | 1.870143 | 0.218563 |
| H | 1.850310 | 2.686470 | 0.541298 |
| C | 3.839207 | 1.886742 | 0.564748 |
| H | 4.242654 | 2.703996 | 1.152646 |
| C | 4.637277 | 0.847465 | 0.137714 |
| C | 6.072393 | 0.573476 | 0.324891 |
| C | 7.053073 | 1.273128 | 0.996384 |
| H | 6.830382 | 2.201901 | 1.510127 |
| C | 8.347617 | 0.758210 | 0.999572 |
| H | 9.131449 | 1.295288 | 1.521483 |
| C | 8.654830 | -0.427454 | 0.347402 |
| H | 9.671427 | -0.801684 | 0.366902 |
| C | 7.665582 | -1.133576 | -0.329759 |
| H | 7.884648 | -2.061615 | -0.846692 |
| C | 6.387117 | -0.622160 | -0.331158 |
| C | 5.154292 | -1.164647 | -0.967823 |
| C | 4.084206 | -0.180849 | -0.632816 |
| C | 2.754379 | -0.191795 | -0.981040 |
| H | 2.367203 | -0.993624 | -1.603116 |

**Table S2:** Selected bond lengths for optimized ground state **($^{Fluor}N_3$)Ni($CF_3$)$_2$**

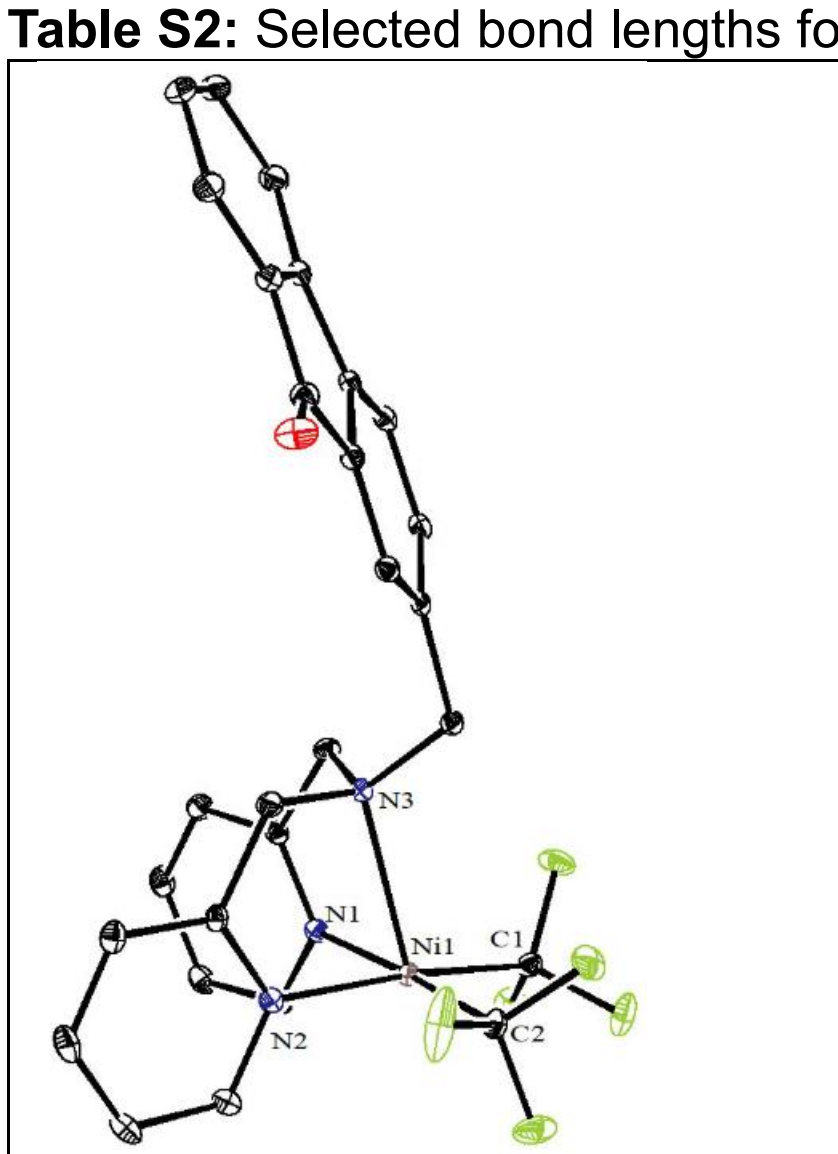


| Bond | Length (Å) |
|---|---|
| Ni-N1 | 1.98813 |
| Ni-N2 | 1.99592 |
| Ni-N3 | 2.49347 |
| Ni-C1 | 1.92226 |
| Ni-C2 | 1.91717 |

**Visualized molecular orbitals for optimized ground state of ($^{Fluor}N_3$)Ni($CF_3$)$_2$:**

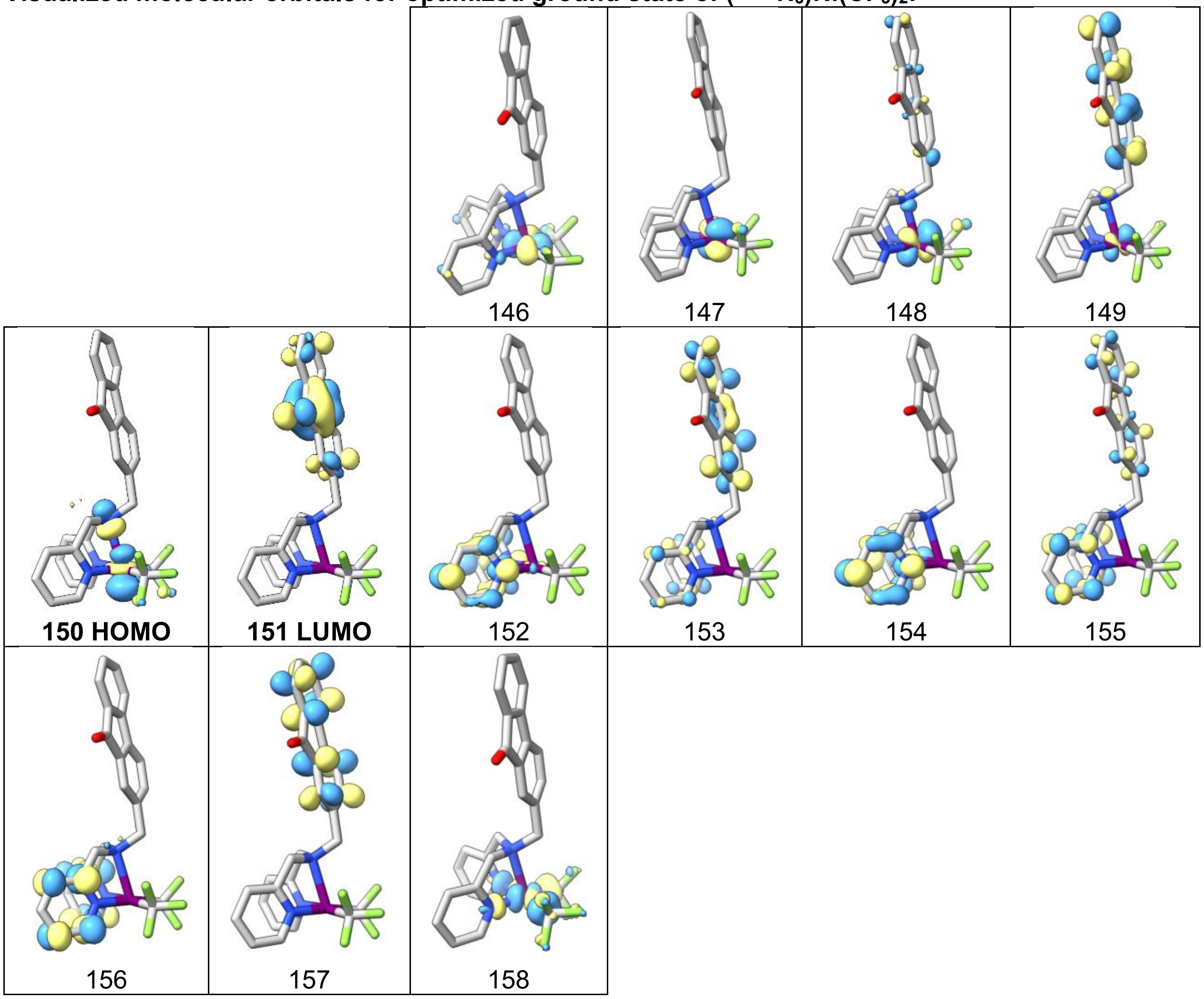


**Natural population analysis on optimized geometry:**

```
# freq scrf=(solvent=benzene) m06 def2tzvp Population=NPA Density=Current
```

**TD-DFT with natural population analysis on optimized geometry:**

```
# td(nstates=50) m06 def2tzvp scrf=(solvent=benzene) Pop=NPA Geom=AllCheck Guess=Read
```

**TD-DFT output: excited states with λ > 370 nm**

```
Excited State   1:      Singlet-A      2.1120 eV  587.04 nm  f=0.0009  <S**2>=0.000
Excited State   2:      Singlet-A      2.6330 eV  470.89 nm  f=0.0090  <S**2>=0.000
Excited State   3:      Singlet-A      2.6827 eV  462.16 nm  f=0.0028  <S**2>=0.000
Excited State   4:      Singlet-A      2.7657 eV  448.30 nm  f=0.0007  <S**2>=0.000
Excited State   5:      Singlet-A      2.8411 eV  436.40 nm  f=0.0004  <S**2>=0.000
Excited State   6:      Singlet-A      3.0126 eV  411.55 nm  f=0.0004  <S**2>=0.000
Excited State   7:      Singlet-A      3.1320 eV  395.86 nm  f=0.0066  <S**2>=0.000
Excited State   8:      Singlet-A      3.2112 eV  386.09 nm  f=0.0009  <S**2>=0.000
Excited State   9:      Singlet-A      3.2686 eV  379.32 nm  f=0.0074  <S**2>=0.000
Excited State  10:      Singlet-A      3.2697 eV  379.19 nm  f=0.0001  <S**2>=0.000
```

**Natural transition orbitals calculated using the following input:**

```
# ChkBasis Guess=(Only,Read) Pop=SaveNTO Geom=AllCheck Density=(Check, Transition=n)
```

**Natural transition orbitals (NTOs) of excited states 1-10:**

| Excited State | Hole | Particle |
|---|---|---|
| **1**<br>**587 nm**<br>**f = 0.0009** | 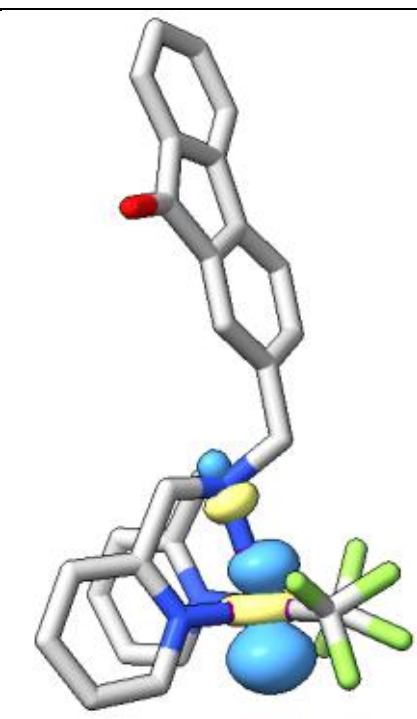 | 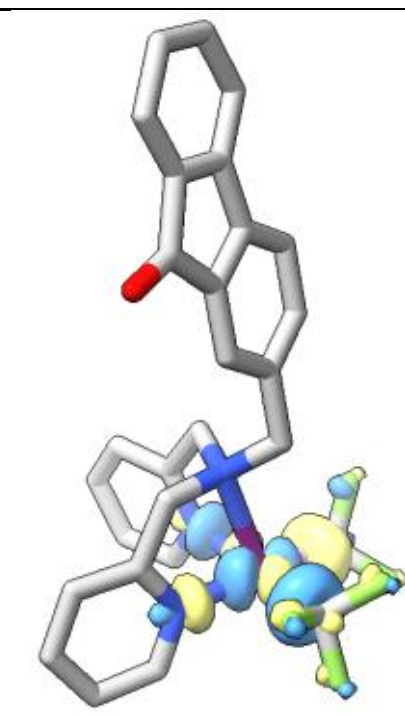 |
| **2**<br>**471 nm**<br>**f = 0.0090** | 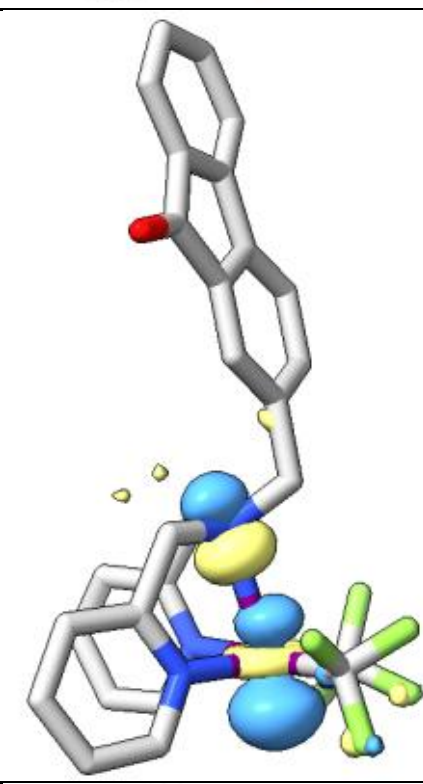 | 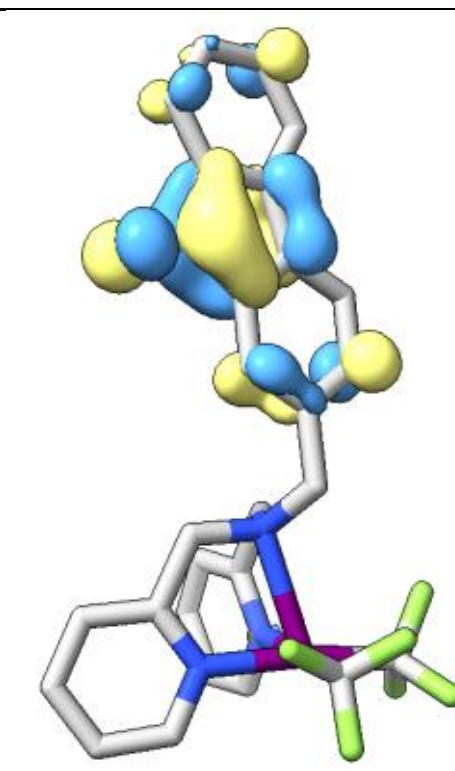 |
| **3**<br>**462 nm**<br>**f = 0.0028** | 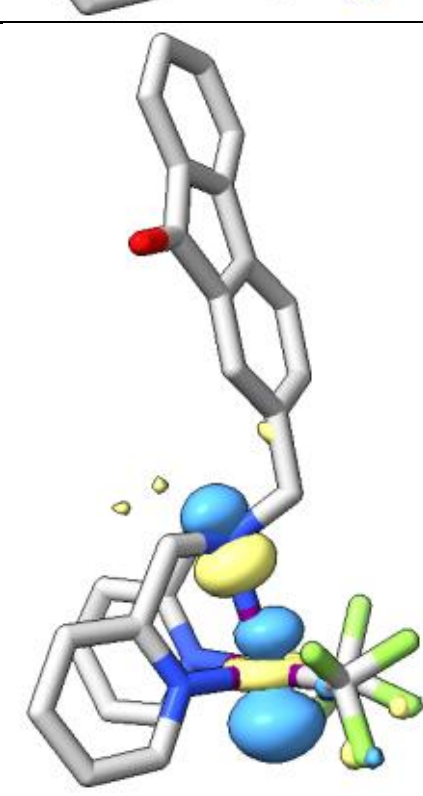 | 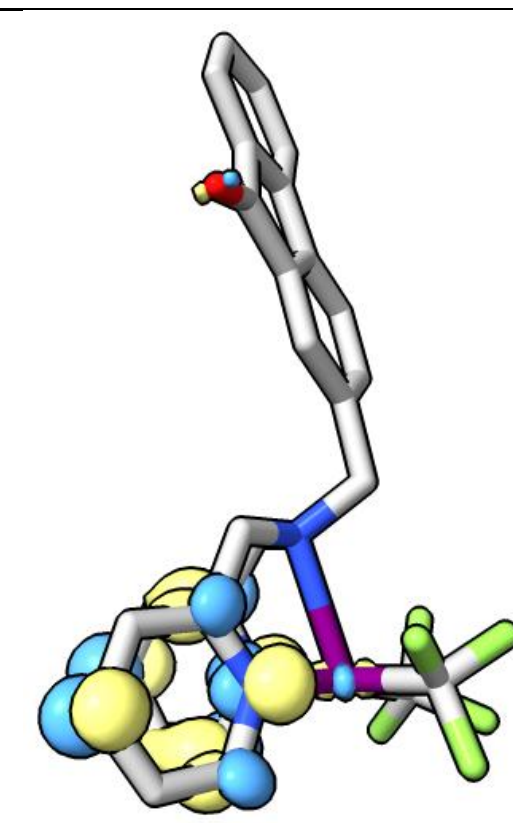 |
| **4**<br>**448 nm**<br>**f = 0.0007** | 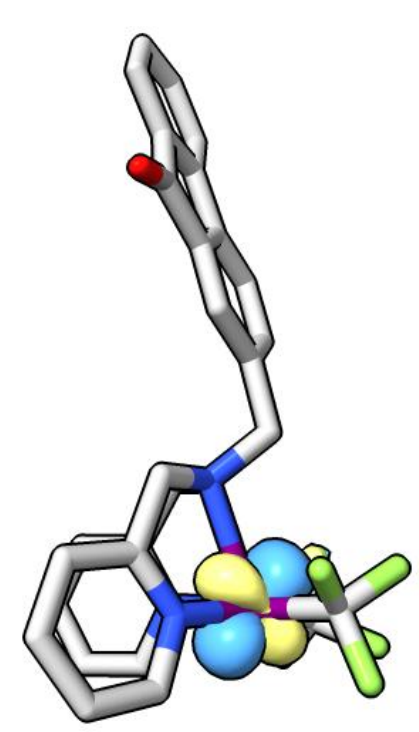 | 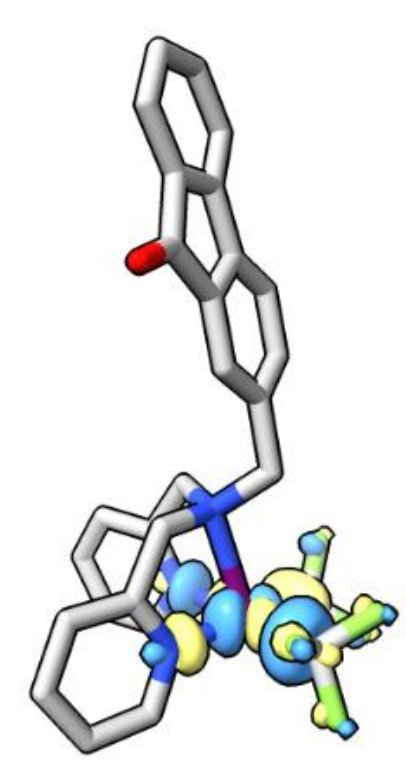 |

| | | |
|---|---|---|
| **5**<br>**436 nm**<br>**f = 0.0004** | 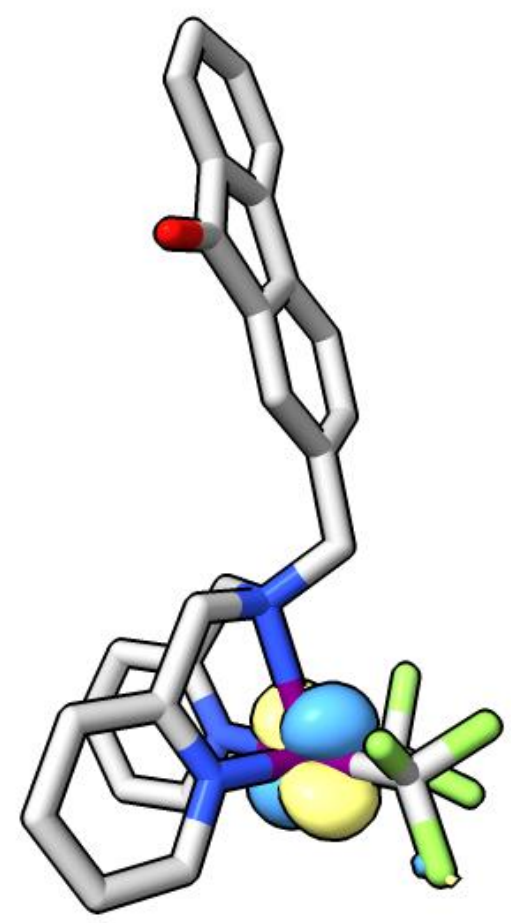 | 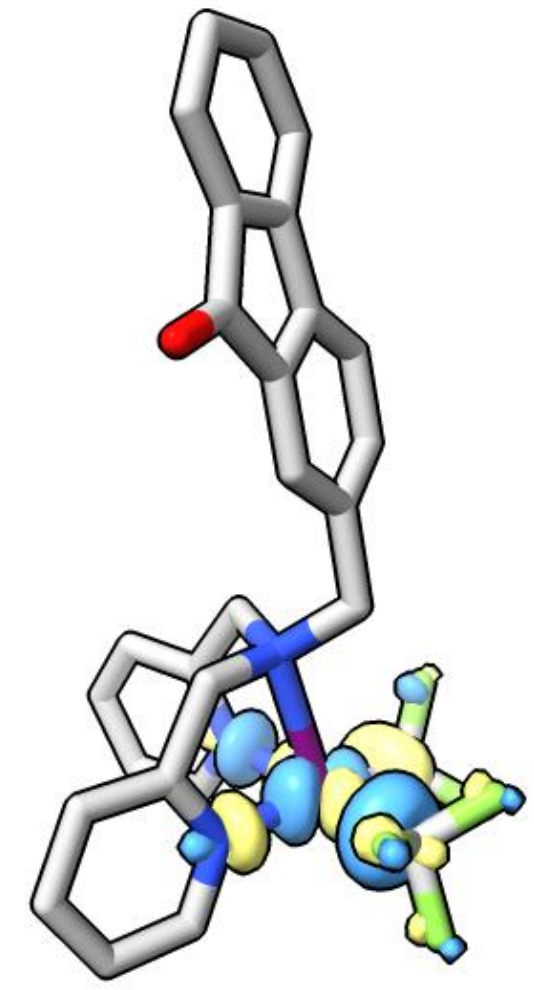 |
| **6**<br>**412 nm**<br>**f = 0.0004** | 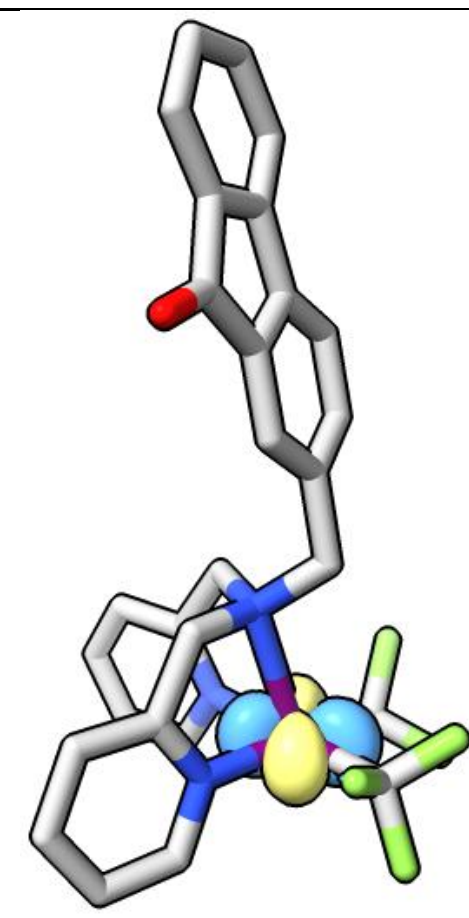 | 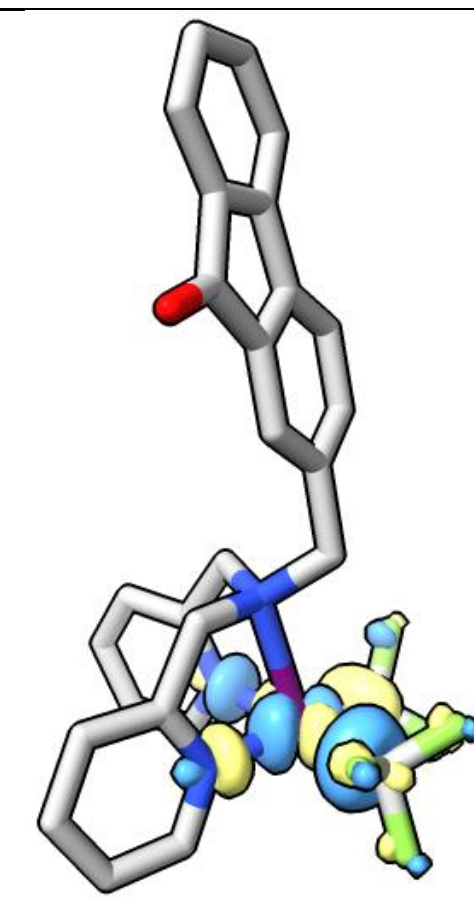 |
| **7**<br>**396 nm**<br>**f = 0.0066** | 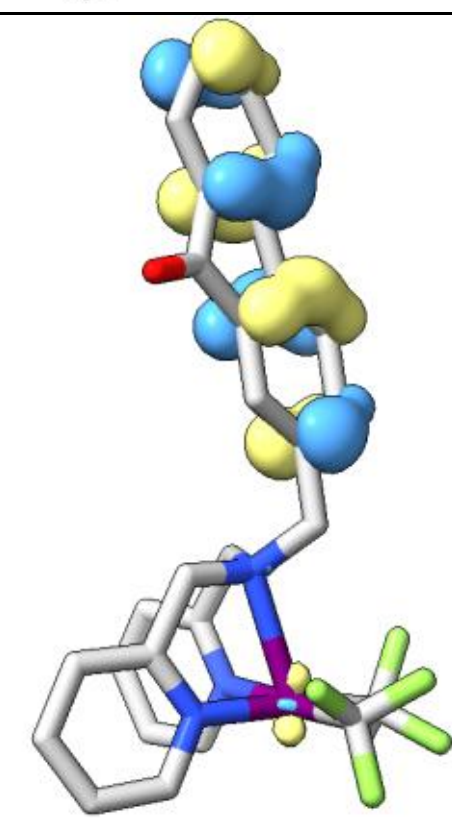 | 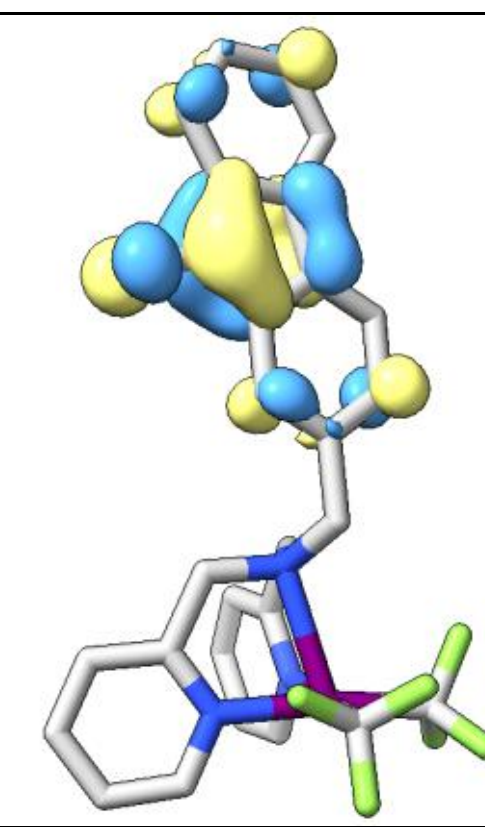 |

| | | |
|---|---|---|
| **8**<br>**386 nm**<br>**f = 0.0009** | 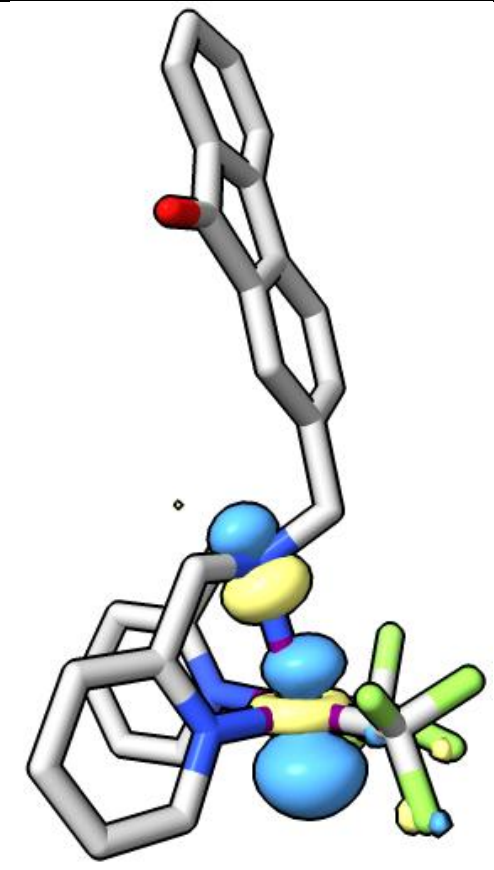 | 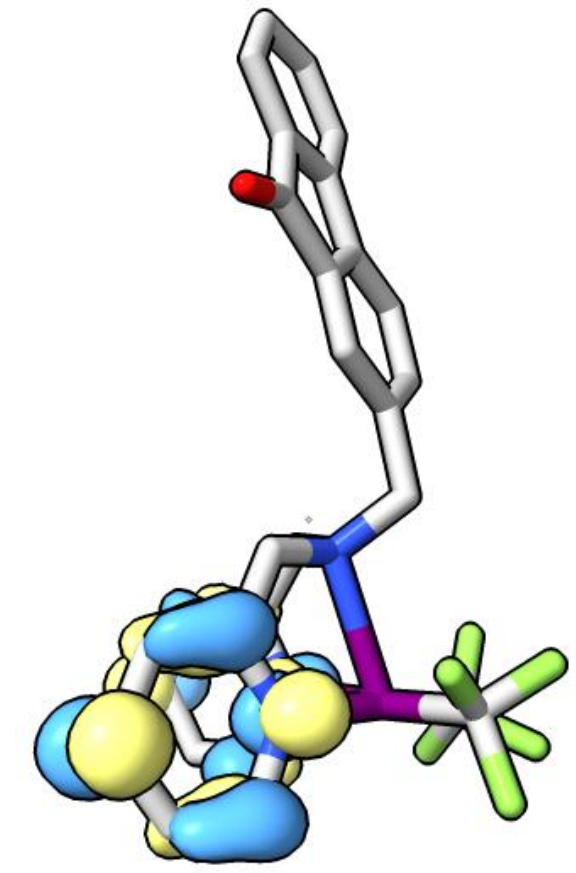 |
| **9**<br>**379 nm**<br>**f = 0.0074** | 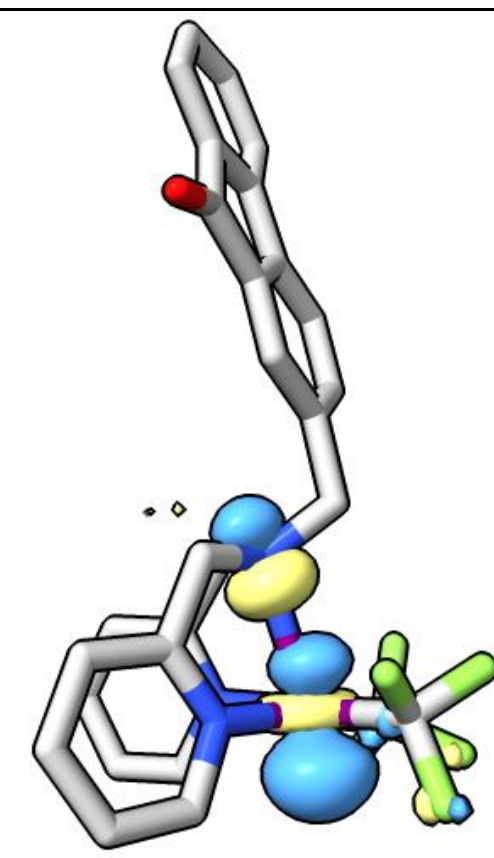 | 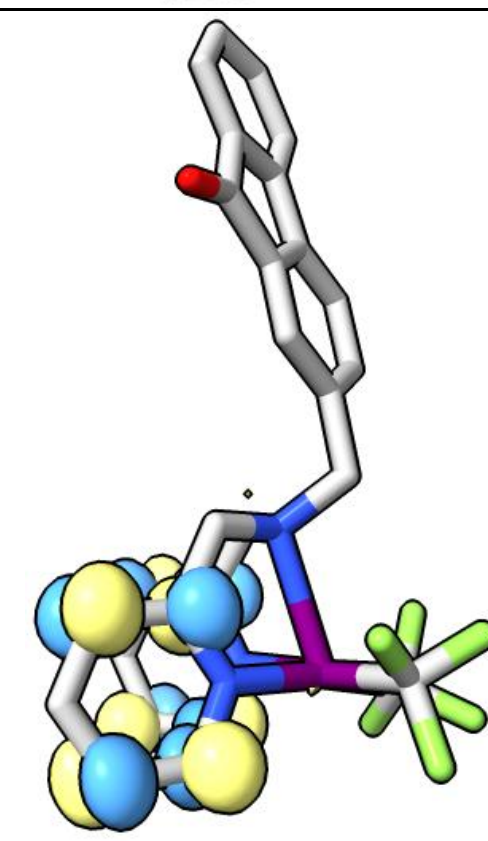 |
| **10**<br>**379 nm**<br>**f = 0.0001** | 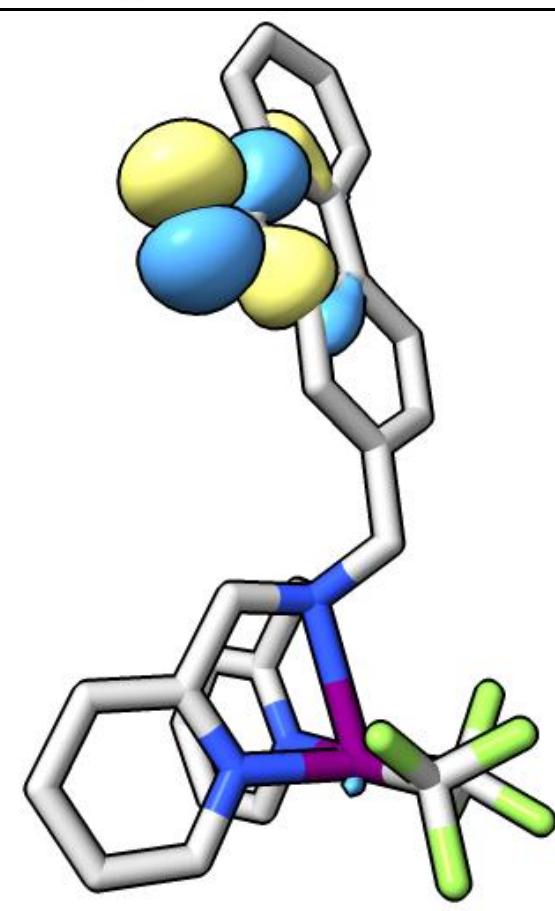 | 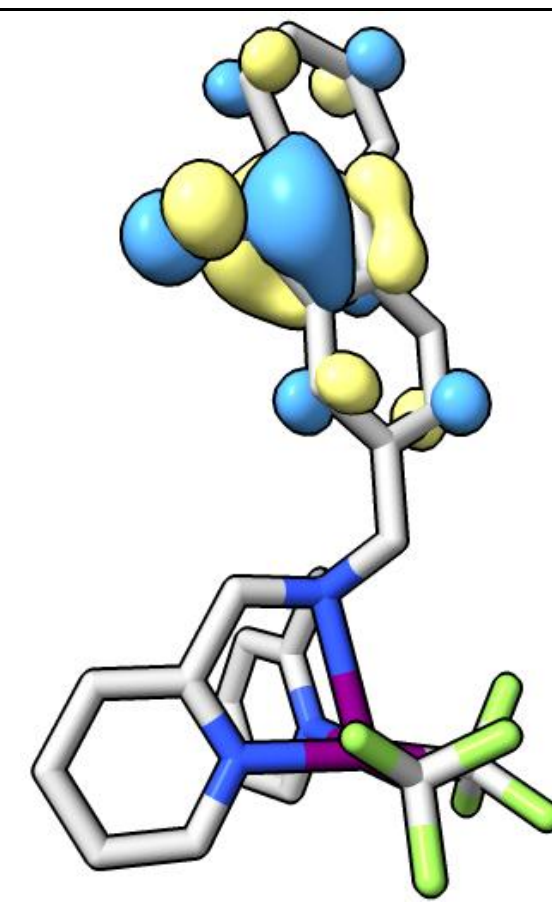 |

**Computed UV-Visible Spectrum**

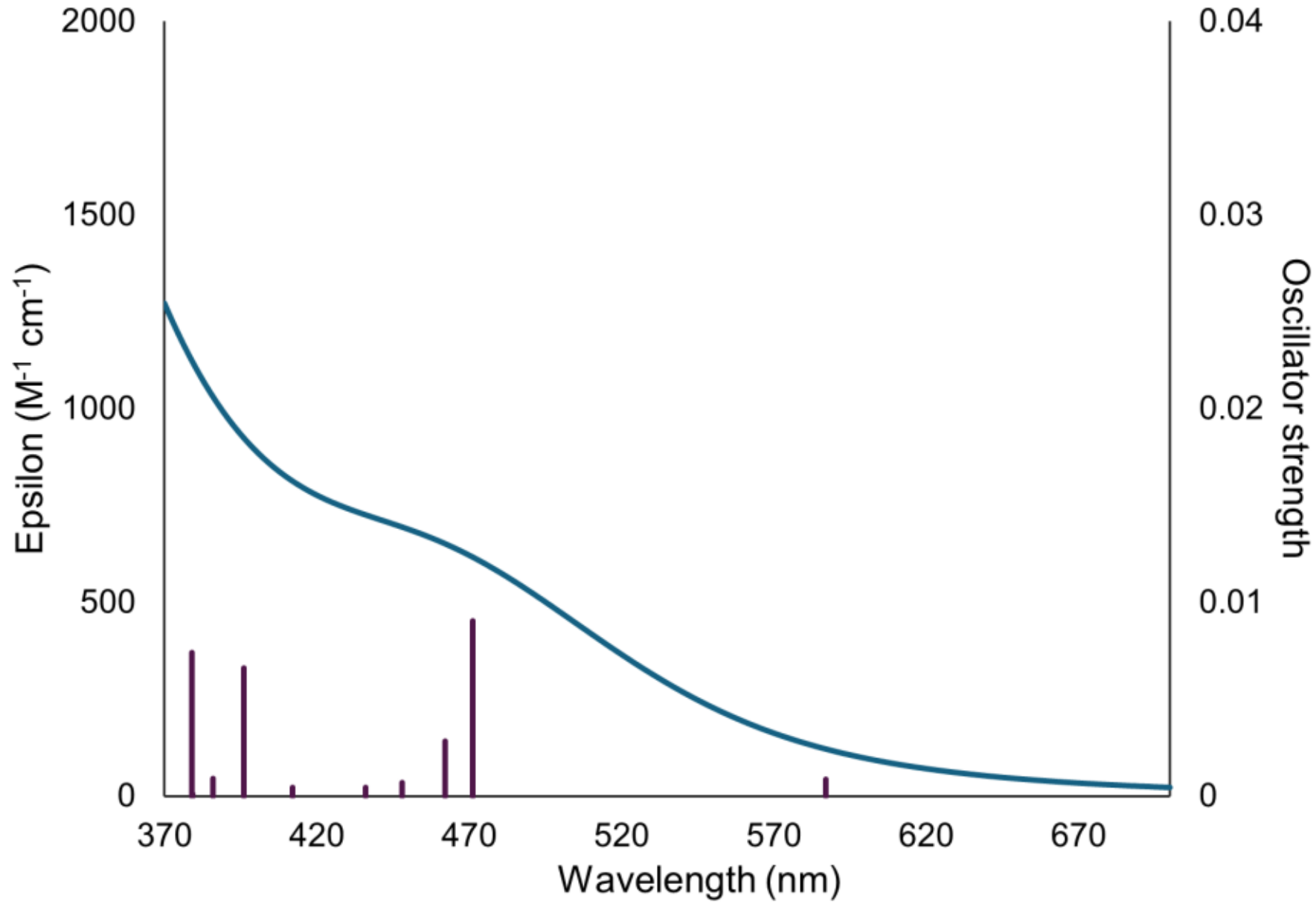


**Figure S40:** Computed UV-Visible Spectrum of **($^{Fluor}N_3$)Ni($CF_3$)$_2$** with electronic transitions depicted in purple.

**TD-DFT calculation, triplets**
# td(triplets, nstates=50) um06 def2tzvp scrf=(solvent=benzene) Pop=NPA Geom=AllCheck Guess=Read

**Visualization of lowest energy triplet state:**

| Hole | Particle |
|---|---|
| 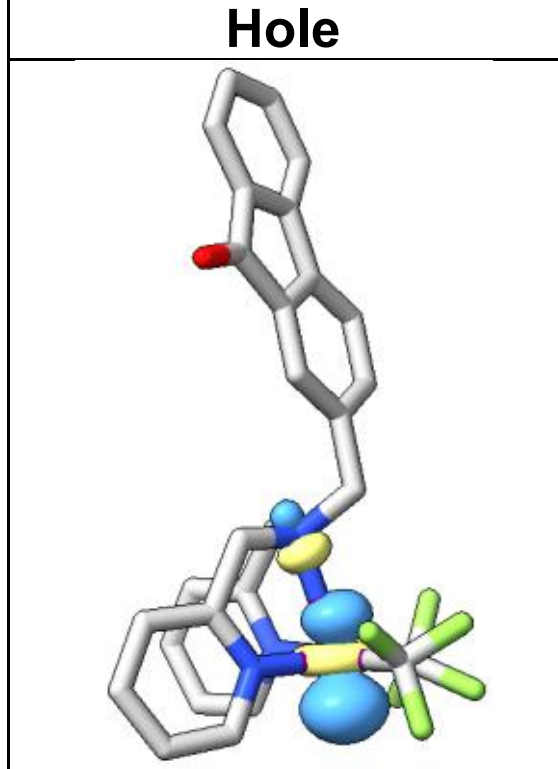 | 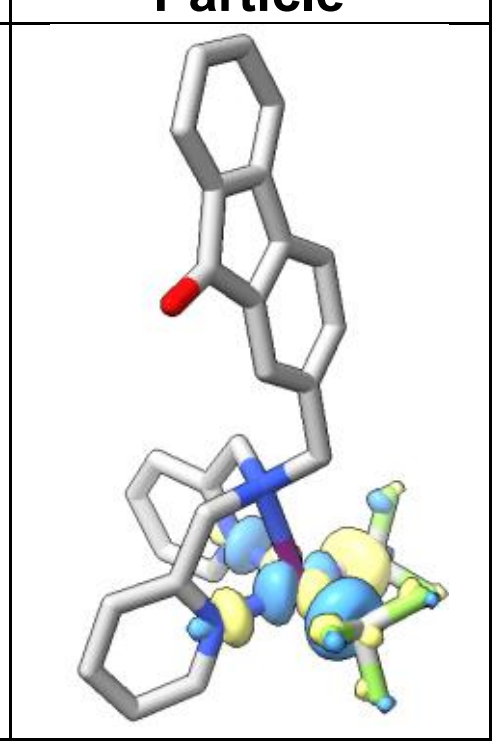 |

**Triplet state geometry optimization:**
# opt freq um06 tzvp scrf=(solvent=benzene)
0 3

**Output of triplet state geometry optimization:**
Zero-point correction= 0.437533 (Hartree/Particle)
Thermal correction to Energy= 0.471803
Thermal correction to Enthalpy= 0.472747
Thermal correction to Gibbs Free Energy= 0.366458
Sum of electronic and zero-point Energies= -3425.564897
Sum of electronic and thermal Energies= -3425.530627
Sum of electronic and thermal Enthalpies= -3425.529683

Sum of electronic and thermal Free Energies= -3425.635972

**Cartesian coordinates of optimized triplet state geometry:**

| | | | |
|---|---|---|---|
| Ni | 2.580588 | -0.674303 | -0.318677 |
| F | 1.808886 | -2.640397 | 1.682603 |
| F | 3.912159 | -2.712517 | 1.185078 |
| F | 2.458953 | -3.639839 | -0.105666 |
| F | 2.310429 | -2.424847 | -2.703727 |
| F | 3.813602 | -0.891398 | -2.886076 |
| F | 1.718712 | -0.392610 | -3.076870 |
| O | -4.876293 | 1.500490 | -2.273625 |
| N | 2.807318 | 0.262362 | 1.650011 |
| N | 2.755681 | 1.422633 | -0.877007 |
| N | 0.556849 | -0.052305 | 0.008315 |
| C | 2.680829 | -2.510176 | 0.608197 |
| C | 2.591066 | -1.157845 | -2.315767 |
| C | 3.936452 | 0.653361 | 2.229264 |
| H | 4.853599 | 0.236129 | 1.823172 |
| C | 3.958089 | 1.538305 | 3.291991 |
| H | 4.899214 | 1.830571 | 3.739043 |
| C | 2.753367 | 2.042306 | 3.750190 |
| H | 2.730044 | 2.748784 | 4.571452 |
| C | 1.575984 | 1.630235 | 3.149896 |
| H | 0.614696 | 2.001711 | 3.485229 |
| C | 1.642403 | 0.728451 | 2.100804 |
| C | 0.412310 | 0.158833 | 1.458506 |
| H | 0.244754 | -0.820908 | 1.915377 |
| H | -0.463949 | 0.781043 | 1.683347 |
| C | 0.361250 | 1.194376 | -0.750367 |
| H | -0.494424 | 1.766109 | -0.367476 |
| H | 0.135867 | 0.913596 | -1.783715 |
| C | 1.587035 | 2.059248 | -0.800541 |
| C | 1.507074 | 3.441438 | -0.845566 |
| H | 0.543240 | 3.931286 | -0.771435 |
| C | 2.673567 | 4.172378 | -0.987849 |
| H | 2.638865 | 5.254702 | -1.025584 |
| C | 3.882119 | 3.504819 | -1.085826 |
| H | 4.815068 | 4.040049 | -1.204410 |
| C | 3.875128 | 2.123905 | -1.017174 |
| H | 4.795735 | 1.550587 | -1.079004 |
| C | -0.335417 | -1.143267 | -0.473586 |
| H | 0.028147 | -2.067908 | -0.020272 |
| H | -0.170431 | -1.227634 | -1.549982 |
| C | -1.796490 | -0.963451 | -0.167321 |
| C | -2.365128 | -1.624622 | 0.919354 |
| H | -1.737772 | -2.282224 | 1.514416 |
| C | -3.709712 | -1.487835 | 1.251803 |
| H | -4.119905 | -2.025272 | 2.099629 |
| C | -4.496493 | -0.666573 | 0.472511 |
| C | -5.926570 | -0.322102 | 0.548463 |
| C | -6.909828 | -0.708793 | 1.434784 |
| H | -6.694395 | -1.383169 | 2.256334 |
| C | -8.198209 | -0.212093 | 1.249571 |
| H | -8.984376 | -0.505860 | 1.935679 |
| C | -8.496099 | 0.650131 | 0.203956 |
| H | -9.508045 | 1.018546 | 0.085257 |

| | | | |
|---|---|---|---|
| C | -7.504122 | 1.040168 | -0.690245 |
| H | -7.716512 | 1.711743 | -1.514994 |
| C | -6.231825 | 0.547420 | -0.504758 |
| C | -4.997923 | 0.796044 | -1.301649 |
| C | -3.937323 | -0.008803 | -0.627603 |
| C | -2.611286 | -0.151821 | -0.961568 |
| H | -2.220625 | 0.346085 | -1.844214 |

**Table S3:** Selected bond lengths for optimized triplet state **($^{Fluor}N_3$)Ni($CF_3$)$_2$**

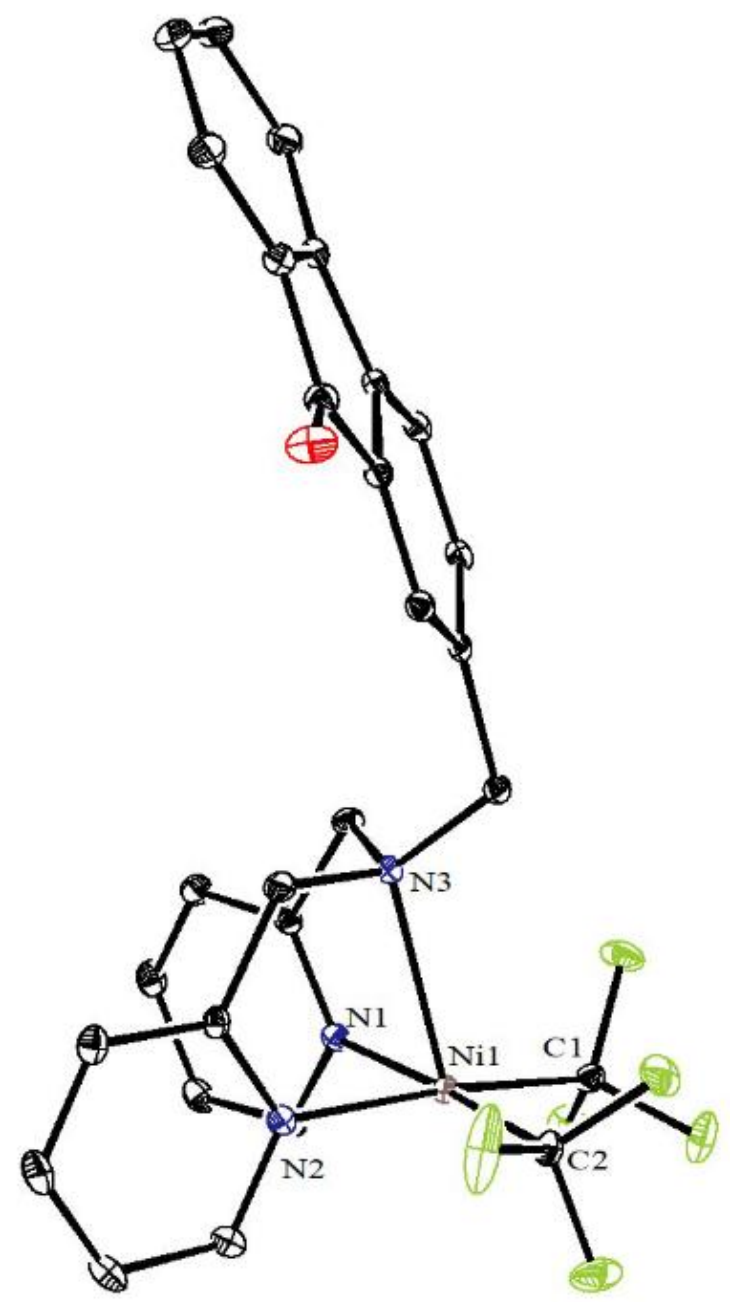


| Bond | Length (Å) |
|---|---|
| Ni-N1 | 2.19191 |
| Ni-N2 | 2.17705 |
| Ni-N3 | 2.14227 |
| Ni-C1 | 2.05902 |
| Ni-C2 | 2.05476 |

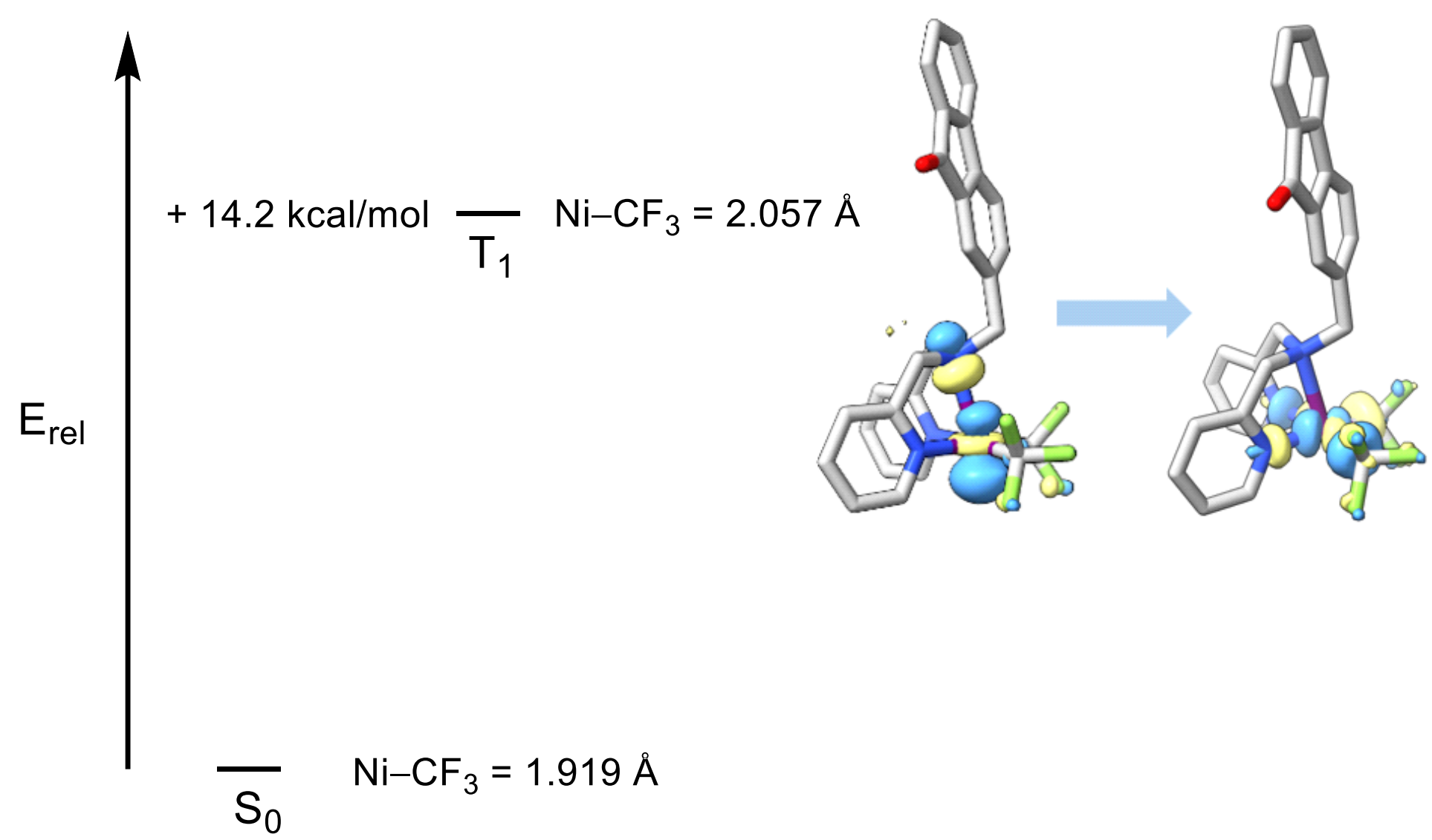


**Figure S41**: Relative energy difference between the optimized ground state and triplet state of **($^{Fluor}N_3$)Ni($CF_3$)$_2$** . Ni-$CF_3$ bonds lengthen by an average of 0.138 Å.

ii. $(^{Bn}N_3)Ni(CF_3)_2$

**Geometry optimization:**
# opt freq m06 tzvp scrf=(solvent=benzene)

**Output of ground state geometry optimization:**
Zero-point correction = 0.370929 Hartree/Particle
Thermal correction to Energy = 0.398602
Thermal correction to Enthalpy = 0.399546
Thermal correction to Gibbs Free Energy = 0.311068
Sum of electronic and zero-point Energies = -3082.598588
Sum of electronic and thermal Energies = -3082.570916
Sum of electronic and thermal Enthalpies = -3082.569971
Sum of electronic and thermal Free Energies = -3082.658450

**Cartesian coordinates of optimized ground state geometry:**

| | | | |
|---|---|---|---|
| Ni | 1.235163 | -0.673730 | 0.006030 |
| F | 0.267644 | -2.737883 | -1.550847 |
| F | 2.332548 | -2.149372 | 2.055338 |
| F | 1.899488 | -1.695950 | -2.482911 |
| F | 2.300261 | -3.099720 | -0.920560 |
| F | 0.587932 | -3.133246 | 1.264261 |
| F | 0.356078 | -1.399359 | 2.491027 |
| N | 1.340645 | 0.860288 | 1.286131 |
| N | 1.329818 | 0.729315 | -1.393918 |
| N | -1.085217 | 0.216051 | 0.007666 |
| C | 0.296750 | 1.664258 | 1.495493 |
| C | -3.501201 | -0.454175 | 0.092906 |
| C | 2.540110 | 1.201692 | 1.765190 |
| H | 3.345392 | 0.500351 | 1.573041 |
| C | 0.214659 | 1.381319 | -1.741553 |
| C | -2.056809 | -0.869063 | 0.201703 |
| H | -1.855908 | -1.307200 | 1.183505 |
| H | -1.823331 | -1.641340 | -0.537109 |
| C | 1.442443 | -2.107570 | -1.257474 |
| C | 2.511371 | 1.178762 | -1.817923 |
| H | 3.376838 | 0.597600 | -1.517832 |
| C | -1.073221 | 1.205396 | 1.063533 |
| H | -1.529365 | 0.759387 | 1.953741 |
| H | -1.688694 | 2.086992 | 0.818636 |
| C | -4.156271 | -0.474687 | -1.135361 |
| H | -3.632005 | -0.842868 | -2.012353 |
| C | 1.126314 | -1.912095 | 1.469304 |
| C | -1.095456 | 0.773998 | -1.328540 |
| H | -1.900231 | 1.514509 | -1.472994 |
| H | -1.293484 | -0.048763 | -2.026762 |
| C | 0.441243 | 2.862420 | 2.180990 |
| H | -0.423657 | 3.499860 | 2.325635 |
| C | 2.754265 | 2.367821 | 2.469350 |
| H | 3.742492 | 2.601479 | 2.842559 |
| C | -4.209636 | -0.024933 | 1.211843 |
| H | -3.730370 | -0.041515 | 2.186334 |
| C | -5.472255 | -0.056826 | -1.248451 |
| H | -5.967227 | -0.086632 | -2.212550 |
| C | 0.266345 | 2.527376 | -2.518960 |
| H | -0.654194 | 3.036928 | -2.777776 |

| | | | |
|---|---|---|---|
| C | 1.681151 | 3.219848 | 2.671720 |
| H | 1.810296 | 4.151155 | 3.210268 |
| C | 2.638393 | 2.308610 | -2.600613 |
| H | 3.618123 | 2.637101 | -2.921254 |
| C | -6.159007 | 0.384807 | -0.128494 |
| H | -7.190583 | 0.706548 | -0.213591 |
| C | 1.491913 | 3.000101 | -2.950314 |
| H | 1.551722 | 3.896160 | -3.556477 |
| C | -5.525971 | 0.394113 | 1.104282 |
| H | -6.063437 | 0.717848 | 1.988445x |

**Table S4:** Selected bond lengths for optimized ground state **$(^{Bn}N_3)Ni(CF_3)_2$**

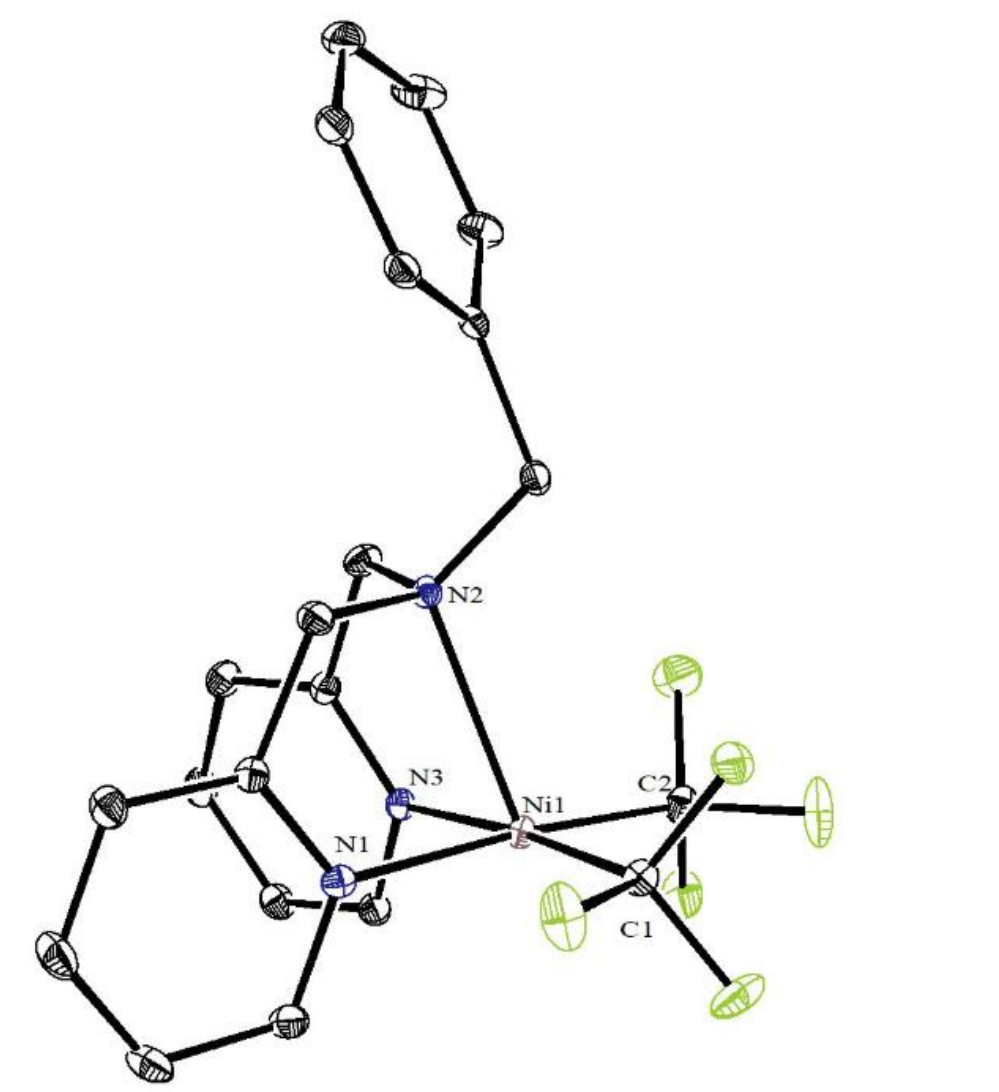


| Bond | Length (Å) |
|---|---|
| Ni-N1 | 1.98428 |
| Ni-N3 | 2.00075 |
| Ni-N2 | 2.48513 |
| Ni-C1 | 1.92232 |
| Ni-C2 | 1.92004 |

Visualized molecular orbitals for optimized ground state **$(^{Bn}N_3)Ni(CF_3)_2$**

| | | | | |
|---|---|---|---|---|
| | | | 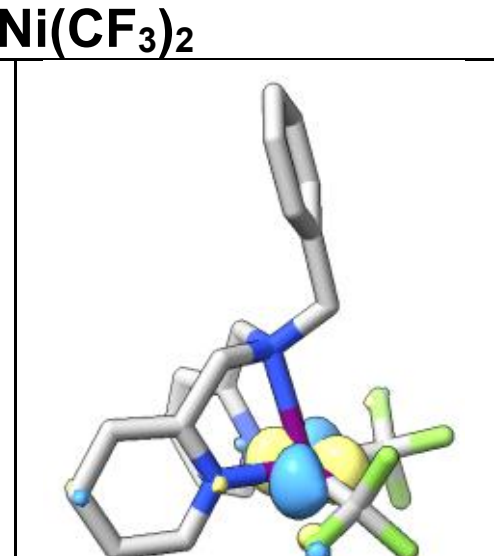 | 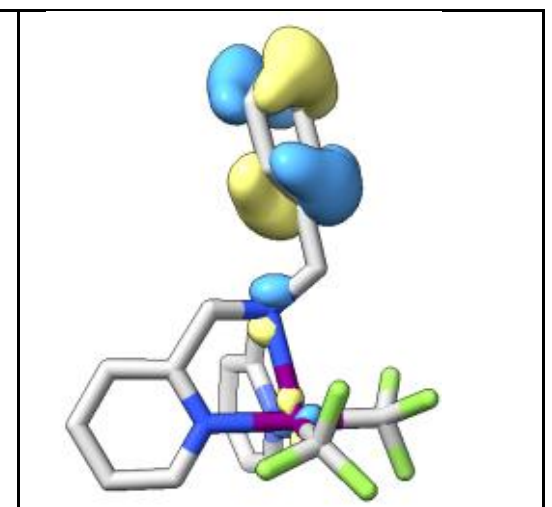 |
| | | | 120 | 121 |
| 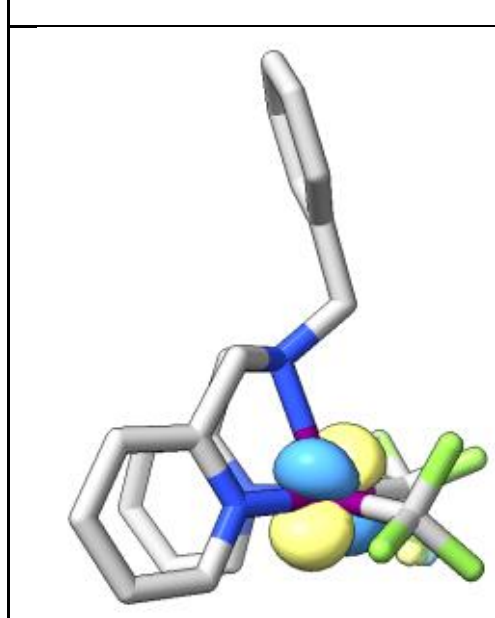 | 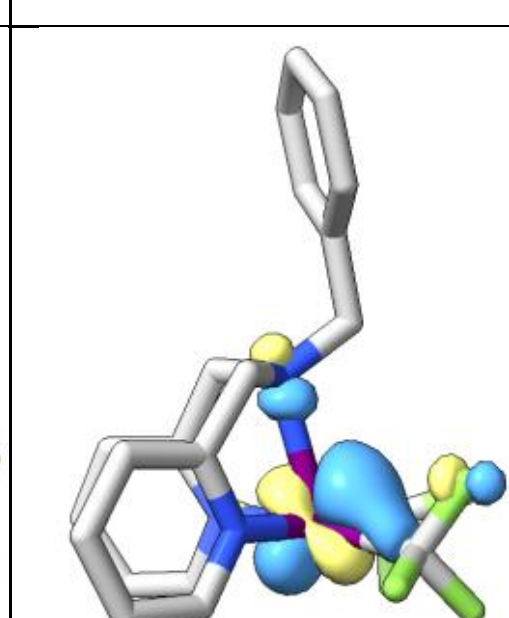 | 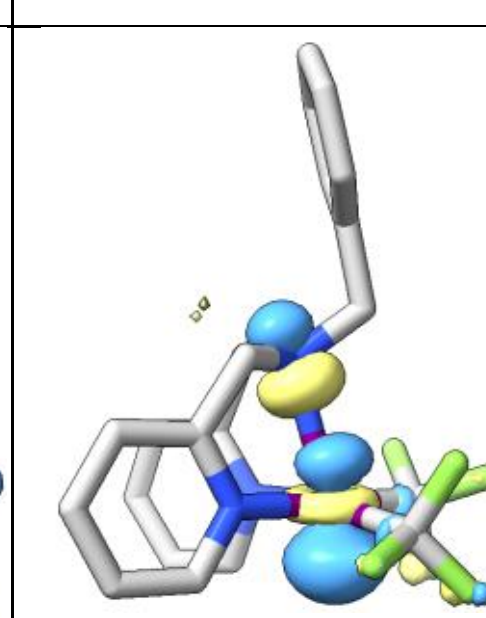 | 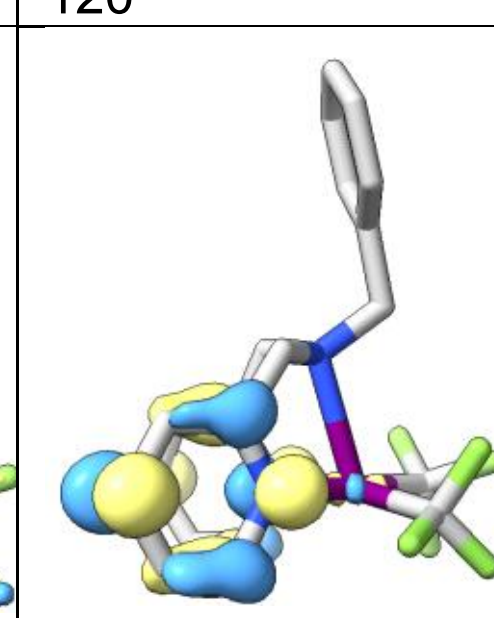 | 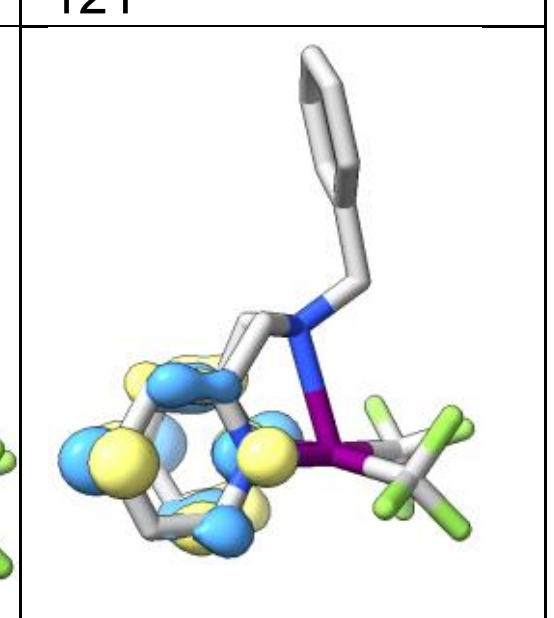 |
| 122 | 123 | 124 | 125 | 126 |

| | | | | |
|---|---|---|---|---|
| 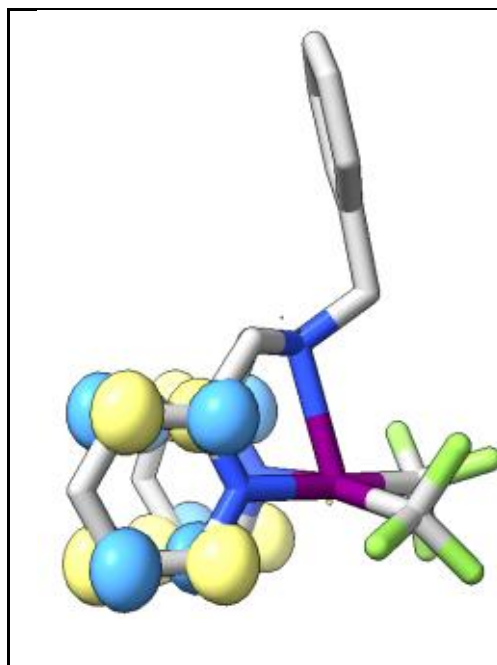 | 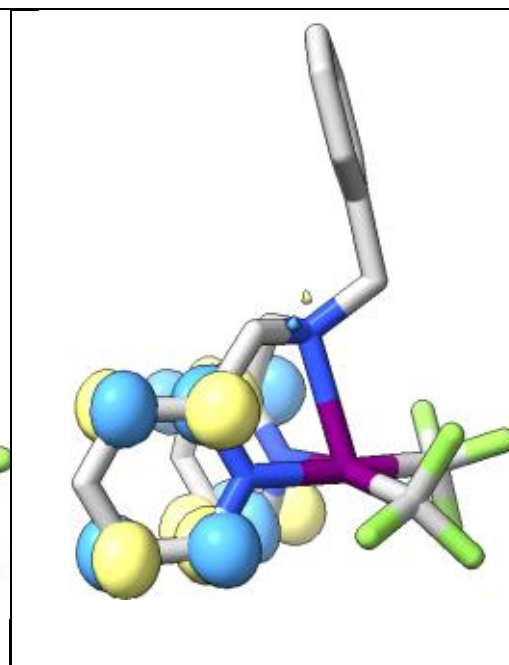 | 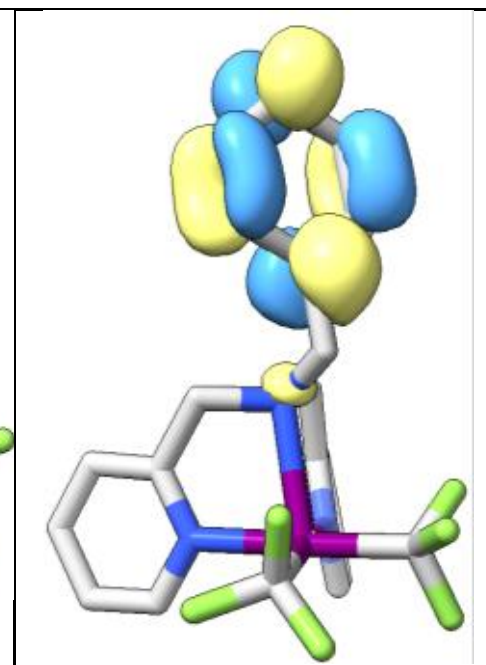 | 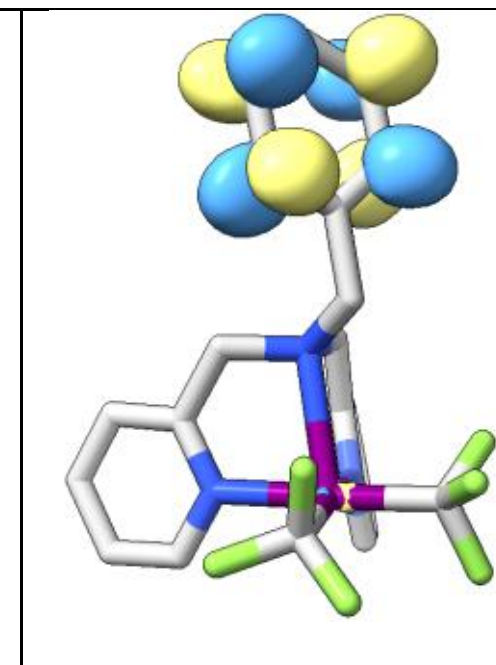 | 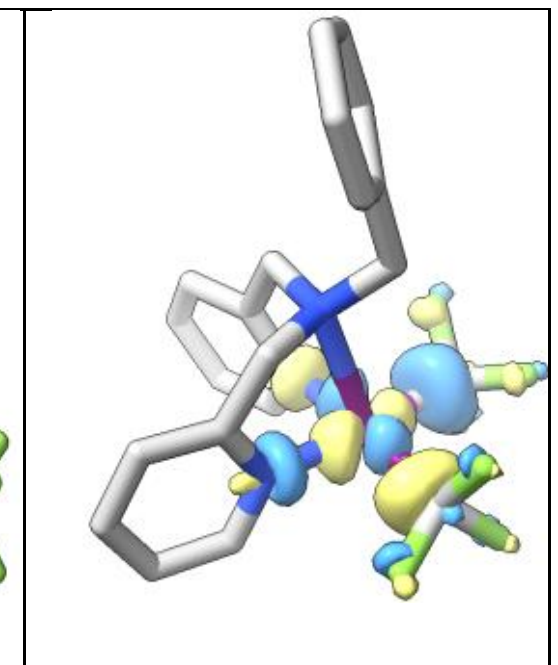 |
| 127 | 128 | 129 | 130 | 131 |

**Natural population analysis on optimized geometry:**
# freq scrf=(solvent=benzene) m06 def2tzvp Population=NPA Density=Current

**TD-DFT with natural population analysis on optimized geometry:**
# td(nstates=50) m06 def2tzvp scrf=(solvent=benzene) Pop=NPA Geom=AllCheck Guess=Read

**TD-DFT output: excited states with λ > 370 nm**

```
Excited State   1:      Singlet-A      2.0917 eV  592.74 nm  f=0.0006  <S**2>=0.000
Excited State   2:      Singlet-A      2.6503 eV  467.82 nm  f=0.0013  <S**2>=0.000
Excited State   3:      Singlet-A      2.7562 eV  449.84 nm  f=0.0007  <S**2>=0.000
Excited State   4:      Singlet-A      2.8331 eV  437.63 nm  f=0.0004  <S**2>=0.000
Excited State   5:      Singlet-A      3.0054 eV  412.54 nm  f=0.0005  <S**2>=0.000
Excited State   6:      Singlet-A      3.1837 eV  389.44 nm  f=0.0010  <S**2>=0.000
Excited State   7:      Singlet-A      3.2483 eV  381.70 nm  f=0.0037  <S**2>=0.000
```

**Natural transition orbitals calculated using the following input:**
# ChkBasis Guess=(Only,Read) Pop=SaveNTO Geom=AllCheck Density=(Check, Transition=n)

**Natural transition orbitals (NTOs) of excited states 1-7:**

| Excited State | Hole | Particle |
|---|---|---|
| **1**<br>**593**<br>**f = 0.0006** | 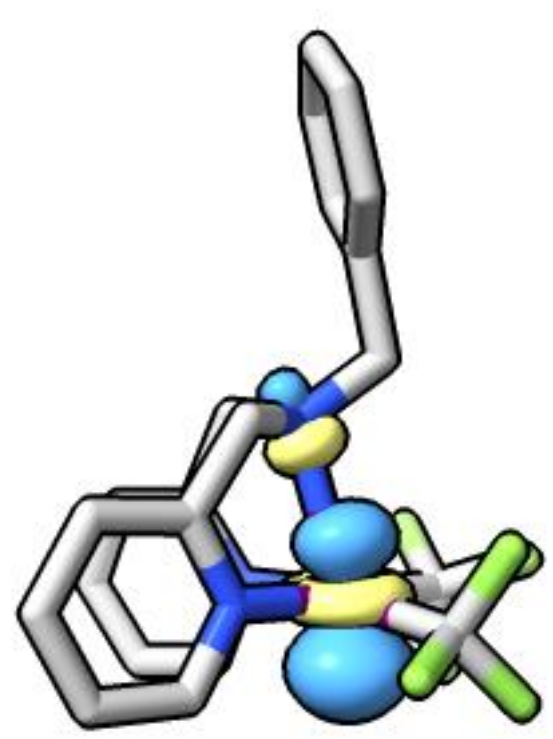 | 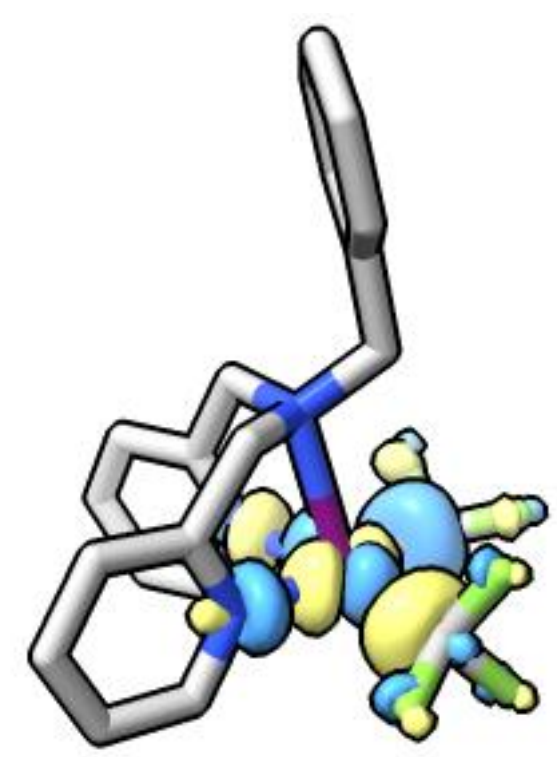 |

| | | |
|---|---|---|
| **2**<br>**468 nm**<br>**f = 0.0013** | 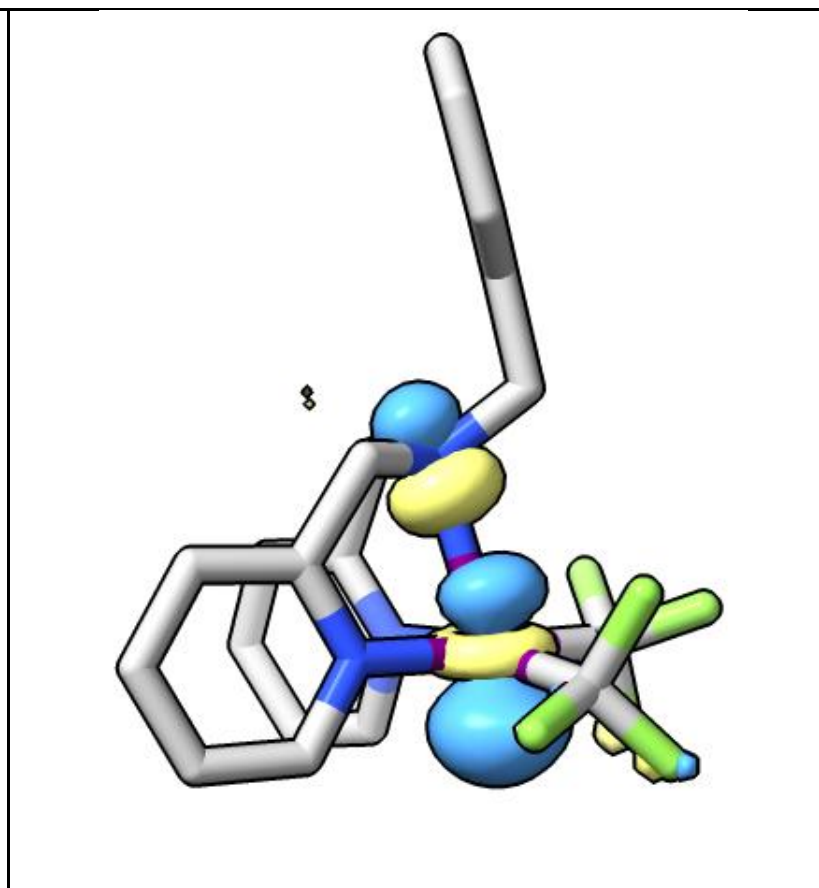 | 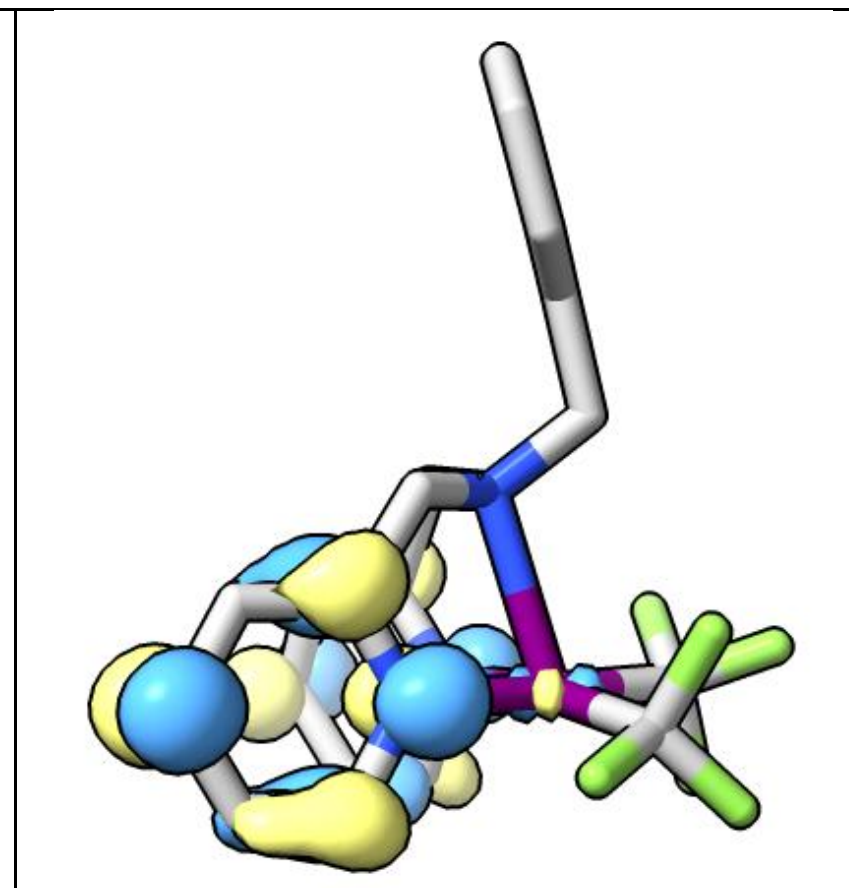 |
| **3**<br>**450 nm**<br>**f = 0.0007** | 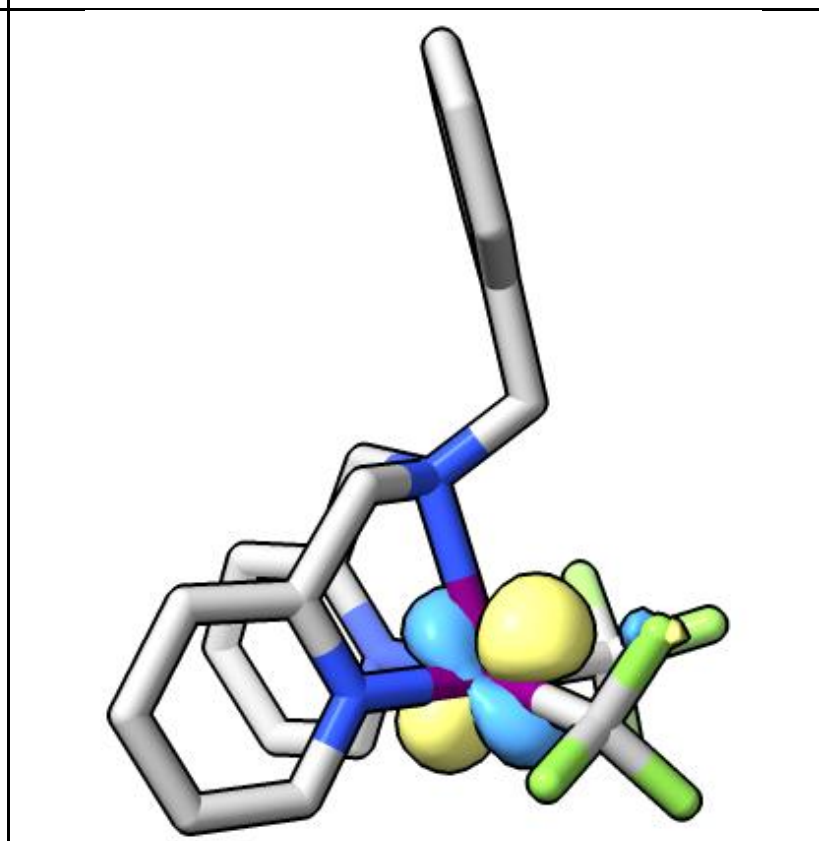 | 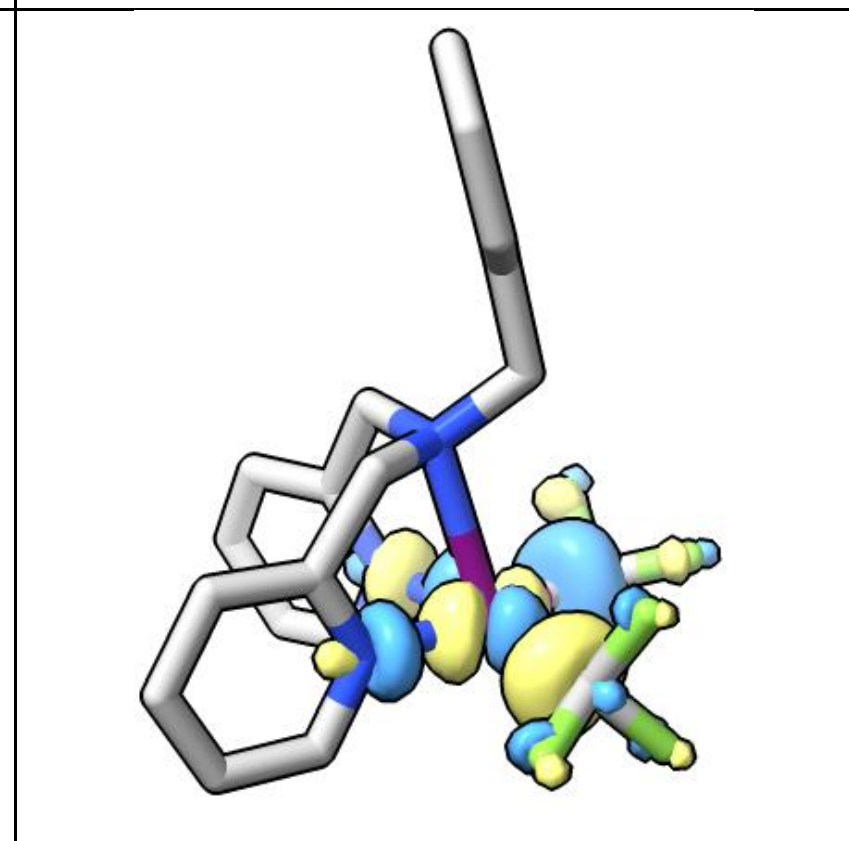 |
| **4**<br>**438 nm**<br>**f = 0.0004** | 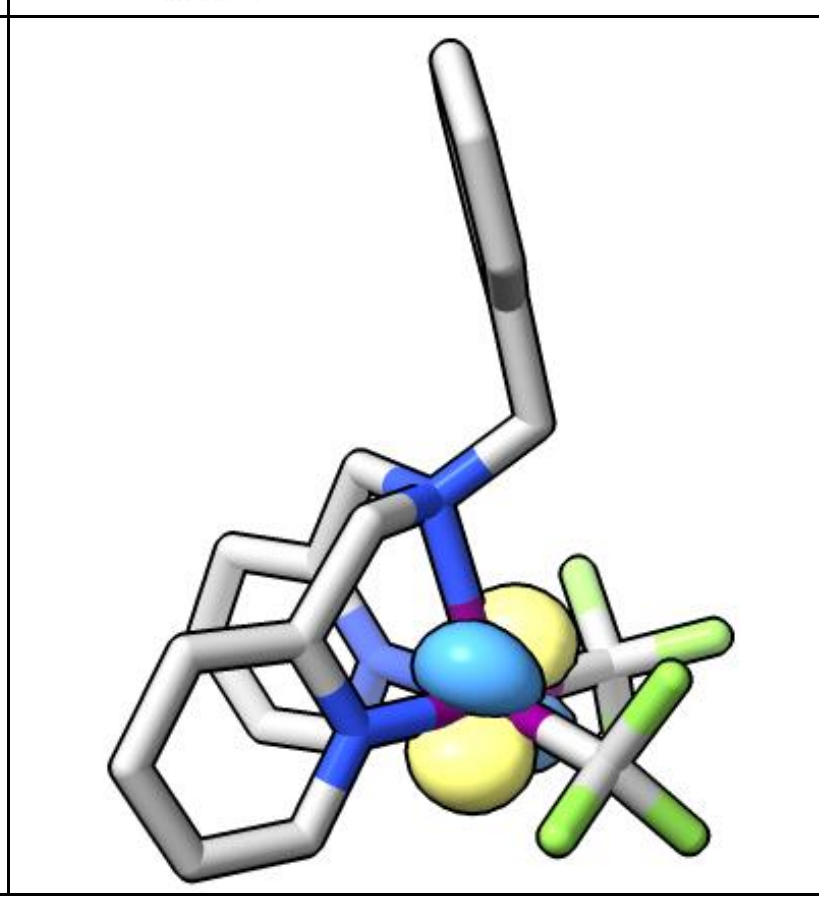 | 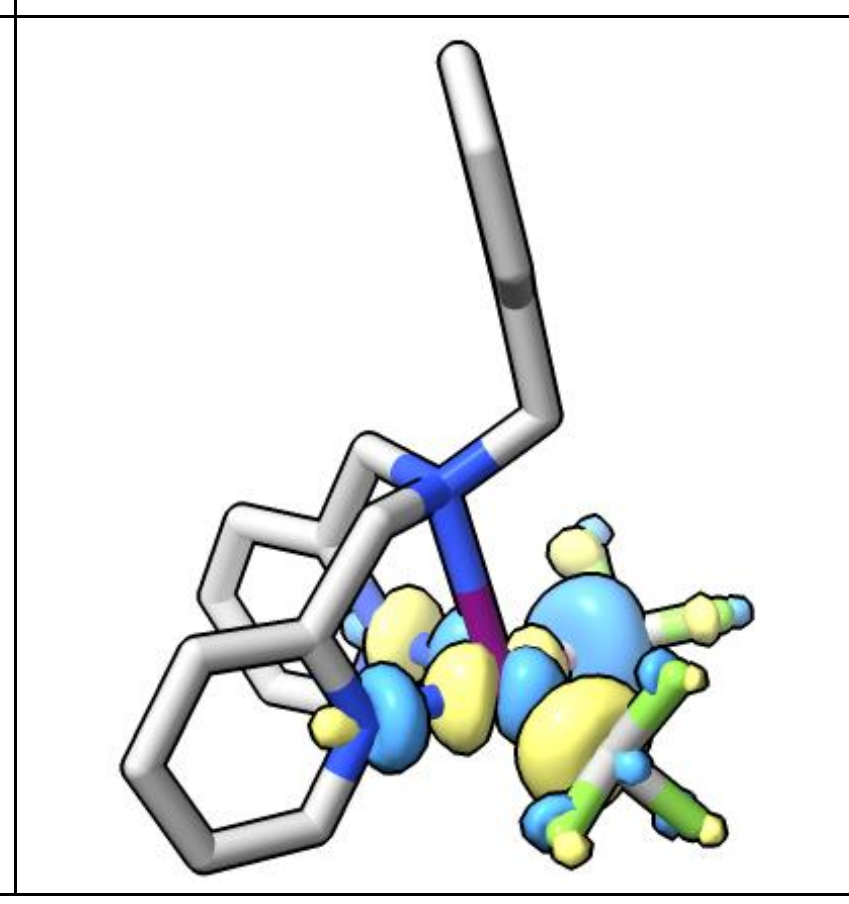 |

| | | |
|---|---|---|
| **5**<br>**413 nm**<br>**f = 0.0005** | 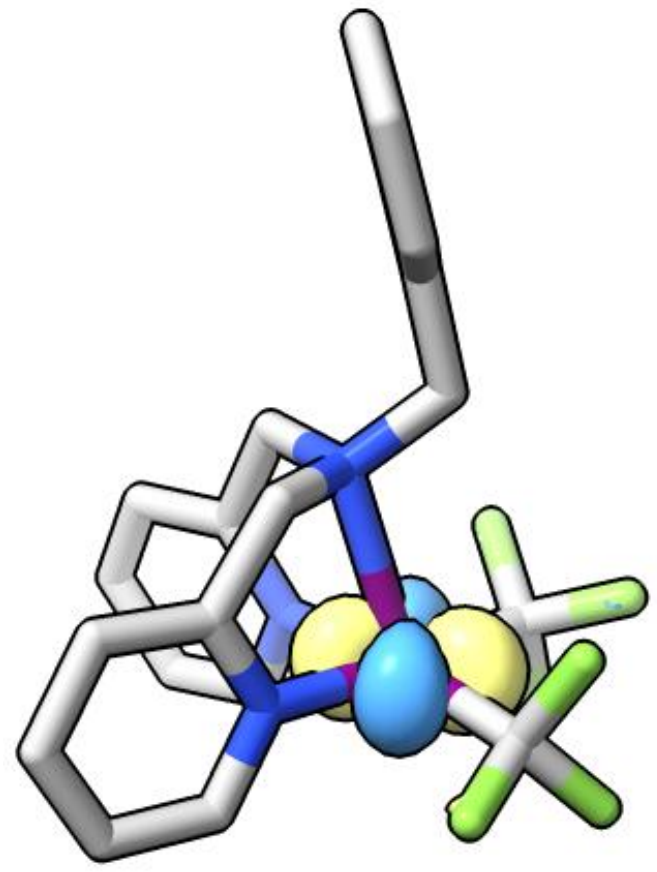 | 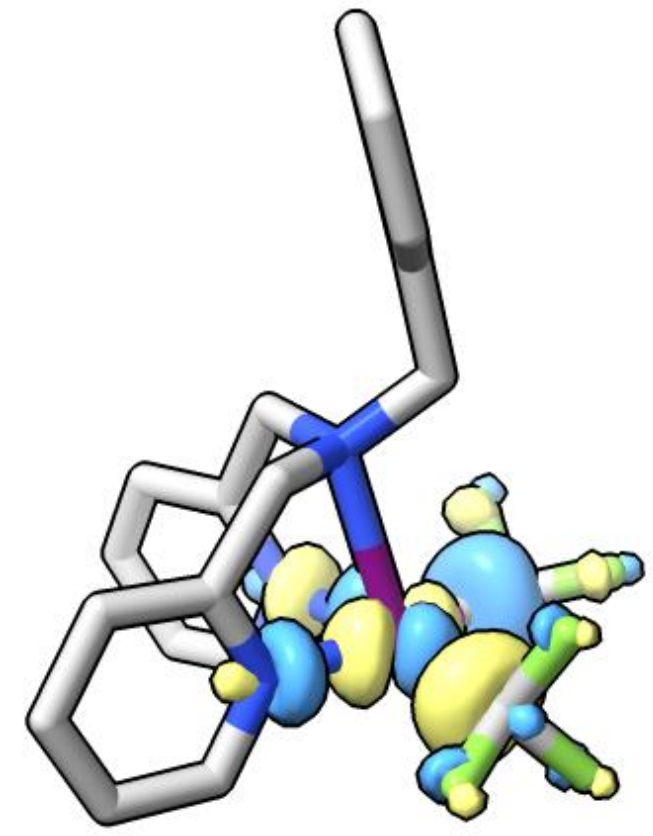 |
| **6**<br>**389**<br>**f = 0.0010** | 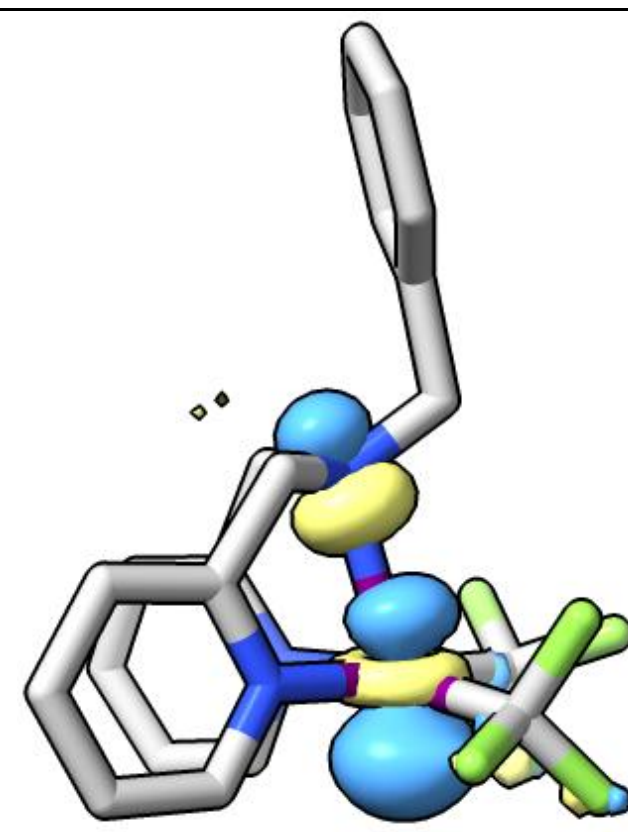 | 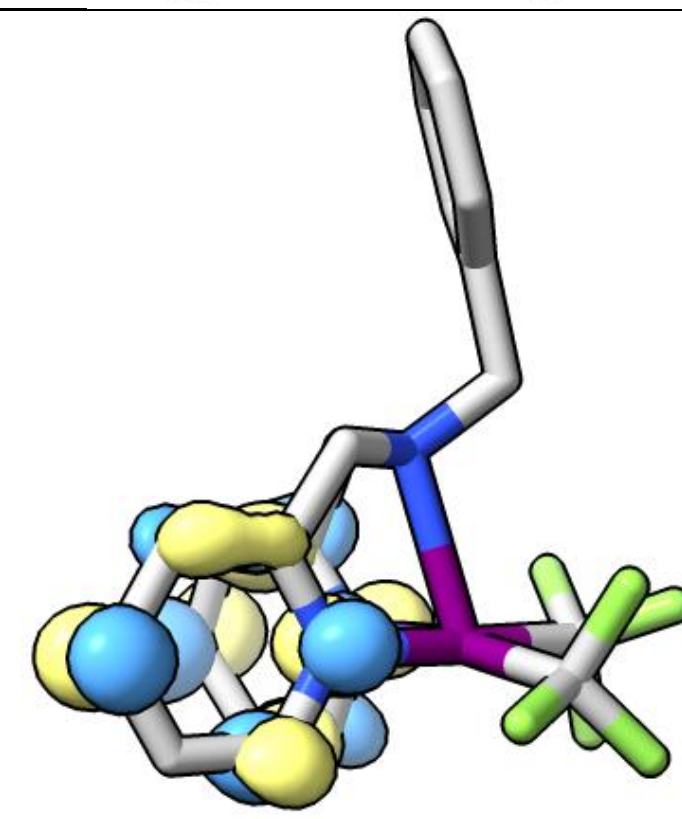 |
| **7**<br>**382 nm**<br>**f = 0.0037** | 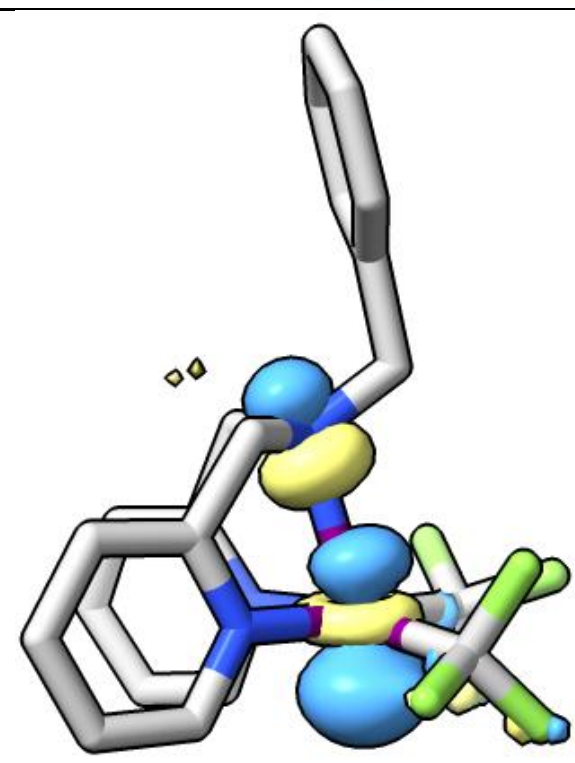 | 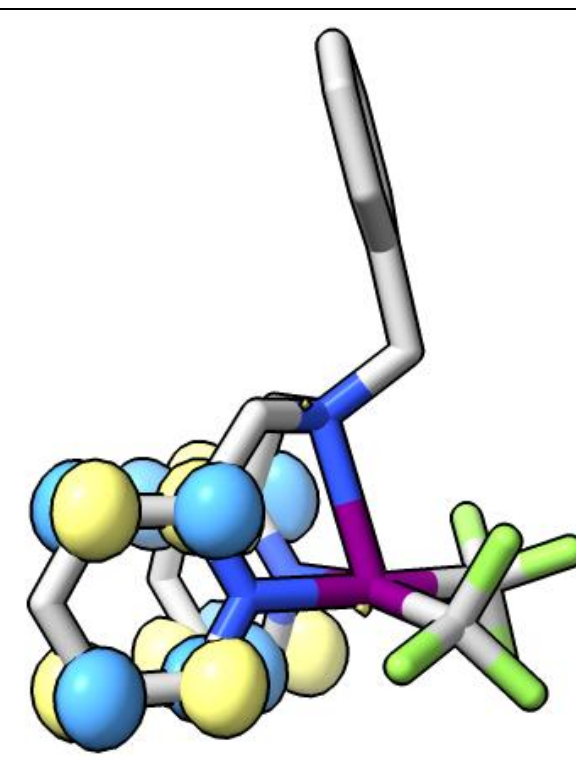 |

**Computed UV-Visible Spectrum**

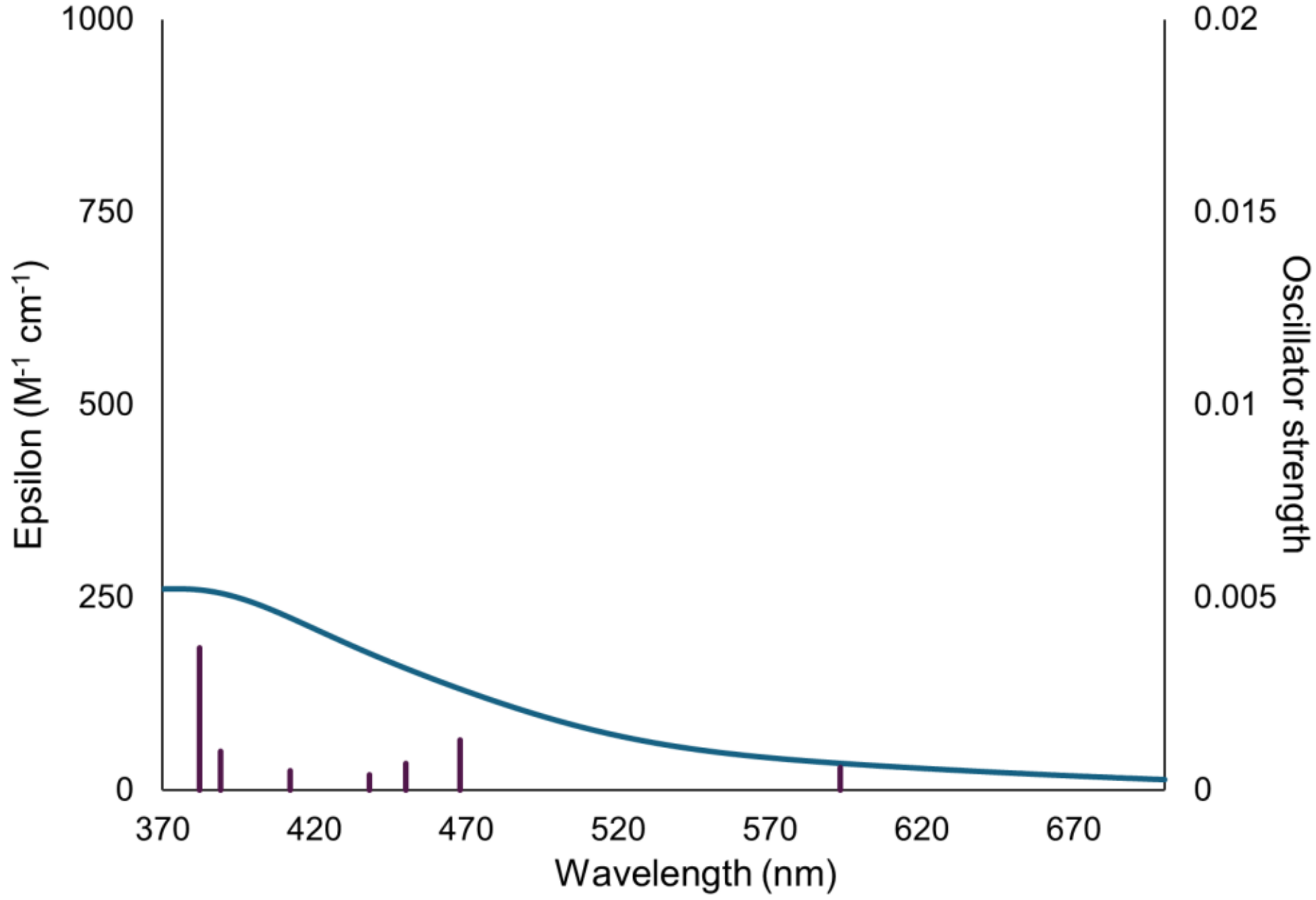


**Figure S42:** Computed UV-Visible spectrum of **($^{Bn}N_3$)Ni($CF_3$)$_2$** with electronic transitions depicted in purple.

**TD-DFT calculation, triplets**:
# td(triplets, nstates=50) um06 def2tzvp scrf=(solvent=benzene) Pop=NPA Geom=AllCheck Guess=Read

**Visualization of lowest energy triplet state:**

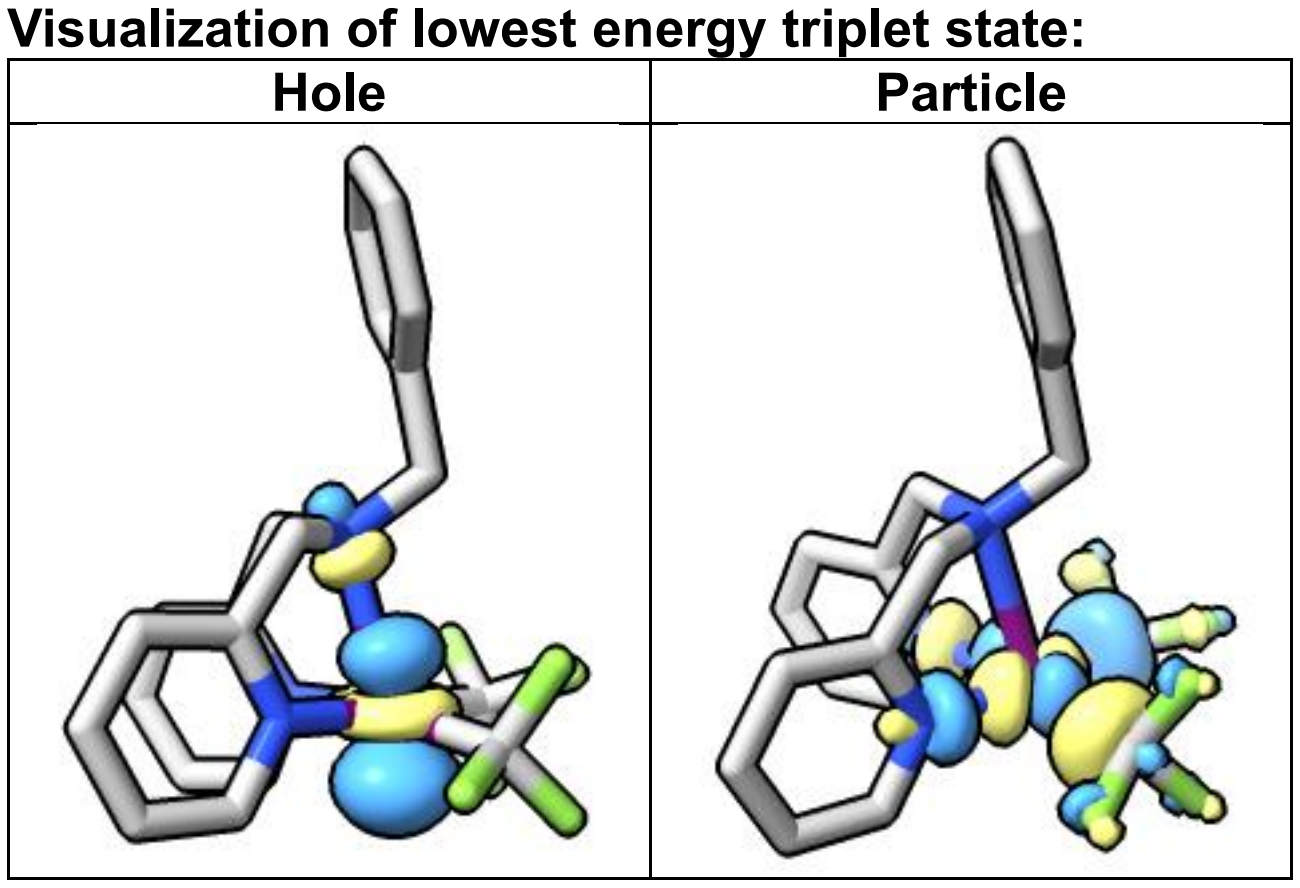


**Triplet state geometry optimization:**
# opt freq um06 tzvp scrf=(solvent=benzene)
0 3

**Output of triplet state geometry optimization:**

Zero-point correction= 0.368968 (Hartree/Particle)
Thermal correction to Energy= 0.397657
Thermal correction to Enthalpy= 0.398601
Thermal correction to Gibbs Free Energy= 0.306253
Sum of electronic and zero-point Energies= -3082.572401
Sum of electronic and thermal Energies= -3082.543712

Sum of electronic and thermal Enthalpies= -3082.542768
Sum of electronic and thermal Free Energies= -3082.635116

**Cartesian coordinates of optimized triplet geometry:**

| | | | |
|---|---|---|---|
| Ni | -0.98537 | -0.91386 | 0.03906 |
| F | 0.4335 | -2.73251 | 1.85368 |
| F | -1.92056 | -2.69744 | -1.98978 |
| F | -1.05668 | -1.59258 | 2.90509 |
| F | -1.65638 | -3.21245 | 1.61739 |
| F | -0.10006 | -3.47693 | -1.13748 |
| F | -0.01096 | -1.84106 | -2.53239 |
| N | -1.57028 | 0.5545 | -1.44807 |
| N | -1.54217 | 0.79555 | 1.30928 |
| N | 0.83052 | 0.22271 | -0.02618 |
| C | -0.64165 | 1.50015 | -1.58745 |
| C | 3.34548 | -0.0543 | -0.11501 |
| C | -2.80599 | 0.78013 | -1.8804 |
| H | -3.51649 | -0.03052 | -1.74631 |
| C | -0.48204 | 1.55388 | 1.58725 |
| C | 1.99111 | -0.70858 | -0.12904 |
| H | 1.8493 | -1.2753 | -1.05167 |
| H | 1.89414 | -1.41511 | 0.69736 |
| C | -0.79745 | -2.15233 | 1.67402 |
| C | -2.75445 | 1.22442 | 1.6386 |
| H | -3.58269 | 0.56713 | 1.38953 |
| C | 0.75687 | 1.12564 | -1.18818 |
| H | 1.18681 | 0.5935 | -2.04174 |
| H | 1.36742 | 2.02502 | -1.03271 |
| C | 4.01873 | 0.16242 | 1.08448 |
| H | 3.56953 | -0.16914 | 2.01576 |
| C | -0.72193 | -2.30971 | -1.43984 |
| C | 0.85158 | 0.96233 | 1.24424 |
| H | 1.62742 | 1.73965 | 1.24258 |
| H | 1.10846 | 0.2444 | 2.03159 |
| C | -0.93699 | 2.73157 | -2.1487 |
| H | -0.16503 | 3.4874 | -2.23479 |
| C | -3.17951 | 1.9743 | -2.4693 |
| H | -4.19579 | 2.11943 | -2.81144 |
| C | 3.96739 | 0.32675 | -1.30082 |
| H | 3.4807 | 0.12268 | -2.24979 |
| C | 5.2634 | 0.76982 | 1.10221 |
| H | 5.77488 | 0.92426 | 2.04537 |
| C | -0.60793 | 2.78812 | 2.20291 |
| H | 0.27371 | 3.38471 | 2.40557 |
| C | -2.22696 | 2.97105 | -2.5907 |
| H | -2.48474 | 3.92701 | -3.03095 |
| C | -2.96836 | 2.43838 | 2.26634 |
| H | -3.97283 | 2.7509 | 2.52021 |
| C | 5.86025 | 1.16379 | -0.08495 |
| H | 6.83719 | 1.63314 | -0.07352 |
| C | -1.8722 | 3.23589 | 2.54479 |
| H | -2.00025 | 4.19818 | 3.02606 |
| C | 5.21212 | 0.93464 | -1.28817 |
| H | 5.68355 | 1.21806 | -2.22223 |

**Table S5:** Selected bond lengths for optimized triplet state **($^{Bn}N_3$)Ni($CF_3$)$_2$**

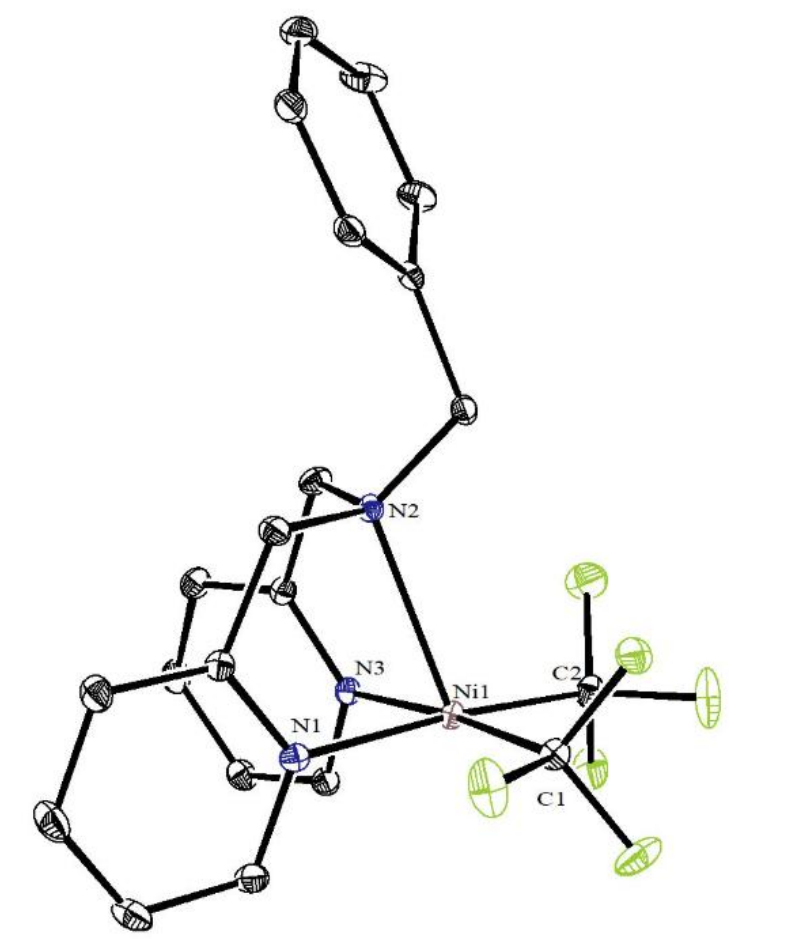


| Bond | Length (Å) |
|---|---|
| Ni-N1 | 2.20127 |
| Ni-N3 | 2.17020 |
| Ni-N2 | 2.14324 |
| Ni-C1 | 2.05966 |
| Ni-C2 | 2.05059 |

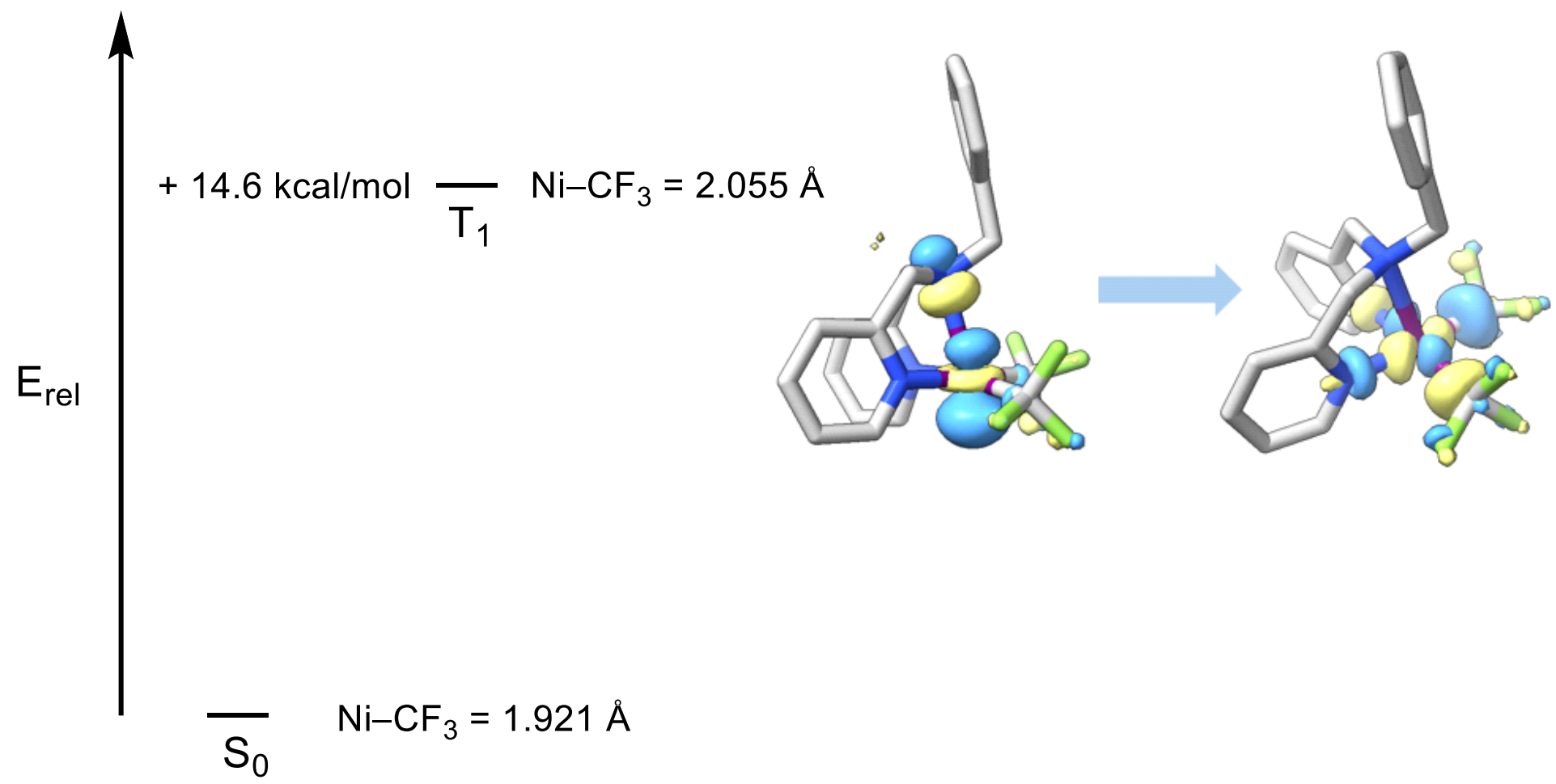


**Figure S43**: Relative energy difference between the optimized ground state and triplet state of **($^{Bn}N_3$)Ni($CF_3$)$_2$** . Ni-$CF_3$ bonds lengthen by an average of 0.134 Å.

## BDFE of Ni(II)–$CF_3$

### ($^{Fluor}N_3$)Ni($CF_3$)$_2$

# opt freq=noraman cam-b3lyp/6-31g(d,p) int=grid=ultrafine

fluorenonecomp_optfreq_ground

0 1

Sum of electronic and thermal Free Energies= -3425.233296

### •$CF_3$

# opt freq=noraman ucam-b3lyp/6-31g(d,p) int=grid=ultrafine

CF3_02

0 2

Sum of electronic and thermal Free Energies= -337.482410

**($^{Fluor}N_3$)$NiCF_3$**

# opt freq=noraman ucam-b3lyp/6-31g(d,p) int=grid=ultrafine

1fluorenonecomp_optfreq_02

0 2

Sum of electronic and thermal Free Energies= -3087.654705

[(-3087.654705)+(-337.482410)]-(-3425.233296) = 0.0961809 Hartree/particle = **60.3 kcal/mol**

## XII. Reactivity Under Visible Light Irradiation (450 nm).

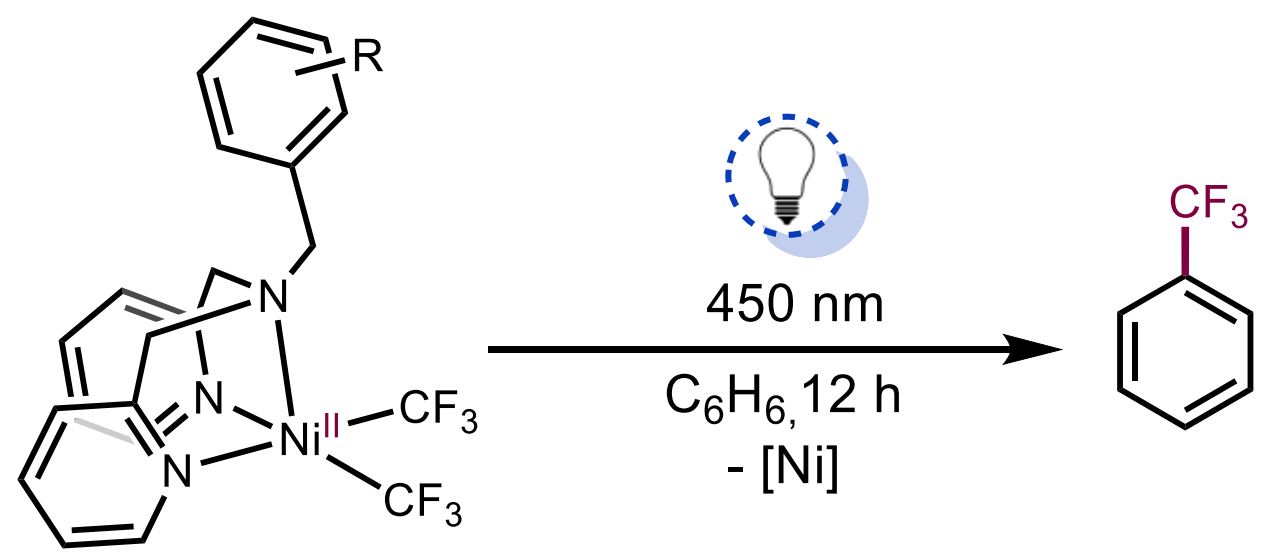


To a 20 mL scintillation vial was added 4 mL of a 0.032 mM solution of **($^{Fluor}N_3$)Ni($CF_3$)$_2$** in benzene containing hexafluorobenzene as an internal standard. A small aliquot of the homogeneous solution was transferred to a J young tube and analyzed by $^{19}F$ NMR after the addition of 5 drops of benzene-d6 (for NMR lock protocol). A stir bar was added to the reaction vial, the vial was capped and rigorously sealed with electrical tape before being removed from the glovebox and placed in the Hepatochem Lucent 360 photoreactor. The photoreactor was equipped with four 450 nm bulbs, with intensity set to 50%. A temperature of 35°C was maintained throughout the reaction with the use of an external Julabo chiller connected to the photoreactor water bath. The vials were irradiated with stirring for 12 hours before being brought back into the glovebox. An aliquot of the homogeneous reaction mixture was analyzed by $^{19}F$ NMR after the addition of 5 drops of benzene-d6 (for NMR lock protocol).

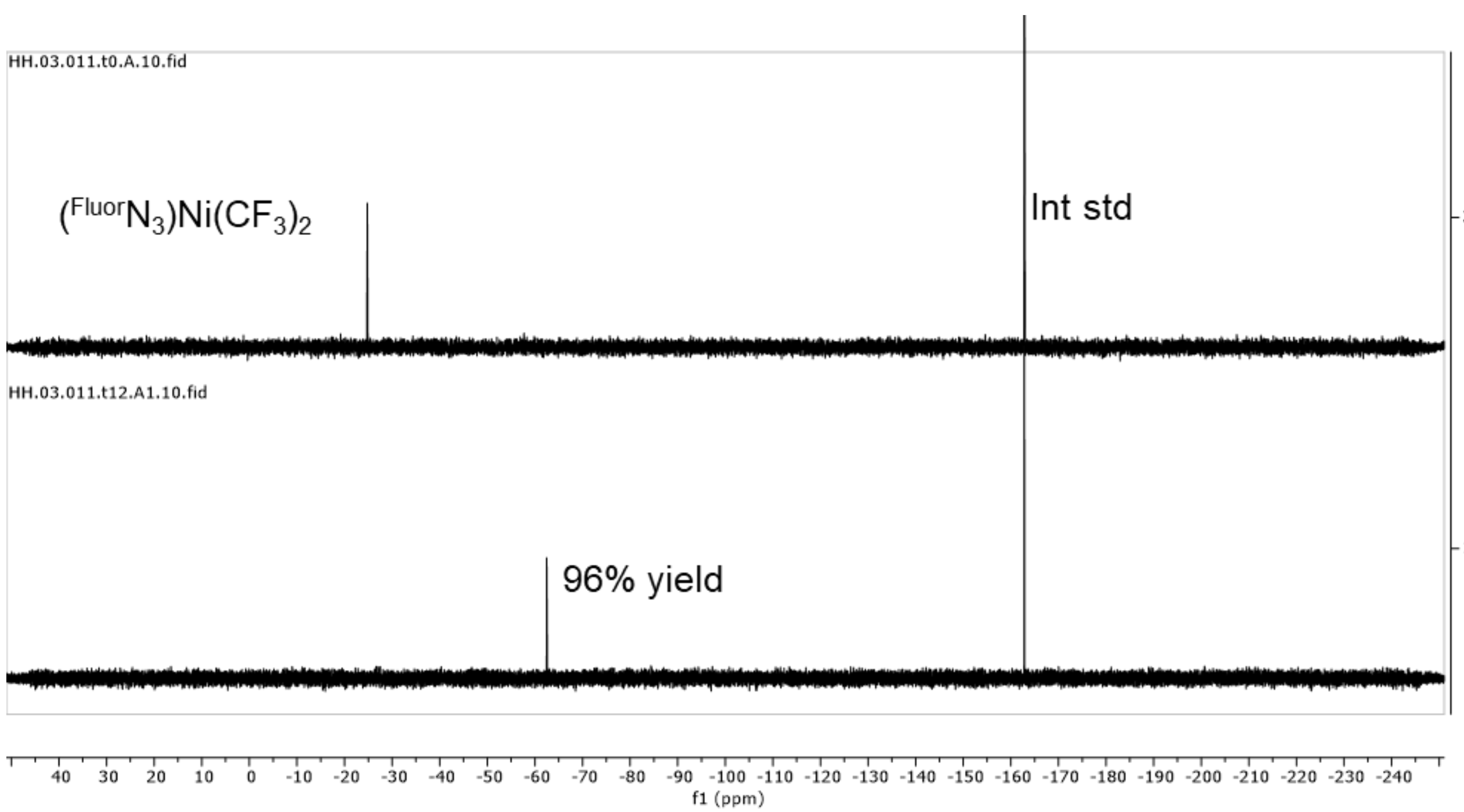

**Figure S44**: **($^{Fluor}N_3$)Ni($CF_3$)$_2$** $^{19}F$ NMR of reaction mixture at T = 0 (top) and T = 12 h (bottom), showing 96% yield of trifluorotoluene after 450 nm irradiation for 12 h in benzene, using hexafluorobenzene as an internal standard (471 MHz, $C_6D_6$).

To a 20 mL scintillation vial was added 4 mL of a 0.032 mM solution of **($^{Bn}N_3$)Ni($CF_3$)$_2$** in benzene containing hexafluorobenzene as an internal standard. A small aliquot of the homogeneous solution was transferred to a J young tube and analyzed by $^{19}F$ NMR after the addition of 5 drops of benzene-d6 (for NMR lock protocol). A stir bar was added to the reaction vial, the vial was capped and rigorously sealed with electrical tape before being removed from the glovebox and placed in the Hepatochem Lucent 360 photoreactor. The photoreactor was equipped with four 450 nm bulbs, with intensity set to 100%. A temperature of 35°C was maintained throughout the reaction with the use of an external Julabo chiller connected to the photoreactor water bath. The vials were irradiated with stirring for 12 hours before being brought back into the glovebox. An aliquot of the homogeneous reaction mixture was analyzed by $^{19}F$ NMR after the addition of 5 drops of benzene-d6 (for NMR lock protocol).

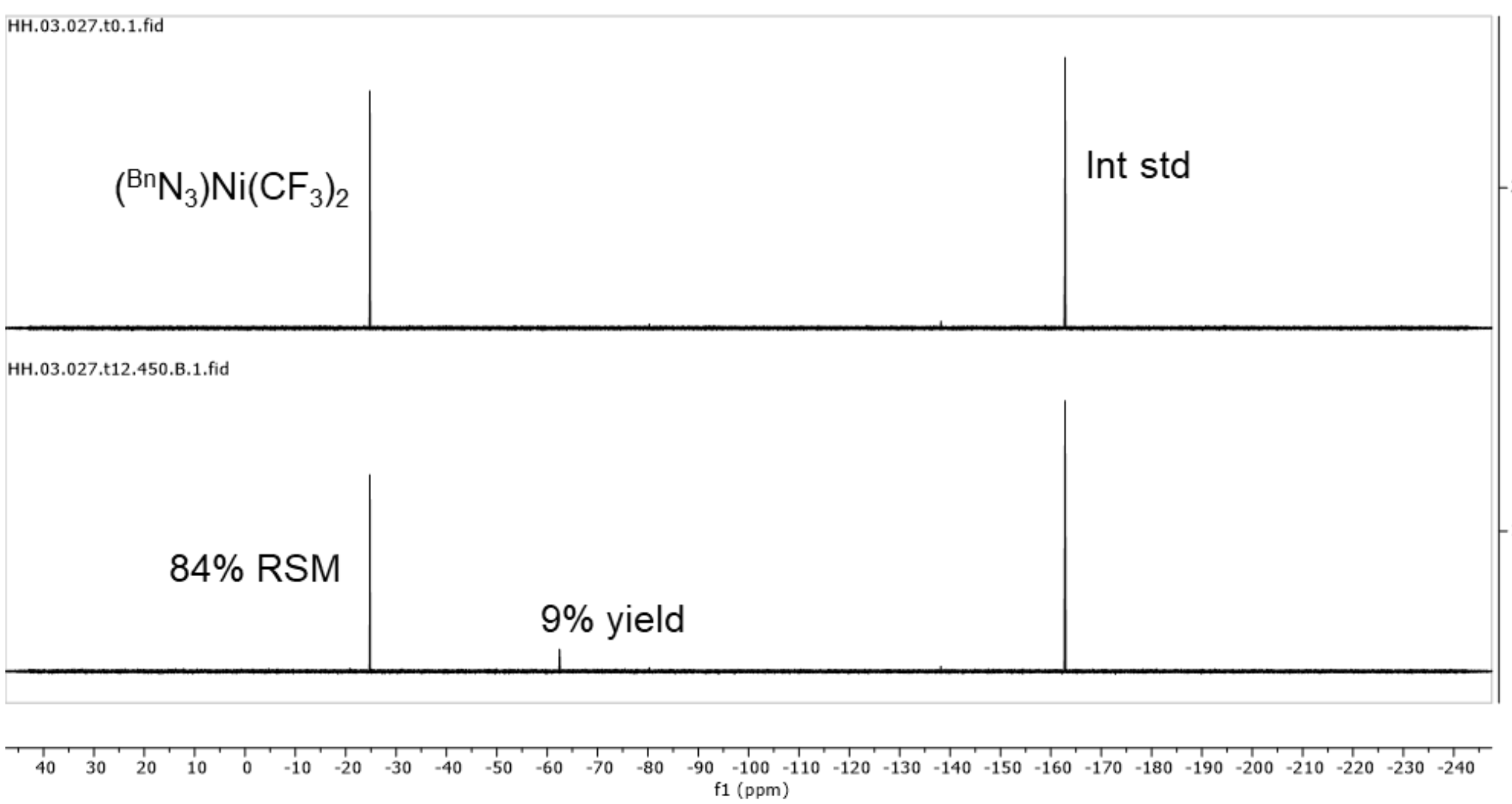


**Figure S45**: **($^{Bn}N_3$)Ni($CF_3$)$_2$** $^{19}F$ NMR of reaction mixture at T = 0 (top) and T = 12 h (bottom), showing 84% RSM and 9% yield of trifluorotoluene after 450 nm irradiation for 12 h in benzene, using hexafluorobenzene as an internal standard (471 MHz, $C_6D_6$).

## XIII. Spectroelectrochemistry.

Spectroelectrochemistry experiments on fluorenone and **($^{Fluor}N_3$)Ni($CF_3$)$_2$** were performed in an MBraun inert atmosphere (nitrogen) dry glovebox with $O_2$ < 0.5 ppm and $H_2O$ < 0.5 ppm. The acetonitrile used was dried and degassed using literature procedures.[1] All solutions were prepared in the glovebox. The spectroelectrochemistry was performed using the Pine Research Honeycomb Spectroelectrochemical Cell Kit with a platinum honeycomb electrode. A 3 mL solution of each species (0.01 M unless otherwise noted) and $NBu_4PF_6$ (0.1 M) in acetonitrile was added to the cell. Prior to controlled potential electrolysis, a UV-Visible spectrum was obtained. All UV-Visible spectra were obtained using an Agilent 8453 G1103A spectrophotometer. Controlled potential electrolysis was carried out at the specified potential with a BASi Eclipse potentiostat and a UV-Visible spectrum was obtained immediately after. All experiments were carried out at room temperature.

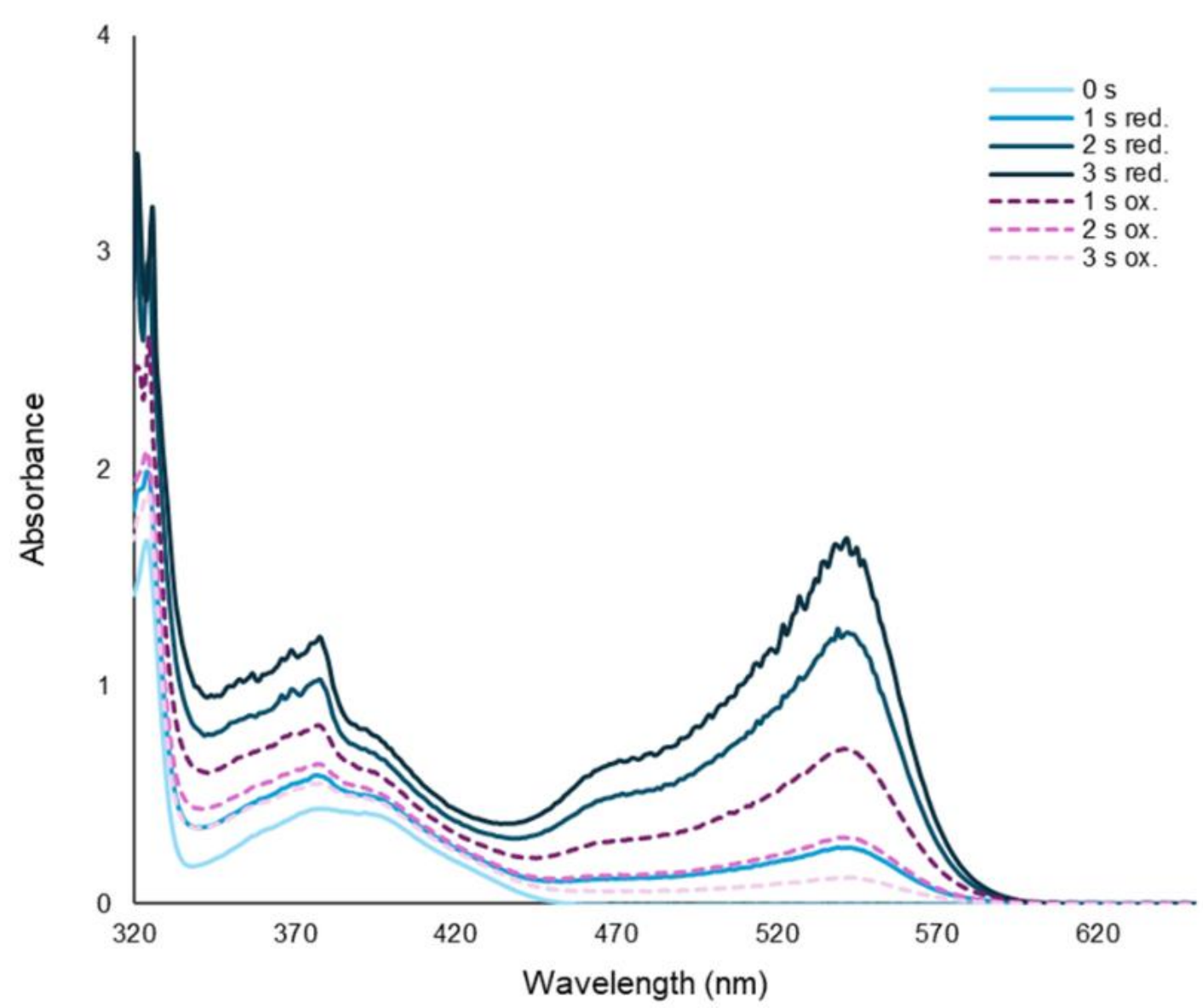


**Figure S46**: UV-Visible spectra of fluorenone (0.01M) in MeCN after *in situ* electrochemical reduction (-1800 mV), followed by oxidation (-1000 mV).

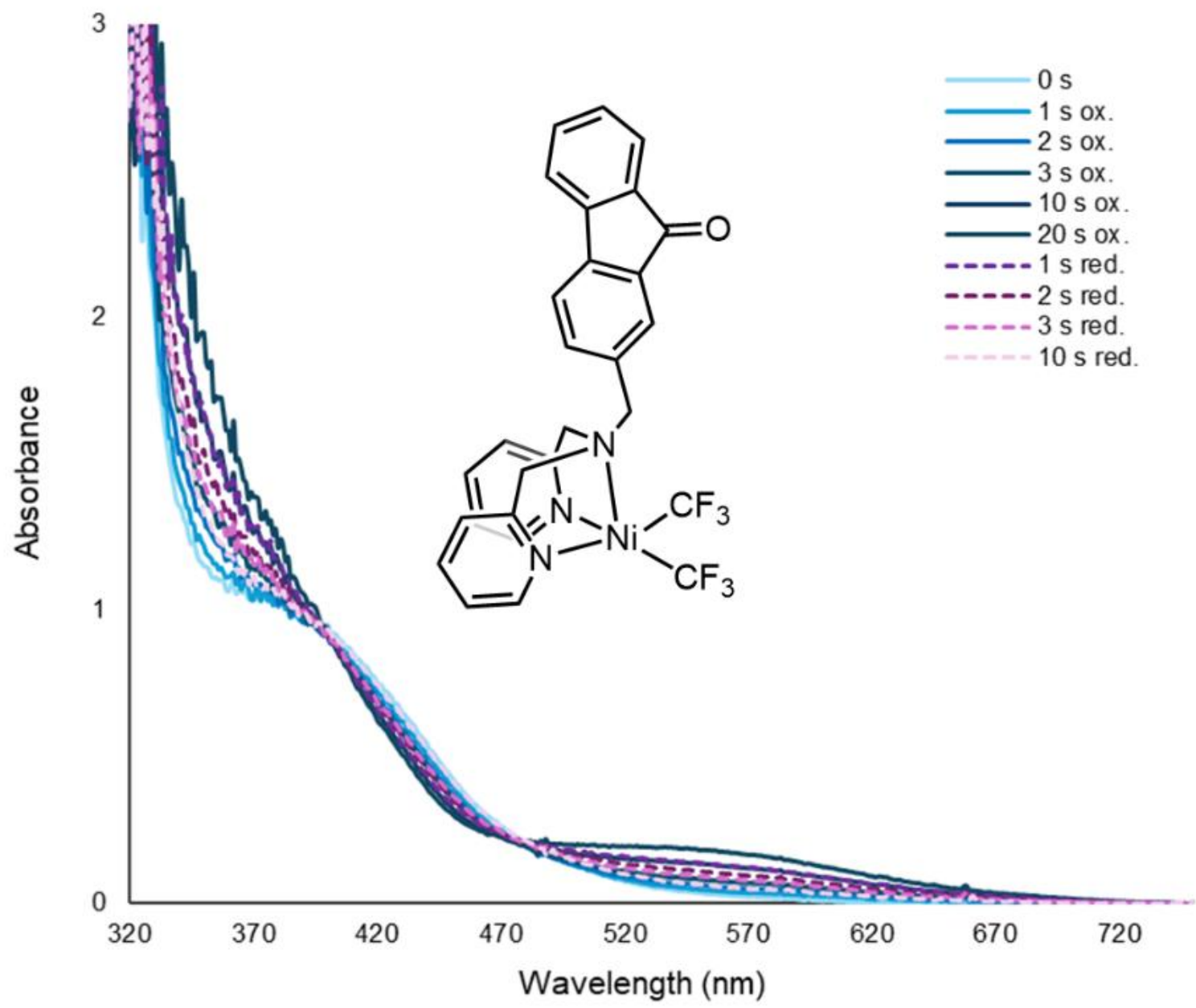


**Figure S47**: UV-Visible spectra of **($^{Fluor}N_3$)Ni($CF_3$)$_2$** (0.0075M) in MeCN after *in situ* electrochemical oxidation (120 mV), followed by reduction (-150 mV).

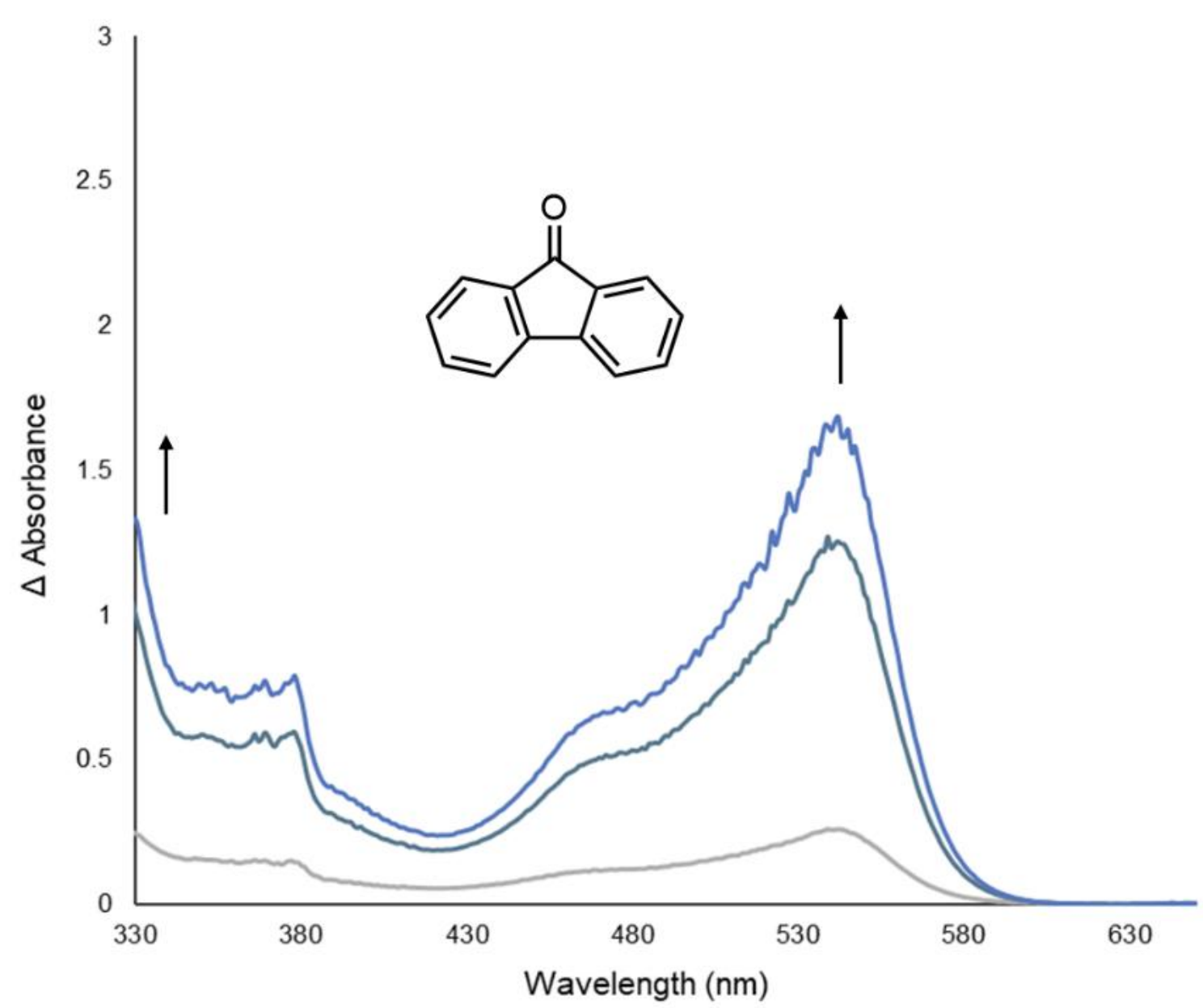


**Figure S48:** UV-Visible absorption difference spectra of fluorenone in MeCN after *in situ* electrochemical reduction (-1800 mV) over the course of 3 s.

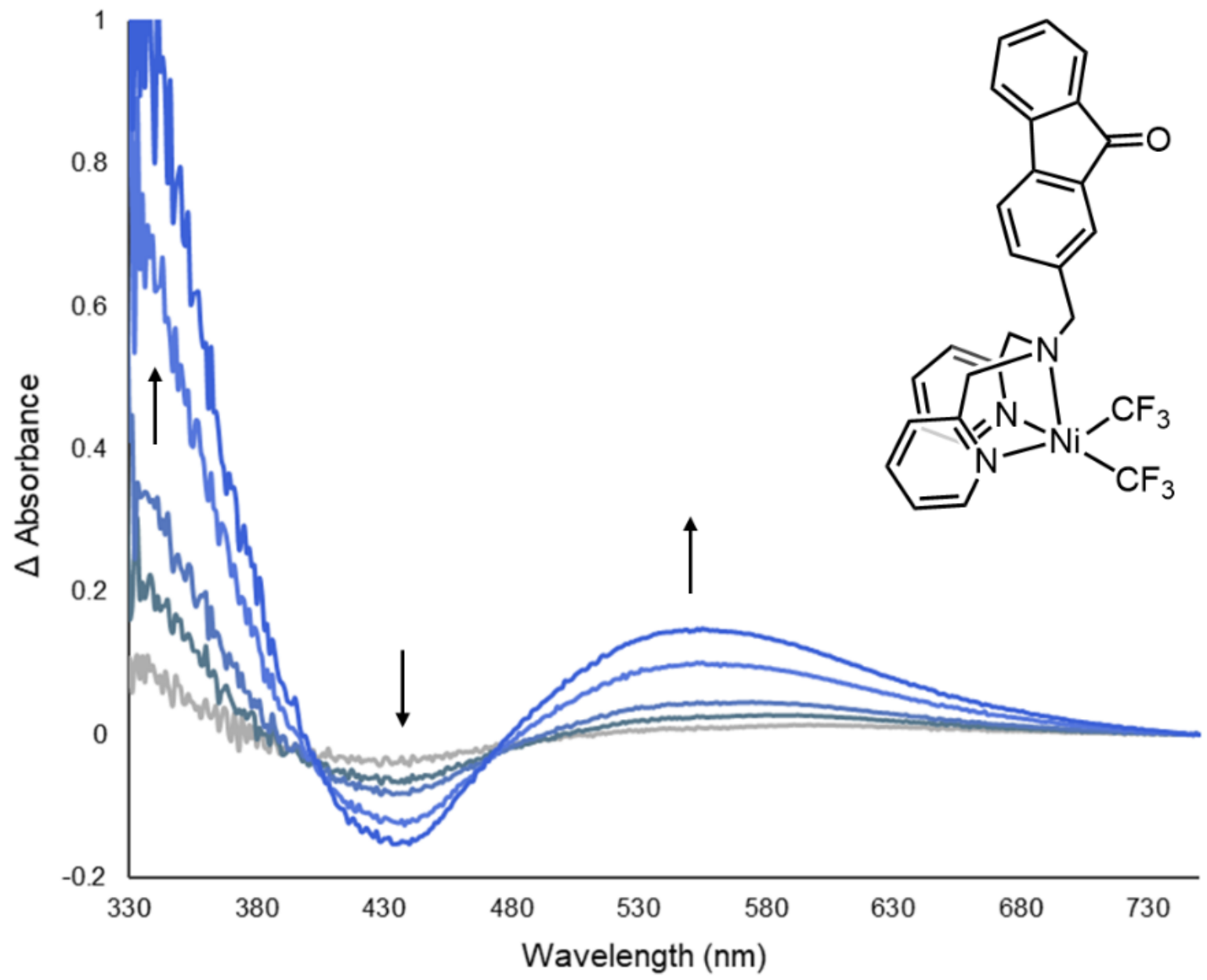


**Figure S49:** UV-Visible absorption difference spectra of **($^{Fluor}N_3$)$Ni(CF_3)_2$** (0.0075M) in MeCN after *in situ* electrochemical oxidation (120 mV) over the course of 20 s.

## XIV. Synthesis and Reactivity of Ni(III) Bis(trifluoromethyl) Complex.

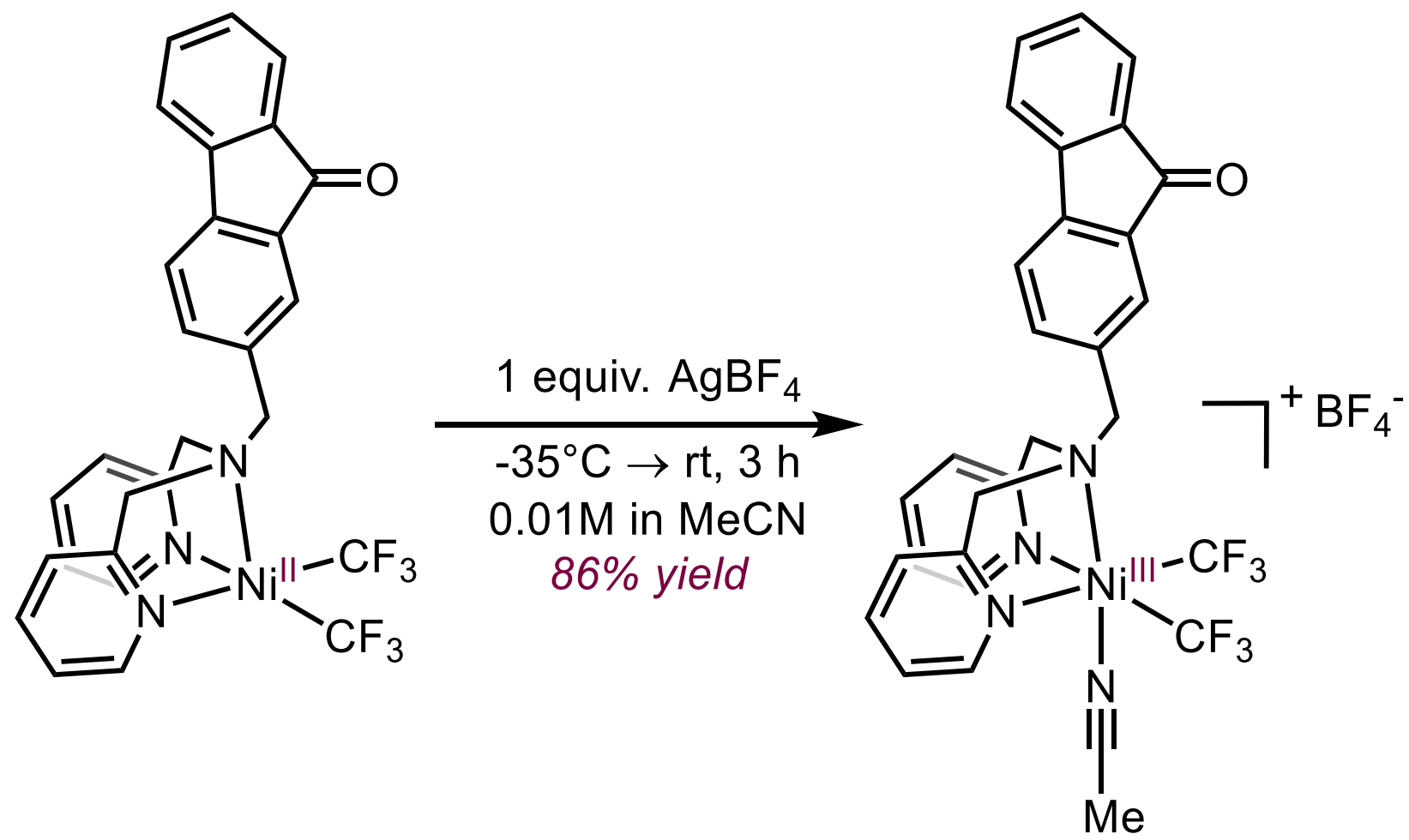


**[$^{Fluor}N_3$)Ni($CF_3$)$_2$(MeCN)]$BF_4$** was synthesized according to adapted literature procedure.[7a] In a nitrogen filled glovebox, **($^{Fluor}N_3$)Ni($CF_3$)$_2$** (0.050 g, 0.085 mmol, 1 equiv.) was added to a 20 mL scintillation vial and dissolved in 5 mL MeCN with a stir bar. In a separate vial, $AgBF_4$ (0.016g, 0.085 mmol, 1 equiv.) was dissolved 3 mL of in MeCN. Both vials were placed in the glovebox freezer (-35 °C) for 15 minutes. Immediately upon removal from the freezer, the $AgBF_4$ solution was added dropwise while stirring to the vial containing **($^{Fluor}N_3$)Ni($CF_3$)$_2$**. This was allowed to warm to room temperature and stir for 3 hours. The reaction mixture immediately turned maroon upon the addition of oxidant and continued to darken to a deep maroon-purple over the course of the reaction. The reaction mixture was filtered through a pad of celite to remove $Ag^0$. The maroon-purple solution was concentrated under reduced pressure to yield a purple solid **[$^{Fluor}N_3$)Ni($CF_3$)$_2$(MeCN)]$BF_4$** in an 86% yield.

The paramagnetic nature of the complex precluded characterization by NMR. The **[$^{Fluor}N_3$)Ni($CF_3$)$_2$(MeCN)]$BF_4$** identity was confirmed by XRD and EPR experiments. Attempts to obtain an NMR of **[$^{Fluor}N_3$)Ni($CF_3$)$_2$(MeCN)]$BF_4$** in benzene-d6 led to immediate decomposition. When the solid **[$^{Fluor}N_3$)Ni($CF_3$)$_2$(MeCN)]$BF_4$** was dissolved in acetonitrile and 5 drops of benzene-d6 were added for NMR locking protocol, $^{19}F$ NMR showed an intractable mixture immediately after the addition of benzene-d6. Within 1 hour, the solution changed in color from deep purple to yellow. Analysis of the decomposed sample by $^{19}F$ NMR after 12 hours revealed an intractable mixture signifying complex decomposition (Figure S51). When the solid **[$^{Fluor}N_3$)Ni($CF_3$)$_2$(MeCN)]$BF_4$** was dissolved in a 1:1 v/v mixture of acetonitrile and benzene, a similar yellow color was observed, and $^{19}F$ NMR revealed an intractable mixture.

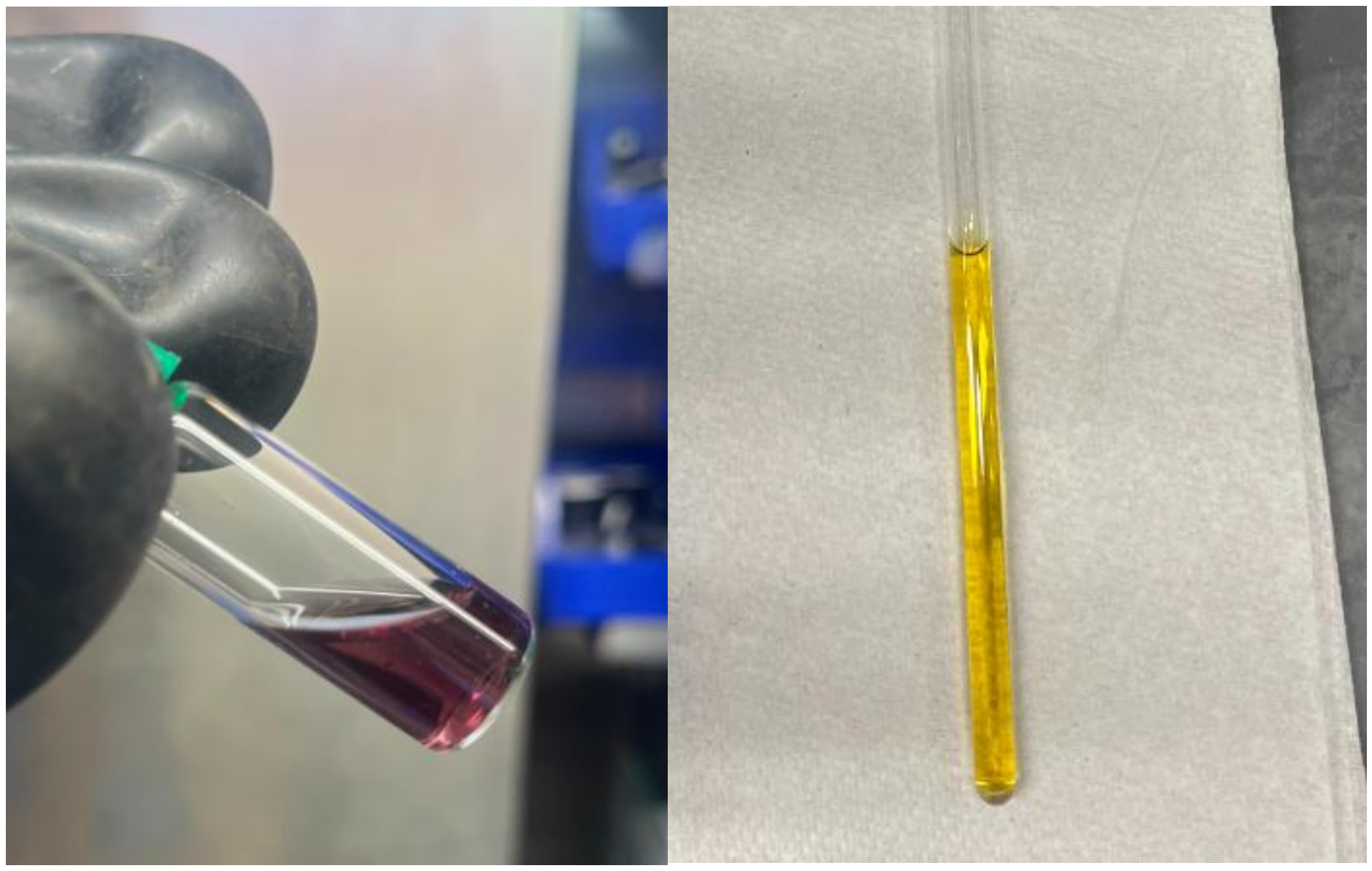

**Figure S50:** (Left) [$^{Fluor}$**N$_3$)Ni(CF$_3$)$_2$(MeCN)]BF$_4$** in acetonitrile; (right) [$^{Fluor}$**N$_3$)Ni(CF$_3$)$_2$(MeCN)]BF$_4$** in a J young tube dissolved in acetonitrile with 5 drops of $C_6D_6$ after 12 hours at room temperature.

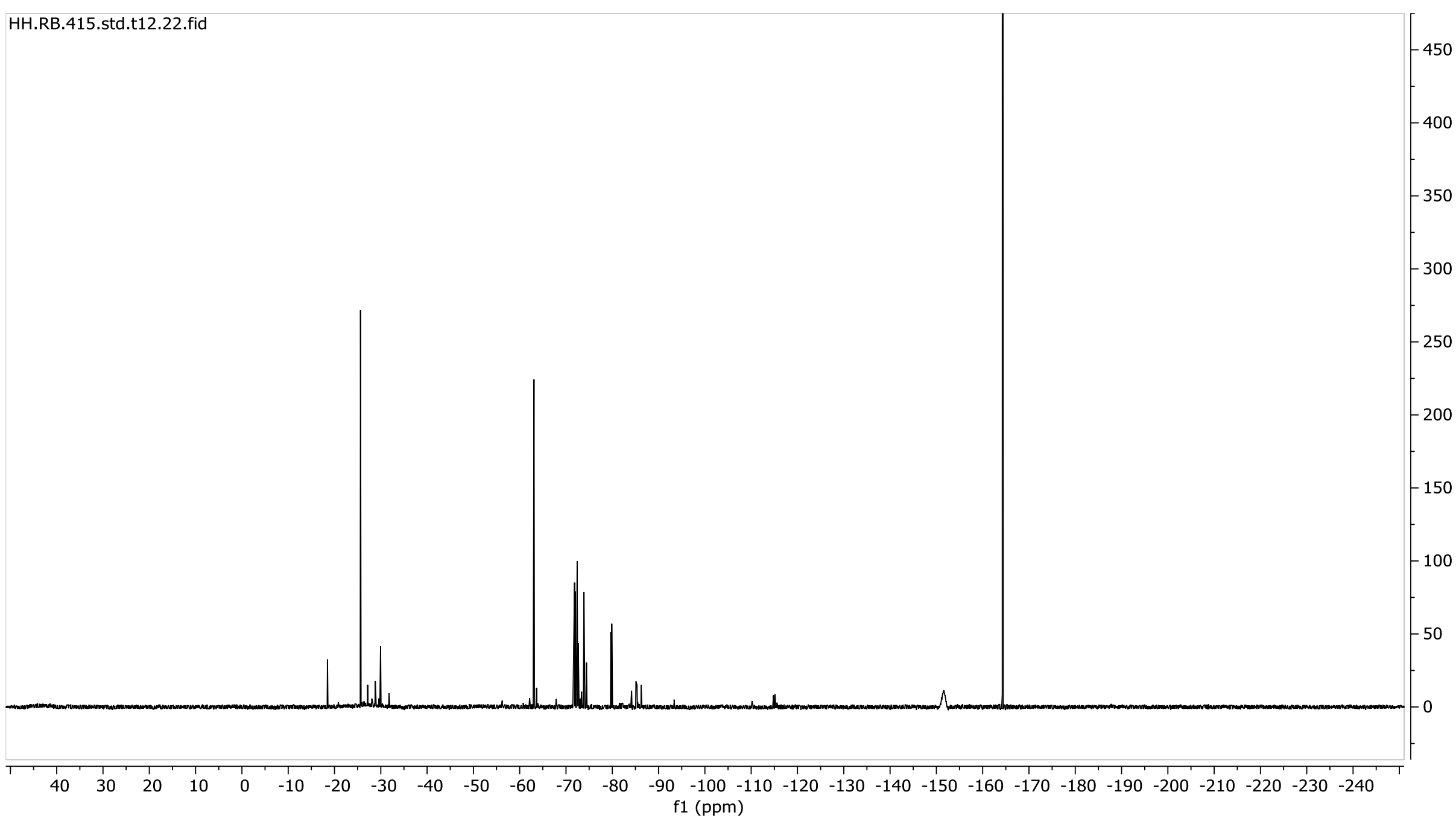


**Figure S51**: **[$^{Fluor}$N$_3$)Ni(CF$_3$)$_2$(MeCN)]BF$_4$** in a solution of MeCN with 5 drops of $C_6D_6$ after sitting at room temperature for 12 hours. $^{19}$F NMR shows an intractable mixture of fluorinated products upon exposure to benzene (471 MHz, $C_6D_6$).

While the mixture resulting from exposure of [$^{Fluor}$**N$_3$)Ni(CF$_3$)$_2$(MeCN)]BF$_4$** to benzene was largely intractable (see above discussion), storage of the mixture at -35 °C yielded the formation of light

yellow crystals suitable for XRD analysis. The identity of the yellow crystals was determined to be the post bond-homolysis nickel complex, **[($^{Fluor}N_3$)Ni($CF_3$)]$BF_4$**.

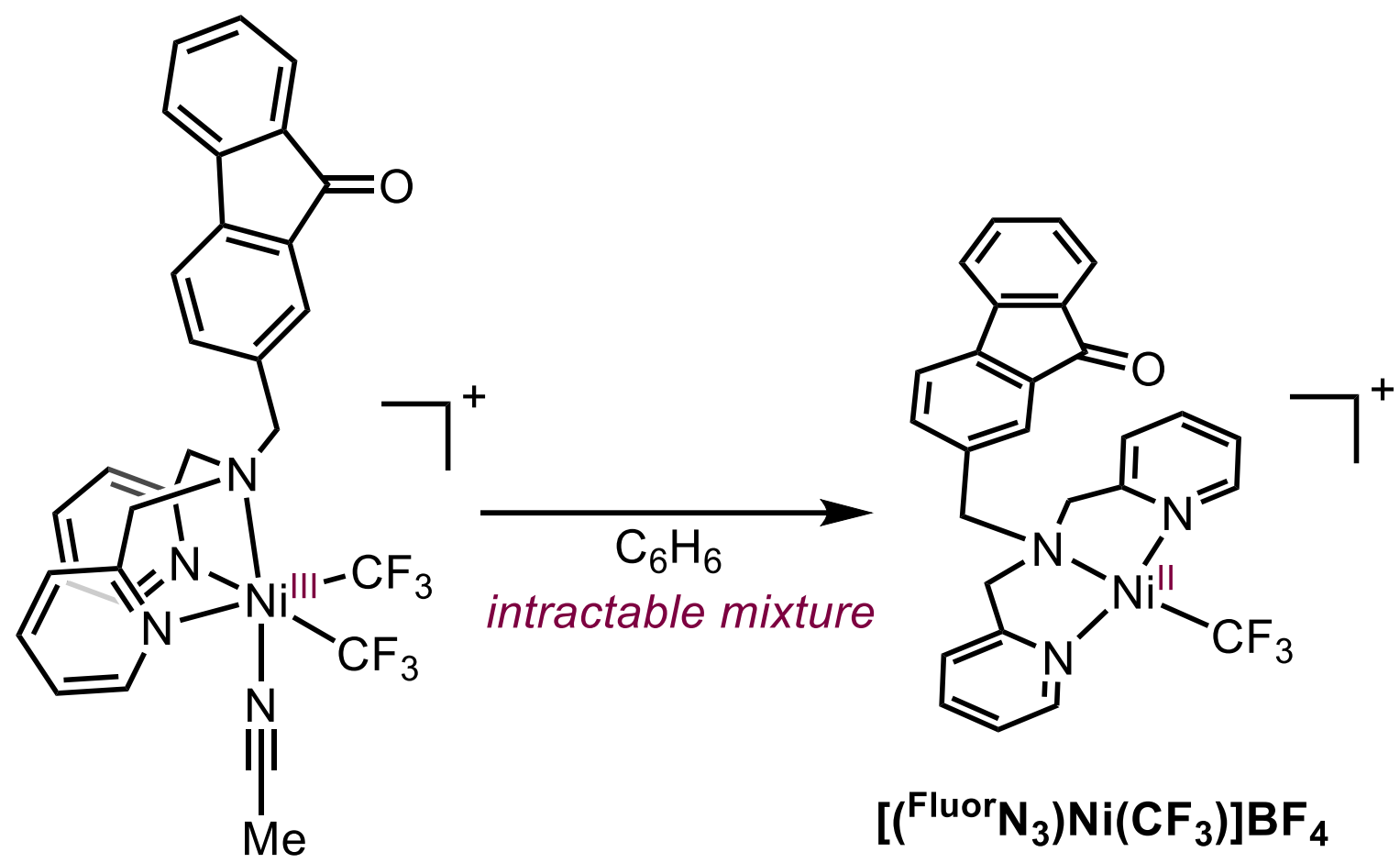


**[($^{Fluor}N_3$)Ni($CF_3$)]$BF_4$**

## XV. EPR Experiments.

Continuous wave EPR spectra were recorded at cryogenic temperatures on an X-band Bruker EMXPlus spectrometer equipped with an EMX standard resonator and a Bruker PremiumX microwave bridge. The spectra were simulated using EasySpin for MATLAB.[20]

**Procedure for EPR detection of [$^{Fluor}N_3$)Ni($CF_3$)$_2$(MeCN)]$BF_4$ :** A 4 mL vial was charged with 0.004 g (0.005 mmol) of [$^{Fluor}N_3$)Ni($CF_3$)$_2$(MeCN)]$BF_4$ and a 1:1 v/v mixture of acetonitrile:toluene. 300 μL of this solution was transferred to an EPR tube and equipped with a septum-cap and rigorously sealed with black electrical tape. The sample was then flash-frozen in liquid nitrogen until analysis. The experimental parameters were: microwave frequency = 9.6 GHz, field range = 240-360 mT, 750 points.

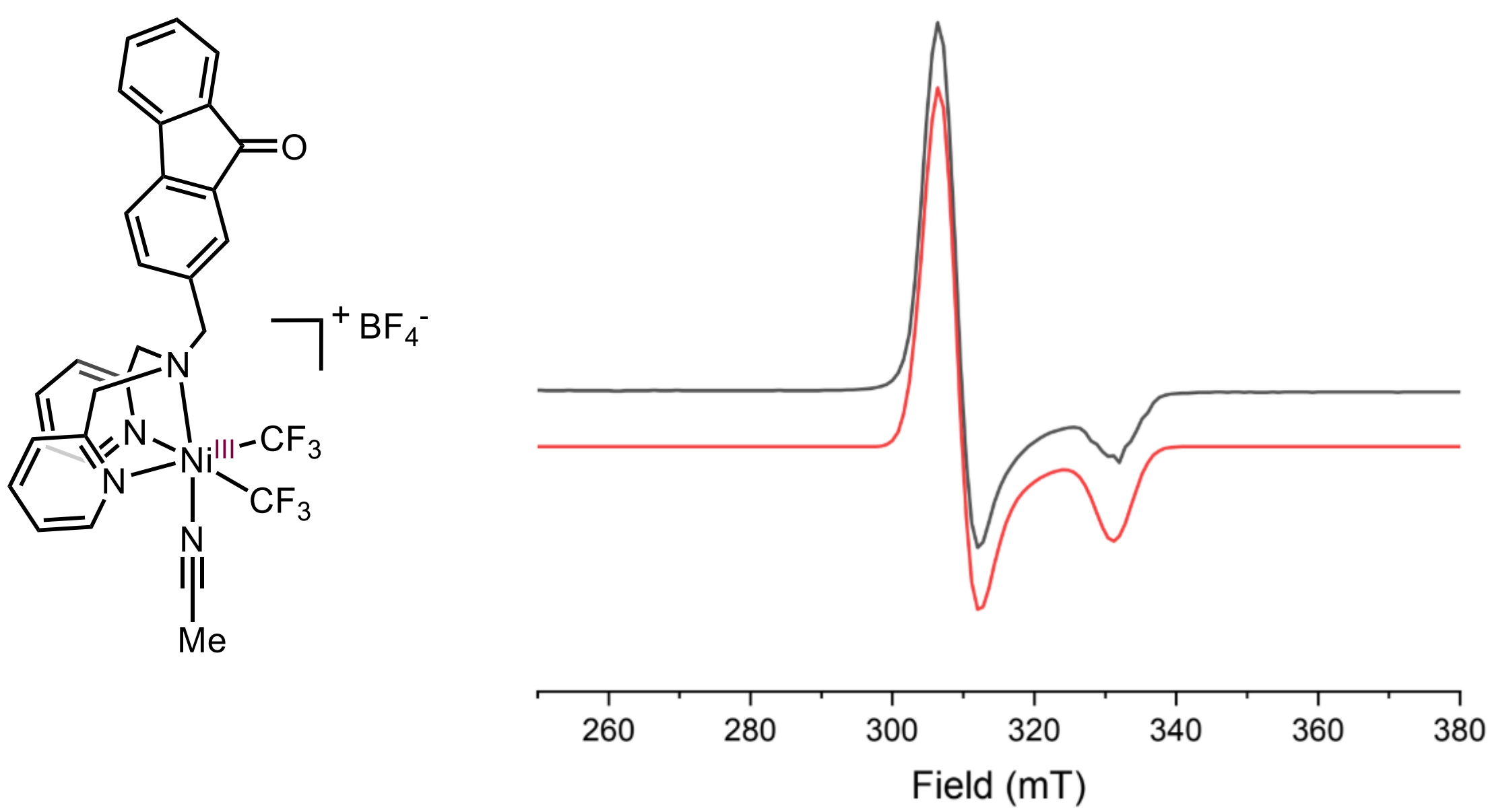


**Figure S52**: EPR spectrum of [$^{Fluor}N_3$)Ni($CF_3$)$_2$(MeCN)]$BF_4$ (top/black) and the simulated spectrum (bottom/red). Fit using the following parameters: $g_x$ = 2.30, $g_y$ = 2.30, $g_z$ = 2.27.

## XIV. Reactivity Under Red Light Irradiation.

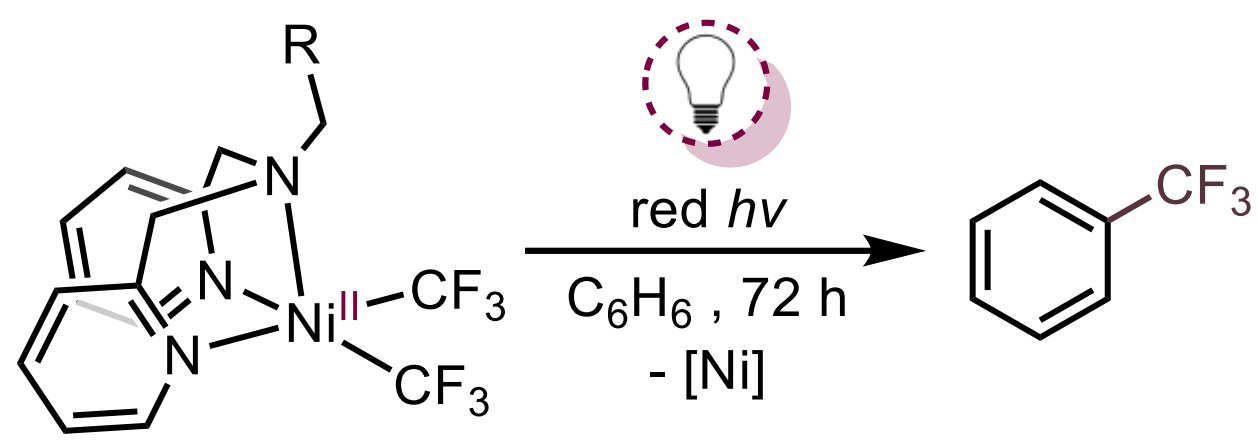


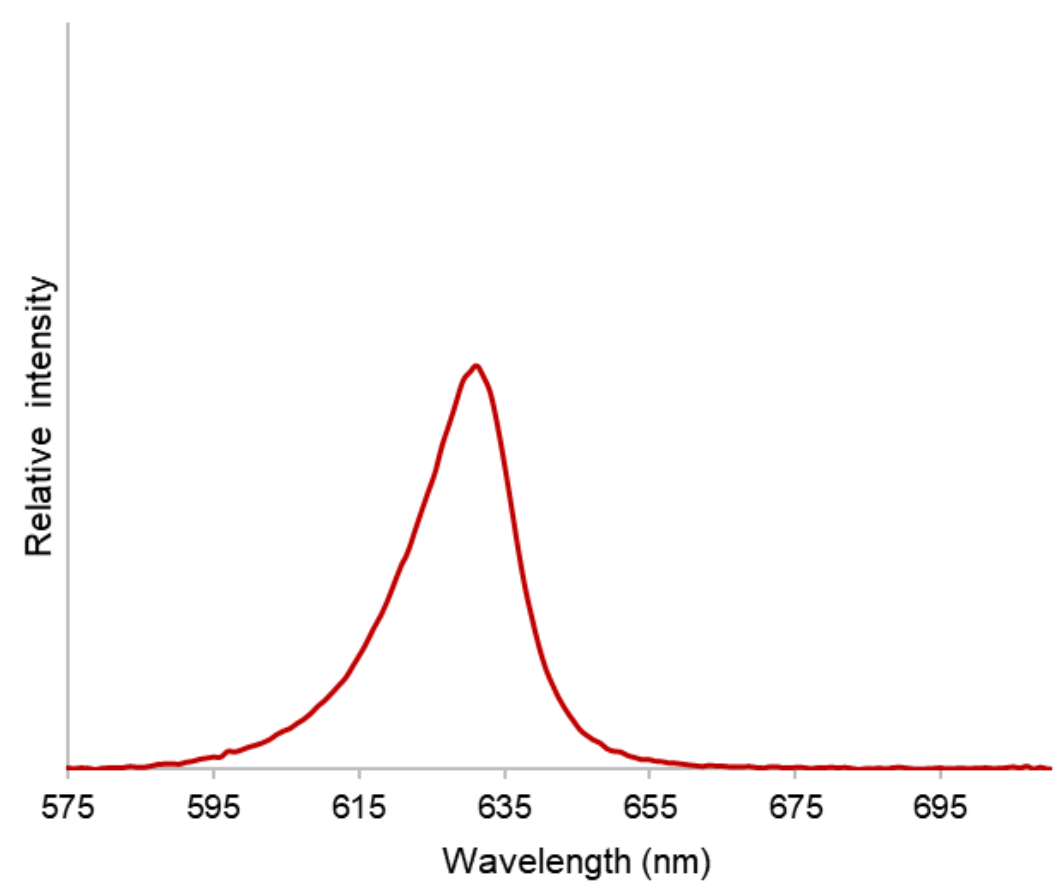


**Figure S53**: Emission profile of red light.

To a 20 mL scintillation vial was added 4 mL of a 0.032 mM solution of **($^{Fluor}N_3$)Ni($CF_3$)$_2$** in benzene containing hexafluorobenzene as an internal standard. A small aliquot of the homogeneous solution was analyzed by $^{19}F$ NMR after the addition of 5 drops of benzene-d6 (for NMR lock protocol). A stir bar was added to the reaction vial, the vial was capped and rigorously sealed with electrical tape before being removed from the glovebox and placed in front of a red LED lamp on a stir plate. The vials were irradiated and the temperature held constant with the use of two external fans. The vials were irradiated with stirring for 72 hours before being brought back into the glovebox. An aliquot of the homogeneous reaction mixture was analyzed by $^{19}F$ NMR after the addition of 5 drops of benzene-d6 (for NMR lock protocol).

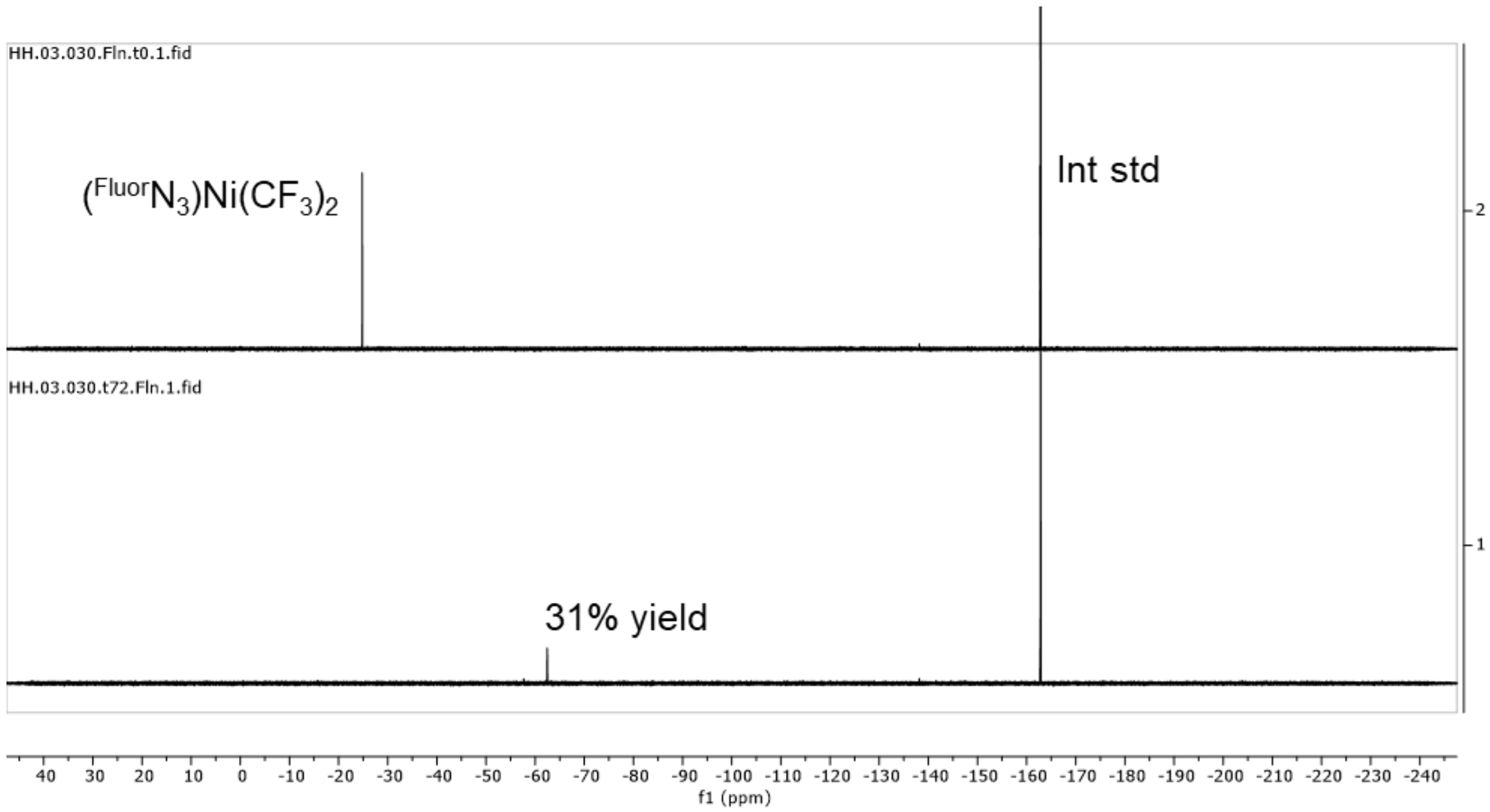


**Figure S54**: $^{19}$F NMR of reaction mixture at T = 0 (top) and T = 72 h (bottom), showing 31% yield of trifluorotoluene after red light irradiation for 72 h in benzene, using hexafluorobenzene as an internal standard (471 MHz, $C_6D_6$).

To a 20 mL scintillation vial was added 4 mL of a 0.032 mM solution of **($^{Bn}N_3$)Ni($CF_3$)$_2$** in benzene containing hexafluorobenzene as an internal standard. A small aliquot of the homogeneous solution was analyzed by $^{19}$F NMR after the addition of 5 drops of benzene-d6 (for NMR lock protocol). A stir bar was added to the reaction vial, the vial was capped and rigorously sealed with electrical tape before being removed from the glovebox and placed in front of a red LED lamp on a stir plate. The vials were irradiated and the temperature held constant with the use of two external fans. The vials were irradiated with stirring for 72 hours before being brought back into the glovebox. An aliquot of the homogeneous reaction mixture was analyzed by $^{19}$F NMR after the addition of 5 drops of benzene-d6 (for NMR lock protocol).

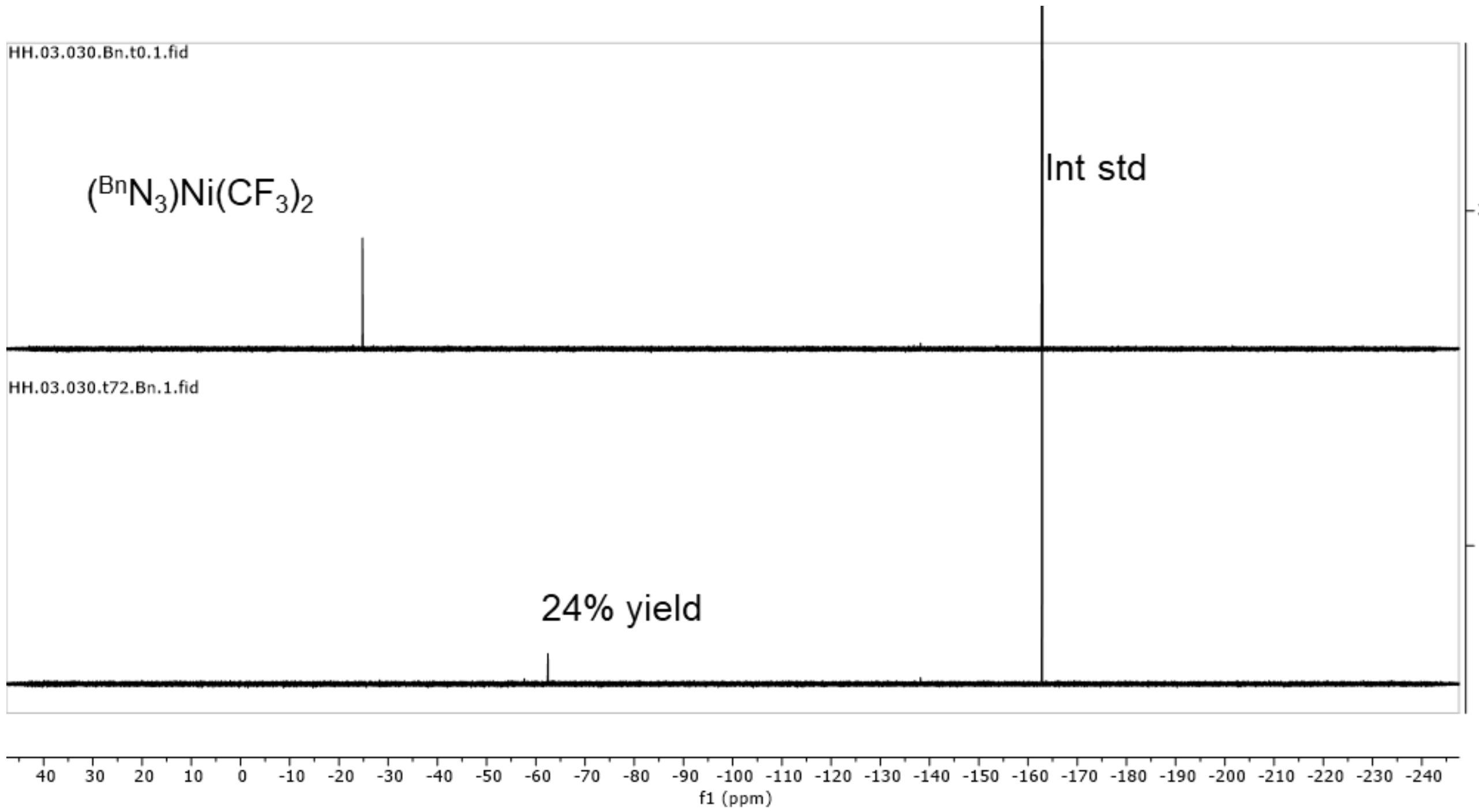


**Figure S55:** $^{19}F$ NMR of reaction mixture at T = 0 (top) and T = 72 h (bottom), showing 31% yield of trifluorotoluene after red light irradiation for 72 h in benzene, using hexafluorobenzene as an internal standard (471 MHz, $C_6D_6$).

## XVII. Evaluation of Chemical Oxidation.

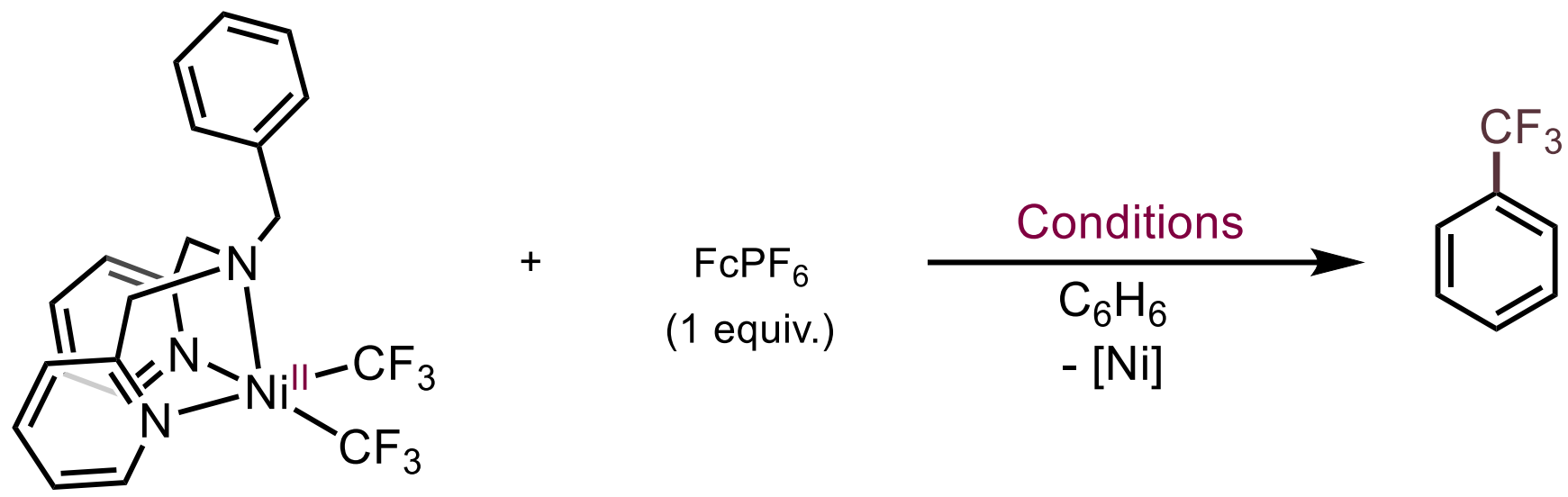


To a 20 mL scintillation vial was added **($^{Bn}N_3$)Ni($CF_3$)$_2$** (0.005 g, 0.00987 mmol, 1 equiv.) and 3 mL of benzene, followed by 50 µL of a hexafluorobenzene in benzene solution. A small aliquot of the homogeneous solution was transferred to a J young tube and analyzed by $^{19}F$ NMR after the addition of 5 drops of benzene-d6 (for NMR lock protocol). To a separate vial was added $FcPF_6$ (0.003 g, 0.00987 mmol, 1 equiv.) and 1 mL of benzene. The oxidant solution was added dropwise to the **($^{Bn}N_3$)Ni($CF_3$)$_2$** solution. A small aliquot of the homogeneous solution was transferred to a J young tube and analyzed by $^{19}F$ NMR after the addition of 5 drops of benzene-d6 (for NMR lock protocol). After sitting for 18 hours at room temperature in the dark, the reaction mixture was analyzed by $^{19}F$ NMR (Figure S56B, revealing an 8% yield of trifluorotoluene). The J young containing the reaction mixture was placed in the Hepatochem Lucent 360 photoreactor equipped with four 405 nm bulbs, with intensity set to 20%. A temperature of 35°C was maintained throughout the reaction with the use of an external Julabo chiller connected to the photoreactor water bath. The tube was irradiated for 12 hours before being analyzed by $^{19}F$ NMR.

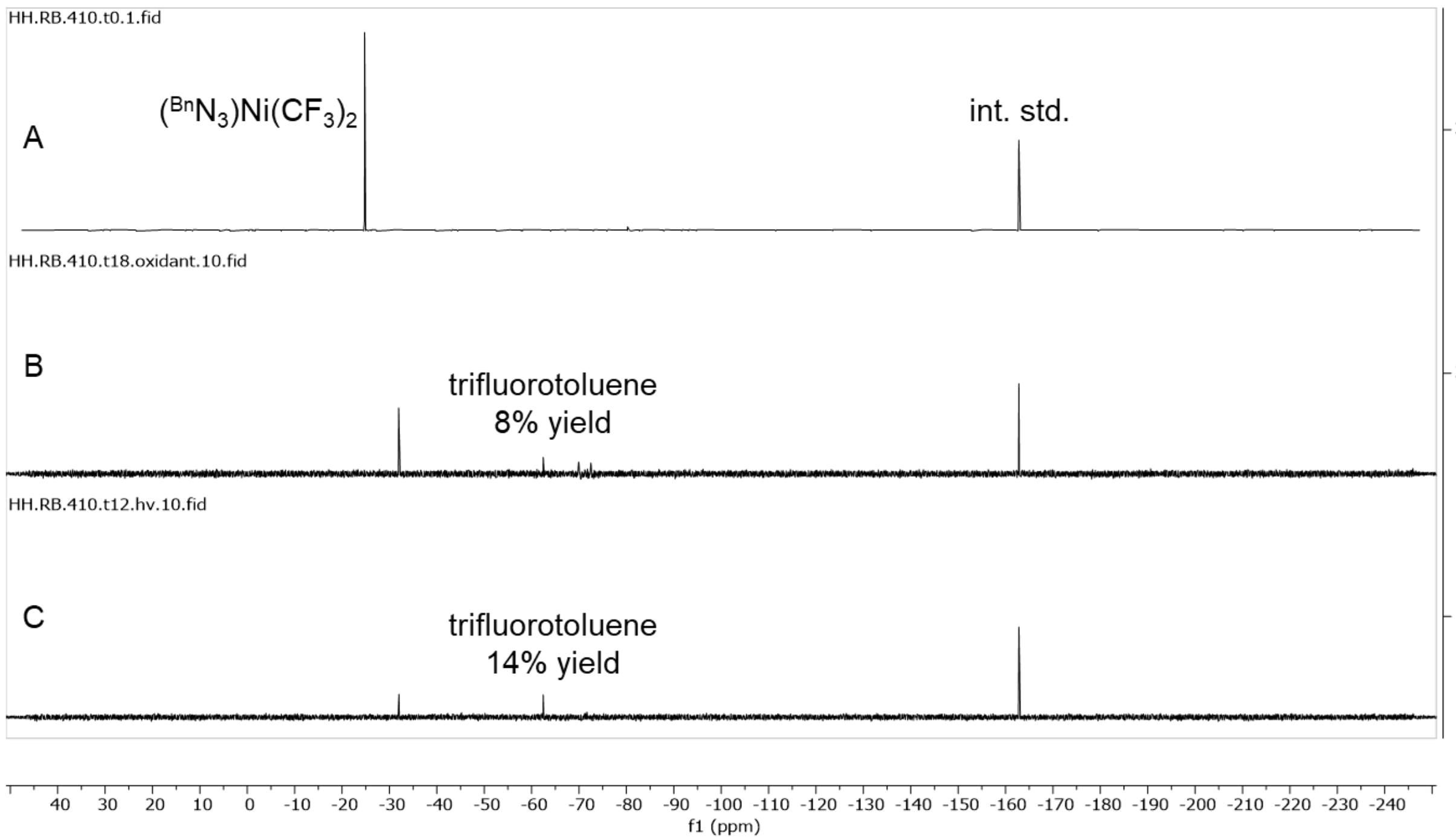


**Figure S56**: **($^{Bn}N_3$)Ni($CF_3$)$_2$** (A) $^{19}$F NMR of reaction mixture at T = 0. (B) $^{19}$F NMR of reaction mixture after treatment with 1 equiv. of $FcPF_6$ after sitting at room temperature for 18 hours in the dark. (C) $^{19}$F NMR of the reaction mixture (B) after irradiation with 405 nm light for 12 hours, showing a 14% yield of trifluorotoluene (471 MHz, $C_6D_6$).

## XVIII. Attempts at Characterization Post-Irradiation.

After the photoreactions at 405 nm and 450 nm with **($^{Fluor}N_3$)Ni($CF_3$)$_2$** in benzene, the samples were concentrated to dryness to yield a light beige powder. Poor solubility in $C_6D_6$ led to poor $^1H$ NMR resolution. The post-*hv* [Ni] complex exhibits diamagnetic resonances in $^1H$ NMR that cannot be assigned to free ligand or the **($^{Fluor}N_3$)Ni($CF_3$)$_2$** starting complex. There are no signals in the paramagnetic $^1H$ NMR. There are no signals in the $^{19}F$ NMR spectrum, indicating that the post-*hv* [Ni] complex does not bear any $CF_3$ ligands. This is further supported by the > 50% yields obtained for the photoirradiation of the complex, indicating the homolysis of both $CF_3$ ligands. Attempts at growing XRD quality single crystals were unsuccessful despite screening a wide range of solvents and crystallization methods. The intact free ligand **($^{Fluor}N_3$)** was detected via mass spectrometry (**HRMS** (ESI): calc for $C_{26}H_{21}N_3O$ [M+H]+ 392.1757, found 392.1772) after decomposition of the post-*hv* [Ni] solid by passing through silica.

## XIX. NMR Spectra.

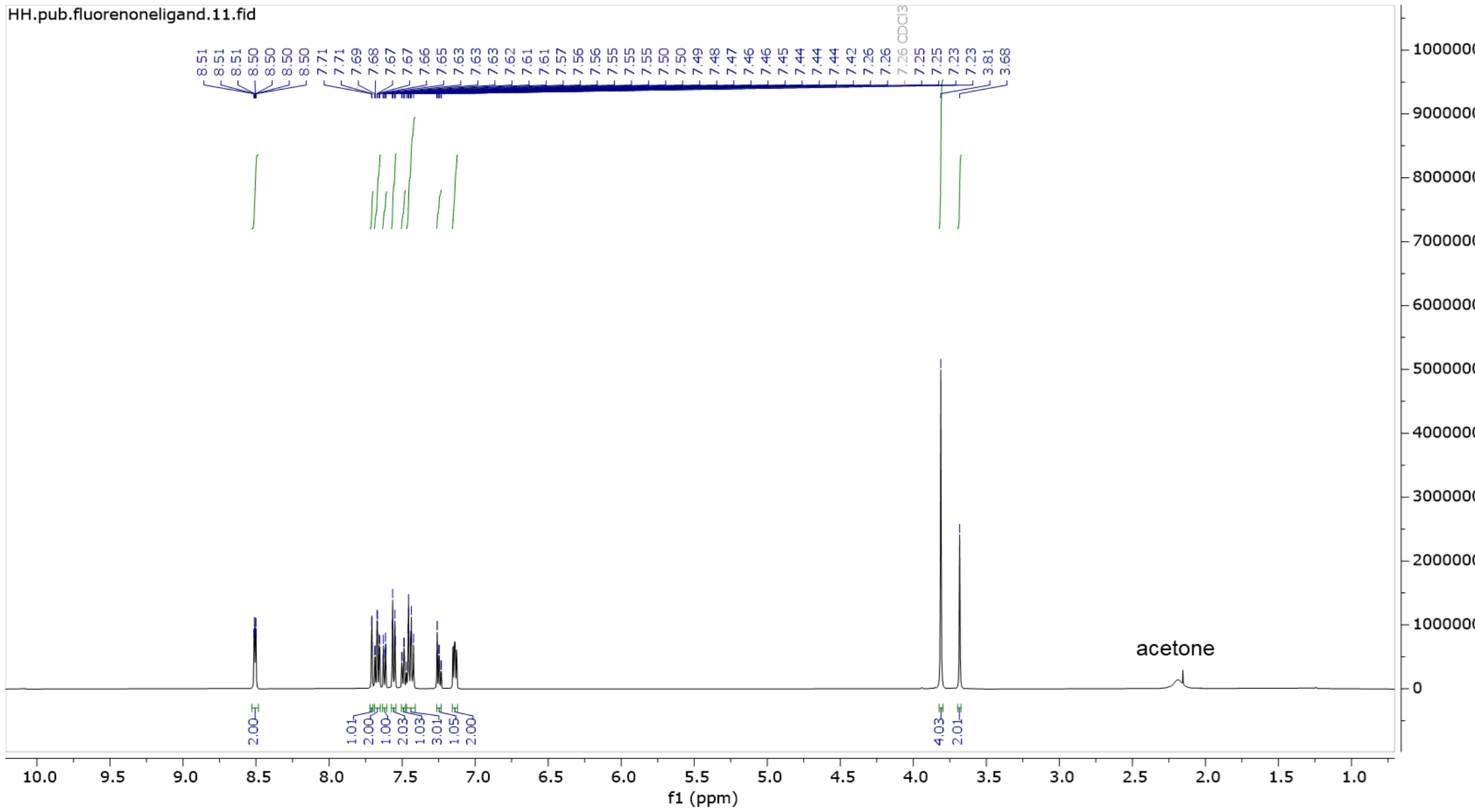


**Figure S57**: $^{1}$H NMR spectrum of $^{\mathbf{Fluor}}\mathbf{N_3}$ (500 MHz, $CDCl_3$).

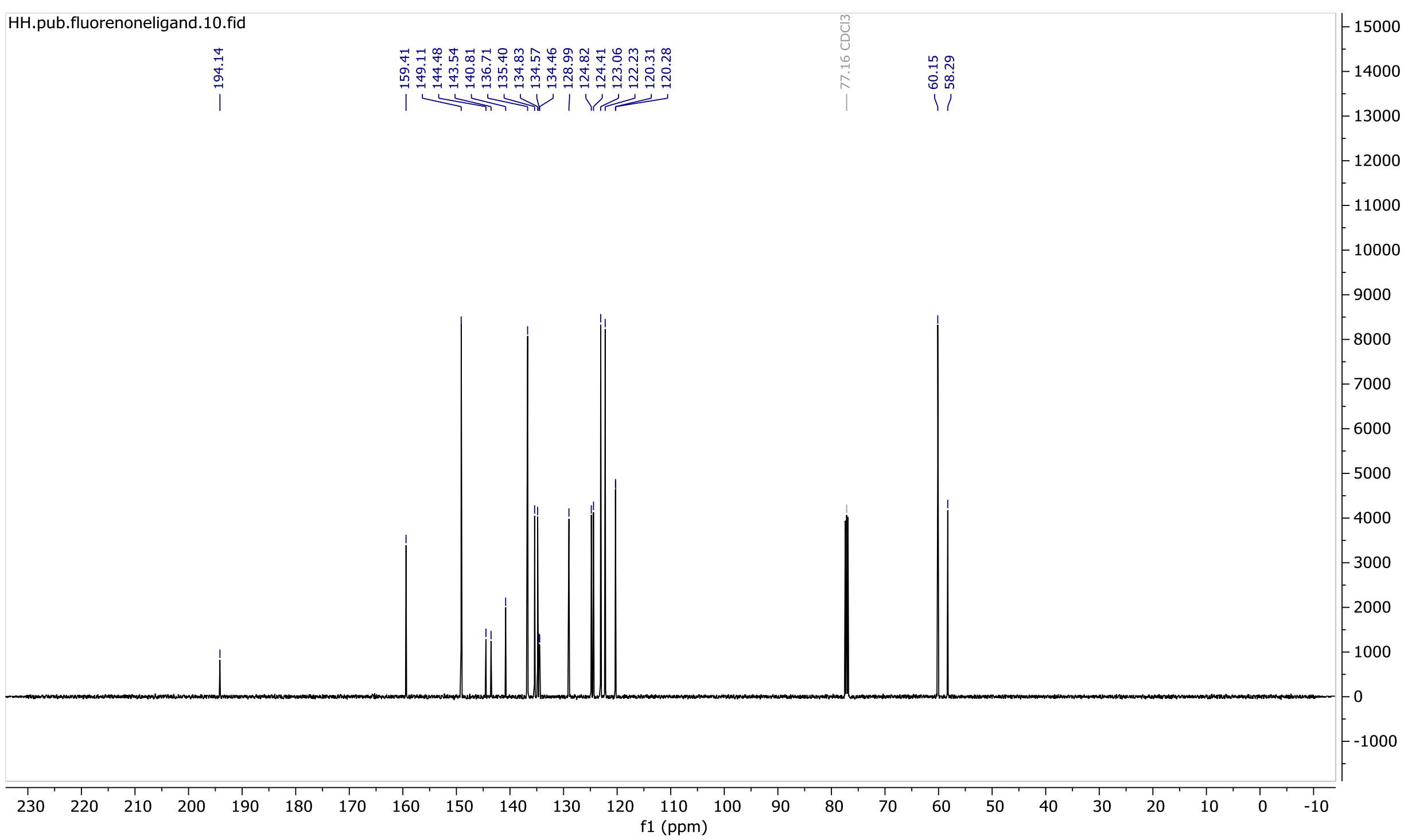


**Figure S58**: $^{13}$C NMR spectrum of $^{\mathbf{Fluor}}\mathbf{N_3}$ (126 MHz, $CDCl_3$).

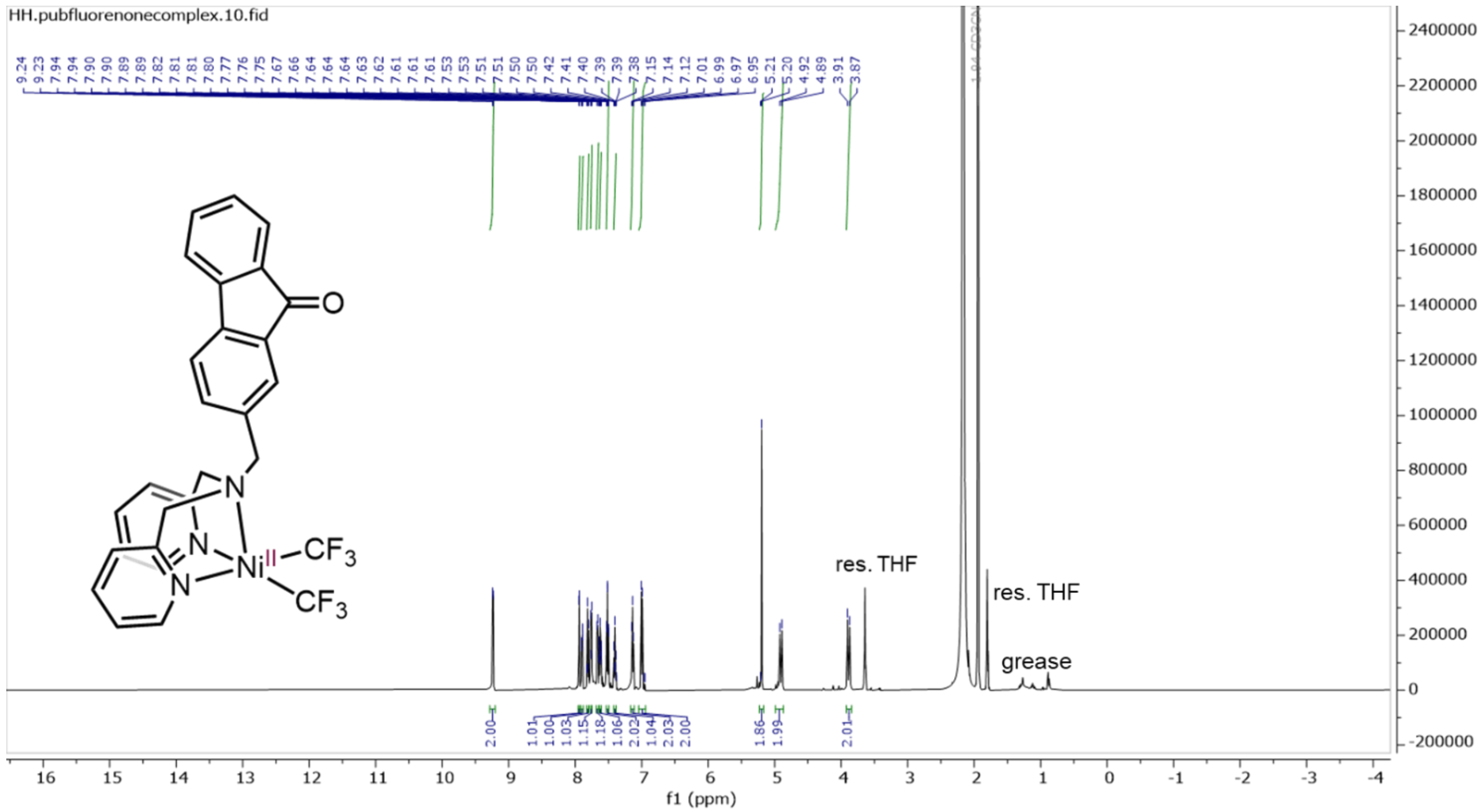


**Figure S59**:$^{1}$H NMR spectrum of **($^{Fluor}$N$_3$)Ni(CF$_3$)$_2$** (500 MHz, $CD_3CN$).

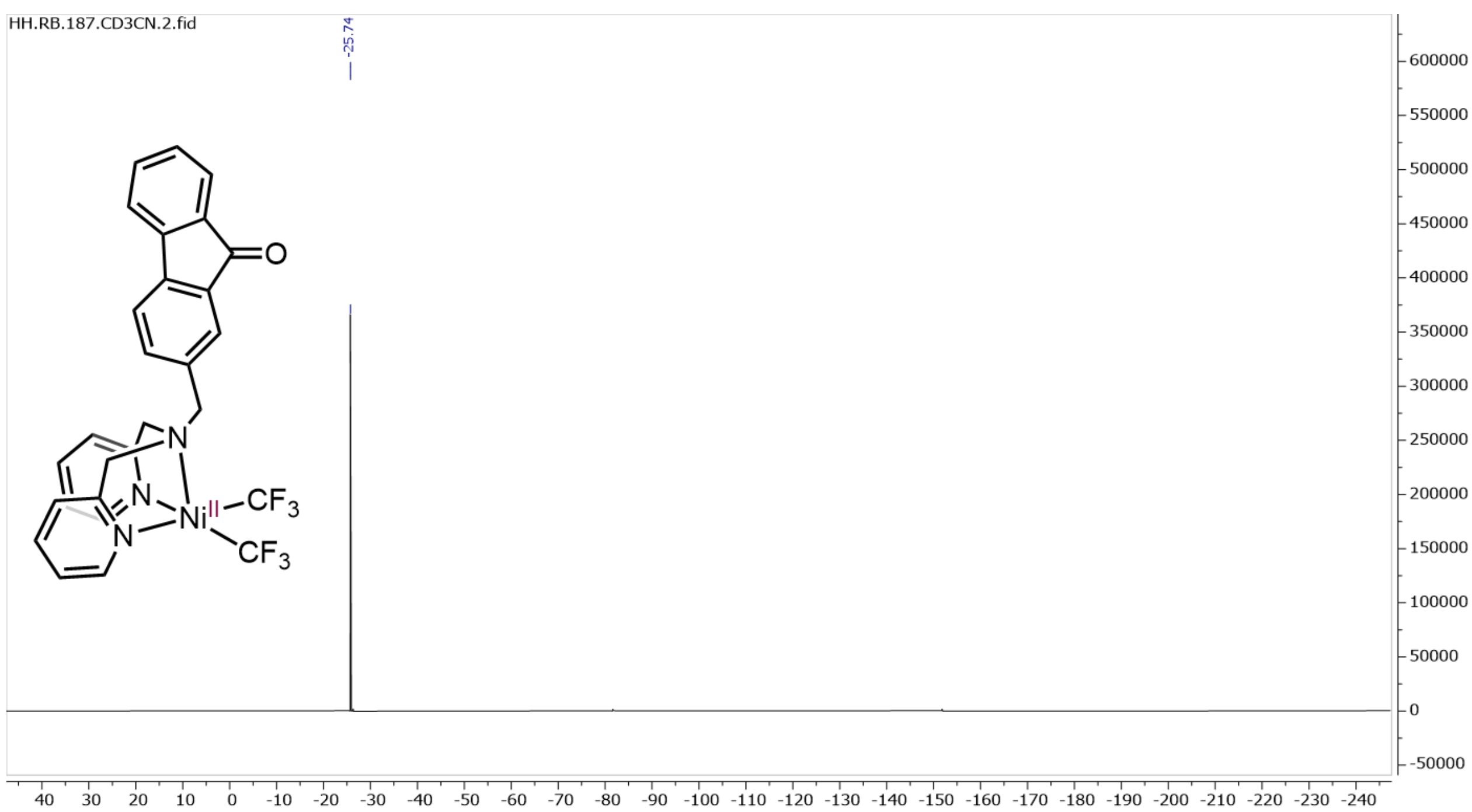


**Figure S60**: $^{19}$F NMR spectrum of **($^{Fluor}$N$_3$)Ni(CF$_3$)$_2$** (471 MHz, $CD_3CN$)

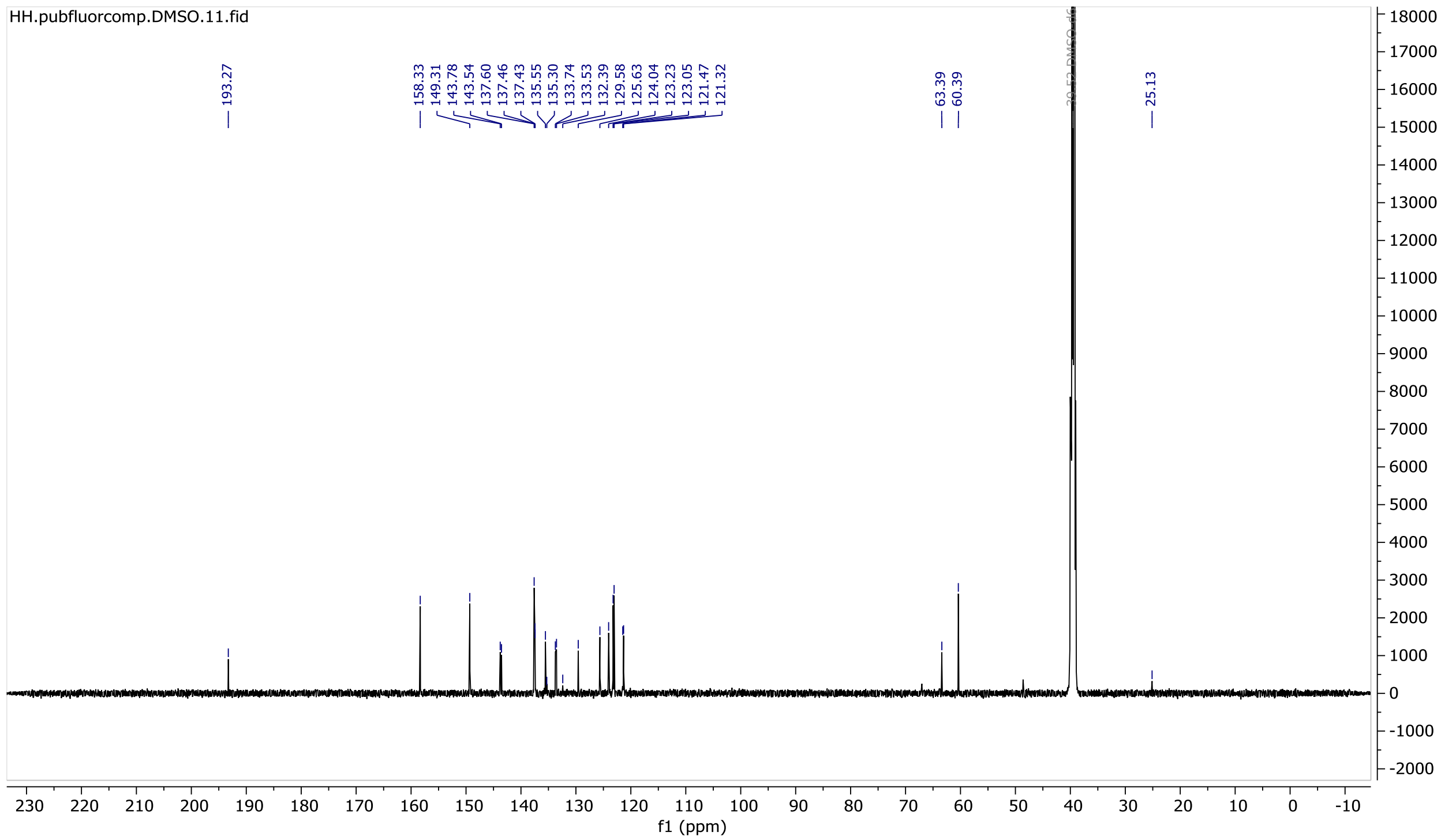


**Figure S61**: $^{13}$C NMR spectrum of **($^{Fluor}$N$_3$)Ni(CF$_3$)$_2$** (126 MHz, DMSO$_{d6}$).

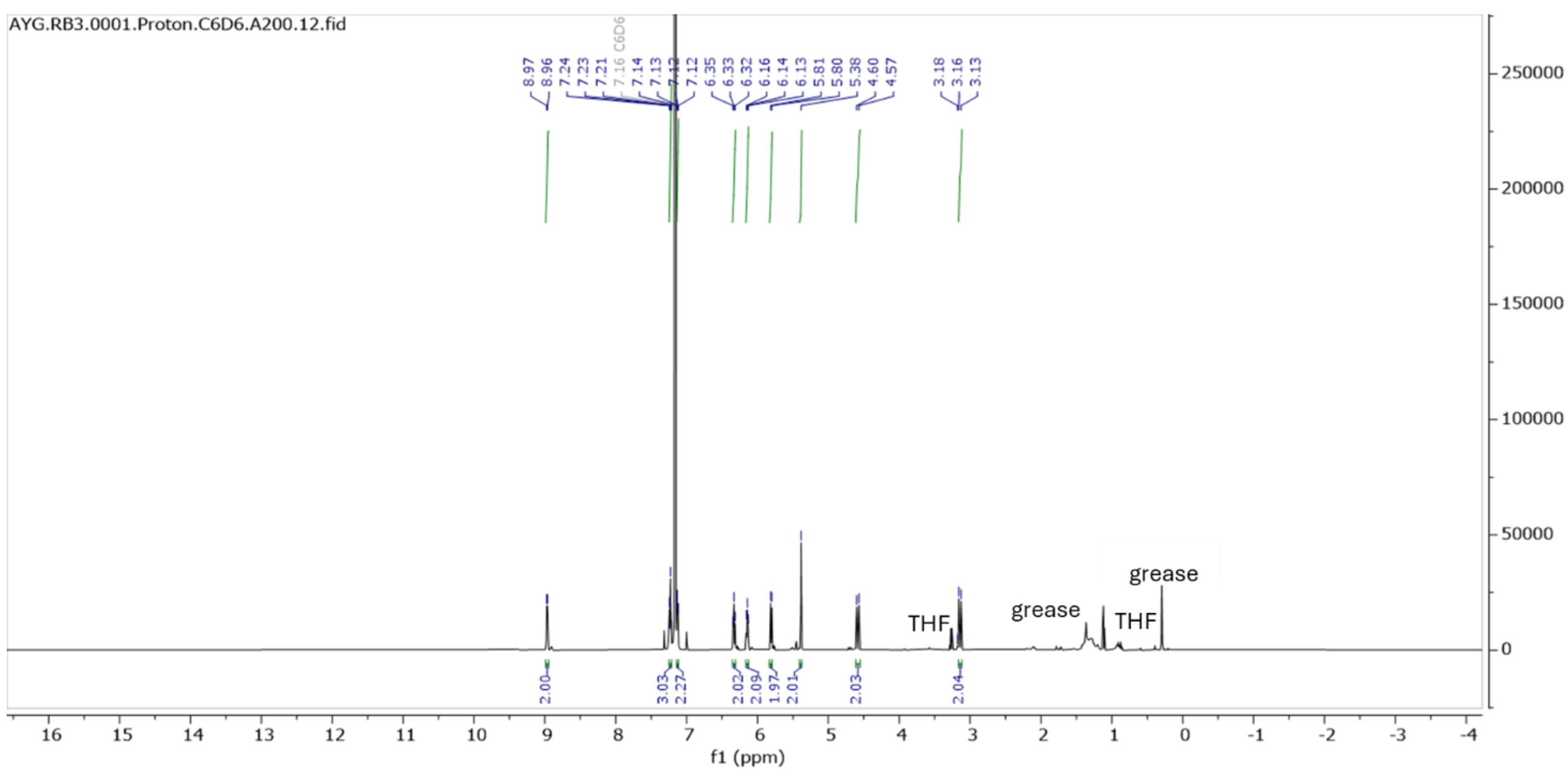


**Figure S62**: $^{1}$H NMR spectrum of **($^{Bn}$N$_3$)Ni(CF$_3$)$_2$** (500 MHz, $C_6D_6$).

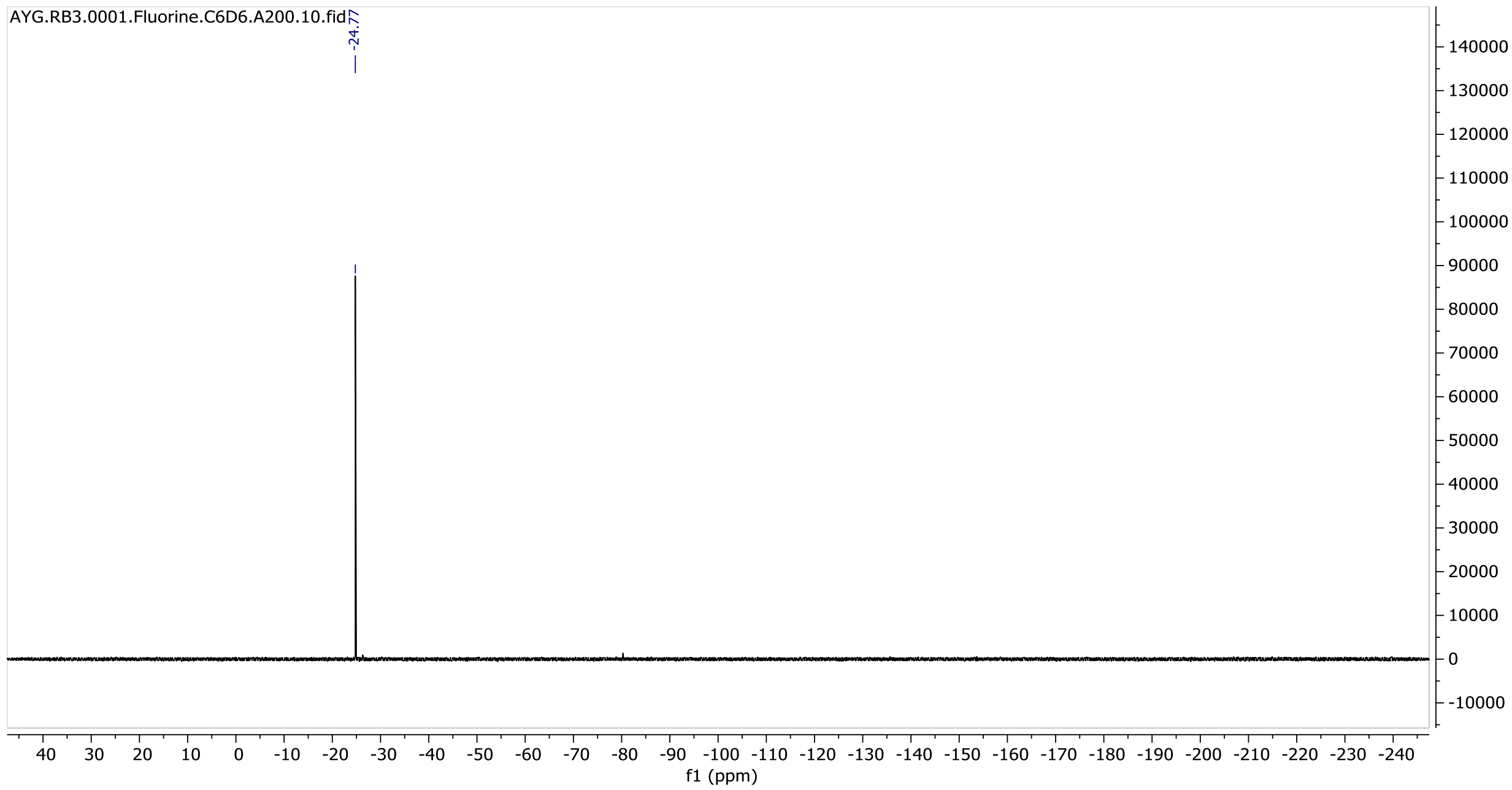


**Figure S63**: $^{19}F$ NMR spectrum of **($^{Bn}N_3$)Ni($CF_3$)$_2$** (471 MHz, $C_6D_6$).

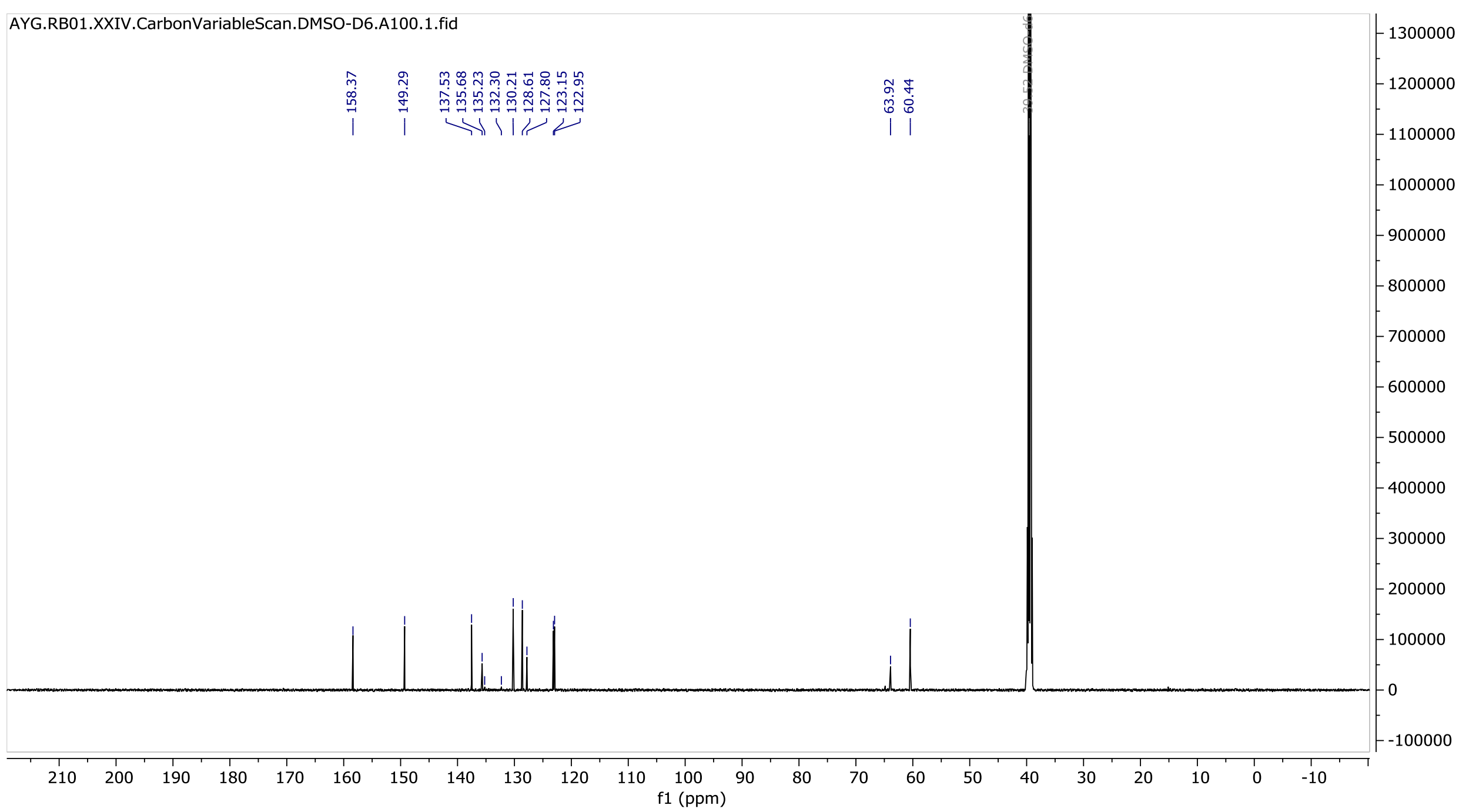


**Figure S64**: $^{13}C$ NMR spectrum of **($^{Bn}N_3$)Ni($CF_3$)$_2$** (126 MHz, $DMSO_{d6}$).

## XX. Geometry Scan Along Ni–O Distance

To evaluate the possibility of C=O $\eta^2$ coordination to the metal center, a geometry scan calculation was performed using Rowan.[21] The scan calculation was performed using OMol25's eSEN Sm. Cons. General NNP level of theory in the gas phase.[22,23] Beginning with the XRD structure of **($^{Fluor}N_3$)Ni($CF_3$)$_2$**, a 12 conformers were generated varying the Ni–O distance.

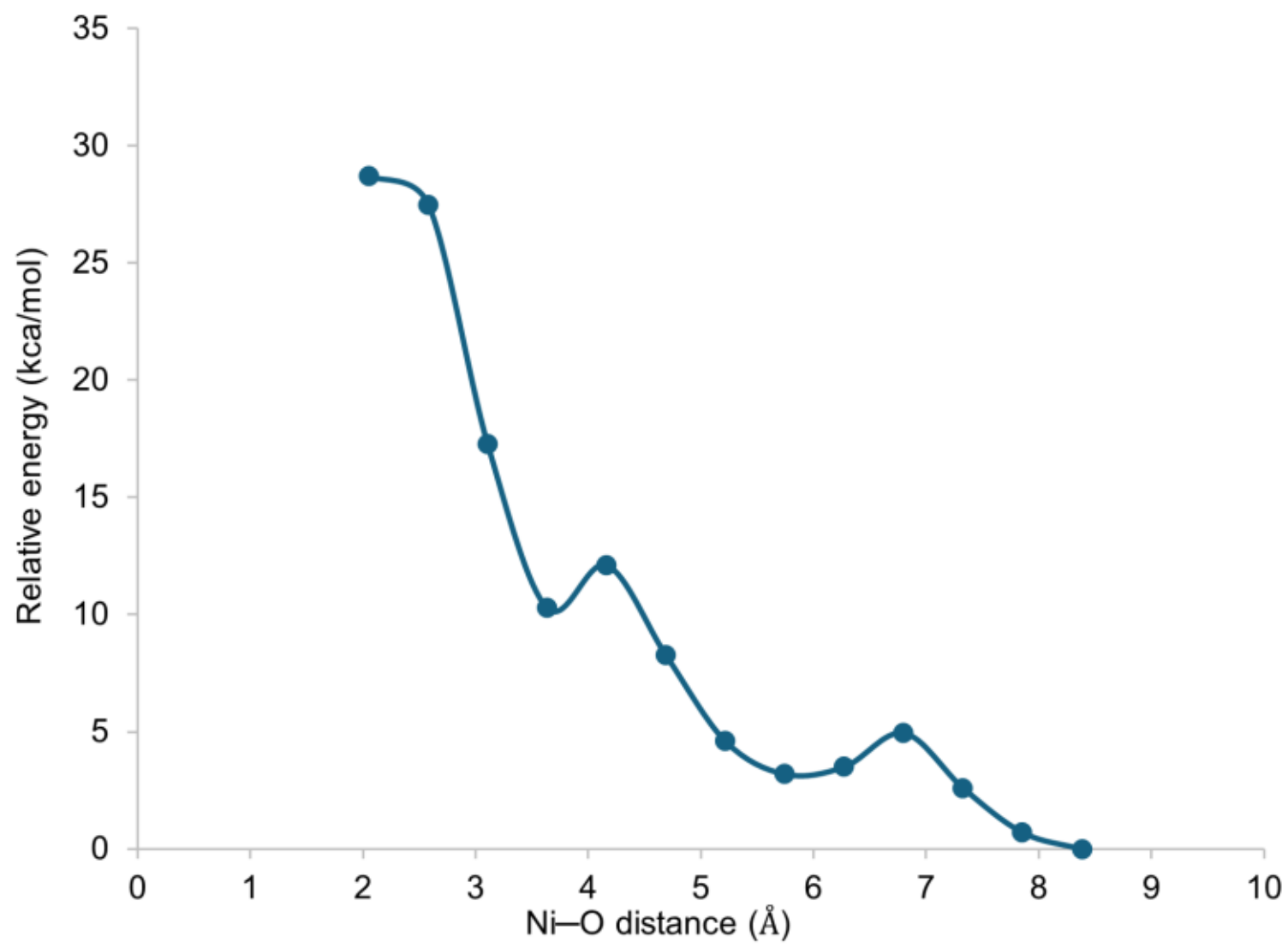


**Figure S65**: Relative energy (kcal/mol) plotted against the Ni–O distance.

## XXI. Hirshfeld Spin Density Analysis

The geometry of the Ni(III) complex **[($^{Fluor}$N$_3$)Ni(CF$_3$)$_2$(MeCN)]BF$_4$** was optimized without the counterion as a cationic doublet species at the m06 tzvp level of theory. Analysis of the spin density was conducted using Multiwfn with the Hirshfeld method.[24,25] The Hirshfeld spin population table is provided below with the corresponding spin density map.

# opt freq scrf=(solvent=benzene) guess=save pop=full m06 tzvp int=ultrafine scf=tight

NiIIIXRD_noBF4_opt

1 2

| Atomic space | Value | % of sum | % of sum abs |
|---|---|---|---|
| 1(Ni) | 0.90653699 | 90.653381 | 77.442756 |
| 2(F ) | -0.00157960 | -0.157959 | -0.134940 |
| 3(O ) | -0.00004955 | -0.004955 | -0.004233 |
| 4(N ) | 0.04255935 | 4.255920 | 3.635718 |
| 5(C ) | 0.01116631 | 1.116627 | 0.953904 |
| 6(F ) | -0.00536994 | -0.536992 | -0.458738 |
| 7(N ) | 0.00640323 | 0.640321 | 0.547009 |
| 8(C ) | 0.00123836 | 0.123836 | 0.105789 |
| 9(H ) | 0.00098333 | 0.098333 | 0.084003 |
| 10(H ) | -0.00007354 | -0.007354 | -0.006282 |
| 11(H ) | -0.00023923 | -0.023923 | -0.020437 |
| 12(F ) | -0.00660089 | -0.660087 | -0.563894 |
| 13(N ) | 0.00556018 | 0.556016 | 0.474989 |
| 14(C ) | -0.02585364 | -2.585355 | -2.208600 |
| 15(F ) | -0.00547250 | -0.547248 | -0.467499 |
| 16(N ) | 0.08900779 | 8.900748 | 7.603671 |
| 17(C ) | -0.02833445 | -2.833435 | -2.420528 |
| 18(F ) | -0.00237621 | -0.237621 | -0.202993 |
| 19(C ) | 0.00107405 | 0.107405 | 0.091753 |
| 20(F ) | -0.00692434 | -0.692432 | -0.591526 |
| 21(C ) | 0.00011539 | 0.011539 | 0.009857 |
| 22(H ) | 0.00016960 | 0.016960 | 0.014489 |
| 23(C ) | -0.00056204 | -0.056204 | -0.048014 |

| | | | |
|---|---|---|---|
| 24(H ) | -0.00005596 | -0.005596 | -0.004781 |
| 25(C ) | 0.00033818 | 0.033817 | 0.028889 |
| 26(H ) | 0.00010409 | 0.010409 | 0.008892 |
| 27(C ) | -0.00013391 | -0.013391 | -0.011439 |
| 28(H ) | 0.00042863 | 0.042863 | 0.036616 |
| 29(C ) | 0.00226552 | 0.226551 | 0.193537 |
| 30(H ) | 0.00023672 | 0.023672 | 0.020223 |
| 31(H ) | 0.00283091 | 0.283090 | 0.241836 |
| 32(C ) | -0.00049755 | -0.049755 | -0.042504 |
| 33(H ) | 0.00037434 | 0.037434 | 0.031978 |
| 34(C ) | 0.00034256 | 0.034256 | 0.029264 |
| 35(H ) | 0.00010660 | 0.010660 | 0.009106 |
| 36(C ) | -0.00070432 | -0.070431 | -0.060168 |
| 37(H ) | -0.00006343 | -0.006343 | -0.005419 |
| 38(C ) | 0.00032157 | 0.032157 | 0.027471 |
| 39(H ) | 0.00018978 | 0.018978 | 0.016212 |
| 40(C ) | 0.00035287 | 0.035287 | 0.030144 |
| 41(C ) | 0.00108334 | 0.108333 | 0.092546 |
| 42(H ) | 0.00291664 | 0.291663 | 0.249160 |
| 43(H ) | 0.00021446 | 0.021446 | 0.018320 |
| 44(C ) | 0.00099922 | 0.099922 | 0.085361 |
| 45(H ) | 0.00066299 | 0.066298 | 0.056637 |
| 46(H ) | 0.00035042 | 0.035042 | 0.029936 |
| 47(C ) | 0.00311128 | 0.311127 | 0.265788 |
| 48(C ) | 0.00091835 | 0.091835 | 0.078452 |
| 49(H ) | 0.00010598 | 0.010598 | 0.009054 |
| 50(C ) | -0.00011779 | -0.011779 | -0.010063 |
| 51(H ) | 0.00001012 | 0.001012 | 0.000865 |
| 52(C ) | 0.00071234 | 0.071233 | 0.060853 |
| 53(C ) | -0.00009356 | -0.009355 | -0.007992 |
| 54(C ) | 0.00073814 | 0.073814 | 0.063057 |
| 55(H ) | 0.00014438 | 0.014437 | 0.012334 |
| 56(C ) | 0.00002334 | 0.002334 | 0.001994 |

| | | | |
|---|---|---|---|
| 57(C ) | -0.00005376 | -0.005376 | -0.004593 |
| 58(C ) | 0.00018445 | 0.018445 | 0.015757 |
| 59(H ) | 0.00000856 | 0.000856 | 0.000731 |
| 60(C ) | -0.00006535 | -0.006535 | -0.005583 |
| 61(H ) | -0.00000437 | -0.000437 | -0.000373 |
| 62(C ) | 0.00020990 | 0.020990 | 0.017931 |
| 63(H ) | 0.00001103 | 0.001103 | 0.000942 |
| 64(C ) | -0.00006302 | -0.006302 | -0.005384 |
| 65(H ) | -0.00000419 | -0.000419 | -0.000358 |
| 66(C ) | 0.00018538 | 0.018538 | 0.015836 |

Summing up above values: 1.00000350

Summing up absolute value of above values: 1.17058978

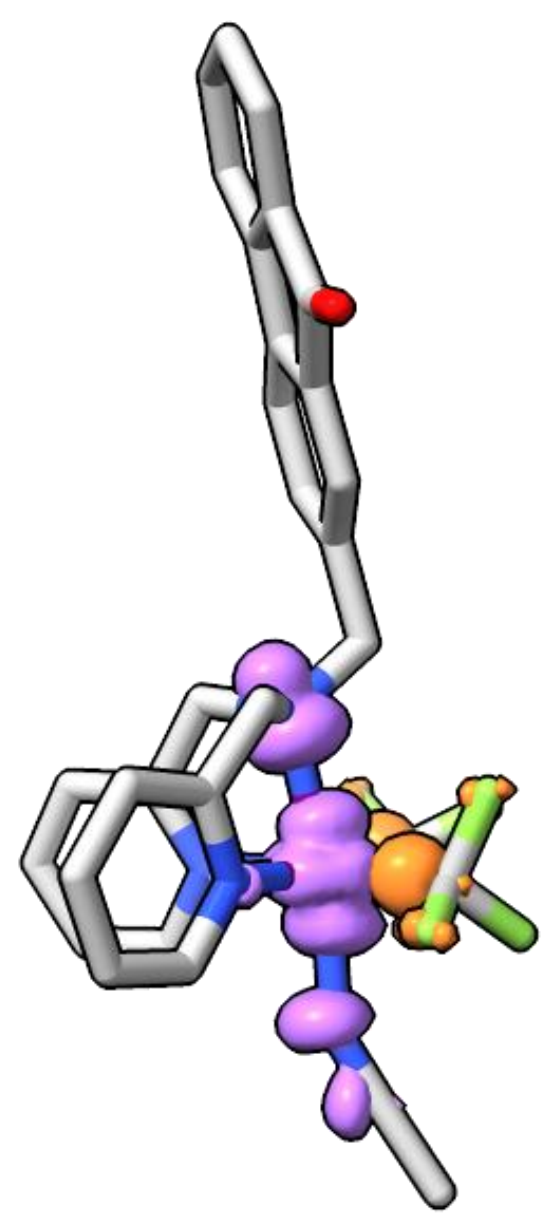

**Figure S66**: Spin density map of **[($^{Fluor}$N$_3$)Ni(CF$_3$)$_2$(MeCN)]BF$_4$**.

## XXII. Crystallographic Data.

**($^{Bn}N_3$)Ni($CF_3$)$_2$**

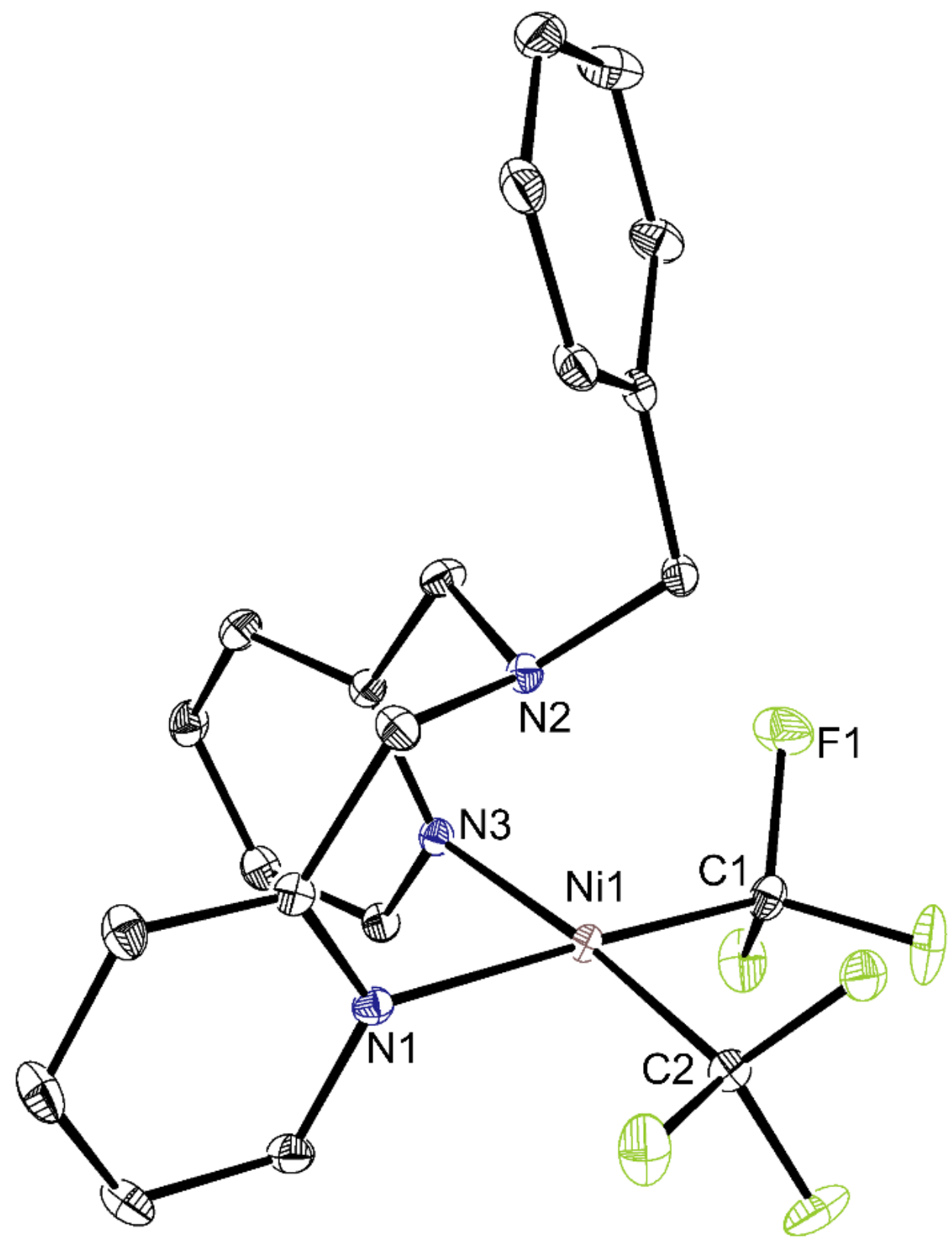


X-ray crystal structure of **($^{Bn}N_3$)Ni($CF_3$)$_2$** at 30% probability ellipsoids. Hydrogen atoms and solvent omitted for clarity.

### Refinement

Crystal data, data collection and structure refinement details are summarized in Table 7.

***Table 7***

*Experimental details*

| | |
|---|---|
| Crystal data | |
| Chemical formula | $C_{21}H_{19}F_6N_3Ni{\cdot}C_4H_8O$ |
| $M_r$ | 558.20 |
| Crystal system, space group | Monoclinic, $P2_1/n$ |
| Temperature (K) | 100 |

| | |
|---|---|
| *a*, *b*, *c* (Å) | 11.5205 (2), 18.1380 (4), 12.4674 (3) |
| β (°) | 111.049 (1) |
| $V$ (Å$^3$) | 2431.34 (9) |
| $Z$ | 4 |
| Radiation type | Cu $K\alpha$ |
| μ (mm$^{-1}$) | 1.78 |
| Crystal size (mm) | 0.50 × 0.29 × 0.17 |
| | |
| Data collection | |
| Diffractometer | Bruker *APEX*-II CCD |
| Absorption correction | Multi-scan<br>*SADABS2016*/2 (Bruker,2016/2) was used for absorption correction. wR2(int) was 0.1559 before and 0.0617 after correction. The Ratio of minimum to maximum transmission is 0.7697. The λ/2 correction factor is Not present. |
| $T_{min}$, $T_{max}$ | 0.579, 0.753 |
| No. of measured, independent and observed [$I > 2\sigma(I)$] reflections | 27287, 4281, 3986 |
| $R_{int}$ | 0.090 |
| $(\sin \theta/\lambda)_{max}$ (Å$^{-1}$) | 0.596 |
| | |
| Refinement | |
| $R[F^2 > 2\sigma(F^2)]$, $wR(F^2)$, $S$ | 0.033, 0.083, 1.06 |
| No. of reflections | 4281 |
| No. of parameters | 325 |
| H-atom treatment | H-atom parameters constrained |
| $\Delta\rangle_{max}$, $\Delta\rangle_{min}$ (e Å$^{-3}$) | 0.57, -0.49 |

Computer programs: SHELXT 2014/5 (Sheldrick, 2014), *SHELXL* 2018/3 (Sheldrick, 2015), Olex2 1.5 (Dolomanov *et al.*, 2009).

## References


Dolomanov, O. V., Bourhis, L. J., Gildea, R. J., Howard, J. A. K. & Puschmann, H. (2009). *J. Appl. Cryst.* **42**, 339–341.

Sheldrick, G. M. (2015). *Acta Cryst.* C**71**, 3–8.


Document origin: *publCIF* [Westrip, S. P. (2010). *J. Apply. Cryst.*, **43**, 920-925].

**supporting information**

## Computing details

Program(s) used to solve structure: SHELXT 2014/5 (Sheldrick, 2014); program(s) used to refine structure: *SHELXL* 2018/3 (Sheldrick, 2015); molecular graphics: Olex2 1.5 (Dolomanov *et al.*, 2009); software used to prepare material for publication: Olex2 1.5 (Dolomanov *et al.*, 2009).

## (hh01_full_1_a)

### *Crystal data*

| | |
|---|---|
| $C_{21}H_{19}F_6N_3Ni{\cdot}C_4H_8O$ | $F(000) = 1152$ |
| $M_r = 558.20$ | $D_x = 1.525$ Mg m$^{-3}$ |
| Monoclinic, $P2_1/n$ | Cu $K\alpha$ radiation, $\lambda = 1.54178$ Å |
| $a = 11.5205$ (2) Å | Cell parameters from 9773 reflections |
| $b = 18.1380$ (4) Å | $\theta = 4.5–66.8°$ |
| $c = 12.4674$ (3) Å | $\mu = 1.78$ mm$^{-1}$ |
| $\beta = 111.049$ (1)° | $T = 100$ K |
| $V = 2431.34$ (9) Å$^3$ | Irregular, light yellow |
| $Z = 4$ | 0.50 × 0.29 × 0.17 mm |

### *Data collection*

| | |
|---|---|
| Bruker APEX-II CCD diffractometer | 3986 reflections with $I > 2\sigma(I)$ |
| $\phi$ and $\omega$ scans | $R_{int} = 0.090$ |
| Absorption correction: multi-scan *SADABS2016*/2 (Bruker,2016/2) was used for absorption correction. wR2(int) was 0.1559 before and 0.0617 after correction. The Ratio of minimum to maximum transmission is 0.7697. The λ/2 correction factor is Not present. | $\theta_{max} = 66.8°$, $\theta_{min} = 4.5°$ |
| $T_{min} = 0.579$, $T_{max} = 0.753$ | $h = -13\rightarrow13$ |
| 27287 measured reflections | $k = -19\rightarrow21$ |
| 4281 independent reflections | $l = -14\rightarrow14$ |

### *Refinement*

| | |
|---|---|
| Refinement on $F^2$ | 0 restraints |
| Least-squares matrix: full | Hydrogen site location: inferred from neighbouring sites |
| $R[F^2 > 2\sigma(F^2)] = 0.033$ | H-atom parameters constrained |

| | |
|---|---|
| $wR(F^2)$ = 0.083 | $w = 1/[\sigma^2(F_o^2) + (0.0279P)^2 + 1.4845P]$ where $P = (F_o^2 + 2F_c^2)/3$ |
| $S$ = 1.06 | $(\Delta/\sigma)_{max}$ = 0.001 |
| 4281 reflections | $\Delta\rangle_{max}$ = 0.57 e Å$^{-3}$ |
| 325 parameters | $\Delta\rangle_{min}$ = -0.49 e Å$^{-3}$ |

*Special details*

| |
|---|
| *Geometry*. All esds (except the esd in the dihedral angle between two l.s. planes) are estimated using the full covariance matrix. The cell esds are taken into account individually in the estimation of esds in distances, angles and torsion angles; correlations between esds in cell parameters are only used when they are defined by crystal symmetry. An approximate (isotropic) treatment of cell esds is used for estimating esds involving l.s. planes. |

*Fractional atomic coordinates and isotropic or equivalent isotropic displacement parameters (Å$^2$) for (hh01_full_1_a)*

| | $x$ | $y$ | $z$ | $U_{iso}$*/$U_{eq}$ |
|---|---|---|---|---|
| Ni1 | 0.79202 (2) | 0.40544 (2) | 0.43988 (2) | 0.01286 (10) |
| F1 | 0.83063 (9) | 0.27354 (6) | 0.55929 (8) | 0.0239 (2) |
| F5 | 0.88793 (11) | 0.50687 (6) | 0.61131 (9) | 0.0298 (3) |
| F3 | 0.88853 (12) | 0.26844 (6) | 0.41495 (9) | 0.0355 (3) |
| F2 | 1.00243 (9) | 0.32411 (7) | 0.56809 (10) | 0.0334 (3) |
| F6 | 0.88287 (13) | 0.40181 (6) | 0.68562 (9) | 0.0370 (3) |
| F4 | 0.71306 (10) | 0.46139 (7) | 0.60512 (9) | 0.0342 (3) |
| N3 | 0.71097 (12) | 0.49847 (8) | 0.37432 (11) | 0.0146 (3) |
| N1 | 0.77069 (12) | 0.38058 (8) | 0.28152 (11) | 0.0152 (3) |
| N2 | 0.57690 (12) | 0.36637 (8) | 0.36585 (11) | 0.0140 (3) |
| O1 | 0.27030 (14) | 0.48492 (9) | 0.08211 (12) | 0.0379 (4) |
| C17 | 0.58677 (15) | 0.49995 (9) | 0.31919 (13) | 0.0152 (3) |
| C10 | 0.39848 (15) | 0.29946 (9) | 0.39774 (13) | 0.0153 (3) |
| C21 | 0.77911 (15) | 0.55652 (10) | 0.36391 (13) | 0.0172 (3) |
| H21 | 0.866669 | 0.555358 | 0.403073 | 0.021* |
| C7 | 0.66455 (15) | 0.35034 (9) | 0.21090 (13) | 0.0157 (3) |
| C9 | 0.53504 (15) | 0.32094 (10) | 0.44439 (13) | 0.0158 (3) |
| H9A | 0.551967 | 0.348577 | 0.516905 | 0.019* |
| H9B | 0.585588 | 0.275339 | 0.463193 | 0.019* |
| C1 | 0.88012 (15) | 0.31736 (10) | 0.49734 (14) | 0.0178 (3) |
| C3 | 0.85881 (16) | 0.39990 (10) | 0.23908 (15) | 0.0202 (4) |
| H3 | 0.933008 | 0.422480 | 0.288848 | 0.024* |

| | | | | |
|---|---|---|---|---|
| C16 | 0.51087 (15) | 0.43691 (10) | 0.33949 (14) | 0.0183 (3) |
| H16A | 0.483864 | 0.450209 | 0.403929 | 0.022* |
| H16B | 0.435179 | 0.430683 | 0.270065 | 0.022* |
| C15 | 0.35682 (16) | 0.23945 (10) | 0.32412 (14) | 0.0198 (4) |
| H15 | 0.415286 | 0.209307 | 0.306901 | 0.024* |
| C2 | 0.82207 (15) | 0.44167 (10) | 0.58945 (14) | 0.0181 (3) |
| C8 | 0.57109 (15) | 0.32488 (10) | 0.26262 (14) | 0.0182 (3) |
| H8A | 0.486422 | 0.329687 | 0.204098 | 0.022* |
| H8B | 0.585581 | 0.272022 | 0.282758 | 0.022* |
| C18 | 0.52827 (16) | 0.55946 (10) | 0.25036 (14) | 0.0201 (4) |
| H18 | 0.440668 | 0.559456 | 0.211339 | 0.024* |
| C20 | 0.72653 (16) | 0.61763 (10) | 0.29847 (14) | 0.0200 (4) |
| H20 | 0.776682 | 0.658233 | 0.293980 | 0.024* |
| C11 | 0.31132 (16) | 0.34093 (10) | 0.42578 (15) | 0.0223 (4) |
| H11 | 0.338363 | 0.381170 | 0.477589 | 0.027* |
| C14 | 0.23063 (17) | 0.22314 (11) | 0.27552 (14) | 0.0242 (4) |
| H14 | 0.203124 | 0.182715 | 0.224153 | 0.029* |
| C6 | 0.64468 (17) | 0.33737 (10) | 0.09572 (14) | 0.0223 (4) |
| H6 | 0.568931 | 0.315919 | 0.046816 | 0.027* |
| C19 | 0.59905 (17) | 0.61854 (10) | 0.23943 (14) | 0.0216 (4) |
| H19 | 0.560784 | 0.659281 | 0.192021 | 0.026* |
| C4 | 0.84479 (18) | 0.38794 (11) | 0.12562 (16) | 0.0264 (4) |
| H4 | 0.908757 | 0.401473 | 0.098110 | 0.032* |
| C13 | 0.14497 (17) | 0.26608 (12) | 0.30226 (16) | 0.0276 (4) |
| H13 | 0.058615 | 0.255676 | 0.268033 | 0.033* |
| C5 | 0.73635 (19) | 0.35601 (12) | 0.05295 (15) | 0.0281 (4) |
| H5 | 0.724736 | 0.346939 | -0.025279 | 0.034* |
| C12 | 0.18557 (17) | 0.32401 (11) | 0.37880 (18) | 0.0285 (4) |
| H12 | 0.127261 | 0.352288 | 0.399258 | 0.034* |
| C22 | 0.16964 (19) | 0.45235 (13) | 0.10623 (17) | 0.0334 (5) |
| H22A | 0.095053 | 0.484256 | 0.077821 | 0.040* |
| H22B | 0.192352 | 0.445296 | 0.190039 | 0.040* |
| C25 | 0.29402 (19) | 0.44284 (13) | -0.00587 (18) | 0.0337 (5) |
| H25A | 0.370923 | 0.413345 | 0.027773 | 0.040* |
| H25B | 0.303881 | 0.476030 | -0.065061 | 0.040* |
| C24 | 0.1838 (2) | 0.39332 (13) | -0.05769 (19) | 0.0359 (5) |
| H24A | 0.207290 | 0.346738 | -0.086023 | 0.043* |

| | | | | |
|---|---|---|---|---|
| H24B | 0.117212 | 0.417946 | -0.121285 | 0.043* |
| C23 | 0.1447 (3) | 0.38007 (14) | 0.0460 (2) | 0.0470 (6) |
| H23A | 0.055494 | 0.366924 | 0.021160 | 0.056* |
| H23B | 0.194830 | 0.340377 | 0.095795 | 0.056* |

*Atomic displacement parameters (Å$^2$) for (hh01_full_1_a)*

| | $U^{11}$ | $U^{22}$ | $U^{33}$ | $U^{12}$ | $U^{13}$ | $U^{23}$ |
|---|---|---|---|---|---|---|
| Ni1 | 0.01356 (16) | 0.01192 (17) | 0.01138 (15) | 0.00077 (10) | 0.00239 (11) | 0.00014 (10) |
| F1 | 0.0251 (5) | 0.0185 (6) | 0.0286 (5) | 0.0016 (4) | 0.0101 (4) | 0.0090 (4) |
| F5 | 0.0397 (6) | 0.0216 (6) | 0.0231 (5) | -0.0144 (5) | 0.0050 (5) | -0.0062 (4) |
| F3 | 0.0584 (8) | 0.0234 (6) | 0.0271 (6) | 0.0194 (5) | 0.0182 (5) | 0.0019 (4) |
| F2 | 0.0144 (5) | 0.0251 (6) | 0.0500 (7) | 0.0017 (4) | -0.0015 (5) | 0.0085 (5) |
| F6 | 0.0650 (8) | 0.0233 (6) | 0.0136 (5) | 0.0085 (5) | 0.0032 (5) | 0.0030 (4) |
| F4 | 0.0291 (6) | 0.0493 (8) | 0.0277 (5) | -0.0028 (5) | 0.0143 (5) | -0.0169 (5) |
| N3 | 0.0153 (7) | 0.0155 (7) | 0.0124 (6) | -0.0006 (5) | 0.0044 (5) | -0.0014 (5) |
| N1 | 0.0147 (7) | 0.0132 (7) | 0.0177 (6) | 0.0013 (5) | 0.0057 (5) | 0.0006 (5) |
| N2 | 0.0150 (6) | 0.0135 (7) | 0.0132 (6) | -0.0009 (5) | 0.0046 (5) | -0.0007 (5) |
| O1 | 0.0386 (8) | 0.0484 (10) | 0.0255 (7) | -0.0135 (7) | 0.0099 (6) | -0.0058 (6) |
| C17 | 0.0158 (8) | 0.0149 (9) | 0.0142 (7) | 0.0011 (6) | 0.0047 (6) | -0.0016 (6) |
| C10 | 0.0181 (8) | 0.0159 (9) | 0.0134 (7) | -0.0006 (7) | 0.0074 (6) | 0.0026 (6) |
| C21 | 0.0178 (8) | 0.0172 (9) | 0.0166 (7) | -0.0013 (7) | 0.0061 (6) | -0.0003 (6) |
| C7 | 0.0172 (8) | 0.0132 (9) | 0.0163 (8) | -0.0010 (6) | 0.0054 (6) | -0.0006 (6) |
| C9 | 0.0176 (8) | 0.0170 (9) | 0.0130 (7) | -0.0007 (7) | 0.0057 (6) | 0.0010 (6) |
| C1 | 0.0168 (8) | 0.0178 (9) | 0.0180 (8) | 0.0004 (7) | 0.0052 (6) | -0.0001 (6) |
| C3 | 0.0160 (8) | 0.0227 (10) | 0.0227 (8) | -0.0007 (7) | 0.0079 (7) | -0.0011 (7) |
| C16 | 0.0140 (8) | 0.0161 (9) | 0.0235 (8) | 0.0010 (7) | 0.0049 (6) | 0.0021 (6) |
| C15 | 0.0229 (9) | 0.0230 (10) | 0.0163 (8) | -0.0043 (7) | 0.0105 (7) | -0.0021 (7) |
| C2 | 0.0198 (8) | 0.0144 (9) | 0.0169 (7) | -0.0009 (7) | 0.0026 (6) | 0.0011 (6) |
| C8 | 0.0191 (8) | 0.0198 (9) | 0.0168 (8) | -0.0063 (7) | 0.0077 (7) | -0.0046 (6) |
| C18 | 0.0182 (8) | 0.0187 (10) | 0.0213 (8) | 0.0042 (7) | 0.0046 (7) | 0.0022 (7) |
| C20 | 0.0250 (9) | 0.0158 (9) | 0.0206 (8) | -0.0027 (7) | 0.0100 (7) | 0.0005 (6) |
| C11 | 0.0238 (9) | 0.0173 (10) | 0.0301 (9) | -0.0014 (7) | 0.0149 (8) | -0.0008 (7) |
| C14 | 0.0270 (9) | 0.0291 (11) | 0.0169 (8) | -0.0110 (8) | 0.0083 (7) | -0.0012 (7) |
| C6 | 0.0272 (9) | 0.0224 (10) | 0.0172 (8) | -0.0076 (8) | 0.0078 (7) | -0.0049 (7) |
| C19 | 0.0259 (9) | 0.0173 (9) | 0.0207 (8) | 0.0043 (7) | 0.0073 (7) | 0.0048 (7) |
| C4 | 0.0282 (10) | 0.0303 (11) | 0.0285 (9) | -0.0031 (8) | 0.0196 (8) | -0.0020 (8) |
| C13 | 0.0181 (9) | 0.0317 (11) | 0.0308 (9) | -0.0037 (8) | 0.0062 (7) | 0.0128 (8) |

| | | | | | | |
|---|---|---|---|---|---|---|
| C5 | 0.0394 (11) | 0.0302 (11) | 0.0200 (8) | -0.0074 (9) | 0.0172 (8) | -0.0054 (7) |
| C12 | 0.0233 (9) | 0.0204 (10) | 0.0482 (11) | 0.0031 (8) | 0.0206 (9) | 0.0070 (8) |
| C22 | 0.0259 (10) | 0.0448 (13) | 0.0286 (10) | -0.0008 (9) | 0.0089 (8) | -0.0046 (9) |
| C25 | 0.0301 (10) | 0.0393 (13) | 0.0341 (10) | 0.0061 (9) | 0.0144 (9) | 0.0078 (9) |
| C24 | 0.0453 (12) | 0.0321 (12) | 0.0387 (11) | -0.0027 (10) | 0.0253 (10) | -0.0075 (9) |
| C23 | 0.0644 (16) | 0.0361 (14) | 0.0610 (15) | -0.0102 (12) | 0.0472 (14) | -0.0096 (11) |

*Geometric parameters (Å, º) for (hh01_full_1_a)*

| | | | |
|---|---|---|---|
| Ni1—N3 | 1.9592 (14) | C16—H16B | 0.9900 |
| Ni1—N1 | 1.9527 (13) | C15—H15 | 0.9500 |
| Ni1—N2 | 2.4193 (13) | C15—C14 | 1.391 (3) |
| Ni1—C1 | 1.8905 (18) | C8—H8A | 0.9900 |
| Ni1—C2 | 1.8882 (17) | C8—H8B | 0.9900 |
| F1—C1 | 1.367 (2) | C18—H18 | 0.9500 |
| F5—C2 | 1.378 (2) | C18—C19 | 1.383 (3) |
| F3—C1 | 1.387 (2) | C20—H20 | 0.9500 |
| F2—C1 | 1.373 (2) | C20—C19 | 1.387 (3) |
| F6—C2 | 1.359 (2) | C11—H11 | 0.9500 |
| F4—C2 | 1.386 (2) | C11—C12 | 1.388 (3) |
| N3—C17 | 1.347 (2) | C14—H14 | 0.9500 |
| N3—C21 | 1.347 (2) | C14—C13 | 1.388 (3) |
| N1—C7 | 1.341 (2) | C6—H6 | 0.9500 |
| N1—C3 | 1.349 (2) | C6—C5 | 1.385 (3) |
| N2—C9 | 1.486 (2) | C19—H19 | 0.9500 |
| N2—C16 | 1.464 (2) | C4—H4 | 0.9500 |
| N2—C8 | 1.472 (2) | C4—C5 | 1.380 (3) |
| O1—C22 | 1.426 (3) | C13—H13 | 0.9500 |
| O1—C25 | 1.441 (3) | C13—C12 | 1.383 (3) |
| C17—C16 | 1.515 (2) | C5—H5 | 0.9500 |
| C17—C18 | 1.393 (2) | C12—H12 | 0.9500 |
| C10—C9 | 1.519 (2) | C22—H22A | 0.9900 |
| C10—C15 | 1.393 (2) | C22—H22B | 0.9900 |
| C10—C11 | 1.396 (2) | C22—C23 | 1.487 (3) |
| C21—H21 | 0.9500 | C25—H25A | 0.9900 |
| C21—C20 | 1.381 (3) | C25—H25B | 0.9900 |
| C7—C8 | 1.512 (2) | C25—C24 | 1.499 (3) |
| C7—C6 | 1.391 (2) | C24—H24A | 0.9900 |

| | | | |
|---|---|---|---|
| C9—H9A | 0.9900 | C24—H24B | 0.9900 |
| C9—H9B | 0.9900 | C24—C23 | 1.533 (3) |
| C3—H3 | 0.9500 | C23—H23A | 0.9900 |
| C3—C4 | 1.382 (3) | C23—H23B | 0.9900 |
| C16—H16A | 0.9900 | | |
| | | | |
| N3—Ni1—N2 | 79.85 (5) | F6—C2—F4 | 102.65 (13) |
| N1—Ni1—N3 | 84.78 (5) | F4—C2—Ni1 | 112.03 (11) |
| N1—Ni1—N2 | 78.87 (5) | N2—C8—C7 | 113.65 (14) |
| C1—Ni1—N3 | 175.20 (6) | N2—C8—H8A | 108.8 |
| C1—Ni1—N1 | 92.49 (6) | N2—C8—H8B | 108.8 |
| C1—Ni1—N2 | 103.53 (6) | C7—C8—H8A | 108.8 |
| C2—Ni1—N3 | 90.18 (6) | C7—C8—H8B | 108.8 |
| C2—Ni1—N1 | 172.19 (7) | H8A—C8—H8B | 107.7 |
| C2—Ni1—N2 | 106.15 (6) | C17—C18—H18 | 120.3 |
| C2—Ni1—C1 | 92.11 (7) | C19—C18—C17 | 119.30 (16) |
| C17—N3—Ni1 | 119.33 (11) | C19—C18—H18 | 120.3 |
| C17—N3—C21 | 118.97 (14) | C21—C20—H20 | 120.7 |
| C21—N3—Ni1 | 120.42 (11) | C21—C20—C19 | 118.66 (16) |
| C7—N1—Ni1 | 120.82 (11) | C19—C20—H20 | 120.7 |
| C7—N1—C3 | 119.08 (14) | C10—C11—H11 | 119.6 |
| C3—N1—Ni1 | 119.84 (11) | C12—C11—C10 | 120.72 (17) |
| C9—N2—Ni1 | 116.19 (9) | C12—C11—H11 | 119.6 |
| C16—N2—Ni1 | 102.01 (9) | C15—C14—H14 | 120.1 |
| C16—N2—C9 | 111.53 (12) | C13—C14—C15 | 119.86 (18) |
| C16—N2—C8 | 113.18 (13) | C13—C14—H14 | 120.1 |
| C8—N2—Ni1 | 101.66 (9) | C7—C6—H6 | 120.3 |
| C8—N2—C9 | 111.66 (13) | C5—C6—C7 | 119.39 (16) |
| C22—O1—C25 | 109.39 (16) | C5—C6—H6 | 120.3 |
| N3—C17—C16 | 118.09 (14) | C18—C19—C20 | 119.22 (16) |
| N3—C17—C18 | 121.33 (16) | C18—C19—H19 | 120.4 |
| C18—C17—C16 | 120.48 (14) | C20—C19—H19 | 120.4 |
| C15—C10—C9 | 121.12 (15) | C3—C4—H4 | 120.5 |
| C15—C10—C11 | 118.51 (15) | C5—C4—C3 | 118.90 (16) |
| C11—C10—C9 | 120.35 (15) | C5—C4—H4 | 120.5 |
| N3—C21—H21 | 118.8 | C14—C13—H13 | 120.1 |
| N3—C21—C20 | 122.49 (15) | C12—C13—C14 | 119.89 (17) |

| | | | |
|---|---|---|---|
| C20—C21—H21 | 118.8 | C12—C13—H13 | 120.1 |
| N1—C7—C8 | 117.77 (13) | C6—C5—H5 | 120.5 |
| N1—C7—C6 | 121.36 (15) | C4—C5—C6 | 119.04 (16) |
| C6—C7—C8 | 120.68 (15) | C4—C5—H5 | 120.5 |
| N2—C9—C10 | 115.24 (13) | C11—C12—H12 | 119.9 |
| N2—C9—H9A | 108.5 | C13—C12—C11 | 120.15 (17) |
| N2—C9—H9B | 108.5 | C13—C12—H12 | 119.9 |
| C10—C9—H9A | 108.5 | O1—C22—H22A | 110.4 |
| C10—C9—H9B | 108.5 | O1—C22—H22B | 110.4 |
| H9A—C9—H9B | 107.5 | O1—C22—C23 | 106.43 (16) |
| F1—C1—Ni1 | 115.42 (11) | H22A—C22—H22B | 108.6 |
| F1—C1—F3 | 101.58 (14) | C23—C22—H22A | 110.4 |
| F1—C1—F2 | 103.23 (13) | C23—C22—H22B | 110.4 |
| F3—C1—Ni1 | 115.52 (11) | O1—C25—H25A | 110.4 |
| F2—C1—Ni1 | 117.10 (12) | O1—C25—H25B | 110.4 |
| F2—C1—F3 | 101.78 (13) | O1—C25—C24 | 106.42 (16) |
| N1—C3—H3 | 118.9 | H25A—C25—H25B | 108.6 |
| N1—C3—C4 | 122.21 (16) | C24—C25—H25A | 110.4 |
| C4—C3—H3 | 118.9 | C24—C25—H25B | 110.4 |
| N2—C16—C17 | 114.37 (13) | C25—C24—H24A | 111.6 |
| N2—C16—H16A | 108.7 | C25—C24—H24B | 111.6 |
| N2—C16—H16B | 108.7 | C25—C24—C23 | 100.99 (18) |
| C17—C16—H16A | 108.7 | H24A—C24—H24B | 109.4 |
| C17—C16—H16B | 108.7 | C23—C24—H24A | 111.6 |
| H16A—C16—H16B | 107.6 | C23—C24—H24B | 111.6 |
| C10—C15—H15 | 119.6 | C22—C23—C24 | 103.05 (19) |
| C14—C15—C10 | 120.78 (17) | C22—C23—H23A | 111.2 |
| C14—C15—H15 | 119.6 | C22—C23—H23B | 111.2 |
| F5—C2—Ni1 | 112.74 (11) | C24—C23—H23A | 111.2 |
| F5—C2—F4 | 102.31 (14) | C24—C23—H23B | 111.2 |
| F6—C2—Ni1 | 122.66 (12) | H23A—C23—H23B | 109.1 |
| F6—C2—F5 | 102.08 (13) | | |
| | | | |
| Ni1—N3—C17—C16 | 17.60 (18) | C21—N3—C17—C18 | 1.2 (2) |
| Ni1—N3—C17—C18 | -165.91 (12) | C21—C20—C19—C18 | 1.8 (2) |
| Ni1—N3—C21—C20 | 166.80 (12) | C7—N1—C3—C4 | -1.6 (3) |

| | | | |
|---|---|---|---|
| Ni1—N1—C7—C8 | -9.7 (2) | C7—C6—C5—C4 | -0.8 (3) |
| Ni1—N1—C7—C6 | 175.23 (13) | C9—N2—C16—C17 | 148.22 (13) |
| Ni1—N1—C3—C4 | -175.73 (15) | C9—N2—C8—C7 | -151.93 (14) |
| Ni1—N2—C9—C10 | 177.82 (10) | C9—C10—C15—C14 | -175.57 (15) |
| Ni1—N2—C16—C17 | 23.54 (14) | C9—C10—C11—C12 | 176.62 (16) |
| Ni1—N2—C8—C7 | -27.40 (15) | C1—Ni1—C2—F5 | 119.47 (12) |
| N3—Ni1—C2—F5 | -56.31 (12) | C1—Ni1—C2—F6 | -3.09 (15) |
| N3—Ni1—C2—F6 | -178.87 (14) | C1—Ni1—C2—F4 | -125.77 (12) |
| N3—Ni1—C2—F4 | 58.45 (12) | C3—N1—C7—C8 | 176.21 (15) |
| N3—C17—C16—N2 | -29.8 (2) | C3—N1—C7—C6 | 1.2 (3) |
| N3—C17—C18—C19 | -0.7 (2) | C3—C4—C5—C6 | 0.4 (3) |
| N3—C21—C20—C19 | -1.3 (2) | C16—N2—C9—C10 | 61.50 (18) |
| N1—Ni1—C1—F1 | -123.01 (12) | C16—N2—C8—C7 | 81.23 (17) |
| N1—Ni1—C1—F3 | -4.77 (13) | C16—C17—C18—C19 | 175.71 (15) |
| N1—Ni1—C1—F2 | 115.13 (12) | C15—C10—C9—N2 | 82.23 (19) |
| N1—C7—C8—N2 | 28.1 (2) | C15—C10—C11—C12 | -1.8 (3) |
| N1—C7—C6—C5 | 0.0 (3) | C15—C14—C13—C12 | -1.2 (3) |
| N1—C3—C4—C5 | 0.8 (3) | C2—Ni1—C1—F1 | 63.30 (13) |
| N2—Ni1—C1—F1 | -43.86 (12) | C2—Ni1—C1—F3 | -178.46 (13) |
| N2—Ni1—C1—F3 | 74.37 (13) | C2—Ni1—C1—F2 | -58.56 (13) |
| N2—Ni1—C1—F2 | -165.73 (11) | C8—N2—C9—C10 | -66.23 (18) |
| N2—Ni1—C2—F5 | -135.79 (11) | C8—N2—C16—C17 | -84.87 (16) |
| N2—Ni1—C2—F6 | 101.65 (14) | C8—C7—C6—C5 | -174.87 (18) |
| N2—Ni1—C2—F4 | -21.03 (13) | C18—C17—C16—N2 | 153.66 (15) |
| O1—C22—C23—C24 | -29.4 (2) | C11—C10—C9—N2 | -96.15 (18) |
| O1—C25—C24—C23 | -31.1 (2) | C11—C10—C15—C14 | 2.8 (2) |
| C17—N3—C21—C20 | -0.2 (2) | C14—C13—C12—C11 | 2.3 (3) |
| C17—C18—C19—C20 | -0.8 (3) | C6—C7—C8—N2 | -156.78 (16) |
| C10—C15—C14—C13 | -1.3 (3) | C22—O1—C25—C24 | 14.0 (2) |
| C10—C11—C12—C13 | -0.7 (3) | C25—O1—C22—C23 | 10.1 (2) |
| C21—N3—C17—C16 | -175.29 (14) | C25—C24—C23—C22 | 36.4 (2) |

Document origin: *publCIF* [Westrip, S. P. (2010). *J. Apply. Cryst.*, **43**, 920-925].

**($^{Fluor}N_3$)Ni($CF_3$)$_2$**

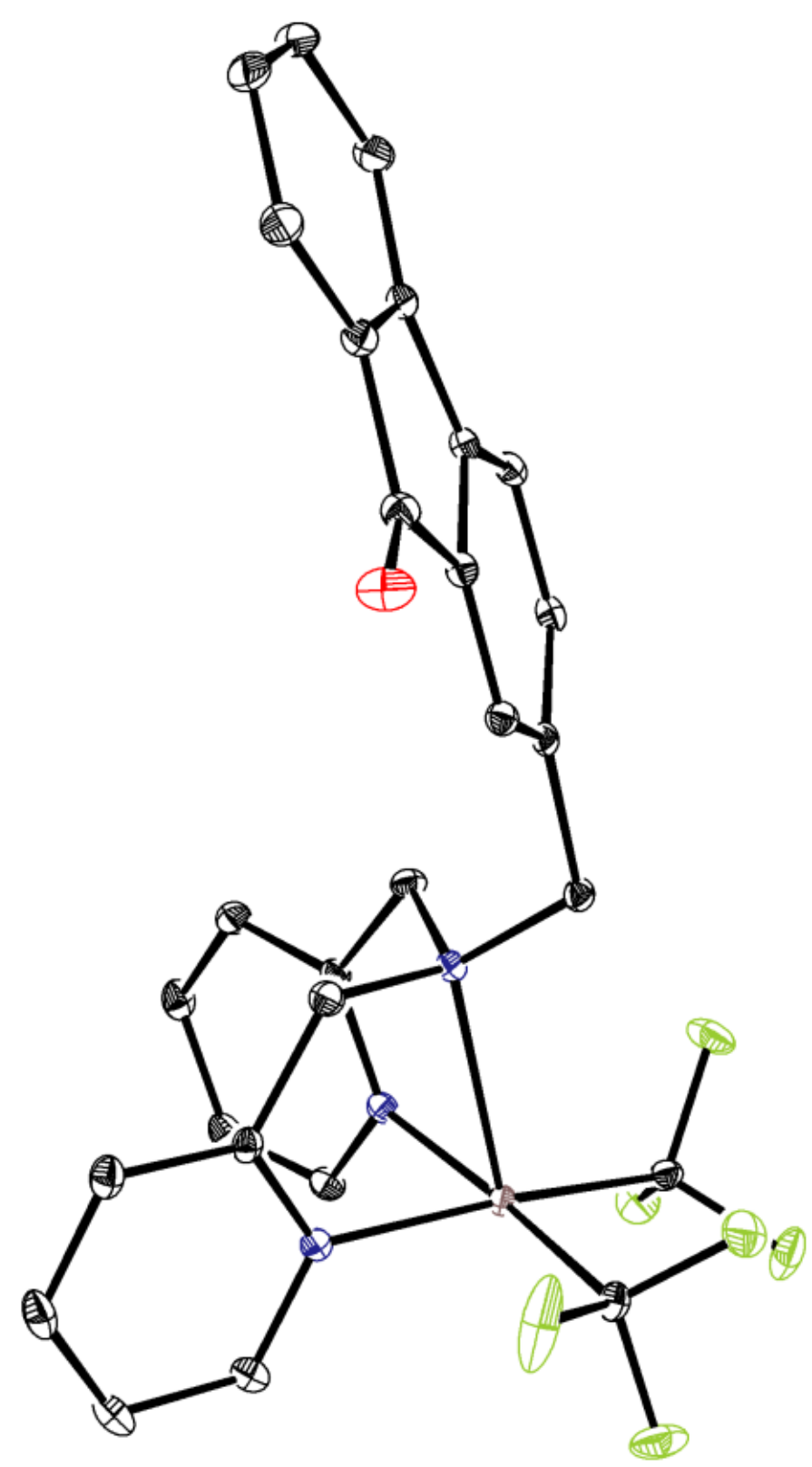

X-ray crystal structure for **($^{Fluor}N_3$)Ni($CF_3$)$_2$**. Hydrogen atoms omitted for clarity. Ellipsoids presented with 30% probabilities.

## 6. Refinement

Crystal data, data collection and structure refinement details are summarized in Table 9.

***Table 9***

*Experimental details*

| | |
|---|---|
| Crystal data | |
| Chemical formula | $C_{28}H_{21}F_6N_3NiO$ |

| | |
|---|---|
| $M_r$ | 588.19 |
| Crystal system, space group | Monoclinic, $P2_1/c$ |
| Temperature (K) | 100 |
| $a$, $b$, $c$ (Å) | 18.9667 (4), 14.9783 (3), 8.6799 (2) |
| β (°) | 100.401 (1) |
| $V$ (Å$^3$) | 2425.34 (9) |
| $Z$ | 4 |
| Radiation type | Cu $K\alpha$ |
| μ (mm$^{-1}$) | 1.83 |
| Crystal size (mm) | 0.26 × 0.17 × 0.15 |
| | |
| Data collection | |
| Diffractometer | Bruker *APEX*-II CCD |
| Absorption correction | Multi-scan<br>*SADABS2016*/2 (Bruker,2016/2) was used for absorption correction. wR2(int) was 0.0902 before and 0.0464 after correction. The Ratio of minimum to maximum transmission is 0.8828. The λ/2 correction factor is Not present. |
| $T_{min}$, $T_{max}$ | 0.665, 0.753 |
| No. of measured, independent and observed [$I > 2\sigma(I)$] reflections | 43377, 4296, 3972 |
| $R_{int}$ | 0.034 |
| $(\sin \theta/\lambda)_{max}$ (Å$^{-1}$) | 0.596 |
| | |
| Refinement | |
| $R[F^2 > 2\sigma(F^2)]$, $wR(F^2)$, $S$ | 0.027, 0.067, 1.04 |
| No. of reflections | 4296 |
| No. of parameters | 352 |
| H-atom treatment | H-atom parameters constrained |
| $\Delta\rangle_{max}$, $\Delta\rangle_{min}$ (e Å$^{-3}$) | 0.62, -0.35 |

Computer programs: SHELXT 2014/5 (Sheldrick, 2014), *SHELXL* 2018/3 (Sheldrick, 2015), Olex2 1.5 (Dolomanov *et al.*, 2009).

## References

Dolomanov, O. V., Bourhis, L. J., Gildea, R. J., Howard, J. A. K. & Puschmann, H. (2009). *J. Appl.*

*Cryst.* **42**, 339–341.

Sheldrick, G. M. (2015). *Acta Cryst.* C**71**, 3–8.

Document origin: *publCIF* [Westrip, S. P. (2010). *J. Apply. Cryst.*, **43**, 920-925].

**supporting information**

## Computing details

Program(s) used to solve structure: SHELXT 2014/5 (Sheldrick, 2014); program(s) used to refine structure: *SHELXL* 2018/3 (Sheldrick, 2015); molecular graphics: Olex2 1.5 (Dolomanov *et al.*, 2009); software used to prepare material for publication: Olex2 1.5 (Dolomanov *et al.*, 2009).

## (hh08_full_1_a)

### *Crystal data*

| | |
|---|---|
| $C_{28}H_{21}F_6N_3NiO$ | $F(000) = 1200$ |
| $M_r = 588.19$ | $D_x = 1.611$ Mg m$^{-3}$ |
| Monoclinic, $P2_1/c$ | Cu $K\alpha$ radiation, $\lambda = 1.54178$ Å |
| $a = 18.9667$ (4) Å | Cell parameters from 9994 reflections |
| $b = 14.9783$ (3) Å | $\theta = 2.4–66.6°$ |
| $c = 8.6799$ (2) Å | $\mu = 1.83$ mm$^{-1}$ |
| $\beta = 100.401$ (1)° | $T = 100$ K |
| $V = 2425.34$ (9) Å$^3$ | Plate, clear light yellow |
| $Z = 4$ | 0.26 × 0.17 × 0.15 mm |

### *Data collection*

| | |
|---|---|
| Bruker APEX-II CCD diffractometer | 3972 reflections with $I > 2\sigma(I)$ |
| $\phi$ and $\omega$ scans | $R_{int} = 0.034$ |
| Absorption correction: multi-scan *SADABS2016*/2 (Bruker,2016/2) was used for absorption correction. wR2(int) was 0.0902 before and 0.0464 after correction. The Ratio of minimum to maximum transmission is 0.8828. The λ/2 correction factor is Not present. | $\theta_{max} = 66.7°$, $\theta_{min} = 2.4°$ |
| $T_{min} = 0.665$, $T_{max} = 0.753$ | $h = -22\rightarrow22$ |
| 43377 measured reflections | $k = -17\rightarrow17$ |
| 4296 independent reflections | $l = -10\rightarrow10$ |

### *Refinement*

| | |
|---|---|
| Refinement on $F^2$ | 0 restraints |
| Least-squares matrix: full | Hydrogen site location: inferred from neighbouring sites |
| $R[F^2 > 2\sigma(F^2)] = 0.027$ | H-atom parameters constrained |
| $wR(F^2) = 0.067$ | $w = 1/[\sigma^2(F_o^2) + (0.026P)^2 + 2.2141P]$ where $P = (F_o^2 + 2F_c^2)/3$ |
| $S = 1.04$ | $(\Delta/\sigma)_{max} = 0.001$ |
| 4296 reflections | $\Delta\rangle_{max} = 0.62$ e Å$^{-3}$ |
| 352 parameters | $\Delta\rangle_{min} = -0.35$ e Å$^{-3}$ |

### *Special details*

| |
|---|
| *Geometry*. All esds (except the esd in the dihedral angle between two l.s. planes) are estimated using the full covariance matrix. The cell esds are taken into account individually in the estimation of esds in distances, angles and torsion angles; correlations between esds in cell parameters are only used when they are defined by crystal symmetry. An approximate (isotropic) treatment of cell esds is used for estimating esds involving l.s. planes. |

### *Fractional atomic coordinates and isotropic or equivalent isotropic displacement parameters (Å$^2$) for (hh08_full_1_a)*

| | $x$ | $y$ | $z$ | $U_{iso}$*/$U_{eq}$ |
|---|---|---|---|---|
| Ni1 | 0.86181 (2) | 0.53555 (2) | 0.84905 (3) | 0.01200 (8) |
| F1 | 0.90313 (5) | 0.69058 (7) | 0.73021 (12) | 0.0284 (2) |
| F2 | 0.99236 (5) | 0.61800 (7) | 0.86019 (12) | 0.0268 (2) |
| F3 | 0.92674 (6) | 0.69549 (7) | 0.98298 (12) | 0.0325 (3) |
| F4 | 0.78785 (6) | 0.66744 (7) | 0.98012 (12) | 0.0317 (3) |
| F5 | 0.84912 (6) | 0.58311 (8) | 1.15283 (11) | 0.0343 (3) |
| F6 | 0.75132 (7) | 0.53613 (9) | 1.01570 (19) | 0.0566 (4) |
| O1 | 0.43948 (6) | 0.57426 (9) | 0.35964 (14) | 0.0256 (3) |
| N1 | 0.91131 (7) | 0.48043 (9) | 0.69486 (15) | 0.0139 (3) |
| N2 | 0.81260 (7) | 0.42062 (9) | 0.84883 (15) | 0.0136 (3) |
| N3 | 0.77045 (7) | 0.54821 (8) | 0.61457 (15) | 0.0131 (3) |
| C1 | 0.92054 (9) | 0.63783 (11) | 0.86025 (18) | 0.0166 (3) |
| C2 | 0.81282 (9) | 0.58200 (11) | 1.00243 (19) | 0.0172 (3) |
| C3 | 0.97086 (8) | 0.43127 (11) | 0.74112 (19) | 0.0173 (3) |
| H3 | 0.994167 | 0.434502 | 0.847468 | 0.021* |
| C4 | 0.99942 (9) | 0.37634 (11) | 0.6402 (2) | 0.0199 (3) |
| H4 | 1.041055 | 0.341743 | 0.676808 | 0.024* |
| C5 | 0.96619 (9) | 0.37274 (11) | 0.4847 (2) | 0.0210 (4) |

| | | | | |
|---|---|---|---|---|
| H5 | 0.984508 | 0.335443 | 0.412627 | 0.025* |
| C6 | 0.90562 (9) | 0.42457 (11) | 0.43591 (19) | 0.0188 (3) |
| H6 | 0.882561 | 0.424021 | 0.329234 | 0.023* |
| C7 | 0.87892 (8) | 0.47721 (10) | 0.54374 (18) | 0.0142 (3) |
| C8 | 0.81548 (8) | 0.53797 (11) | 0.49444 (18) | 0.0159 (3) |
| H8A | 0.833019 | 0.597519 | 0.469302 | 0.019* |
| H8B | 0.785752 | 0.513882 | 0.397862 | 0.019* |
| C9 | 0.72367 (9) | 0.47181 (11) | 0.62530 (19) | 0.0179 (3) |
| H9A | 0.711073 | 0.444180 | 0.520315 | 0.021* |
| H9B | 0.678696 | 0.493816 | 0.654729 | 0.021* |
| C10 | 0.75494 (8) | 0.40065 (10) | 0.74087 (18) | 0.0146 (3) |
| C11 | 0.72151 (9) | 0.31775 (11) | 0.7400 (2) | 0.0211 (4) |
| H11 | 0.680377 | 0.304591 | 0.663377 | 0.025* |
| C12 | 0.74855 (10) | 0.25505 (12) | 0.8511 (2) | 0.0263 (4) |
| H12 | 0.726067 | 0.198445 | 0.852358 | 0.032* |
| C13 | 0.80894 (9) | 0.27542 (12) | 0.9611 (2) | 0.0240 (4) |
| H13 | 0.828850 | 0.232994 | 1.038187 | 0.029* |
| C14 | 0.83942 (9) | 0.35861 (11) | 0.95609 (19) | 0.0183 (3) |
| H14 | 0.880891 | 0.372822 | 1.031038 | 0.022* |
| C15 | 0.72998 (8) | 0.63335 (10) | 0.59586 (18) | 0.0147 (3) |
| H15A | 0.764473 | 0.683172 | 0.598442 | 0.018* |
| H15B | 0.705349 | 0.640780 | 0.686350 | 0.018* |
| C16 | 0.67458 (8) | 0.64033 (10) | 0.44589 (18) | 0.0137 (3) |
| C17 | 0.69539 (8) | 0.66689 (10) | 0.30605 (19) | 0.0152 (3) |
| H17 | 0.743692 | 0.684404 | 0.308148 | 0.018* |
| C18 | 0.64748 (8) | 0.66847 (10) | 0.16364 (18) | 0.0155 (3) |
| H18 | 0.662871 | 0.685862 | 0.069792 | 0.019* |
| C19 | 0.57723 (8) | 0.64416 (10) | 0.16252 (18) | 0.0144 (3) |
| C20 | 0.51592 (8) | 0.63284 (10) | 0.03120 (18) | 0.0162 (3) |
| C21 | 0.51120 (9) | 0.64283 (11) | -0.12894 (19) | 0.0213 (4) |
| H21 | 0.550456 | 0.665341 | -0.171343 | 0.026* |
| C22 | 0.44699 (10) | 0.61885 (12) | -0.2265 (2) | 0.0253 (4) |
| H22 | 0.442787 | 0.624635 | -0.336796 | 0.030* |
| C23 | 0.38926 (10) | 0.58669 (12) | -0.1647 (2) | 0.0257 (4) |
| H23 | 0.346476 | 0.569880 | -0.233362 | 0.031* |
| C24 | 0.39325 (9) | 0.57877 (12) | -0.0035 (2) | 0.0224 (4) |
| H24 | 0.353498 | 0.557985 | 0.039108 | 0.027* |

| | | | | |
|---|---|---|---|---|
| C25 | 0.45684 (9) | 0.60209 (11) | 0.09253 (19) | 0.0179 (3) |
| C26 | 0.47759 (8) | 0.59529 (11) | 0.26698 (19) | 0.0172 (3) |
| C27 | 0.55512 (8) | 0.62020 (10) | 0.30263 (18) | 0.0145 (3) |
| C28 | 0.60272 (8) | 0.61772 (10) | 0.44355 (18) | 0.0146 (3) |
| H28 | 0.586930 | 0.600954 | 0.537272 | 0.018* |

*Atomic displacement parameters (Å$^2$) for (hh08_full_1_a)*

| | $U^{11}$ | $U^{22}$ | $U^{33}$ | $U^{12}$ | $U^{13}$ | $U^{23}$ |
|---|---|---|---|---|---|---|
| Ni1 | 0.01249 (14) | 0.01137 (14) | 0.01256 (14) | -0.00081 (10) | 0.00340 (10) | -0.00097 (10) |
| F1 | 0.0274 (5) | 0.0265 (5) | 0.0284 (5) | -0.0072 (4) | -0.0026 (4) | 0.0149 (4) |
| F2 | 0.0149 (5) | 0.0301 (6) | 0.0340 (6) | -0.0063 (4) | 0.0011 (4) | -0.0002 (5) |
| F3 | 0.0460 (7) | 0.0263 (6) | 0.0299 (6) | -0.0190 (5) | 0.0195 (5) | -0.0142 (5) |
| F4 | 0.0418 (6) | 0.0260 (6) | 0.0294 (6) | 0.0175 (5) | 0.0119 (5) | 0.0000 (4) |
| F5 | 0.0404 (6) | 0.0499 (7) | 0.0136 (5) | 0.0140 (5) | 0.0078 (4) | 0.0020 (5) |
| F6 | 0.0388 (7) | 0.0486 (8) | 0.0969 (11) | -0.0292 (6) | 0.0509 (7) | -0.0481 (8) |
| O1 | 0.0157 (6) | 0.0389 (7) | 0.0223 (6) | -0.0041 (5) | 0.0042 (5) | 0.0060 (5) |
| N1 | 0.0128 (6) | 0.0135 (6) | 0.0156 (6) | -0.0005 (5) | 0.0033 (5) | 0.0017 (5) |
| N2 | 0.0137 (6) | 0.0141 (6) | 0.0142 (6) | 0.0011 (5) | 0.0057 (5) | 0.0000 (5) |
| N3 | 0.0130 (6) | 0.0124 (6) | 0.0141 (6) | 0.0006 (5) | 0.0031 (5) | 0.0008 (5) |
| C1 | 0.0187 (8) | 0.0185 (8) | 0.0129 (7) | -0.0017 (6) | 0.0035 (6) | -0.0002 (6) |
| C2 | 0.0156 (8) | 0.0160 (8) | 0.0200 (8) | -0.0025 (6) | 0.0034 (6) | -0.0032 (6) |
| C3 | 0.0148 (8) | 0.0179 (8) | 0.0192 (8) | 0.0012 (6) | 0.0033 (6) | 0.0029 (6) |
| C4 | 0.0162 (8) | 0.0169 (8) | 0.0277 (9) | 0.0037 (6) | 0.0075 (7) | 0.0034 (7) |
| C5 | 0.0247 (9) | 0.0166 (8) | 0.0246 (9) | 0.0028 (7) | 0.0127 (7) | -0.0016 (7) |
| C6 | 0.0225 (8) | 0.0194 (8) | 0.0154 (8) | 0.0014 (7) | 0.0057 (6) | -0.0003 (6) |
| C7 | 0.0157 (8) | 0.0130 (7) | 0.0146 (7) | -0.0005 (6) | 0.0042 (6) | 0.0015 (6) |
| C8 | 0.0168 (8) | 0.0192 (8) | 0.0121 (7) | 0.0028 (6) | 0.0033 (6) | 0.0008 (6) |
| C9 | 0.0166 (8) | 0.0172 (8) | 0.0181 (8) | -0.0044 (6) | -0.0015 (6) | 0.0016 (6) |
| C10 | 0.0157 (8) | 0.0139 (7) | 0.0154 (7) | 0.0000 (6) | 0.0061 (6) | -0.0025 (6) |
| C11 | 0.0218 (8) | 0.0178 (8) | 0.0240 (9) | -0.0043 (7) | 0.0051 (7) | -0.0031 (7) |
| C12 | 0.0312 (10) | 0.0140 (8) | 0.0350 (10) | -0.0048 (7) | 0.0095 (8) | 0.0024 (7) |
| C13 | 0.0251 (9) | 0.0176 (8) | 0.0302 (9) | 0.0030 (7) | 0.0076 (7) | 0.0091 (7) |
| C14 | 0.0176 (8) | 0.0189 (8) | 0.0189 (8) | 0.0023 (6) | 0.0046 (6) | 0.0041 (7) |
| C15 | 0.0143 (7) | 0.0133 (7) | 0.0159 (7) | 0.0018 (6) | 0.0010 (6) | -0.0016 (6) |
| C16 | 0.0146 (7) | 0.0097 (7) | 0.0161 (7) | 0.0027 (6) | 0.0011 (6) | -0.0014 (6) |
| C17 | 0.0134 (7) | 0.0109 (7) | 0.0213 (8) | 0.0000 (6) | 0.0032 (6) | 0.0012 (6) |

| | | | | | | |
|---|---|---|---|---|---|---|
| C18 | 0.0189 (8) | 0.0124 (7) | 0.0161 (8) | 0.0020 (6) | 0.0059 (6) | 0.0025 (6) |
| C19 | 0.0169 (8) | 0.0108 (7) | 0.0154 (7) | 0.0023 (6) | 0.0022 (6) | 0.0003 (6) |
| C20 | 0.0189 (8) | 0.0123 (8) | 0.0164 (8) | 0.0027 (6) | 0.0006 (6) | -0.0005 (6) |
| C21 | 0.0254 (9) | 0.0199 (9) | 0.0180 (8) | 0.0041 (7) | 0.0026 (7) | 0.0019 (7) |
| C22 | 0.0332 (10) | 0.0245 (9) | 0.0154 (8) | 0.0060 (8) | -0.0035 (7) | -0.0005 (7) |
| C23 | 0.0244 (9) | 0.0237 (9) | 0.0238 (9) | 0.0013 (7) | -0.0093 (7) | -0.0029 (7) |
| C24 | 0.0184 (8) | 0.0214 (9) | 0.0250 (9) | -0.0013 (7) | -0.0022 (7) | 0.0003 (7) |
| C25 | 0.0182 (8) | 0.0158 (8) | 0.0187 (8) | 0.0012 (6) | 0.0004 (6) | 0.0004 (6) |
| C26 | 0.0145 (8) | 0.0178 (8) | 0.0187 (8) | 0.0003 (6) | 0.0011 (6) | 0.0013 (6) |
| C27 | 0.0145 (8) | 0.0127 (7) | 0.0162 (8) | 0.0006 (6) | 0.0028 (6) | -0.0001 (6) |
| C28 | 0.0156 (7) | 0.0138 (7) | 0.0150 (7) | 0.0021 (6) | 0.0038 (6) | -0.0003 (6) |

*Geometric parameters (Å, º) for (hh08_full_1_a)*

| | | | |
|---|---|---|---|
| Ni1—N1 | 1.9514 (13) | C10—C11 | 1.394 (2) |
| Ni1—N2 | 1.9581 (13) | C11—H11 | 0.9500 |
| Ni1—N3 | 2.4306 (13) | C11—C12 | 1.377 (3) |
| Ni1—C1 | 1.8863 (16) | C12—H12 | 0.9500 |
| Ni1—C2 | 1.8886 (16) | C12—C13 | 1.386 (3) |
| F1—C1 | 1.3685 (19) | C13—H13 | 0.9500 |
| F2—C1 | 1.3943 (19) | C13—C14 | 1.378 (2) |
| F3—C1 | 1.3599 (19) | C14—H14 | 0.9500 |
| F4—C2 | 1.3661 (19) | C15—H15A | 0.9900 |
| F5—C2 | 1.3616 (19) | C15—H15B | 0.9900 |
| F6—C2 | 1.3763 (19) | C15—C16 | 1.522 (2) |
| O1—C26 | 1.216 (2) | C16—C17 | 1.400 (2) |
| N1—C3 | 1.347 (2) | C16—C28 | 1.401 (2) |
| N1—C7 | 1.345 (2) | C17—H17 | 0.9500 |
| N2—C10 | 1.339 (2) | C17—C18 | 1.396 (2) |
| N2—C14 | 1.348 (2) | C18—H18 | 0.9500 |
| N3—C8 | 1.4702 (19) | C18—C19 | 1.380 (2) |
| N3—C9 | 1.461 (2) | C19—C20 | 1.484 (2) |
| N3—C15 | 1.4821 (19) | C19—C27 | 1.403 (2) |
| C3—H3 | 0.9500 | C20—C21 | 1.385 (2) |
| C3—C4 | 1.382 (2) | C20—C25 | 1.402 (2) |
| C4—H4 | 0.9500 | C21—H21 | 0.9500 |
| C4—C5 | 1.384 (2) | C21—C22 | 1.399 (2) |
| C5—H5 | 0.9500 | C22—H22 | 0.9500 |

| | | | |
|---|---|---|---|
| C5—C6 | 1.388 (2) | C22—C23 | 1.390 (3) |
| C6—H6 | 0.9500 | C23—H23 | 0.9500 |
| C6—C7 | 1.388 (2) | C23—C24 | 1.393 (3) |
| C7—C8 | 1.508 (2) | C24—H24 | 0.9500 |
| C8—H8A | 0.9900 | C24—C25 | 1.381 (2) |
| C8—H8B | 0.9900 | C25—C26 | 1.498 (2) |
| C9—H9A | 0.9900 | C26—C27 | 1.494 (2) |
| C9—H9B | 0.9900 | C27—C28 | 1.384 (2) |
| C9—C10 | 1.510 (2) | C28—H28 | 0.9500 |
| | | | |
| N1—Ni1—N2 | 85.16 (5) | N2—C10—C9 | 118.72 (14) |
| N1—Ni1—N3 | 79.47 (5) | N2—C10—C11 | 121.06 (15) |
| N2—Ni1—N3 | 78.47 (5) | C11—C10—C9 | 120.11 (14) |
| C1—Ni1—N1 | 91.50 (6) | C10—C11—H11 | 120.3 |
| C1—Ni1—N2 | 172.32 (6) | C12—C11—C10 | 119.49 (16) |
| C1—Ni1—N3 | 107.75 (6) | C12—C11—H11 | 120.3 |
| C1—Ni1—C2 | 91.47 (7) | C11—C12—H12 | 120.4 |
| C2—Ni1—N1 | 176.58 (6) | C11—C12—C13 | 119.29 (16) |
| C2—Ni1—N2 | 91.69 (6) | C13—C12—H12 | 120.4 |
| C2—Ni1—N3 | 101.21 (6) | C12—C13—H13 | 120.8 |
| C3—N1—Ni1 | 120.54 (11) | C14—C13—C12 | 118.45 (16) |
| C7—N1—Ni1 | 119.49 (10) | C14—C13—H13 | 120.8 |
| C7—N1—C3 | 118.80 (13) | N2—C14—C13 | 122.48 (16) |
| C10—N2—Ni1 | 121.73 (11) | N2—C14—H14 | 118.8 |
| C10—N2—C14 | 119.21 (14) | C13—C14—H14 | 118.8 |
| C14—N2—Ni1 | 118.99 (11) | N3—C15—H15A | 108.6 |
| C8—N3—Ni1 | 99.68 (9) | N3—C15—H15B | 108.6 |
| C8—N3—C15 | 111.57 (12) | N3—C15—C16 | 114.63 (12) |
| C9—N3—Ni1 | 103.58 (9) | H15A—C15—H15B | 107.6 |
| C9—N3—C8 | 113.62 (12) | C16—C15—H15A | 108.6 |
| C9—N3—C15 | 111.86 (12) | C16—C15—H15B | 108.6 |
| C15—N3—Ni1 | 115.83 (9) | C17—C16—C15 | 120.12 (14) |
| F1—C1—Ni1 | 111.97 (10) | C17—C16—C28 | 118.81 (14) |
| F1—C1—F2 | 102.30 (12) | C28—C16—C15 | 121.03 (14) |
| F2—C1—Ni1 | 113.25 (11) | C16—C17—H17 | 118.9 |
| F3—C1—Ni1 | 121.52 (11) | C18—C17—C16 | 122.15 (14) |
| F3—C1—F1 | 104.67 (13) | C18—C17—H17 | 118.9 |

| | | | |
|---|---|---|---|
| F3—C1—F2 | 100.92 (12) | C17—C18—H18 | 120.8 |
| F4—C2—Ni1 | 116.57 (11) | C19—C18—C17 | 118.37 (14) |
| F4—C2—F6 | 101.67 (13) | C19—C18—H18 | 120.8 |
| F5—C2—Ni1 | 116.89 (11) | C18—C19—C20 | 131.11 (14) |
| F5—C2—F4 | 103.11 (13) | C18—C19—C27 | 120.06 (14) |
| F5—C2—F6 | 102.44 (14) | C27—C19—C20 | 108.74 (14) |
| F6—C2—Ni1 | 114.04 (11) | C21—C20—C19 | 131.19 (15) |
| N1—C3—H3 | 118.7 | C21—C20—C25 | 120.34 (15) |
| N1—C3—C4 | 122.68 (15) | C25—C20—C19 | 108.36 (14) |
| C4—C3—H3 | 118.7 | C20—C21—H21 | 120.9 |
| C3—C4—H4 | 120.7 | C20—C21—C22 | 118.13 (16) |
| C3—C4—C5 | 118.69 (15) | C22—C21—H21 | 120.9 |
| C5—C4—H4 | 120.7 | C21—C22—H22 | 119.5 |
| C4—C5—H5 | 120.6 | C23—C22—C21 | 121.04 (16) |
| C4—C5—C6 | 118.81 (15) | C23—C22—H22 | 119.5 |
| C6—C5—H5 | 120.6 | C22—C23—H23 | 119.5 |
| C5—C6—H6 | 120.2 | C22—C23—C24 | 120.93 (16) |
| C7—C6—C5 | 119.66 (15) | C24—C23—H23 | 119.5 |
| C7—C6—H6 | 120.2 | C23—C24—H24 | 121.1 |
| N1—C7—C6 | 121.34 (14) | C25—C24—C23 | 117.88 (16) |
| N1—C7—C8 | 117.11 (13) | C25—C24—H24 | 121.1 |
| C6—C7—C8 | 121.42 (14) | C20—C25—C26 | 108.90 (14) |
| N3—C8—C7 | 113.43 (12) | C24—C25—C20 | 121.64 (15) |
| N3—C8—H8A | 108.9 | C24—C25—C26 | 129.36 (16) |
| N3—C8—H8B | 108.9 | O1—C26—C25 | 127.39 (15) |
| C7—C8—H8A | 108.9 | O1—C26—C27 | 127.45 (15) |
| C7—C8—H8B | 108.9 | C27—C26—C25 | 105.16 (13) |
| H8A—C8—H8B | 107.7 | C19—C27—C26 | 108.75 (14) |
| N3—C9—H9A | 108.5 | C28—C27—C19 | 121.59 (14) |
| N3—C9—H9B | 108.5 | C28—C27—C26 | 129.57 (14) |
| N3—C9—C10 | 115.15 (13) | C16—C28—H28 | 120.5 |
| H9A—C9—H9B | 107.5 | C27—C28—C16 | 118.96 (14) |
| C10—C9—H9A | 108.5 | C27—C28—H28 | 120.5 |
| C10—C9—H9B | 108.5 | | |
| | | | |
| Ni1—N1—C3—C4 | 166.42 (12) | C9—N3—C8—C7 | -77.12 (16) |
| Ni1—N1—C7—C6 | -167.71 (12) | C9—N3—C15—C16 | -63.20 (17) |

| | | | |
|---|---|---|---|
| Ni1—N1—C7—C8 | 16.40 (18) | C9—C10—C11—C12 | -176.75 (16) |
| Ni1—N2—C10—C9 | -5.31 (19) | C10—N2—C14—C13 | -1.2 (2) |
| Ni1—N2—C10—C11 | 178.46 (12) | C10—C11—C12—C13 | -0.5 (3) |
| Ni1—N2—C14—C13 | -178.36 (13) | C11—C12—C13—C14 | 0.7 (3) |
| Ni1—N3—C8—C7 | 32.43 (14) | C12—C13—C14—N2 | 0.2 (3) |
| Ni1—N3—C9—C10 | -15.85 (15) | C14—N2—C10—C9 | 177.65 (14) |
| Ni1—N3—C15—C16 | 178.42 (10) | C14—N2—C10—C11 | 1.4 (2) |
| O1—C26—C27—C19 | -176.98 (17) | C15—N3—C8—C7 | 155.30 (13) |
| O1—C26—C27—C28 | 6.4 (3) | C15—N3—C9—C10 | -141.29 (14) |
| N1—Ni1—C1—F1 | 73.91 (11) | C15—C16—C17—C18 | 175.60 (14) |
| N1—Ni1—C1—F2 | -41.13 (11) | C15—C16—C28—C27 | -176.32 (14) |
| N1—Ni1—C1—F3 | -161.49 (13) | C16—C17—C18—C19 | 1.0 (2) |
| N1—C3—C4—C5 | 1.1 (2) | C17—C16—C28—C27 | 1.6 (2) |
| N1—C7—C8—N3 | -36.68 (19) | C17—C18—C19—C20 | -175.02 (15) |
| N2—Ni1—C2—F4 | -137.32 (12) | C17—C18—C19—C27 | 1.1 (2) |
| N2—Ni1—C2—F5 | 100.13 (12) | C18—C19—C20—C21 | 0.4 (3) |
| N2—Ni1—C2—F6 | -19.25 (14) | C18—C19—C20—C25 | 176.55 (16) |
| N2—C10—C11—C12 | -0.6 (2) | C18—C19—C27—C26 | -178.80 (14) |
| N3—Ni1—C1—F1 | -5.51 (12) | C18—C19—C27—C28 | -1.9 (2) |
| N3—Ni1—C1—F2 | -120.55 (10) | C19—C20—C21—C22 | 173.80 (16) |
| N3—Ni1—C1—F3 | 119.09 (13) | C19—C20—C25—C24 | -174.93 (15) |
| N3—Ni1—C2—F4 | -58.75 (12) | C19—C20—C25—C26 | 1.77 (18) |
| N3—Ni1—C2—F5 | 178.71 (11) | C19—C27—C28—C16 | 0.4 (2) |
| N3—Ni1—C2—F6 | 59.33 (14) | C20—C19—C27—C26 | -1.84 (17) |
| N3—C9—C10—N2 | 16.0 (2) | C20—C19—C27—C28 | 175.09 (14) |

| | | | |
|---|---|---|---|
| N3—C9—C10—C11 | -167.71 (14) | C20—C21—C22—C23 | 0.6 (3) |
| N3—C15—C16—C17 | -83.34 (18) | C20—C25—C26—O1 | 177.00 (17) |
| N3—C15—C16—C28 | 94.57 (17) | C20—C25—C26—C27 | -2.81 (17) |
| C1—Ni1—C2—F4 | 49.67 (13) | C21—C20—C25—C24 | 1.7 (2) |
| C1—Ni1—C2—F5 | -72.87 (13) | C21—C20—C25—C26 | 178.39 (15) |
| C1—Ni1—C2—F6 | 167.75 (14) | C21—C22—C23—C24 | 1.1 (3) |
| C2—Ni1—C1—F1 | -107.77 (12) | C22—C23—C24—C25 | -1.3 (3) |
| C2—Ni1—C1—F2 | 137.19 (11) | C23—C24—C25—C20 | 0.0 (3) |
| C2—Ni1—C1—F3 | 16.83 (14) | C23—C24—C25—C26 | -175.98 (16) |
| C3—N1—C7—C6 | 0.0 (2) | C24—C25—C26—O1 | -6.6 (3) |
| C3—N1—C7—C8 | -175.89 (14) | C24—C25—C26—C27 | 173.56 (17) |
| C3—C4—C5—C6 | 0.2 (2) | C25—C20—C21—C22 | -1.9 (2) |
| C4—C5—C6—C7 | -1.3 (2) | C25—C26—C27—C19 | 2.83 (17) |
| C5—C6—C7—N1 | 1.2 (2) | C25—C26—C27—C28 | -173.78 (16) |
| C5—C6—C7—C8 | 176.94 (15) | C26—C27—C28—C16 | 176.66 (15) |
| C6—C7—C8—N3 | 147.43 (15) | C27—C19—C20—C21 | -176.08 (17) |
| C7—N1—C3—C4 | -1.2 (2) | C27—C19—C20—C25 | 0.05 (18) |
| C8—N3—C9—C10 | 91.28 (16) | C28—C16—C17—C18 | -2.4 (2) |
| C8—N3—C15—C16 | 65.32 (16) | | |

Document origin: *publCIF* [Westrip, S. P. (2010). *J. Apply. Cryst.*, **43**, 920-925].

# [($^{Fluor}$N$_3$)Ni(CF$_3$)$_2$(MeCN)]BF$_4$

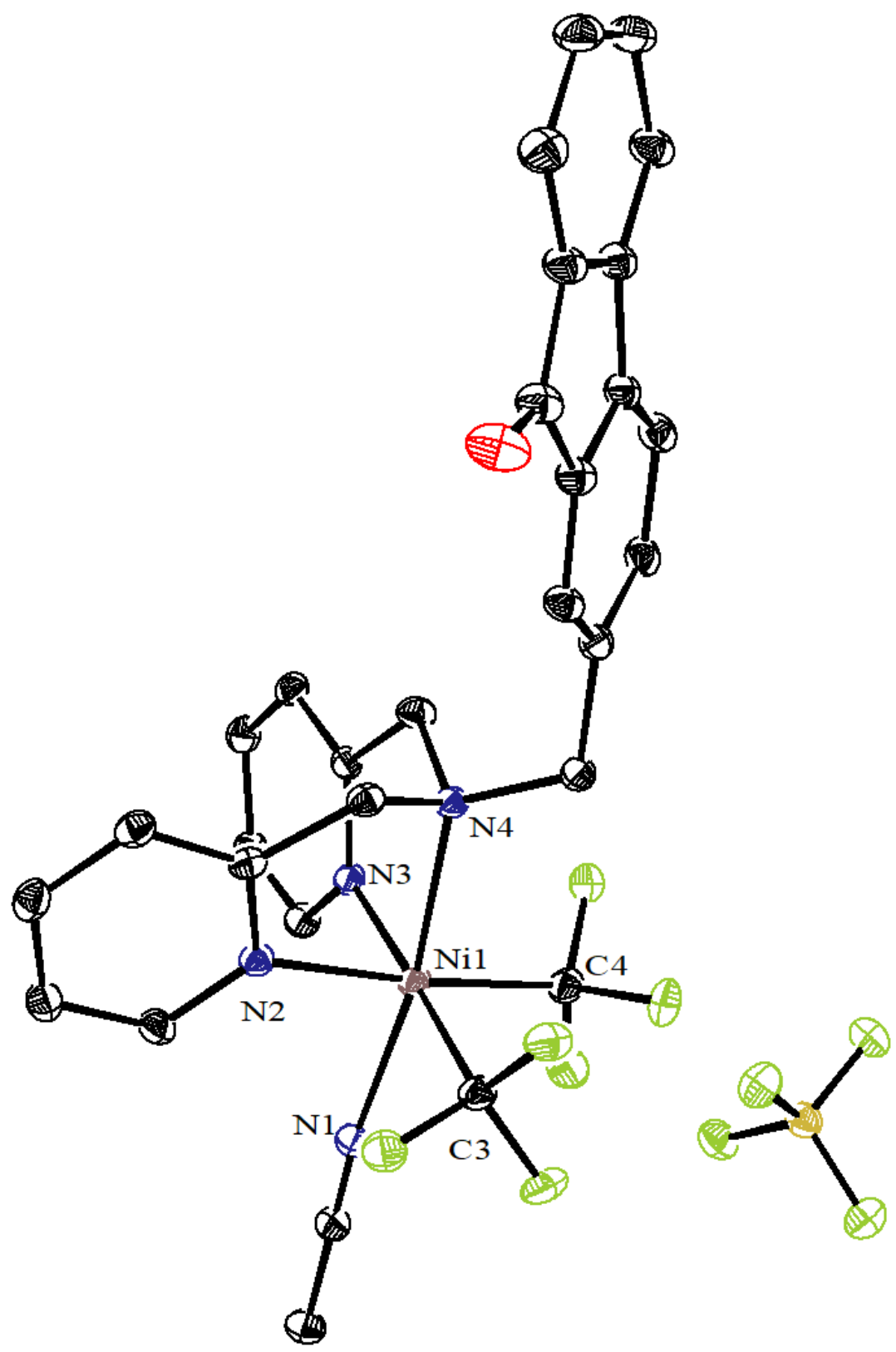


XRD crystal structure for **[($^{Fluor}$N$_3$)Ni(CF$_3$)$_2$(MeCN)]BF$_4$**. Hydrogens omitted for clarity. Ellipsoids presented at 30% probability.

### Refinement

Crystal data, data collection and structure refinement details are summarized in Table S6.

### Results and discussion

### Computing details

Data collection: *CrysAlis PRO* 1.171.43.143a (Rigaku OD, 2024); cell refinement: *CrysAlis PRO* 1.171.43.143a (Rigaku OD, 2024); data reduction: *CrysAlis PRO* 1.171.43.143a (Rigaku OD, 2024); program(s) used to solve structure: SHELXT 2018/2 (Sheldrick, 2018); program(s) used to refine structure: *SHELXL* 2019/3 (Sheldrick, 2015); molecular graphics: Olex2 1.5 (Dolomanov *et al.*, 2009);

software used to prepare material for publication: Olex2 1.5 (Dolomanov *et al.*, 2009).

## References


Dolomanov, O. V., Bourhis, L. J., Gildea, R. J., Howard, J. A. K. & Puschmann, H. (2009). *J. Appl. Cryst.* **42**, 339–341.

Sheldrick, G. M. (2015). *Acta Cryst.* A**71**, 3–8.

Sheldrick, G. M. (2015). *Acta Cryst.* C**71**, 3–8.


## Table S6

### *Crystal data*

| | |
|---|---|
| $C_{30}H_{24}F_6N_4NiO\cdot BF_4$ | $Z = 2$ |
| $M_r = 716.05$ | $F(000) = 726$ |
| Triclinic, $P\bar{1}$ | $D_x = 1.605$ Mg m$^{-3}$ |
| $a = 8.1320$ (2) Å | Cu $K\alpha$ radiation, $\lambda = 1.54184$ Å |
| $b = 10.3778$ (3) Å | Cell parameters from 10948 reflections |
| $c = 18.6281$ (5) Å | $\theta = 4.6–75.0°$ |
| $\alpha = 99.779$ (2)° | $\mu = 1.82$ mm$^{-1}$ |
| $\beta = 94.341$ (2)° | $T = 100$ K |
| $\gamma = 105.398$ (2)° | Rect. Prism, clear dark violet |
| $V = 1481.49$ (7) Å$^3$ | 0.09 × 0.07 × 0.06 mm |

### *Data collection*

| | |
|---|---|
| XtaLAB Synergy, Dualflex, HyPix-Arc 150 diffractometer | 5829 independent reflections |
| Radiation source: micro-focus sealed X-ray tube, PhotonJet (Cu) X-ray Source | 4967 reflections with $I > 2\sigma(I)$ |
| Mirror monochromator | $R_{int} = 0.025$ |
| Detector resolution: 10.0000 pixels mm$^{-1}$ | $\theta_{max} = 75.7°$, $\theta_{min} = 2.4°$ |
| ω scans | $h = -9\rightarrow9$ |
| Absorption correction: multi-scan *CrysAlis PRO* 1.171.43.143a (Rigaku Oxford Diffraction, 2024) Empirical absorption correction using spherical harmonics, implemented in SCALE3 ABSPACK scaling algorithm. | $k = -12\rightarrow12$ |
| $T_{min} = 0.846$, $T_{max} = 1.000$ | $l = -21\rightarrow23$ |
| 16845 measured reflections | |

*Refinement*

| | |
|---|---|
| Refinement on $F^2$ | Primary atom site location: dual |
| Least-squares matrix: full | Hydrogen site location: inferred from neighbouring sites |
| $R[F^2 > 2\sigma(F^2)] = 0.037$ | H-atom parameters constrained |
| $wR(F^2) = 0.100$ | $w = 1/[\sigma^2(F_o^2) + (0.0497P)^2 + 0.6383P]$ where $P = (F_o^2 + 2F_c^2)/3$ |
| $S = 1.07$ | $(\Delta/\sigma)_{max} = 0.001$ |
| 5829 reflections | $\Delta\rangle_{max} = 0.82$ e Å$^{-3}$ |
| 425 parameters | $\Delta\rangle_{min} = -0.37$ e Å$^{-3}$ |
| 0 restraints | |

*Special details*

| |
|---|
| *Geometry*. All esds (except the esd in the dihedral angle between two l.s. planes) are estimated using the full covariance matrix. The cell esds are taken into account individually in the estimation of esds in distances, angles and torsion angles; correlations between esds in cell parameters are only used when they are defined by crystal symmetry. An approximate (isotropic) treatment of cell esds is used for estimating esds involving l.s. planes. |

*Fractional atomic coordinates and isotropic or equivalent isotropic displacement parameters (Å$^2$) for (ayg004_auto)*

| | *x* | *y* | *z* | $U_{iso}$*/$U_{eq}$ |
|---|---|---|---|---|
| Ni1 | 1.09082 (4) | 0.62701 (3) | 0.78602 (2) | 0.02233 (10) |
| F1 | 1.36277 (15) | 0.61311 (14) | 0.70234 (7) | 0.0388 (3) |
| O1 | 0.2423 (2) | 0.40107 (19) | 0.48212 (9) | 0.0474 (4) |
| N1 | 1.3359 (2) | 0.75536 (17) | 0.84553 (9) | 0.0287 (4) |
| C1 | 1.4694 (3) | 0.8125 (2) | 0.87582 (11) | 0.0269 (4) |
| F2 | 1.11863 (15) | 0.56239 (14) | 0.63523 (6) | 0.0374 (3) |
| N2 | 1.0049 (2) | 0.78894 (16) | 0.77812 (8) | 0.0238 (3) |
| C2 | 1.6388 (3) | 0.8856 (2) | 0.91578 (11) | 0.0309 (4) |
| H2A | 1.722131 | 0.836944 | 0.900236 | 0.046* |
| H2B | 1.634082 | 0.891292 | 0.968600 | 0.046* |
| H2C | 1.673956 | 0.977778 | 0.905358 | 0.046* |
| F3 | 1.24767 (17) | 0.77092 (13) | 0.68621 (7) | 0.0390 (3) |
| N3 | 0.9824 (2) | 0.61940 (16) | 0.87805 (8) | 0.0227 (3) |
| C3 | 1.2088 (2) | 0.6411 (2) | 0.69872 (11) | 0.0288 (4) |

| | | | | |
|---|---|---|---|---|
| F4 | 1.30057 (16) | 0.48321 (13) | 0.83495 (8) | 0.0410 (3) |
| N4 | 0.82253 (19) | 0.51702 (16) | 0.73382 (8) | 0.0228 (3) |
| C4 | 1.1525 (3) | 0.4594 (2) | 0.79147 (11) | 0.0283 (4) |
| F5 | 1.16677 (18) | 0.38171 (13) | 0.72764 (7) | 0.0397 (3) |
| C5 | 0.8104 (2) | 0.56674 (18) | 0.87035 (10) | 0.0225 (4) |
| F6 | 1.03180 (15) | 0.37312 (11) | 0.82141 (7) | 0.0327 (3) |
| C6 | 0.7183 (3) | 0.5718 (2) | 0.92989 (10) | 0.0258 (4) |
| H6 | 0.596601 | 0.536503 | 0.922900 | 0.031* |
| C7 | 0.8055 (3) | 0.6287 (2) | 0.99975 (11) | 0.0293 (4) |
| H7 | 0.744814 | 0.633515 | 1.041287 | 0.035* |
| C8 | 0.9831 (3) | 0.6786 (2) | 1.00766 (11) | 0.0301 (4) |
| H8 | 1.046364 | 0.716074 | 1.055016 | 0.036* |
| C9 | 1.0669 (3) | 0.6733 (2) | 0.94630 (10) | 0.0276 (4) |
| H9 | 1.188523 | 0.708813 | 0.952129 | 0.033* |
| C10 | 0.7211 (2) | 0.4894 (2) | 0.79574 (10) | 0.0271 (4) |
| H10A | 0.692351 | 0.390612 | 0.795928 | 0.033* |
| H10B | 0.611677 | 0.512329 | 0.786776 | 0.033* |
| C11 | 1.0868 (3) | 0.9189 (2) | 0.81202 (11) | 0.0283 (4) |
| H11 | 1.198363 | 0.938129 | 0.838064 | 0.034* |
| C12 | 1.0141 (3) | 1.0246 (2) | 0.81013 (11) | 0.0312 (4) |
| H12 | 1.075280 | 1.115087 | 0.833820 | 0.037* |
| C13 | 0.8507 (3) | 0.9962 (2) | 0.77313 (12) | 0.0322 (5) |
| H13 | 0.797062 | 1.066836 | 0.771618 | 0.039* |
| C14 | 0.7663 (3) | 0.8635 (2) | 0.73834 (11) | 0.0296 (4) |
| H14 | 0.653476 | 0.841838 | 0.713135 | 0.035* |
| C15 | 0.8479 (2) | 0.7620 (2) | 0.74055 (10) | 0.0237 (4) |
| C16 | 0.7728 (2) | 0.6201 (2) | 0.69727 (10) | 0.0249 (4) |
| H16A | 0.645870 | 0.599100 | 0.690392 | 0.030* |
| H16B | 0.812051 | 0.614439 | 0.648111 | 0.030* |
| C17 | 0.7903 (2) | 0.3860 (2) | 0.67823 (11) | 0.0284 (4) |
| H17A | 0.841050 | 0.322846 | 0.700445 | 0.034* |
| H17B | 0.850577 | 0.406145 | 0.635457 | 0.034* |
| C18 | 0.6021 (3) | 0.3148 (2) | 0.65134 (10) | 0.0273 (4) |
| C19 | 0.5135 (3) | 0.2084 (2) | 0.68293 (11) | 0.0299 (4) |
| H19 | 0.574570 | 0.179916 | 0.719838 | 0.036* |
| C20 | 0.3387 (3) | 0.1422 (2) | 0.66252 (11) | 0.0308 (4) |
| H20 | 0.281601 | 0.069059 | 0.684273 | 0.037* |

| | | | | |
|---|---|---|---|---|
| C21 | 0.2517 (3) | 0.1867 (2) | 0.60963 (10) | 0.0273 (4) |
| C22 | 0.3392 (3) | 0.2917 (2) | 0.57660 (10) | 0.0282 (4) |
| C23 | 0.5132 (3) | 0.3555 (2) | 0.59540 (10) | 0.0284 (4) |
| H23 | 0.571217 | 0.424727 | 0.571282 | 0.034* |
| C24 | 0.2131 (3) | 0.3209 (2) | 0.52325 (11) | 0.0334 (5) |
| C25 | 0.0671 (3) | 0.1462 (2) | 0.58028 (11) | 0.0301 (4) |
| C26 | -0.0713 (3) | 0.0481 (2) | 0.59520 (12) | 0.0365 (5) |
| H26 | -0.055516 | -0.009354 | 0.628171 | 0.044* |
| C27 | -0.2339 (3) | 0.0358 (3) | 0.56074 (13) | 0.0428 (6) |
| H27 | -0.330401 | -0.030445 | 0.570766 | 0.051* |
| C28 | -0.2580 (3) | 0.1182 (3) | 0.51206 (13) | 0.0437 (6) |
| H28 | -0.370611 | 0.107784 | 0.489385 | 0.052* |
| C29 | -0.1199 (3) | 0.2160 (3) | 0.49586 (12) | 0.0402 (5) |
| H29 | -0.135799 | 0.272538 | 0.462366 | 0.048* |
| C30 | 0.0428 (3) | 0.2279 (2) | 0.53065 (11) | 0.0322 (5) |
| B1 | 1.5150 (3) | 0.2074 (2) | 0.90304 (13) | 0.0317 (5) |
| F7 | 1.41470 (17) | 0.25930 (14) | 0.85992 (7) | 0.0418 (3) |
| F8 | 1.42251 (17) | 0.07907 (13) | 0.91263 (7) | 0.0416 (3) |
| F9 | 1.66186 (17) | 0.19898 (14) | 0.87010 (7) | 0.0427 (3) |
| F10 | 1.56383 (17) | 0.29234 (13) | 0.97251 (7) | 0.0401 (3) |

*Atomic displacement parameters (Å$^2$) for (ayg004_auto)*

| | $U^{11}$ | $U^{22}$ | $U^{33}$ | $U^{12}$ | $U^{13}$ | $U^{23}$ |
|---|---|---|---|---|---|---|
| Ni1 | 0.01801 (17) | 0.02495 (17) | 0.02240 (17) | 0.00645 (13) | -0.00009 (12) | 0.00082 (13) |
| F1 | 0.0219 (6) | 0.0553 (8) | 0.0405 (7) | 0.0144 (6) | 0.0076 (5) | 0.0063 (6) |
| O1 | 0.0412 (10) | 0.0597 (11) | 0.0346 (9) | -0.0003 (8) | -0.0053 (7) | 0.0189 (8) |
| N1 | 0.0250 (9) | 0.0283 (8) | 0.0299 (9) | 0.0053 (7) | 0.0004 (7) | 0.0027 (7) |
| C1 | 0.0268 (10) | 0.0266 (10) | 0.0270 (10) | 0.0071 (8) | 0.0030 (8) | 0.0056 (8) |
| F2 | 0.0281 (6) | 0.0509 (8) | 0.0249 (6) | 0.0031 (5) | 0.0029 (5) | -0.0021 (5) |
| N2 | 0.0209 (8) | 0.0263 (8) | 0.0232 (8) | 0.0066 (6) | 0.0009 (6) | 0.0030 (6) |
| C2 | 0.0259 (10) | 0.0329 (10) | 0.0310 (10) | 0.0036 (8) | -0.0007 (8) | 0.0074 (8) |
| F3 | 0.0411 (7) | 0.0380 (7) | 0.0391 (7) | 0.0082 (6) | 0.0148 (6) | 0.0110 (6) |
| N3 | 0.0206 (8) | 0.0238 (8) | 0.0225 (8) | 0.0072 (6) | 0.0000 (6) | 0.0007 (6) |
| C3 | 0.0204 (9) | 0.0337 (10) | 0.0289 (10) | 0.0065 (8) | 0.0002 (8) | 0.0002 (8) |
| F4 | 0.0285 (6) | 0.0365 (7) | 0.0557 (8) | 0.0118 (5) | -0.0125 (6) | 0.0069 (6) |
| N4 | 0.0191 (8) | 0.0244 (8) | 0.0221 (7) | 0.0048 (6) | 0.0004 (6) | 0.0006 (6) |

| | | | | | | |
|---|---|---|---|---|---|---|
| C4 | 0.0240 (10) | 0.0318 (10) | 0.0277 (10) | 0.0097 (8) | -0.0005 (8) | 0.0007 (8) |
| F5 | 0.0479 (8) | 0.0355 (7) | 0.0386 (7) | 0.0208 (6) | 0.0113 (6) | -0.0016 (5) |
| C5 | 0.0220 (9) | 0.0197 (8) | 0.0246 (9) | 0.0058 (7) | -0.0005 (7) | 0.0031 (7) |
| F6 | 0.0330 (6) | 0.0267 (6) | 0.0391 (7) | 0.0102 (5) | 0.0054 (5) | 0.0053 (5) |
| C6 | 0.0233 (10) | 0.0275 (9) | 0.0275 (10) | 0.0095 (8) | 0.0024 (8) | 0.0048 (8) |
| C7 | 0.0328 (11) | 0.0305 (10) | 0.0250 (10) | 0.0104 (8) | 0.0056 (8) | 0.0034 (8) |
| C8 | 0.0324 (11) | 0.0312 (10) | 0.0224 (9) | 0.0074 (8) | -0.0019 (8) | -0.0017 (8) |
| C9 | 0.0242 (10) | 0.0305 (10) | 0.0248 (9) | 0.0070 (8) | -0.0032 (8) | -0.0001 (8) |
| C10 | 0.0214 (9) | 0.0315 (10) | 0.0238 (9) | 0.0020 (8) | 0.0002 (7) | 0.0030 (8) |
| C11 | 0.0253 (10) | 0.0251 (9) | 0.0304 (10) | 0.0031 (8) | -0.0004 (8) | 0.0023 (8) |
| C12 | 0.0359 (11) | 0.0260 (10) | 0.0320 (10) | 0.0087 (8) | 0.0064 (9) | 0.0058 (8) |
| C13 | 0.0360 (12) | 0.0319 (11) | 0.0353 (11) | 0.0168 (9) | 0.0086 (9) | 0.0111 (9) |
| C14 | 0.0254 (10) | 0.0352 (11) | 0.0314 (10) | 0.0119 (8) | 0.0038 (8) | 0.0103 (8) |
| C15 | 0.0196 (9) | 0.0305 (10) | 0.0211 (9) | 0.0065 (7) | 0.0036 (7) | 0.0058 (7) |
| C16 | 0.0187 (9) | 0.0311 (10) | 0.0233 (9) | 0.0067 (7) | -0.0021 (7) | 0.0038 (8) |
| C17 | 0.0221 (10) | 0.0293 (10) | 0.0283 (10) | 0.0061 (8) | -0.0012 (8) | -0.0052 (8) |
| C18 | 0.0238 (10) | 0.0283 (10) | 0.0246 (9) | 0.0055 (8) | 0.0002 (8) | -0.0045 (8) |
| C19 | 0.0309 (11) | 0.0272 (10) | 0.0280 (10) | 0.0079 (8) | -0.0025 (8) | -0.0010 (8) |
| C20 | 0.0333 (11) | 0.0281 (10) | 0.0262 (10) | 0.0031 (8) | 0.0033 (8) | 0.0011 (8) |
| C21 | 0.0262 (10) | 0.0286 (10) | 0.0210 (9) | 0.0031 (8) | 0.0025 (8) | -0.0039 (7) |
| C22 | 0.0275 (10) | 0.0334 (10) | 0.0190 (9) | 0.0045 (8) | 0.0007 (8) | 0.0001 (8) |
| C23 | 0.0264 (10) | 0.0309 (10) | 0.0217 (9) | 0.0011 (8) | 0.0014 (8) | 0.0000 (8) |
| C24 | 0.0305 (11) | 0.0404 (12) | 0.0226 (10) | 0.0029 (9) | -0.0018 (8) | 0.0016 (9) |
| C25 | 0.0257 (10) | 0.0338 (10) | 0.0224 (9) | 0.0021 (8) | 0.0012 (8) | -0.0061 (8) |
| C26 | 0.0325 (11) | 0.0392 (12) | 0.0285 (10) | -0.0004 (9) | 0.0056 (9) | -0.0025 (9) |
| C27 | 0.0268 (11) | 0.0493 (14) | 0.0389 (12) | -0.0021 (10) | 0.0072 (9) | -0.0080 (11) |
| C28 | 0.0233 (11) | 0.0547 (15) | 0.0409 (13) | 0.0043 (10) | -0.0022 (9) | -0.0102 (11) |
| C29 | 0.0341 (12) | 0.0507 (14) | 0.0302 (11) | 0.0101 (10) | -0.0040 (9) | -0.0011 (10) |
| C30 | 0.0269 (11) | 0.0384 (11) | 0.0235 (10) | 0.0026 (9) | -0.0002 (8) | -0.0030 (8) |
| B1 | 0.0298 (12) | 0.0298 (12) | 0.0340 (12) | 0.0066 (9) | 0.0002 (10) | 0.0071 (10) |
| F7 | 0.0427 (8) | 0.0503 (8) | 0.0394 (7) | 0.0232 (6) | 0.0021 (6) | 0.0132 (6) |
| F8 | 0.0407 (7) | 0.0313 (6) | 0.0467 (8) | 0.0019 (5) | 0.0021 (6) | 0.0058 (6) |
| F9 | 0.0373 (7) | 0.0498 (8) | 0.0453 (8) | 0.0187 (6) | 0.0094 (6) | 0.0089 (6) |
| F10 | 0.0402 (7) | 0.0352 (7) | 0.0376 (7) | 0.0032 (6) | 0.0000 (6) | 0.0015 (5) |

*Geometric parameters (Å, º) for (ayg004_auto)*

| | | | |
|---|---|---|---|
| Ni1—N1 | 2.1750 (17) | C12—H12 | 0.9500 |
| Ni1—N2 | 2.0065 (16) | C12—C13 | 1.383 (3) |
| Ni1—N3 | 1.9899 (16) | C13—H13 | 0.9500 |
| Ni1—C3 | 1.957 (2) | C13—C14 | 1.384 (3) |
| Ni1—N4 | 2.2334 (16) | C14—H14 | 0.9500 |
| Ni1—C4 | 1.950 (2) | C14—C15 | 1.390 (3) |
| F1—C3 | 1.358 (2) | C15—C16 | 1.496 (3) |
| O1—C24 | 1.214 (3) | C16—H16A | 0.9900 |
| N1—C1 | 1.140 (3) | C16—H16B | 0.9900 |
| C1—C2 | 1.457 (3) | C17—H17A | 0.9900 |
| F2—C3 | 1.351 (2) | C17—H17B | 0.9900 |
| N2—C11 | 1.353 (2) | C17—C18 | 1.516 (3) |
| N2—C15 | 1.344 (2) | C18—C19 | 1.395 (3) |
| C2—H2A | 0.9800 | C18—C23 | 1.409 (3) |
| C2—H2B | 0.9800 | C19—H19 | 0.9500 |
| C2—H2C | 0.9800 | C19—C20 | 1.395 (3) |
| F3—C3 | 1.365 (2) | C20—H20 | 0.9500 |
| N3—C5 | 1.346 (2) | C20—C21 | 1.381 (3) |
| N3—C9 | 1.350 (2) | C21—C22 | 1.399 (3) |
| F4—C4 | 1.339 (2) | C21—C25 | 1.480 (3) |
| N4—C10 | 1.491 (2) | C22—C23 | 1.383 (3) |
| N4—C16 | 1.486 (2) | C22—C24 | 1.498 (3) |
| N4—C17 | 1.511 (2) | C23—H23 | 0.9500 |
| C4—F5 | 1.350 (2) | C24—C30 | 1.495 (3) |
| C4—F6 | 1.365 (2) | C25—C26 | 1.384 (3) |
| C5—C6 | 1.386 (3) | C25—C30 | 1.393 (3) |
| C5—C10 | 1.504 (3) | C26—H26 | 0.9500 |
| C6—H6 | 0.9500 | C26—C27 | 1.389 (3) |
| C6—C7 | 1.386 (3) | C27—H27 | 0.9500 |
| C7—H7 | 0.9500 | C27—C28 | 1.385 (4) |
| C7—C8 | 1.386 (3) | C28—H28 | 0.9500 |
| C8—H8 | 0.9500 | C28—C29 | 1.390 (3) |
| C8—C9 | 1.375 (3) | C29—H29 | 0.9500 |
| C9—H9 | 0.9500 | C29—C30 | 1.393 (3) |
| C10—H10A | 0.9900 | B1—F7 | 1.372 (3) |
| C10—H10B | 0.9900 | B1—F8 | 1.393 (3) |
| C11—H11 | 0.9500 | B1—F9 | 1.399 (3) |

| | | | |
|---|---|---|---|
| C11—C12 | 1.381 (3) | B1—F10 | 1.400 (3) |
| | | | |
| N1—Ni1—N4 | 171.73 (6) | C12—C11—H11 | 118.8 |
| N2—Ni1—N1 | 92.13 (7) | C11—C12—H12 | 120.6 |
| N2—Ni1—N4 | 81.22 (6) | C11—C12—C13 | 118.81 (19) |
| N3—Ni1—N1 | 92.20 (6) | C13—C12—H12 | 120.6 |
| N3—Ni1—N2 | 87.14 (6) | C12—C13—H13 | 120.5 |
| N3—Ni1—N4 | 82.62 (6) | C12—C13—C14 | 119.02 (19) |
| C3—Ni1—N1 | 84.71 (7) | C14—C13—H13 | 120.5 |
| C3—Ni1—N2 | 92.84 (8) | C13—C14—H14 | 120.2 |
| C3—Ni1—N3 | 176.90 (7) | C13—C14—C15 | 119.52 (19) |
| C3—Ni1—N4 | 100.44 (7) | C15—C14—H14 | 120.2 |
| C4—Ni1—N1 | 92.89 (7) | N2—C15—C14 | 121.42 (18) |
| C4—Ni1—N2 | 174.76 (8) | N2—C15—C16 | 116.19 (16) |
| C4—Ni1—N3 | 91.15 (8) | C14—C15—C16 | 122.25 (17) |
| C4—Ni1—C3 | 89.14 (9) | N4—C16—C15 | 112.34 (15) |
| C4—Ni1—N4 | 93.65 (7) | N4—C16—H16A | 109.1 |
| C1—N1—Ni1 | 173.89 (16) | N4—C16—H16B | 109.1 |
| N1—C1—C2 | 179.0 (2) | C15—C16—H16A | 109.1 |
| C11—N2—Ni1 | 124.95 (13) | C15—C16—H16B | 109.1 |
| C15—N2—Ni1 | 116.07 (13) | H16A—C16—H16B | 107.9 |
| C15—N2—C11 | 118.78 (17) | N4—C17—H17A | 108.7 |
| C1—C2—H2A | 109.5 | N4—C17—H17B | 108.7 |
| C1—C2—H2B | 109.5 | N4—C17—C18 | 114.37 (16) |
| C1—C2—H2C | 109.5 | H17A—C17—H17B | 107.6 |
| H2A—C2—H2B | 109.5 | C18—C17—H17A | 108.7 |
| H2A—C2—H2C | 109.5 | C18—C17—H17B | 108.7 |
| H2B—C2—H2C | 109.5 | C19—C18—C17 | 119.92 (18) |
| C5—N3—Ni1 | 116.66 (12) | C19—C18—C23 | 118.97 (18) |
| C5—N3—C9 | 118.50 (16) | C23—C18—C17 | 121.11 (19) |
| C9—N3—Ni1 | 124.58 (13) | C18—C19—H19 | 118.7 |
| F1—C3—Ni1 | 115.46 (14) | C18—C19—C20 | 122.67 (19) |
| F1—C3—F3 | 103.77 (15) | C20—C19—H19 | 118.7 |
| F2—C3—Ni1 | 115.47 (13) | C19—C20—H20 | 121.1 |
| F2—C3—F1 | 105.14 (16) | C21—C20—C19 | 117.76 (19) |
| F2—C3—F3 | 104.83 (16) | C21—C20—H20 | 121.1 |
| F3—C3—Ni1 | 111.00 (13) | C20—C21—C22 | 120.26 (19) |

| | | | |
|---|---|---|---|
| C10—N4—Ni1 | 105.58 (11) | C20—C21—C25 | 131.12 (19) |
| C10—N4—C17 | 108.97 (15) | C22—C21—C25 | 108.57 (18) |
| C16—N4—Ni1 | 102.59 (11) | C21—C22—C24 | 108.72 (18) |
| C16—N4—C10 | 111.29 (15) | C23—C22—C21 | 122.17 (19) |
| C16—N4—C17 | 108.99 (14) | C23—C22—C24 | 129.07 (19) |
| C17—N4—Ni1 | 119.24 (11) | C18—C23—H23 | 120.9 |
| F4—C4—Ni1 | 111.93 (13) | C22—C23—C18 | 118.10 (19) |
| F4—C4—F5 | 106.39 (16) | C22—C23—H23 | 120.9 |
| F4—C4—F6 | 105.60 (16) | O1—C24—C22 | 127.8 (2) |
| F5—C4—Ni1 | 117.31 (14) | O1—C24—C30 | 127.3 (2) |
| F5—C4—F6 | 103.66 (16) | C30—C24—C22 | 104.92 (18) |
| F6—C4—Ni1 | 111.02 (13) | C26—C25—C21 | 131.0 (2) |
| N3—C5—C6 | 121.84 (17) | C26—C25—C30 | 120.3 (2) |
| N3—C5—C10 | 117.86 (16) | C30—C25—C21 | 108.72 (18) |
| C6—C5—C10 | 120.05 (17) | C25—C26—H26 | 120.8 |
| C5—C6—H6 | 120.3 | C25—C26—C27 | 118.3 (2) |
| C5—C6—C7 | 119.37 (18) | C27—C26—H26 | 120.8 |
| C7—C6—H6 | 120.3 | C26—C27—H27 | 119.4 |
| C6—C7—H7 | 120.7 | C28—C27—C26 | 121.3 (2) |
| C8—C7—C6 | 118.55 (18) | C28—C27—H27 | 119.4 |
| C8—C7—H7 | 120.7 | C27—C28—H28 | 119.5 |
| C7—C8—H8 | 120.3 | C27—C28—C29 | 121.0 (2) |
| C9—C8—C7 | 119.35 (18) | C29—C28—H28 | 119.5 |
| C9—C8—H8 | 120.3 | C28—C29—H29 | 121.3 |
| N3—C9—C8 | 122.33 (19) | C28—C29—C30 | 117.4 (2) |
| N3—C9—H9 | 118.8 | C30—C29—H29 | 121.3 |
| C8—C9—H9 | 118.8 | C25—C30—C24 | 108.98 (18) |
| N4—C10—C5 | 114.77 (15) | C25—C30—C29 | 121.7 (2) |
| N4—C10—H10A | 108.6 | C29—C30—C24 | 129.3 (2) |
| N4—C10—H10B | 108.6 | F7—B1—F8 | 110.24 (19) |
| C5—C10—H10A | 108.6 | F7—B1—F9 | 109.42 (19) |
| C5—C10—H10B | 108.6 | F7—B1—F10 | 109.72 (18) |
| H10A—C10—H10B | 107.6 | F8—B1—F9 | 110.25 (19) |
| N2—C11—H11 | 118.8 | F8—B1—F10 | 107.66 (18) |
| N2—C11—C12 | 122.39 (19) | F9—B1—F10 | 109.52 (18) |
| | | | |
| Ni1—N2—C11—C12 | -173.84 (15) | C17—N4—C10—C5 | 142.45 (16) |

| | | | |
|---|---|---|---|
| Ni1—N2—C15—C14 | 172.57 (14) | C17—N4—C16—C15 | -162.68 (15) |
| Ni1—N2—C15—C16 | -11.7 (2) | C17—C18—C19—C20 | 177.58 (18) |
| Ni1—N3—C5—C6 | -171.73 (14) | C17—C18—C23—C22 | -176.13 (17) |
| Ni1—N3—C5—C10 | 14.1 (2) | C18—C19—C20—C21 | -1.0 (3) |
| Ni1—N3—C9—C8 | 172.70 (15) | C19—C18—C23—C22 | 2.9 (3) |
| Ni1—N4—C10—C5 | 13.25 (19) | C19—C20—C21—C22 | 2.0 (3) |
| Ni1—N4—C16—C15 | -35.37 (16) | C19—C20—C21—C25 | -174.87 (19) |
| Ni1—N4—C17—C18 | 175.22 (13) | C20—C21—C22—C23 | -0.5 (3) |
| O1—C24—C30—C25 | 176.0 (2) | C20—C21—C22—C24 | -178.59 (18) |
| O1—C24—C30—C29 | -3.5 (4) | C20—C21—C25—C26 | -2.9 (4) |
| N2—C11—C12—C13 | 0.9 (3) | C20—C21—C25—C30 | 176.2 (2) |
| N2—C15—C16—N4 | 34.3 (2) | C21—C22—C23—C18 | -2.0 (3) |
| N3—C5—C6—C7 | -2.0 (3) | C21—C22—C24—O1 | -176.5 (2) |
| N3—C5—C10—N4 | -18.9 (2) | C21—C22—C24—C30 | 2.5 (2) |
| N4—C17—C18—C19 | -97.1 (2) | C21—C25—C26—C27 | 177.7 (2) |
| N4—C17—C18—C23 | 81.9 (2) | C21—C25—C30—C24 | 2.6 (2) |
| C5—N3—C9—C8 | -1.3 (3) | C21—C25—C30—C29 | -177.91 (19) |
| C5—C6—C7—C8 | -0.2 (3) | C22—C21—C25—C26 | 179.9 (2) |
| C6—C5—C10—N4 | 166.80 (17) | C22—C21—C25—C30 | -0.9 (2) |
| C6—C7—C8—C9 | 1.6 (3) | C22—C24—C30—C25 | -3.1 (2) |
| C7—C8—C9—N3 | -0.8 (3) | C22—C24—C30—C29 | 177.4 (2) |
| C9—N3—C5—C6 | 2.8 (3) | C23—C18—C19—C20 | -1.4 (3) |
| C9—N3—C5—C10 | -171.43 (17) | C23—C22—C24—O1 | 5.6 (4) |
| C10—N4—C16—C15 | 77.11 (19) | C23—C22—C24— | -175.4 (2) |

| | | | |
|---|---|---|---|
| | | C30 | |
| C10—N4—C17—C18 | 54.0 (2) | C24—C22—C23—C18 | 175.67 (19) |
| C10—C5—C6—C7 | 172.07 (18) | C25—C21—C22—C23 | 176.99 (18) |
| C11—N2—C15—C14 | -2.5 (3) | C25—C21—C22—C24 | -1.1 (2) |
| C11—N2—C15—C16 | 173.21 (16) | C25—C26—C27—C28 | 0.6 (3) |
| C11—C12—C13—C14 | -0.9 (3) | C26—C25—C30—C24 | -178.18 (19) |
| C12—C13—C14—C15 | -0.7 (3) | C26—C25—C30—C29 | 1.4 (3) |
| C13—C14—C15—N2 | 2.5 (3) | C26—C27—C28—C29 | 0.2 (4) |
| C13—C14—C15—C16 | -172.95 (18) | C27—C28—C29—C30 | -0.2 (3) |
| C14—C15—C16—N4 | -150.02 (17) | C28—C29—C30—C24 | 178.9 (2) |
| C15—N2—C11—C12 | 0.8 (3) | C28—C29—C30—C25 | -0.5 (3) |
| C16—N4—C10—C5 | -97.33 (18) | C30—C25—C26—C27 | -1.4 (3) |
| C16—N4—C17—C18 | -67.6 (2) | | |

Document origin: *publCIF* [Westrip, S. P. (2010). *J. Apply. Cryst.*, **43**, 920-925].

## [($^{Fluor}N_3$)Ni($CF_3$)]$BF_4$

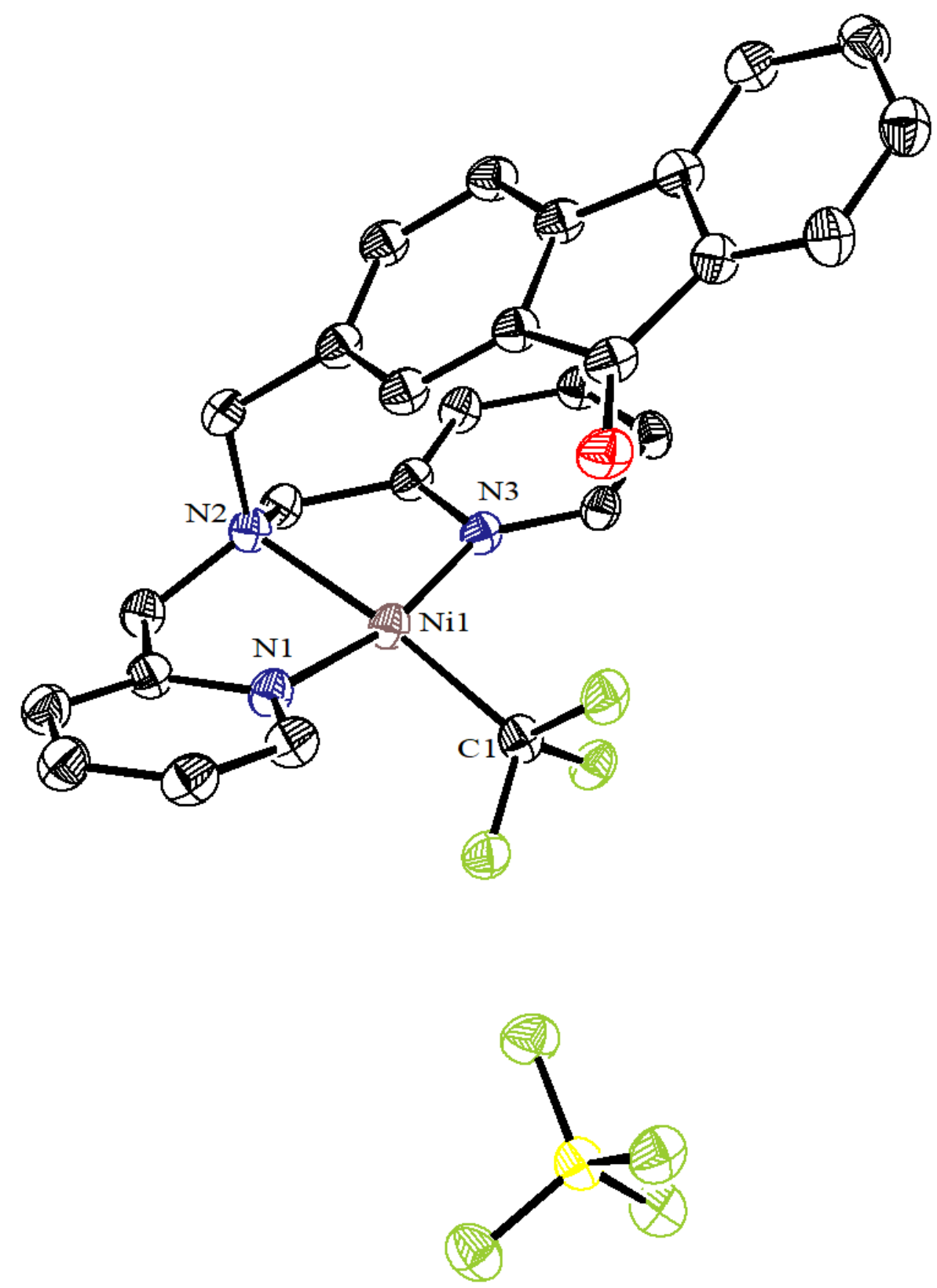


X-ray crystal structure of HH017. Hydrogen atoms omitted for clarity. Ellipsoids presented at 30% probability.

### Refinement

Crystal data, data collection and structure refinement details are summarized in Table 1.

### Results and discussion

### Computing details

Data collection: *CrysAlis PRO* 1.171.44.128a (Rigaku OD, 2025); cell refinement: *CrysAlis PRO* 1.171.44.128a (Rigaku OD, 2025); data reduction: *CrysAlis PRO* 1.171.44.128a (Rigaku OD, 2025); program(s) used to solve structure: SHELXT (Sheldrick, 2015); program(s) used to refine structure: *SHELXL* 2019/3 (Sheldrick, 2015); molecular graphics: Olex2 1.5-ac7-018 (Dolomanov *et al.*, 2009); software used to prepare material for publication: Olex2 1.5-ac7-018 (Dolomanov *et al.*, 2009).

### References


Dolomanov, O. V., Bourhis, L. J., Gildea, R. J., Howard, J. A. K. & Puschmann, H. (2009). *J. Appl.*

*Cryst.* **42**, 339–341.

Sheldrick, G. M. (2015). *Acta Cryst.* A**71**, 3–8.

Sheldrick, G. M. (2015). *Acta Cryst.* C**71**, 3–8.

**HH017**

*Crystal data*

| | |
|---|---|
| $C_{27}H_{21}F_3N_3NiO·BF_4$ | $F$(000) = 1232 |
| $M_r$ = 605.99 | $D_x$ = 1.605 Mg m$^{-3}$ |
| Monoclinic, $P2_1/c$ | Cu $K\alpha$ radiation, $\lambda$ = 1.54184 Å |
| $a$ = 8.4180 (6) Å | Cell parameters from 11019 reflections |
| $b$ = 13.1908 (10) Å | $\theta$ = 3.8–75.5° |
| $c$ = 22.716 (2) Å | $\mu$ = 1.84 mm$^{-1}$ |
| $\beta$ = 96.261 (8)° | $T$ = 100 K |
| $V$ = 2507.3 (4) Å$^3$ | Rhombohedral, clear light yellow |
| $Z$ = 4 | 0.05 × 0.05 × 0.04 mm |

*Data collection*

| | |
|---|---|
| XtaLAB Synergy, Dualflex, HyPix-Arc 150 diffractometer | 5060 independent reflections |
| Radiation source: micro-focus sealed X-ray tube, PhotonJet (Cu) X-ray Source | 3392 reflections with $I > 2\sigma(I)$ |
| Mirror monochromator | $R_{int}$ = 0.073 |
| Detector resolution: 10.0000 pixels mm$^{-1}$ | $\theta_{max}$ = 76.2°, $\theta_{min}$ = 3.9° |
| ω scans | $h$ = -10→10 |
| Absorption correction: multi-scan *CrysAlis PRO* 1.171.44.128a (Rigaku Oxford Diffraction, 2025) Empirical absorption correction using spherical harmonics, implemented in SCALE3 ABSPACK scaling algorithm. | $k$ = -16→14 |
| $T_{min}$ = 0.754, $T_{max}$ = 1.000 | $l$ = -27→27 |
| 33085 measured reflections | |

*Refinement*

| | |
|---|---|
| Refinement on $F^2$ | Hydrogen site location: inferred from neighbouring sites |

| | |
|---|---|
| Least-squares matrix: full | H-atom parameters constrained |
| $R[F^2 > 2\sigma(F^2)] = 0.056$ | $w = 1/[\sigma^2(F_o^2) + (0.0799P)^2 + 1.5996P]$ where $P = (F_o^2 + 2F_c^2)/3$ |
| $wR(F^2) = 0.169$ | $(\Delta/\sigma)_{max} = 0.001$ |
| $S = 1.09$ | $\Delta\rangle_{max} = 0.58$ e Å$^{-3}$ |
| 5060 reflections | $\Delta\rangle_{min} = -0.70$ e Å$^{-3}$ |
| 362 parameters | Extinction correction: *SHELXL2019*/2 (Sheldrick 2019), $Fc^*=kFc[1+0.001xFc^2\lambda^3/sin(2\theta)]^{-1/4}$ |
| 0 restraints | Extinction coefficient: 0.0012 (2) |
| Primary atom site location: dual | |

### *Special details*

| |
|---|
| *Geometry*. All esds (except the esd in the dihedral angle between two l.s. planes) are estimated using the full covariance matrix. The cell esds are taken into account individually in the estimation of esds in distances, angles and torsion angles; correlations between esds in cell parameters are only used when they are defined by crystal symmetry. An approximate (isotropic) treatment of cell esds is used for estimating esds involving l.s. planes. |

### *Fractional atomic coordinates and isotropic or equivalent isotropic displacement parameters (Å$^2$) for (hh017_auto)*

| | *x* | *y* | *z* | $U_{iso}$*/$U_{eq}$ |
|---|---|---|---|---|
| Ni1 | 0.31282 (7) | 0.66727 (5) | 0.60753 (3) | 0.0356 (2) |
| F1 | 0.5647 (2) | 0.53961 (18) | 0.64785 (10) | 0.0471 (6) |
| F2 | 0.4242 (2) | 0.48347 (17) | 0.57054 (10) | 0.0450 (6) |
| F3 | 0.5911 (2) | 0.60679 (18) | 0.56285 (11) | 0.0483 (6) |
| O1 | 0.7243 (3) | 0.8493 (2) | 0.51668 (13) | 0.0468 (7) |
| N1 | 0.4132 (3) | 0.7256 (2) | 0.67857 (14) | 0.0388 (7) |
| N2 | 0.1292 (3) | 0.7496 (2) | 0.62427 (14) | 0.0347 (7) |
| N3 | 0.1872 (3) | 0.6355 (2) | 0.53548 (14) | 0.0345 (7) |
| C1 | 0.4769 (4) | 0.5722 (3) | 0.59727 (17) | 0.0384 (9) |
| C2 | 0.5713 (4) | 0.7419 (3) | 0.69350 (19) | 0.0440 (10) |
| H2 | 0.645438 | 0.716893 | 0.668347 | 0.053* |
| C3 | 0.6274 (5) | 0.7936 (3) | 0.7441 (2) | 0.0491 (10) |
| H3 | 0.738937 | 0.803468 | 0.753809 | 0.059* |
| C4 | 0.5211 (5) | 0.8309 (3) | 0.7805 (2) | 0.0499 (11) |
| H4 | 0.558135 | 0.866214 | 0.815791 | 0.060* |
| C5 | 0.3578 (5) | 0.8159 (3) | 0.76479 (19) | 0.0472 (10) |

| | | | | |
|---|---|---|---|---|
| H5 | 0.282207 | 0.841374 | 0.789197 | 0.057* |
| C6 | 0.3078 (4) | 0.7644 (3) | 0.71396 (18) | 0.0400 (9) |
| C7 | 0.1350 (4) | 0.7483 (3) | 0.68983 (17) | 0.0404 (9) |
| H7A | 0.096031 | 0.682423 | 0.703452 | 0.048* |
| H7B | 0.067266 | 0.802934 | 0.703480 | 0.048* |
| C8 | -0.0136 (4) | 0.6948 (3) | 0.59488 (18) | 0.0381 (9) |
| H8A | -0.106143 | 0.741347 | 0.588714 | 0.046* |
| H8B | -0.042092 | 0.637636 | 0.619931 | 0.046* |
| C9 | 0.0287 (4) | 0.6562 (3) | 0.53653 (17) | 0.0353 (8) |
| C10 | -0.0807 (4) | 0.6405 (3) | 0.48770 (18) | 0.0387 (9) |
| H10 | -0.191205 | 0.652030 | 0.490058 | 0.046* |
| C11 | -0.0286 (4) | 0.6080 (3) | 0.43530 (18) | 0.0405 (9) |
| H11 | -0.102522 | 0.596912 | 0.401241 | 0.049* |
| C12 | 0.1333 (4) | 0.5917 (3) | 0.43322 (18) | 0.0416 (9) |
| H12 | 0.172263 | 0.571213 | 0.397366 | 0.050* |
| C13 | 0.2372 (4) | 0.6057 (3) | 0.48382 (17) | 0.0360 (8) |
| H13 | 0.347993 | 0.593972 | 0.482214 | 0.043* |
| C14 | 0.1348 (4) | 0.8575 (3) | 0.60396 (18) | 0.0396 (9) |
| H14A | 0.197598 | 0.897722 | 0.635133 | 0.048* |
| H14B | 0.024748 | 0.884986 | 0.599156 | 0.048* |
| C15 | 0.2070 (4) | 0.8712 (3) | 0.54645 (18) | 0.0382 (9) |
| C16 | 0.1134 (5) | 0.8898 (3) | 0.49369 (19) | 0.0427 (9) |
| H16 | 0.000777 | 0.893530 | 0.493794 | 0.051* |
| C17 | 0.1790 (4) | 0.9032 (3) | 0.43987 (19) | 0.0425 (9) |
| H17 | 0.112534 | 0.917456 | 0.404297 | 0.051* |
| C18 | 0.3430 (4) | 0.8952 (3) | 0.44016 (18) | 0.0395 (9) |
| C19 | 0.4470 (4) | 0.9052 (3) | 0.39218 (18) | 0.0411 (9) |
| C20 | 0.4141 (5) | 0.9327 (3) | 0.33389 (19) | 0.0460 (10) |
| H20 | 0.307814 | 0.947465 | 0.317607 | 0.055* |
| C21 | 0.5413 (5) | 0.9385 (3) | 0.2989 (2) | 0.0496 (10) |
| H21 | 0.521030 | 0.958401 | 0.258609 | 0.060* |
| C22 | 0.6969 (5) | 0.9154 (3) | 0.3224 (2) | 0.0497 (11) |
| H22 | 0.781208 | 0.918106 | 0.297845 | 0.060* |
| C23 | 0.7290 (5) | 0.8888 (3) | 0.38079 (19) | 0.0468 (10) |
| H23 | 0.835196 | 0.873550 | 0.396931 | 0.056* |
| C24 | 0.6049 (4) | 0.8845 (3) | 0.41599 (18) | 0.0402 (9) |
| C25 | 0.6081 (4) | 0.8667 (3) | 0.48051 (19) | 0.0395 (9) |

| | | | | |
|---|---|---|---|---|
| C26 | 0.4382 (4) | 0.8744 (3) | 0.49352 (17) | 0.0362 (8) |
| C27 | 0.3747 (4) | 0.8647 (3) | 0.54674 (18) | 0.0400 (9) |
| H27 | 0.441864 | 0.853926 | 0.582589 | 0.048* |
| F4 | 0.9108 (3) | 0.53366 (19) | 0.70225 (11) | 0.0527 (6) |
| F5 | 1.1779 (2) | 0.50646 (18) | 0.70233 (11) | 0.0504 (6) |
| F6 | 1.0075 (3) | 0.37301 (19) | 0.70383 (11) | 0.0512 (6) |
| F7 | 1.0544 (3) | 0.4732 (2) | 0.78508 (10) | 0.0527 (6) |
| B1 | 1.0376 (5) | 0.4719 (4) | 0.7236 (2) | 0.0403 (10) |

*Atomic displacement parameters (Å$^2$) for (hh017_auto)*

| | $U^{11}$ | $U^{22}$ | $U^{33}$ | $U^{12}$ | $U^{13}$ | $U^{23}$ |
|---|---|---|---|---|---|---|
| Ni1 | 0.0254 (3) | 0.0405 (4) | 0.0414 (4) | 0.0008 (3) | 0.0053 (2) | -0.0032 (3) |
| F1 | 0.0389 (12) | 0.0560 (15) | 0.0451 (14) | 0.0105 (10) | -0.0013 (10) | -0.0027 (11) |
| F2 | 0.0346 (11) | 0.0449 (13) | 0.0546 (14) | 0.0051 (10) | 0.0011 (10) | -0.0075 (11) |
| F3 | 0.0328 (11) | 0.0566 (15) | 0.0581 (15) | 0.0011 (10) | 0.0166 (10) | 0.0009 (12) |
| O1 | 0.0279 (13) | 0.0555 (18) | 0.0561 (18) | 0.0039 (12) | 0.0006 (12) | 0.0053 (14) |
| N1 | 0.0282 (14) | 0.0436 (19) | 0.0451 (19) | 0.0021 (13) | 0.0059 (13) | -0.0027 (15) |
| N2 | 0.0257 (13) | 0.0393 (18) | 0.0395 (18) | -0.0016 (12) | 0.0053 (12) | -0.0021 (14) |
| N3 | 0.0258 (14) | 0.0369 (17) | 0.0416 (18) | -0.0004 (12) | 0.0081 (12) | 0.0026 (14) |
| C1 | 0.0286 (17) | 0.049 (2) | 0.038 (2) | -0.0040 (16) | 0.0048 (15) | -0.0016 (18) |
| C2 | 0.0334 (19) | 0.046 (2) | 0.052 (3) | -0.0038 (17) | -0.0010 (17) | -0.001 (2) |
| C3 | 0.039 (2) | 0.048 (3) | 0.058 (3) | -0.0004 (18) | -0.0047 (19) | -0.003 (2) |
| C4 | 0.047 (2) | 0.049 (3) | 0.052 (3) | 0.0025 (19) | -0.0041 (19) | -0.010 (2) |
| C5 | 0.043 (2) | 0.049 (3) | 0.049 (2) | 0.0055 (18) | 0.0019 (18) | -0.004 (2) |
| C6 | 0.0367 (19) | 0.042 (2) | 0.041 (2) | -0.0001 (16) | 0.0043 (16) | -0.0002 (18) |
| C7 | 0.0311 (18) | 0.045 (2) | 0.046 (2) | -0.0005 (16) | 0.0083 (16) | -0.0014 (18) |
| C8 | 0.0249 (16) | 0.044 (2) | 0.047 (2) | 0.0005 (15) | 0.0069 (15) | -0.0015 (18) |
| C9 | 0.0264 (16) | 0.036 (2) | 0.044 (2) | -0.0012 (14) | 0.0067 (15) | 0.0012 (17) |
| C10 | 0.0273 (16) | 0.042 (2) | 0.047 (2) | -0.0026 (15) | 0.0045 (15) | -0.0045 (18) |
| C11 | 0.0321 (18) | 0.046 (2) | 0.042 (2) | 0.0012 (16) | -0.0003 (16) | -0.0019 (18) |
| C12 | 0.0371 (19) | 0.049 (2) | 0.039 (2) | -0.0019 (17) | 0.0074 (16) | -0.0032 (19) |
| C13 | 0.0276 (16) | 0.040 (2) | 0.041 (2) | 0.0024 (15) | 0.0068 (15) | -0.0024 (17) |
| C14 | 0.0348 (18) | 0.037 (2) | 0.047 (2) | 0.0042 (16) | 0.0060 (16) | -0.0038 (18) |
| C15 | 0.0280 (17) | 0.039 (2) | 0.048 (2) | 0.0005 (15) | 0.0075 (16) | 0.0013 (18) |
| C16 | 0.0344 (19) | 0.042 (2) | 0.052 (3) | 0.0010 (16) | 0.0049 (17) | 0.0020 (19) |
| C17 | 0.0289 (17) | 0.047 (2) | 0.050 (2) | 0.0033 (16) | 0.0006 (16) | 0.0060 (19) |

| | | | | | | |
|---|---|---|---|---|---|---|
| C18 | 0.0304 (17) | 0.040 (2) | 0.047 (2) | 0.0004 (16) | 0.0016 (16) | 0.0027 (18) |
| C19 | 0.0349 (19) | 0.041 (2) | 0.048 (2) | -0.0036 (16) | 0.0064 (17) | 0.0032 (19) |
| C20 | 0.039 (2) | 0.048 (3) | 0.051 (3) | -0.0008 (18) | 0.0007 (18) | 0.000 (2) |
| C21 | 0.058 (3) | 0.047 (3) | 0.045 (2) | -0.008 (2) | 0.010 (2) | 0.001 (2) |
| C22 | 0.047 (2) | 0.050 (3) | 0.054 (3) | -0.0087 (19) | 0.015 (2) | -0.005 (2) |
| C23 | 0.0347 (19) | 0.055 (3) | 0.052 (3) | -0.0075 (18) | 0.0093 (18) | -0.004 (2) |
| C24 | 0.0354 (18) | 0.042 (2) | 0.044 (2) | -0.0031 (16) | 0.0059 (16) | -0.0014 (18) |
| C25 | 0.0270 (17) | 0.038 (2) | 0.053 (2) | -0.0030 (15) | 0.0025 (16) | 0.0044 (18) |
| C26 | 0.0266 (16) | 0.039 (2) | 0.043 (2) | -0.0004 (15) | 0.0049 (15) | 0.0030 (17) |
| C27 | 0.0294 (18) | 0.042 (2) | 0.048 (2) | 0.0000 (16) | 0.0027 (16) | 0.0016 (18) |
| F4 | 0.0341 (11) | 0.0650 (16) | 0.0587 (15) | 0.0057 (11) | 0.0034 (10) | 0.0110 (13) |
| F5 | 0.0321 (11) | 0.0548 (15) | 0.0660 (16) | -0.0021 (10) | 0.0126 (11) | 0.0053 (12) |
| F6 | 0.0460 (13) | 0.0543 (15) | 0.0527 (15) | -0.0088 (11) | 0.0027 (11) | 0.0003 (12) |
| F7 | 0.0428 (12) | 0.0709 (17) | 0.0435 (14) | -0.0068 (11) | 0.0004 (10) | -0.0002 (12) |
| B1 | 0.033 (2) | 0.047 (3) | 0.042 (2) | -0.0070 (19) | 0.0056 (18) | -0.001 (2) |

*Geometric parameters (Å, º) for (hh017_auto)*

| | | | |
|---|---|---|---|
| Ni1—N1 | 1.900 (3) | C11—C12 | 1.385 (5) |
| Ni1—N2 | 1.960 (3) | C12—C13 | 1.379 (5) |
| Ni1—N3 | 1.896 (3) | C14—C15 | 1.511 (5) |
| Ni1—C1 | 1.899 (4) | C15—C16 | 1.382 (5) |
| F1—C1 | 1.366 (4) | C15—C27 | 1.414 (5) |
| F2—C1 | 1.370 (4) | C16—C17 | 1.406 (6) |
| F3—C1 | 1.381 (4) | C17—C18 | 1.384 (5) |
| O1—C25 | 1.228 (4) | C18—C19 | 1.477 (5) |
| N1—C2 | 1.354 (4) | C18—C26 | 1.405 (5) |
| N1—C6 | 1.360 (5) | C19—C20 | 1.372 (6) |
| N2—C7 | 1.485 (5) | C19—C24 | 1.406 (5) |
| N2—C8 | 1.496 (4) | C20—C21 | 1.404 (6) |
| N2—C14 | 1.498 (5) | C21—C22 | 1.393 (6) |
| N3—C9 | 1.364 (4) | C22—C23 | 1.369 (6) |
| N3—C13 | 1.348 (5) | C23—C24 | 1.384 (5) |
| C2—C3 | 1.375 (6) | C24—C25 | 1.482 (6) |
| C3—C4 | 1.374 (6) | C25—C26 | 1.495 (5) |
| C4—C5 | 1.396 (6) | C26—C27 | 1.380 (5) |
| C5—C6 | 1.366 (6) | F4—B1 | 1.388 (5) |

| | | | |
|---|---|---|---|
| C6—C7 | 1.513 (5) | F5—B1 | 1.400 (5) |
| C8—C9 | 1.499 (5) | F6—B1 | 1.394 (5) |
| C9—C10 | 1.378 (5) | F7—B1 | 1.388 (5) |
| C10—C11 | 1.381 (5) | | |
| | | | |
| N1—Ni1—N2 | 84.14 (13) | C9—C10—C11 | 119.5 (3) |
| N3—Ni1—N1 | 167.63 (13) | C10—C11—C12 | 118.9 (4) |
| N3—Ni1—N2 | 84.98 (13) | C13—C12—C11 | 119.2 (4) |
| N3—Ni1—C1 | 95.34 (15) | N3—C13—C12 | 122.3 (3) |
| C1—Ni1—N1 | 96.29 (15) | N2—C14—C15 | 114.1 (3) |
| C1—Ni1—N2 | 171.67 (15) | C16—C15—C14 | 121.7 (3) |
| C2—N1—Ni1 | 127.6 (3) | C16—C15—C27 | 119.3 (4) |
| C2—N1—C6 | 118.6 (3) | C27—C15—C14 | 119.0 (3) |
| C6—N1—Ni1 | 113.3 (2) | C15—C16—C17 | 122.4 (3) |
| C7—N2—Ni1 | 104.3 (2) | C18—C17—C16 | 118.2 (4) |
| C7—N2—C8 | 112.2 (3) | C17—C18—C19 | 131.4 (4) |
| C7—N2—C14 | 108.8 (3) | C17—C18—C26 | 119.6 (4) |
| C8—N2—Ni1 | 104.9 (2) | C26—C18—C19 | 109.0 (3) |
| C8—N2—C14 | 111.9 (3) | C20—C19—C18 | 131.5 (4) |
| C14—N2—Ni1 | 114.5 (2) | C20—C19—C24 | 120.4 (4) |
| C9—N3—Ni1 | 113.3 (2) | C24—C19—C18 | 108.1 (3) |
| C13—N3—Ni1 | 128.2 (2) | C19—C20—C21 | 118.3 (4) |
| C13—N3—C9 | 118.2 (3) | C22—C21—C20 | 121.0 (4) |
| F1—C1—Ni1 | 116.0 (3) | C23—C22—C21 | 120.3 (4) |
| F1—C1—F2 | 102.9 (3) | C22—C23—C24 | 119.2 (4) |
| F1—C1—F3 | 103.6 (3) | C19—C24—C25 | 109.0 (3) |
| F2—C1—Ni1 | 114.4 (2) | C23—C24—C19 | 120.7 (4) |
| F2—C1—F3 | 104.1 (3) | C23—C24—C25 | 130.1 (4) |
| F3—C1—Ni1 | 114.3 (3) | O1—C25—C24 | 128.2 (4) |
| N1—C2—C3 | 121.8 (4) | O1—C25—C26 | 126.1 (4) |
| C4—C3—C2 | 119.6 (4) | C24—C25—C26 | 105.7 (3) |
| C3—C4—C5 | 118.9 (4) | C18—C26—C25 | 108.1 (3) |
| C6—C5—C4 | 119.4 (4) | C27—C26—C18 | 122.3 (3) |
| N1—C6—C5 | 121.7 (4) | C27—C26—C25 | 129.6 (3) |
| N1—C6—C7 | 113.3 (3) | C26—C27—C15 | 118.2 (4) |
| C5—C6—C7 | 124.9 (4) | F4—B1—F5 | 109.5 (4) |
| N2—C7—C6 | 106.7 (3) | F4—B1—F6 | 109.3 (3) |

| | | | |
|---|---|---|---|
| N2—C8—C9 | 107.6 (3) | F4—B1—F7 | 109.6 (4) |
| N3—C9—C8 | 114.2 (3) | F6—B1—F5 | 108.9 (3) |
| N3—C9—C10 | 121.7 (3) | F7—B1—F5 | 110.2 (3) |
| C10—C9—C8 | 124.1 (3) | F7—B1—F6 | 109.3 (4) |

Document origin: *publCIF* [Westrip, S. P. (2010). *J. Apply. Cryst.*, **43**, 920-925].

## XXIII. References.


1. Pangborn, A. B.; Giardello, M. A.; Grubbs, R. H.; Rosen, R. K.; Timmers, F. J. Safe and Convenient Procedure for Solvent Purification. *Organometallics* **1996**, *15*, 1518–1520.
2. Li, J. F.; Chen, Q. Y. Synthesis, Spectroscopic Characterization and Hydroxylation of Mn(II) Complexes with Bis(2-pyridylmethyl)benzylamine. *Spectrochim. Acta, Part A* **2009**, *73*, 25–28.
3. Zhang, C.-P.; Wang, H.; Biewer, C.; Stirnat, K.; Yamaguchi, T.; Xu, L.; Gomez-Benitez, V.; Vicic, D. A. A Five-Coordinate Nickel(II) Fluoroalkyl Complex as a Precursor to a Spectroscopically Detectable Ni(III) Species. *J. Am. Chem. Soc.* **2013**, *135*, 8141–8144.
4. Kiruthika, S. E.; Perumal, P. T. CuI-catalyzed Coupling of Gem-dibromovinylanilides and Sulfonamides: an Efficient Method for the Synthesis of 2-amidoindoles and Indolo[1,2-a]quinazolines. *Org. Lett*. **2014**, *16*, 484–487.
5. Gannon, S. M.; Krause, J. G. Phase-transfer Permanganate Oxidation of Unfunctionalized Benzylic Positions. *Synthesis* **1987**, *10,* 915–917.
6. Tang, F. Z.; Rath, N. P.; Mirica, L. M. Stable Bis(trifluoromethyl)nickel(III) Complexes. *Chem. Commun.* **2015**, *51*, 3113– 3116
7. a) Bour, J. R.; Camasso, N. M.; Meucci, E. A.; Kampf, J. W.; Canty, A. J.; Sanford, M. S. Carbon–Carbon Bond-Forming Reductive Elimination from Isolated Nickel(III) Complexes. *J. Am. Chem. Soc.* **2016**, *138*, 16105– 16111. b) Elgrishi, N.; Rountree, K. J.; McCarthy, B. D.; Rountree, E. S.; Eisenhart, T. T.; Dempsey, J. L. A Practical Beginner's Guide to Cyclic Voltammetry. *J. Chem. Educ.* **2018**, *95*, 197– 206.
8. Granados, D. A.; Du, Y. E.; Andersson, S. J.; Cirincione-Lynch, A.; Cui, K.; Reinhold, A.; Jeffrey, P. D.; Scholes, G. D.; Hammes-Schiffer, S.; Knowles, R. R. Iridium Polypyridyl Carboxylates as Excited-State PCET Catalysts for the Functionalization of Unactivated C–H Bonds. *J. Am. Chem. Soc.* **2025**, *147*, 20703– 20715.
9. van Stokkum, I. H. M.; Larsen, D. S.; van Grondelle, R. Global and Target Analysis of Time-Resolved Spectra. *Biochim. Biophys. Acta, Bioenerg.***2004**, *1657* (2–3), 82– 104.
10. Holzwarth, A. R. Data Analysis of Time-Resolved Spectra. In *Biophysical Techniques in Photosynthesis*; Amesz, J., Hoff, A. J., Eds.; Springer: Dordrecht, The Netherlands, **1996**; pp 75– 92.
11. Doust, A.B.; van Stokkum, I. H. M.; Larsen, D.S.; Wilk, K. E.; Curmi, P.M.G.; van Grondelle, R.; Scholes, G.D. Mediation of Ultrafast Light-Harvesting by a Central Dimer in Phycoerythrin 545 Studied by Transient Absorption and Global Analysis. *J.P.C. B.,* **2005**, *109*, 14219 – 14226.
12. Alfano, R. R.; Govindjee, W. Y. R.; Becher, B.; Ebrey, T. G. Picosecond Kinetics of Fluorescence from Chromophore of Purple Membrane-Protein of Halobacterium-Halobium. *Biophys. J.* **1976**, *16*, 541– 545.
13. Lee, T.S.; Lin, Y.L.; Kim, H.; Pensack, R.D.; Rand, B.P.; Scholes, G.D. Triplet Energy Transfer Governs the Dissociation of the Correlated Triplet Pair in Exothermic Singlet Fission. *J.P.C.L.* **2018,** *9*, 4087 – 4095.
14. Snellenburg, J.J.; Laptenok, S.P.; Seger, R.; Mullen, K.M.; van Stokkum, I.H.M. Glotaran: A Java-Based Graphical User Interface for the R Package TIMP. *J. Stat. Soft.,* **2012**, *49*, 1-2.
15. Frisch, M. J.; Trucks, G. W.; Schlegel, H. B.; Scuseria, G. E.; Robb, M. A.; Cheeseman, J. R.; Scalmani, G.; Barone, V.; Petersson, G. A.; Nakatsuji, H.; Li, X.; Caricato, M.; Marenich, A. V.; Bloino, J.; Janesko, B. G.; Gomperts, R.; Mennucci, B.; Hratchian, H. P.; Ortiz, J. V.; Izmaylov, A. F.; Sonnenberg, J. L.; Williams-Young, D.; Ding, F.; Lipparini, F.; Egidi, F.; Goings, J.; Peng, B.; Petrone, A.; Henderson, T.; Ranasinghe, D.; Zakrzewski, V. G.; Gao, J.; Rega, N.; Zheng, G.; Liang, W.; Hada, M.; Ehara, M.; Toyota, K.; Fukuda, R.; Hasegawa, J.; Ishida, M.; Nakajima, T.; Honda, Y.; Kitao, O.; Nakai, H.; Vreven, T.; Throssell, K.; Montgomery, J. A., Jr.; Peralta, J. E.; Ogliaro, F.; Bearpark, M. J.; Heyd, J. J.; Brothers, E. N.; Kudin, K. N.; Staroverov, V. N.; Keith, T. A.; Kobayashi, R.; Normand, J.; Raghavachari, K.; Rendell, A. P.; Burant, J. C.; Iyengar, S. S.; Tomasi, J.; Cossi, M.; Millam, J. M.; Klene, M.; Adamo, C.; Cammi, R.; Ochterski, J. W.; Martin,

R. L.; Morokuma, K.; Farkas, O.; Foresman, *Gaussian 16*, revision C.01; Gaussian, Inc.: Wallingford CT, **2016**.

16. Schafer, A.; Huber, C.; Ahlrichs, R. Fully Optimized Contracted Gaussian Basis Sets of Triple Zeta Valence Quality for Atoms Li to Kr. *J. Chem. Phys.* **1994**, *100*, 5829−5835.
17. Zhao, Y.; Truhlar, D. The M06 Suite of Density Functionals for Main Group Thermochemistry, Thermochemical Kinetics, Noncovalent Interactions, Excited States, and Transition Elements: Two New Functionals and Systematic Testing of Four M06-Class Functionals and 12 Other Functionals. *Theor. Chem. Acc.* **2008**, *120*, 215−241.
18. Pettersen, E. F.; Goddard, T. D.; Huang, C. C.; Meng, E. C.; Couch, G. S.; Cross, T. I.; Morris, J. H.; Ferrin, T. E. UCSF ChimeraX: Structure Visualization for Researchers, Educators, and Developers. *Protein Sci.* **2021**, *30*, 70−82.
19. Miertuš, S.; Scrocco, E.; Tomasi, J. Electrostatic Interaction of a Solute with a Continuum. A Direct Utilizaion of AB Initio Molecular Potentials for the Prevision of Solvent Effects. *Chem. Phys.* **1981**, *55*, 117– 129.
20. Stoll, S.; Schweiger, A. EasySpin, a comprehensive software package for spectral simulation and analysis in EPR. *Journal of Magnetic Resonance* **2006**, *178*, 42-55.
21. *Rowan Scientific*. https://www.rowansci.com (accessed 2026-07-10).
22. Levine, D. S.; Shuaibi, M.; Spotte-Smith, W. C.; Taylor, M. G.; Hasyim, M. R.; Michel, K.; Batatia, I.; Csányi, G.; Dzamba, M.; Eastman, P.; Frey, N. C.; Fu, X.; Gharakhanyan, V.; Krishnapriyan, A. S.; Rackers, J. A.; Raja, S.; Rizvi, A.; Rosen, A. S.; Ulissi, Z.; Vargas, S. *Open Molecules 2025 (OMol25) Dataset, Evaluations, and Models*, **2025**, arXiv:2505.08762. arXiv.org e-Print archive. https://arxiv.org/abs/2505.08762 (accessed 2026-07-10).
23. Wang, L.-P.; Song, C. Geometry Optimization Made Simple with Translation and Rotation Coordinates. *J. Chem, Phys.* **2016**, *144*, 214108.
24. Lu, T.; Chen, F. Multiwfn: A Multifunctional Wavefunction Analyzer. *J. Comput. Chem*. **2012**, *33*, 580–592. DOI: 10.1002/jcc.22885
25. Hirshfeld, F. L. Bonded-Atom Fragments for Describing Molecular Charge Densities. *Theor. Chim. Acta* **1977**, *44*, 129–138.
